# Single-chain fluorinated Ir-NHC catalysts for efficient SABRE pyruvate hyperpolarization with average residual iridium below 100 ppb

**DOI:** 10.1039/d6ra07354a

**Published:** 2026-10-12

**Authors:** Federico Floreani, Ahmed Faramawy, Federico De Biasi, Denis Badocco, Salvatore Mamone, Callum A. Gater, Simon B. Duckett, Cristina Tubaro, Gabriele Stevanato

**Affiliations:** a Department of Chemical Sciences, Università di Padova Via Marzolo 1 35131 Padova Italy cristina.tubaro@unipd.it gabriele.stevanato@unipd.it; b Department MESVA (Life, Health & Environmental Sciences), Università dell'Aquila Via Vetoio SNC, Località Coppito 67100 L'Aquila Italy; c Centre for Hyperpolarisation in Magnetic Resonance, University of York Heslington York YO10 5NY UK

## Abstract

Signal Amplification By Reversible Exchange (SABRE) delivers rapid and inexpensive NMR signal enhancement using *para*hydrogen-derived spin order, but biomedical translation requires catalysts that simultaneously deliver high polarization efficiency together with minimal residual metal contamination. Here we introduce a class of single-chain fluorinated Ir-NHC catalysts for SABRE hyperpolarization of [1-^13^C]pyruvate that addresses this longstanding trade-off. Retention of carbene backbone unsaturation preserves SABRE efficiency, enabling ∼4% ^13^C polarization with 50% *para*hydrogen, comparable to the benchmark Ir-IMes catalyst and approximately threefold higher than the previously reported perfluorinated Ir-SIMesF_2_ system under identical conditions. Combined with redissolution SABRE, aqueous extraction, and fluorinated silica filtration, one of these catalysts, Ir-IMesF_1_, yields aqueous hyperpolarized solutions containing <100 ppb residual iridium. The resulting aqueous formulations, while not yet suitable for direct biomedical administration, deliver efficient SABRE hyperpolarization and ultra-low residual iridium contamination, enabling real-time monitoring of *in vitro* pyruvate-to-lactate conversion under partial *para*hydrogen enrichment.

## Abstract 2

Efficient SABRE for biomedical use requires minimal metal contamination. We report a new class of single-chain fluorinated Ir–NHC catalysts; one matches the performance of IMes for ^13^C pyruvate hyperpolarization and enables Ir removal to <100 ppb.

## Graphical abstract

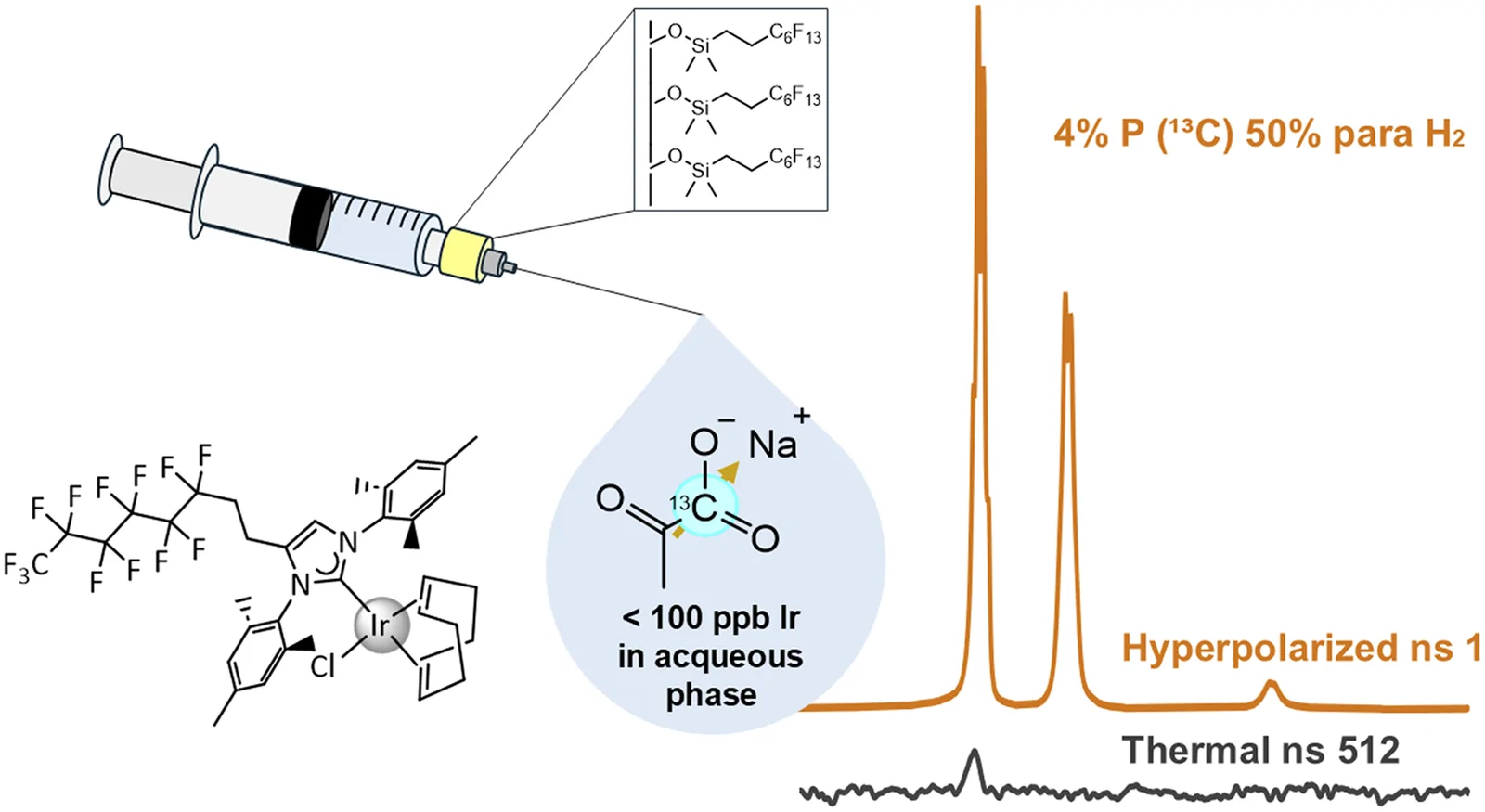

## Introduction

Hyperpolarization techniques enhance the sensitivity of NMR and MRI by increasing nuclear spin polarization by several orders of magnitude.^1^ The *in vivo* detection of hyperpolarized pyruvate, a central metabolic biomarker able to report disease-associated metabolic alterations, is a compelling application for this approach in biomedical imaging.^2–4^ Pyruvate hyperpolarization is most commonly achieved by dissolution Dynamic Nuclear Polarization^5^ (dDNP), which has found clinical utility, with ParaHydrogen-Induced Polarization Side-Arm Hydrogenation^6–9^ (PHIP-SAH), and more recently Signal Amplification By Reversible Exchange (SABRE)^10–30^ where hyperpolarization strategies provide access to preclinical *in vivo* data. Within the class of *para*hydrogen (*p*H_2_)-based methods, SABRE enables hyperpolarization through reversible coordination of the substrate to an iridium catalyst without its chemical modification. While its homogeneous catalytic mechanism enables rapid and efficient polarization transfer, it also necessitates catalyst removal before biomedical applications. For *in vivo* use, hyperpolarized pyruvate must be obtained in an aqueous, biocompatible formulation, with clinically acceptable residual metal content. According to ICH Q3D(R2) guidelines on elemental impurities, the parenteral Permitted Daily Exposure (PDE) for Ir is approximately 10.0 µg per day; iridium compounds have been associated with reduced cell viability and proliferation, oxidative DNA damage, and renal tubular dysfunction, although the effects of chronic low-dose exposure remain poorly defined.^31,32^

This creates a fundamental challenge for biomedical SABRE: catalysts are optimized for operation in organic solvents to maximize polarization efficiency, whereas *in vivo* applications require aqueous injectable formulations with minimal residual metal contamination.

One possible strategy to overcome this is to hyperpolarize substrates directly in water. However, direct aqueous SABRE has so far delivered substantially lower polarization, primarily because hydrogen solubility is approximately five times lower in water than in methanol (0.8 mM *versus* ∼4 mM at 25 °C and 1 bar).^33,34^ An alternative strategy would be to perform hyperpolarization in an organic solvent and subsequently change the solvent and remove the catalyst before administration. Immobilized heterogeneous catalysts represent one possible solution to catalyst removal, although they are poorly compatible with bidentate substrates such as pyruvate, generally afford lower enhancements than homogeneous systems and can be prone to catalyst leaching into the solvent.^35–37^ Importantly, the combination of phase extraction and catalyst capture has been shown to reduce residual Ir contamination to below 1 ppb for hyperpolarized ^15^N-pyridine, and related purification approaches have subsequently been extended to ^15^N-metronidazole.^38,39^ Whether comparable purification efficiency can be achieved for [1-^13^C]pyruvate remains to be established. Currently, approaches such as Ace-SABRE^27^ and catalyst-separated hyperpolarization^40^ (CASH) typically employ the Ir-IMes catalyst system and combine polarization in organic solvents with redissolution^24^ (ReD) to reduce residual Ir levels in aqueous solutions to the ppm or high-ppb range. However, catalyst architectures that simultaneously preserve high polarization efficiency while facilitating production of low-iridium aqueous formulations remain scarce. While Wacker *et al.* have explored modified NHC scaffolds such as Ir-TMes to enhance catalyst stability for pyruvate hyperpolarization, systems designed for efficient catalyst separation remain limited.^41^ Recently, the solubility characteristics of the catalyst Ir-SIMesF_2_, containing the perfluorinated double-chain functionalised N-heterocyclic carbene (NHC) ligand have enabled residual Ir concentrations of ∼177 ppb in aqueous hyperpolarized solutions.^15,42^ This double-chain perfluorinated NHC ligand departs from the parent IMes scaffold by replacing the unsaturated carbene backbone with a saturated analogue, a structural modification previously associated with reduced pyruvate hyperpolarization efficiency.^19,26^

Here we introduce a new class of single-chain fluorinated NHC ligands, comprising the unsaturated IMesF_1_ and IPrF_1_ derivatives and the saturated analogue SIMesF_1_ (Scheme 1). These systems enable efficient catalyst separation, while the unsaturated derivatives retain the carbene backbone required for high SABRE efficiency. Using Ir-IMesF_1_, we achieve threefold higher ^13^C polarization in [1-^13^C]pyruvate dissolved in methanol-d_4_ relative to Ir-SIMesF_2_ under identical experimental conditions, while its fluorinated design combined with the optimized extraction/filtration workflow reduces residual Ir in the final aqueous solution to below 100 ppb. In doing so, this rational ligand architecture reconciles high SABRE efficiency with stringent metal purity requirements.

**Scheme 1:**
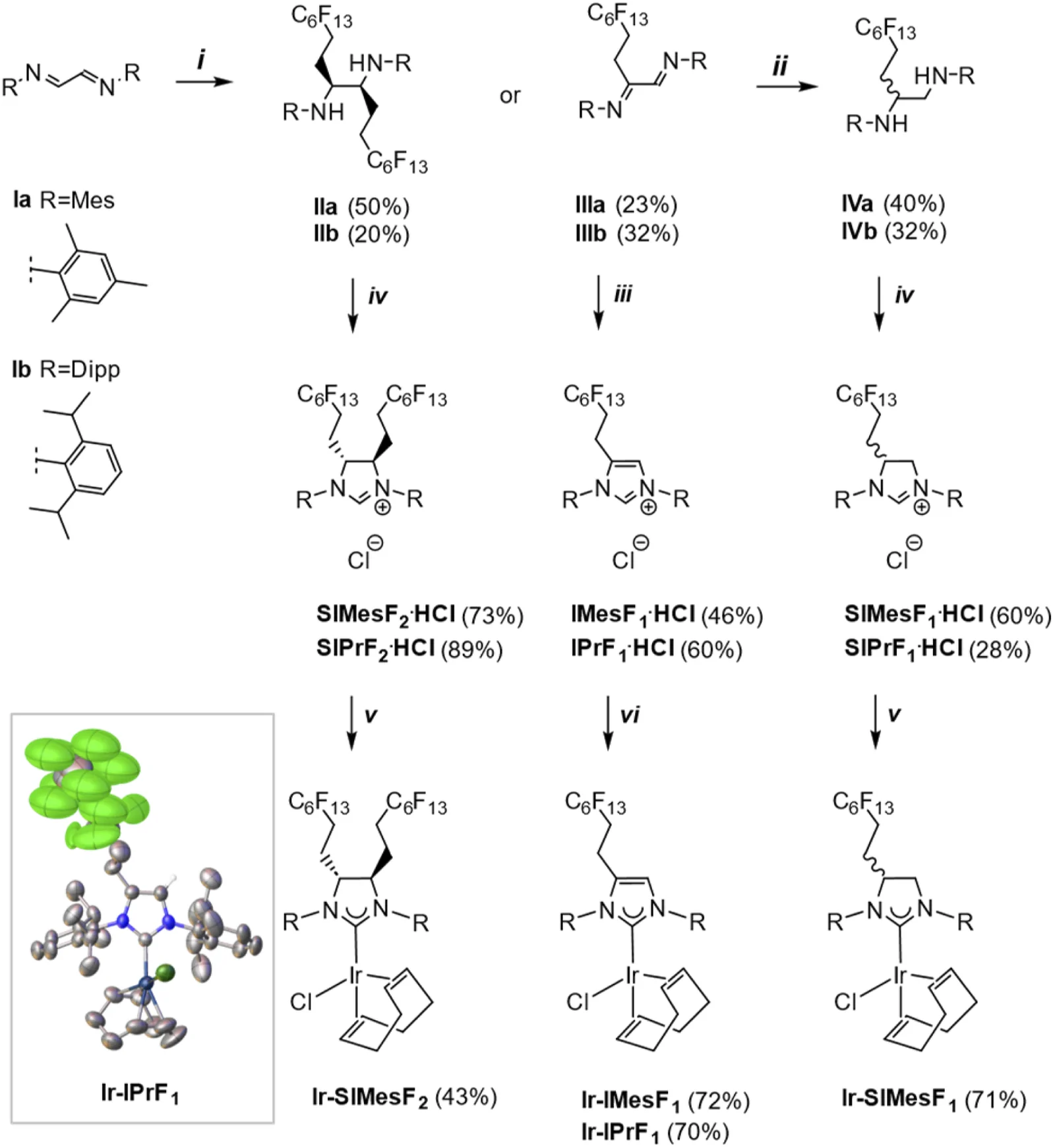
Synthetic route to the perfluorinated catalysts Ir-IMesF_1_, Ir-IPrF_1_, Ir-SIMesF_1_ and Ir-SIMesF_2_. In the inset, the SC-XRD ORTEP structure of Ir-IPrF_1_ is shown, which confirms these precatalysts retain the expected pseudo square planar structure. (i) CH_4_F_13_I, ^*t*^BuLi (different conditions for product II or III, see SI file). (ii) NaBH_4_ or LiAlH_4_. (iii) CH_2_O, HCl. (iv) CH(OEt)_3_, NH_4_BF_4_ and Ambersep. (v) ^*t*^BuOK, [IrCl(COD)]_2_. (vi) K_2_CO_3_, [IrCl(COD)]_2_.

## Results and discussion

### Chemical design

In a recent study, we surveyed a series of SABRE catalysts for [1-^13^C]pyruvate hyperpolarization and rationalized their efficiencies through a detailed mechanistic model accounting for *para*hydrogen pressure, flow and concentration, substrate concentration, intermediate formation, hydrogen exchange, and pyruvate binding kinetics.^26^ In our experiments, Ir-IMes provided approximately threefold higher ^13^C polarization than its saturated counterpart Ir-SIMes (∼3% *vs.* ∼1% at 50% *p*H_2_, 6 bar, in methanol-d_4_). Consistent with these observations, retention of unsaturation in the carbene backbone emerges as a key design element for tuning the substrate–catalyst exchange rate, a critical determinant of SABRE efficiency.^18,19,26,43^ Although NHC-based iridium catalysts are inherently only sparingly soluble in water, an important complementary design strategy was introduced by Ettedgui, Swenson and co-workers, who employed fluorination to increase catalyst hydrophobicity and thereby facilitate catalyst removal during aqueous extraction.^42^ Building upon these complementary design principles, we devised the synthetic route shown in Scheme 1 to retain both carbene backbone unsaturation and hydrophobicity through perfluoroalkyl chains, aiming to maximize polarization efficiency while minimizing residual Ir concentration in aqueous solution.

### Synthesis

Six saturated and unsaturated perfluoroalkylated imidazol(in)ium salts, with one or two perfluoroalkyl chains in the backbone and either mesityl or diisopropylphenyl wingtips, were synthesized (Scheme 1). These synthons act as precursors to the corresponding perfluorinated NHC ligands for SABRE catalysis. This revised alkylation strategy^44^ provides high-yielding access to the desired dialkylated products, as well as to the much rarer *in situ*-reoxidized monoalkylated derivatives. This procedure therefore enables the synthesis of the corresponding, and previously inaccessible, unsaturated perfluoroalkylated imidazolium salt. Coordination of the NHC to [IrCl(COD)]_2_, to form [IrCl(COD)(NHC)], was achieved for all imidazol(in)ium salts, either in the presence of weak or strong bases, with the exception of the two saturated derivatives bearing the 2,6-diisopropylphenyl (Dipp) wingtip substituents (SIPrF_1_·HCl and SIPrF_2_·HCl); this is most likely due to their pronounced steric hindrance. We therefore access three new Ir catalysts, termed Ir-IMesF_1_, Ir-SIMesF_1_, and Ir-IPrF_1_, representing the single-chain fluorinated versions of Ir-IMes, Ir-SIMes and Ir-IPr respectively. Ir-SIMesF_2_ was synthesized for comparison.^42^ Their hyperpolarization performance was evaluated using the SHield Enables Alignment Transfer to Heteronuclei (SABRE-SHEATH) protocol, which enhances the ^13^C NMR signal of sodium [1-^13^C]pyruvate in methanol-d_4_ by several thousand-fold relative to thermal polarization at clinically relevant fields.^18,21,24,27,42,45–52^

### SABRE mechanism

The reaction scheme in Fig. 1a shows how the NHC-based precatalyst 1 is first activated to form intermediate 2 (hydride ligand signals at *δ*(^1^H) = −21.6 ppm and *δ*(^1^H) = −15.6 ppm for Ir-IMesF_1_ in S72), which mediates subsequent pyruvate coordination to the catalyst in either a bidentate 3b (*δ*(^1^H_1_) −29.1 ppm and *δ*(^1^H_2_) −27.1 ppm for Ir-IMesF_1_ in S72) or monodentate 3a (*δ*(^1^H_1_) −24.1 ppm and *δ*(^1^H_2_) −15.0 ppm for Ir-IMesF_1_ in S72) fashion within the equatorial plane. At sub-micro Tesla magnetic fields, the asymmetry in scalar coupling between the hydride protons and the ^13^C nucleus in 3b and 3a creates a level anti-crossing condition^12,53,54^ for spontaneous polarization transfer from the *p*H_2_ derived protons to the carbonyl ^13^C spin of pyruvate, while substrate dissociation releases hyperpolarized free pyruvate in solution. It should be noted that the identity of 3a follows the recent reassignment by Assaf *et al.*, which revises the structure originally proposed by Iali *et al.*^18,55^Fig. 1a shows a single-scan hyperpolarized ^13^C spectrum in methanol-d_4_ (detected at 1.4 T) obtained with Ir-IMes at polarization transfer field (PTF) of 0.35 µT, featuring three pyruvate resonances (free, 3b, 3a) with *ε* > 30 000 relative to thermal signal.

**Fig. 1:**
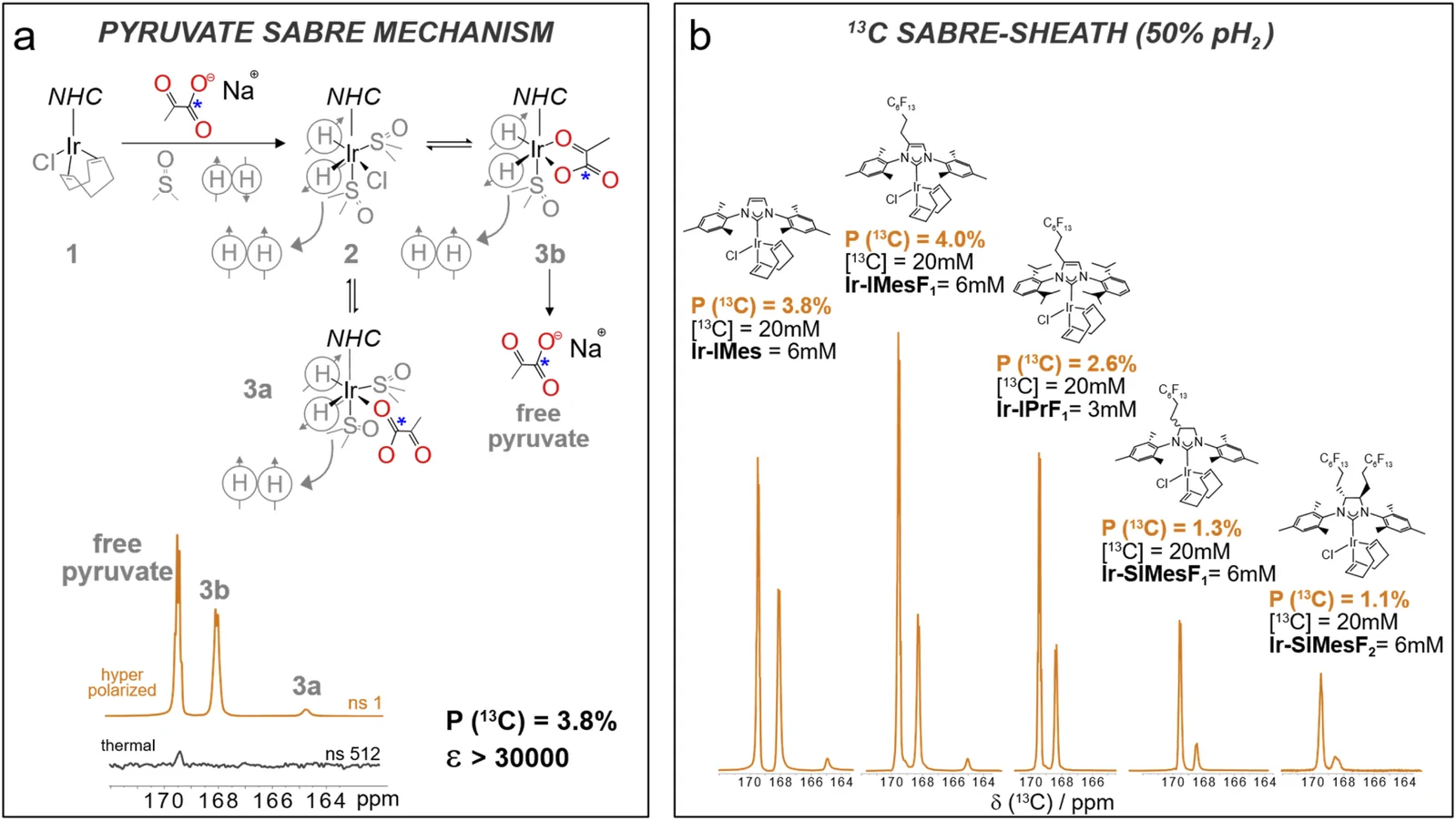
(a) Pyruvate SABRE mechanism and representative hyperpolarized ^13^C spectrum from a 700 µL methanol-d_4_ solution (6 mM Ir-IMes, 30 mM DMSO, 20 mM sodium [1-^13^C]pyruvate), showing free, monodentate bound (3a), and equatorially bidentate bound (3b) species, with enhancement >30 000 (≈3.8% polarization at 1.4 T). (b) Best ^13^C spectra under the same conditions (3 mM for Ir-IPrF_1_ due to solubility). Bubbling: 180 s at 6 bar, 80 sccm, 50% *p*H_2_. *T* = 280 K or 275 K.

### [1-^13^C]pyruvate hyperpolarization

Because SABRE relies on the reversible substrate exchange at the metal centre, catalyst performance is highly sensitive to substrate exchange kinetics, which has been tuned by controlling the bubbling temperature.^26,47^ Consequently, our SABRE-SHEATH experiments were conducted over a wide range of temperatures (240–310 K) with a PTF of 0.35 µT, 50% *para*hydrogen at 6 bar, and a flow of ∼80 sccm in standard 5 mm NMR tubes, using a previously described setup (SI 5.2).^56^ Unless otherwise noted, experiments used 700 µL methanol-d_4_ solutions containing 6 mM precatalyst, 30 mM DMSO, and 20 mM sodium [1-^13^C]pyruvate. Catalysts were activated at 6 bar and room temperature by bubbling H_2_ through the solution for at least 10 min prior to hyperpolarization measurement. This involved 180 s *para*hydrogen bubbling followed by manual sample transfer from the PTF to the observation field, 1.4 T, in 3.0 ± 1.5 s. For all catalysts, the optimal PTF proved to be ∼0.35 µT, with optimal temperatures of around 280 K for the unsaturated catalysts and ∼270–275 K for the saturated versions (see SI 5). The lower optimum temperatures observed for the saturated catalysts are consistent with differences in substrate exchange kinetics, potentially arising from the stronger σ-donor character of the saturated NHC ligands and the resulting changes in electron density at the iridium centre. The ^13^C polarization build-up times (*T*_b_) for catalyst-bound (3b) and free pyruvate in methanol-d_4_ are reported in Section SI 5.3. For the free form, *T*_b_ values for the fluorinated catalysts range from ∼12 s to 26 s at 280 K and 0.35 µT. A markedly longer build-up time (∼42 s) is observed for Ir-IPrF_1_, consistent with hindered substrate–catalyst exchange due to the steric bulk of the Dipp substituents. In addition, the lower optimal polarization temperatures observed for the saturated Ir-SIMesF_1_ and Ir-SIMesF_2_ catalysts suggest that higher temperatures shift these systems away from the optimal exchange regime (Fig. S73).

Fig. 1b summarizes the maximum 1-^13^C pyruvate polarization delivered in methanol-d_4_ for each catalyst with a *p*H_2_ bubbling time of 180 s. The activity of Ir-IMesF_1_ is therefore comparable to that of the widely used Ir-IMes catalyst, delivering around ∼4% total carbon polarization. This demonstrates that fluorination does not compromise polarization efficiency. Ir-IPrF_1_ (tested at 3 mM due to the limited solubility) and Ir-SIMesF_1_ both show a reduction of ∼30% while Ir-SIMesF_2_ shows a 60% decrease in total polarization relative to Ir-IMes and Ir-IMesF_1_. However, since Ir-IPrF_1_ was tested at 3 mM rather than 6 mM because of its limited solubility, a concentration effect on its apparent performance cannot be excluded. Nonetheless, we note that manually performed SABRE experiments inherently display some run-to-run variability, which can be alleviated through automation.^38,57–59^ SABRE-SHEATH generates longitudinal 1-^13^C magnetization that relaxes to its field-dependent thermal equilibrium value. For Ir-IMesF_1_ at 0.35 µT, the pyruvate signal decays with time constants of 20.5 ± 0.7 s and 9.4 ± 0.4 s for the free and 3b forms, respectively (Table SI2); this limits the time available for conversion into an aqueous, biocompatible formulation. In addition, at 0.35 µT Ir-IMes shows relaxation decay constants of 27.4 ± 0.7 s and 9.1 ± 0.6 s for the free and 3b forms, respectively, whereas Ir-SIMesF_2_ exhibits faster decay, with corresponding values of 10.9 ± 0.6 s and 5.7 ± 0.4 s. This faster relaxation may also contribute to the lower overall performance of Ir-SIMesF_2_ relative to Ir-IMesF_1_. Since ^13^C relaxation in pyruvate becomes substantially slower at higher magnetic fields, rapidly transferring the sample after hyperpolarization from the µ-metal chamber to a higher magnetic field provides a practical strategy to extend polarization lifetime during downstream processing. Indeed, placement in a stacked column of 10 neodymium ring magnets (o.d. × i.d. × *h* = 18 × 8 × 9 mm; central field ≳150 mT), extends the ^13^C T_1_ by a factor of 4–10 (Table SI2), providing a practical window for post-processing steps.

Having established that single-chain fluorination of the unsaturated NHC backbone does not compromise SABRE efficiency, we next examined whether a single perfluoroalkyl chain provides sufficient hydrophobicity for efficient catalyst separation from the aqueous hyperpolarized substrate.^31,32^

### Catalyst separation and pyruvate-to-lactate *in vitro* conversion

Over the years, several purification workflows have been proposed to reduce residual catalyst contamination in SABRE-derived formulations.^24,38,39,42^ In the present work, we adopted the ReD SABRE protocol reported by Schmidt and co-workers^24^ for selected catalysts (Ir-IMes, Ir-IMesF_1_ and Ir-SIMesF_2_; Fig. 2b) for a direct comparison with reported results for Ir-SIMesF_2_, and complemented the aqueous extraction step with passage of the aqueous phase through a cartridge packed with fluorinated silica (Fig. 2b). We anticipated that the fluorinated stationary phase would preferentially retain the fluorinated catalysts, thereby providing an additional reduction in residual iridium content beyond that achieved by phase separation alone. The experiments involved hyperpolarizing a 100 µL methanol-d_4_ solution of [1-^13^C]pyruvate for 180 s under the conditions described above (50% *p*H_2_, 6 bar, 0.35 µT, 270–280 K), followed by manual transfer (∼3 s) to a Falcon tube equipped with 10 neodymium ring magnets.^26,56^ Following ReD SABRE, the NMR tube was depressurized and 300 µL of ethyl acetate and 400 µL of D_2_O were added. Brief flushing with H_2_ (∼6 s) induced mixing and rapid phase separation (∼1 s), leaving the aqueous phase at the bottom of the NMR tube (Fig. S75). The aqueous layer was withdrawn with an extraction syringe and injected through a 1.2 µm filter (Whatman glass fibre membrane) into a second NMR tube positioned inside a 1.4 T benchtop NMR for signal acquisition. The additional filtration step, not included in the original ReD protocol, brought the total duration of purification to ∼60 s.

**Fig. 2:**
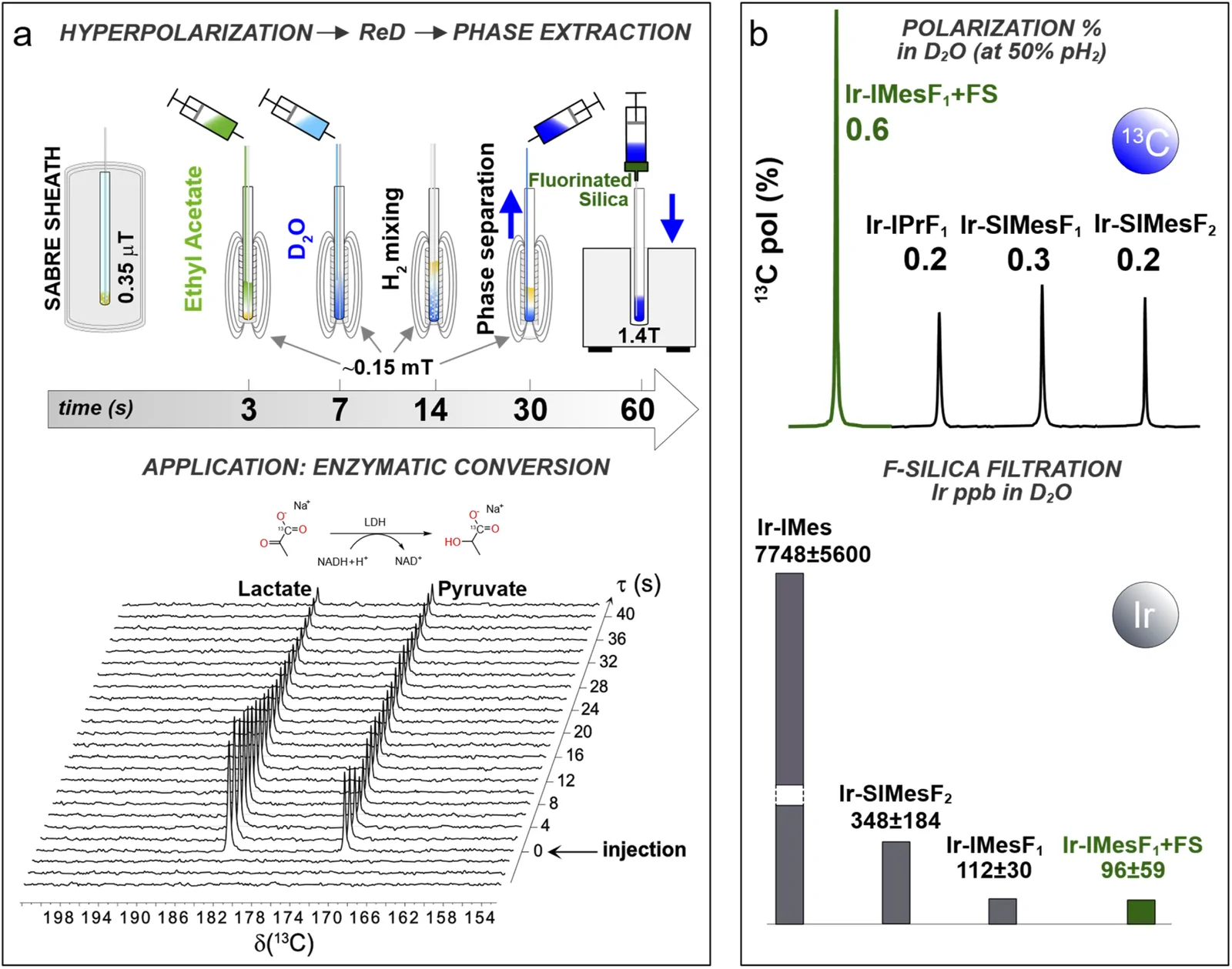
SABRE-SHEATH hyperpolarization of a 100 µL solution containing 6 mM catalyst, 20 mM [1-^13^C]pyruvate and 30 mM DMSO in methanol-d_4_. Conditions: 0.35 µT; 280 K for Ir-IMesF_1_ and Ir-IPrF_1_, 270 K for Ir-SIMesF_1_ and Ir-SIMesF_2_; *p*H_2_ bubbling for 180 s at 6 bar and 80 sccm. (a) Work-up protocol: within ∼3 s the sample is transferred to the ring-magnet region (≳150 mT), the pressure is released, 300 µL ethyl acetate and 400 µL D_2_O are injected and mixed by H_2_ bubbling for ∼6 s. The aqueous phase is then withdrawn with a syringe and transferred to a second NMR tube positioned at 1.4 T through a 1.2 µm filter, with or without fluorinated silica beads. Total protocol duration ∼50 s or ∼60 s with fluorinated silica. At the bottom: *in vitro* enzymatic real-time pyruvate to lactate conversion monitoring of 250 units of bovine heart LDH and 10 mM NADH in phosphate buffer. Total final volume 600 µL. (b) Carbon pyruvate hyperpolarization levels in D_2_O after the ReD, extraction and filtration process for Ir-IMesF_1_ (0.6%), Ir-IPrF_1_ (0.25%), Ir-SIMesF_1_ (0.3%) and Ir-SIMesF_2_ (0.2%). At the bottom: residual Ir content measured in the aqueous phase *via* ICP MS in ppb for Ir-IMes (7748 ± 5600), Ir-SIMesF_2_ (348 ± 184), Ir-IMesF_1_ (112 ± 30) ligands. When adding 50 mg of perfluorinated silica (FS) to the 1.2 µm filter the Ir residual levels for the IMesF_1_ ligand drops to 96 ± 59.

For each catalyst system, three independent replicates were analyzed and residual iridium quantified by ICP-MS. Residual elemental Ir levels were 7748 ± 5600 ppb for Ir-IMes and 348 ± 184 ppb for Ir-SIMesF_2_, achieved after filtration through the non-fluorinated glass fiber membrane and consistent with literature values (*ca.* 8000 ppb for Ir-IMes and an average 542 ± 212 ppb for Ir-SIMesF_2_).^24,51^Ir-IMesF_1_ had the lowest residual Ir concentration at 112 ± 30 ppb. Since Ir-SIMesF_2_ and Ir-IMesF_1_ differ in both backbone saturation and fluorination pattern, the specific contribution of the number of fluorinated chains to catalyst removal cannot be disentangled. When ∼50 mg of fluorinated silica^60^ beads were added above the 1.2 µm filter (Fig. S75), the residual Ir further decreased to an average of 96 ± 59 ppb (*n* = 3), with two out of three measurements below 100 ppb and a best-case value of 56 ppb (see SI 5 and Fig. 2b). The origin of the more efficient removal of Ir-IMesF_1_ remains unclear; differences in conformational mobility and phase partitioning of the fluorinated chains may contribute, although this interpretation remains speculative. Systematic optimization of the fluorinated stationary phase was beyond the scope of the present work. Increasing the amount of fluorinated silica may further decrease residual iridium levels, but this improvement is expected to come at the expense of longer purification times. Because the present workflow is performed manually, the specific polarization loss attributable to the fluorinated-silica filtration step has not been quantified independently from other losses occurring during transfer and sample handling. Given the relatively short ^13^C relaxation time of hyperpolarized pyruvate, any additional processing time is nevertheless expected to reduce the retained polarization. Future optimization of the fluorinated stationary phase will therefore require balancing catalyst removal against polarization preservation. Under identical ReD/extraction/filtration conditions, Ir-IMesF_1_ reproducibly retained detectable aqueous ^13^C polarization, whereas Ir-SIMesF_2_ often yielded weak or undetectable signals. The absolute polarization value (0.6% for Ir-IMesF_1_, Fig. 2b) is, however, lower than those reported in optimized setups,^25,42,51^ reflecting the combined impact of the ∼60 s purification protocol, manual transfer, and use of 50% *p*H_2_ (see SI 5). Based on the measured *T*_1_ values, relaxation is expected to reduce the polarization to ∼40% of its initial value over this timescale, under ideal conditions. Additional signal losses arise from magnetic field inhomogeneities both during transfer and handling inside the stacked column of ring magnets, as well as from removal of the catalyst-bound pyruvate during filtration. We note that the dilution during workup reduces the pyruvate concentration and absolute signal intensity, but not the polarization itself, which was calculated after correction for the final pyruvate concentration.

These results therefore represent a conservative estimate of the achievable performance under non-optimized, yet practical, experimental conditions. Residual ethyl acetate, pyruvate and methanol concentrations reported in SI 5.6 are consistent with previous reports.^42,51^ but are currently not compatible with direct *in vivo* administration and will require further reduction for the biomedical translation of the method.

Despite these reductions, the remaining polarization level is sufficient to monitor the *in vitro* enzymatic pyruvate to lactate conversion with Ir-IMesF_1_. Fig. 2a presents the results of a 100 µL solution of 250 units of bovine heart lactate dehydrogenase (LDH) and 10 mM nicotinamide-adenine dinucleotide (NADH) in phosphate buffer in the benchtop NMR. After hyperpolarization and ReD purification, hyperpolarized [1-^13^C]pyruvate was injected into this solution and the pyruvate (at 170 ppm)-to-lactate (at 182.4 ppm) conversion monitored using consecutive 45° pulses separated by 2 s delays.

Although the present study specifically addresses catalyst-derived metal contamination, clinically translatable aqueous formulations will also require further reduction of residual ethyl acetate and methanol. Additionally, this experiment is intended as a proof-of-concept for benchtop NMR analysis, rather than a detailed study of enzymatic kinetics which remains outside the scope of the paper. Future work will therefore focus on solvent removal and automation of the post-hyperpolarization purification workflow. More broadly, however, the significance of the fluorinated catalysts extends beyond methanol-based SABRE and beyond simply increasing catalyst hydrophobicity. Fluorinated catalysts are also naturally well suited for operation in fluorinated solvents, whose high *para*hydrogen solubility (approximately 3–8 times higher than that of methanol) makes them particularly attractive for homogeneous SABRE hyperpolarization of non-polar substrates. Among these, methylperfluorobutyl ether has been shown to support highly efficient SABRE hyperpolarization at low catalyst loadings, achieving up to 13% ^15^N polarization of pyridine, while also offering a more favorable toxicological profile than conventional SABRE solvents.^61^ These findings suggest that fluorinated catalyst design may be used for expanding both the solvent landscape and substrate scope beyond pyruvate of homogeneous SABRE hyperpolarization.^34^

## Conclusions

In conclusion, we have demonstrated that through a rational NHC ligand design it is possible to achieve high SABRE hyperpolarization efficiency while minimizing metal catalyst contamination of the aqueous pyruvate solution. By engineering single-chain fluorinated NHC architectures, the Ir-IMesF_1_ complex retains essential carbene backbone unsaturation, preserving optimal substrate exchange kinetics, while exploiting enhanced fluorophilicity for rapid liquid–liquid extraction and stationary-phase capture. This synergistic design yields a ^13^C polarization level of ∼4% for [1-^13^C]pyruvate under 50% *p*H_2_ enrichment in methanol-d_4_, matching the benchmark Ir-IMes system and outperforming double-chain saturated analogues by a factor of three. The downstream purification protocol, combining redissolution, phase extraction, and fluorinated silica filtration, reduces residual iridium content to <100 ppb on average (reaching 56 ppb in our best-case experiment), yielding the lowest residual iridium contamination reported to date for SABRE-hyperpolarized pyruvate. The functional viability of the resulting aqueous formulation was validated through real-time *in vitro* monitoring of enzymatic pyruvate-to-lactate conversion. Further translation towards *in vivo* applications will require additional reduction of residual methanol and ethyl acetate. Beyond improving catalyst separation, the fluorinated nature of these complexes may also enable future SABRE implementations in biocompatible fluorous solvents where conventional Ir-IMes displays limited solubility, extending their applicability beyond the aqueous purification strategy demonstrated here. More broadly, the present results identify unsaturated single-chain fluorinated NHC ligands as an effective molecular design principle for balancing hyperpolarization efficiency with catalyst separability in SABRE.

## Author contributions

Conceptualization: G. S. and C. T.; investigation: F. F., A. F., F. D. B., D. B., C. A. G.; formal analysis: F. F., S. M., D. B., C. A. G.; supervision: G. S., C. T, S. B. D.; funding acquisition: G. S., S. B. D.

## Conflicts of interest

There are no conflicts to declare.

## Supplementary Material

RA-OLF-D6RA07354A-s001

## Data Availability

CCDC 2551245 (Ir-IPrF_1_) contains the supplementary crystallographic data for this paper.^62^ All the raw NMR data associated with the manuscript can be accessed at the following link DOI: https://doi.org/10.5281/zenodo.20282998 and is available under the CC-BY-4.0 (Creative Commons Attribution-ShareAlike 4.0 International) license. Additional supplementary information (SI) can be found online in the SI data section. Supplementary information: the synthesis of the catalysts, crystallographic data, characterisation data and hyperpolarisation experiments. See DOI: https://doi.org/10.1039/d6ra07354a.
