# Supplementary material for: Single-chain fluorinated Ir-NHC catalysts for efficient SABRE pyruvate hyperpolarization with average residual iridium below 100 ppb
Source: RSC Adv. 2026 Oct 12;16(47):62474–81. doi: 10.1039/d6ra07354a (PMC13657090; doi:10.1039/d6ra07354a)
Supplement: Supplementary file 1 [file RA-016-D6RA07354A-s001.pdf]

Supplementary Information (SI) for RSC Advances.
This journal is © The Royal Society of Chemistry

SUPPLEMENTARY INFORMATION

Single-Chain Fluorinated Ir-NHC Catalysts For Efficient SABRE Pyruvate Hyperpolarization with Average Residual Iridium Below 100 ppb

Federico Floreani[a], Ahmed Faramawy[a], Federico De Biasi[a], Denis Badocco[a], Salvatore Mamone[b], Callum A. Gater[c], Simon B. Duckett[c], Cristina Tubaro[a*] and Gabriele Stevanato[a*]

[a] Dipartimento di Scienze Chimiche, Università di Padova, Via Marzolo 1, 35131, Padova, Italy

[b] Dept. MESVA (Life, Health & Environmental Sciences), Università dell'Aquila, Via Vetoio SNC, Localita' Coppito, 67100, L'Aquila, Italy

[c] Centre for Hyperpolarisation in Magnetic Resonance University of York (UK), Heslington, York YO10 5NY

Index

1. General 3
2. Synthesis of [IrCl(COD)(NHC)] complexes 4
2.1. General procedure for synthesis of Ia, Ib. 4
2.2. General procedure for synthesis of IIa, IIb, IIIa, IIIb 5
2.3. Synthesis of IVa 7
2.4. Synthesis of IVb 8
2.5. General procedure for synthesis of $SIMesF_2 \cdot HCl$, $SIPrF_2 \cdot HCl$, $SIMesF_1 \cdot HCl$ and $SIPrF_1 \cdot HCl$ 8
2.6. General procedure for synthesis of $IMesF_1 \cdot HCl$, $IPrF_1 \cdot HCl$ 10
2.7. General procedure for synthesis of Ir-$SIMesF_2$, Ir-$SIMesF_1$ 11
2.8. General procedure for synthesis of Ir-$IMesF_1$, Ir-$IPrF_1$ 12
3. Crystallographic data 14
4. Characterization data for the synthesized compounds 17
5. Hyperpolarization experiments 52
5.1. Catalyst activation 52
5.2. Temperature and Magnetic Field profiles 52
5.3. Build-up times 55
5.4. T_1 measurements 56

5.5. Extraction and Filtration 56

5.6. Residual Iridium, pyruvate and solvents concentration in D_2O 58

5.7. ICP-MS data 58

5.8. Polarization calculation 60

5.9. LDH Solution for the In Vitro Conversion of Pyruvate to Lactate 60

6. Characterization of SABRE active catalysts 62

1. General

All the syntheses and manipulations were carried out under an argon atmosphere using standard Schlenk techniques and flame-dried glassware, unless otherwise specified. All chemicals and reagents were purchased from Sigma-Aldrich Merck and used as received, unless otherwise stated. Lithium aluminum hydride (LiAlH₄) was used in the form of commercially available pellets dispersed in mineral oil, to facilitate safe handling under ambient conditions. tert-Butyllithium (^{t}BuLi) was used as a 1.7 M commercial solution in pentane, supplied in 25 mL bottles. The reagent was handled exclusively under inert atmosphere using standard Schlenk techniques to avoid exposure to air and moisture. The solution was consumed shortly after opening to ensure reliability of the stated concentration, and no additional titration was deemed necessary. The fluorous silica used in the hyperpolarization experiments was prepared starting from high-purity silica gel 60 and 1H,1H,2H,2H-perfluorodecyldimethylchlorosilane, following a procedure fully analogous to that reported by Curran et al.[1] ^{1}H and ^{13}C{^{1}H} NMR spectra of salts and complexes were recorded on Bruker Avance 300 and 400 MHz spectrometers at 298 K. Chemical shifts (δ) for ^{1}H and ^{13}C nuclei were referenced to the residual proton and the deuterated solvent signals, respectively. The following abbreviations (and combinations thereof) are used in the compressed notation of the characterization data: br, broad; s, singlet; d, doublet; t, triplet; q, quartet; h, heptet; m, multiplet.

Hyperpolarization and enzymatic conversion experiments were run using a benchtop 60 MHz XPulse NMR (1.4 T) using the standard NMR sequences for ^{13}C NMR with and without proton decoupling

2. Synthesis of [IrCl(COD)(NHC)] complexes

2.1. General procedure for synthesis of Ia, Ib.

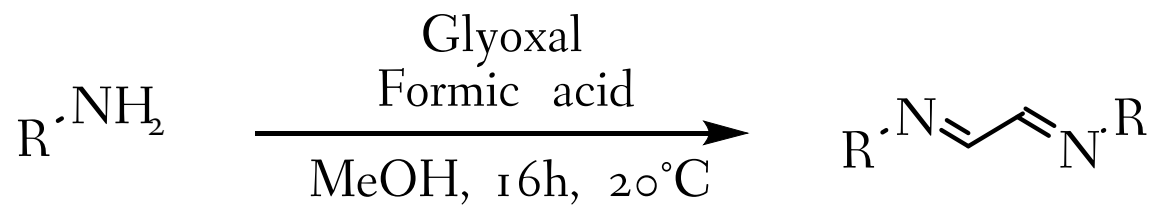

Glyoxal (40 % solution in water, 12.1 mL, 107 mmol) was added to a stirring solution of mesitylaniline (30.0 mL, 214 mmol) or 2,6-diisopropylaniline (40.3 mL, 214 mmol) in methanol (85 mL) at room temperature. After stirring for 5 minutes, a catalytic amount of formic acid (5 drops) was added to the reaction mixture and stirring continued for an additional 16 hours at room temperature. A yellow precipitate formed, which was collected by filtration, washed with cold water (3 × 20 mL), and dried under vacuum to afford the corresponding diimines **Ia** (26.6 g, 85%) and **Ib** (33.0 g, 82%). The ^{1}H NMR spectra of **Ia** and **Ib** agree with those previously reported in the literature.[2] The obtained diimines were used directly in the subsequent step without further purification.

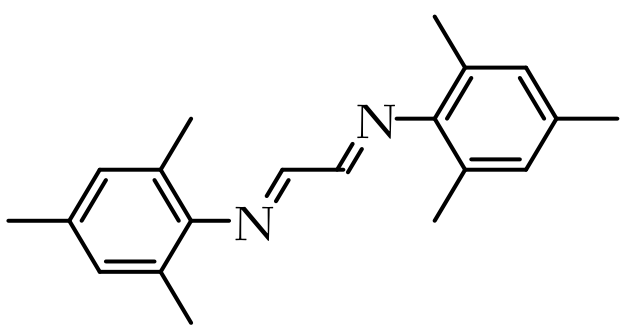

Ia

^{1}H NMR (400 MHz, $CDCl_3$): δ 8.10 (s, 2H, CH), 6.91 (s, 4H, Ar), 2.29 (s, 6H, CH_3), 2.16 (s, 12H, CH_3) ppm.

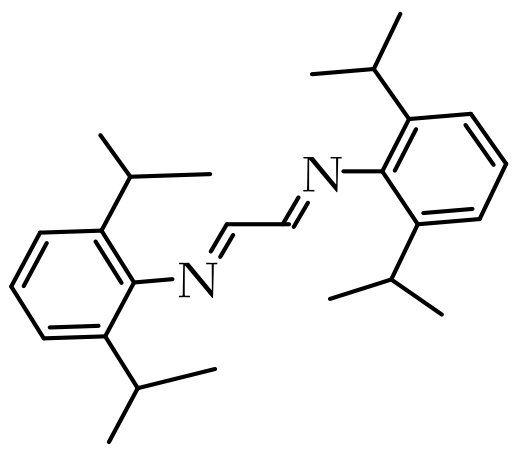

Ib

^{1}H NMR (400 MHz, $CDCl_3$): δ 8.10 (s, 2H, CH), 7.21 – 7.14 (m, 4H, Ar), 2.94 (h, J = 6.8 Hz, 4H, CH), 1.21 (d, J = 6.8 Hz, 24H, CH_3) ppm.

2.2. General procedure for synthesis of IIa, IIb, IIIa, IIIb

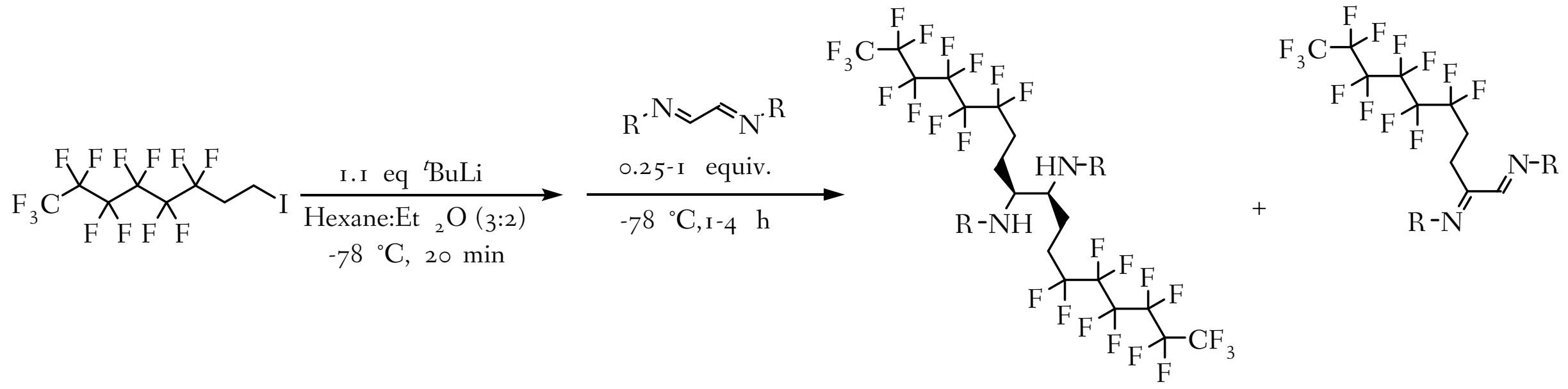

IIa (R=Mes)
IIb (R=Dipp)

IIIa (R=Mes)
IIIb (R=Dipp)

A 1.7 M solution of tert-butyllithium in pentane (1.8 mL, 3.06 mmol) was added to a solution of 1H,1H,2H,2H-perfluorooctyl iodide (0.70 mL, 2.76 mmol) in dry diethyl ether/hexane mixture (2:3) at −78 °C, using 50 mL of solvent for the syntheses of **IIa**, **IIb**, and **IIIb** and 100 mL for the synthesis of **IIIa**. The mixture was stirred for 20 min at −78 °C. A dispersion of the corresponding diimine (amount as reported in Table 1) in the same solvent mixture was then added, employing 25 mL for **IIa**, **IIb**, **IIIb** or 50 mL for **IIIa**, while maintaining the internal temperature at −78 °C.

Product	Diimine Loading
IIa	**Ia** (0.25 equiv, 0.7 mmol, 200 mg)
IIb	**Ib** (0.25 equiv, 0.7 mmol, 260 mg)
IIIa	**Ia** (1 equiv, 2.7 mmol, 800 mg)
IIIb	**Ib** (0.50 equiv, 1.4 mmol, 520 mg)

Table SI1. Diimine loadings used for the synthesis of **IIa,b** and **IIIa,b**

The reaction mixture was stirred for 4 h for the synthesis of **IIa**, **IIb**, and **IIIb**, whereas a reaction time of 1 h was used for the synthesis of **IIIa**. The mixture was then slowly warmed to −30 °C, followed by the addition of saturated NH_4Cl solution (2 mL). Deionized water (30 mL) was then added, the organic phase was separated, and the aqueous phase was extracted with diethyl ether (3 × 20 mL). The combined organic layers were dried over $MgSO_4$ and concentrated. The crude product was then purified by flash column chromatography on silica gel (hexane/dichloromethane 4:1) to afford the corresponding products as a yellow oil **IIa** (Rf=0.68, 340 mg, 50% Yield), **IIIa** (Rf=0.11, 397 mg, 23% Yield), **IIb** (Rf=0.54, 145 mg, 20% Yield), **IIIb** (Rf=0.38, 330 mg, 32% Yield). The NMR spectra of **IIa** agree with those previously reported in the literature.[3]

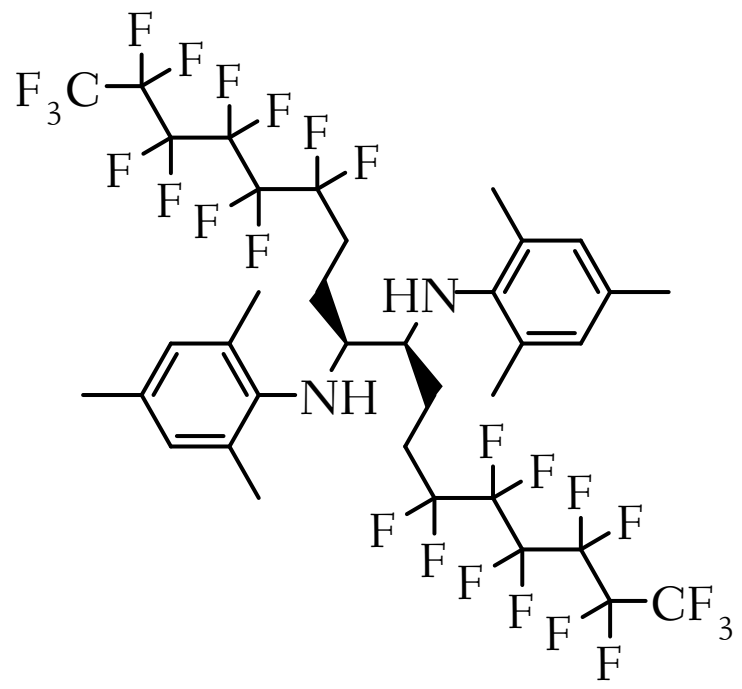

IIa

^{1}H NMR (400 MHz, $CDCl_3$): δ 6.73 (s, 4H, Ar), 3.11 (br, 2H, CH), 2.86 (br s, 2H, NH), 2.75-2.53 (m, 2H, CH_2), 2.23 (s, 6H, CH_3), 2.14-1.98 (m, 4H, CH_2), 1.90 (s, 12H, CH_3), 1.70-1.50 (m, 2H, CH_2) ppm.

^{19}F NMR (376 MHz, $CDCl_3$): δ -83.80 (t, 6F), -114.46 (m, 4F), -121.93 (m, 4F), -122.89 (m, 4F), -123.46 (m, 4F), -126.13 (m, 4F) ppm.

HRMS (ESI+): calcd for $[M+H]^+$ = $C_{36}H_{35}F_{26}N_2^+$: 989.2385 Found 989.2365.

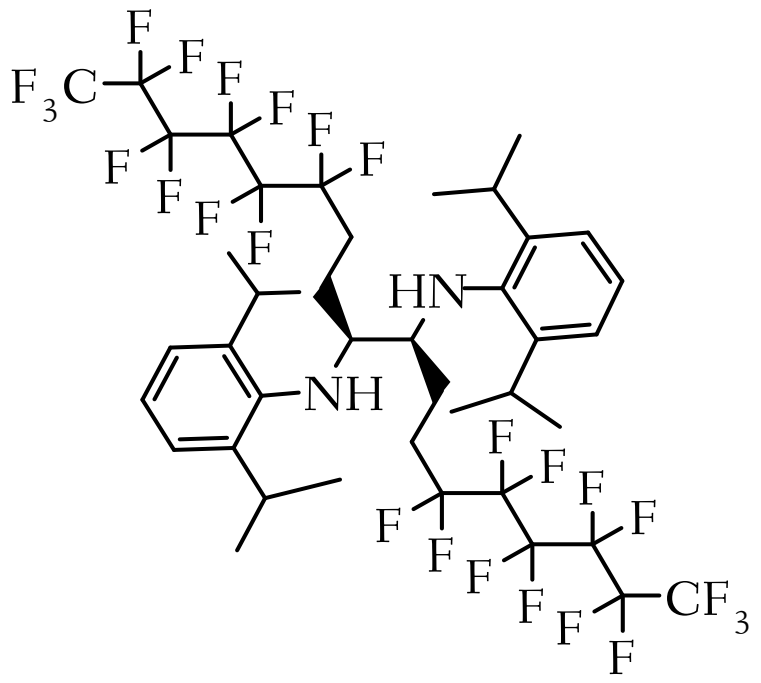

IIb

^{1}H NMR (400 MHz, $CDCl_3$): δ 7.02 (m, 6H, Ar), 3.14 (m, 4H, CH+NH), 3.02 (h, J=6.80 Hz, 4H, CH_{iPr}), 2.34-2.72 (m, 2H, CH_2), 1.94-2.30 (m, 4H, CH_2) 1.65-1.87 (m, 2H, CH_2), 1.18 (d, J=6.80 Hz, 12H, CH_3), 1.00 (d, J=6.80 Hz, 12H, CH_3) ppm.

^{19}F NMR (376 MHz, $CDCl_3$): δ -80.91 (t, 6F), -114.88 (m, 4F), -121.93 (m, 4F), -122.98 (m, 4F), -123.80 (m, 4F), -126.22 (m, 4F) ppm.

^{13}C NMR (100.5 MHz, $CDCl_3$): δ 141.7, 139.8, 123.9, 122.6-103.5, 60.2, 29.1, 28.0, 23.9, 23.6, 21.9 ppm.

HRMS (ESI+): calcd for $[M+H]^+$= $C_{42}H_{47}F_{26}N_2^+$: 1073.3324. Found: 1073.3243.

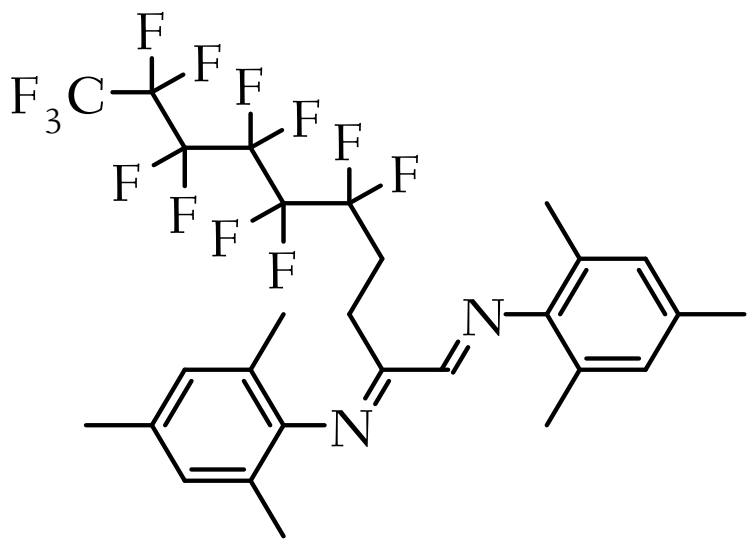

both isomers E/Z

IIIa

The spectra display two distinct sets of signals corresponding to the E and Z isomers. Two species, A and B, are observed in an approximate 1:2 ratio. However, the correlation between A/B and the E/Z configurations has not been established.

^{1}H NMR (400 MHz, $CDCl_3$) isomer A: δ 8.05 (s, 1H, CH), 6.95 (s, 2H, Ar), 6.93 (s, 2H, Ar), 3.63 (m, 2H, CH_2), 2.55-2.36 (m, 2H, CH_2), 2.32 (s, 3H ,CH_3), 2.31 (s, 3H, CH_3), 2.25 (s, 6H, CH_3), 2.24 (s, 6H CH_3). **Isomer B:** δ 7.56 (s, 1H, CH), 6.84 (s, 2H, Ar), 6.82 (s, 2H, Ar), 3.63 (m, 2H, CH_2), 2.90-2.63 (m, 2H, CH_2), 2.18 (s, 3H, CH_3), 2.03 (s, 3H, CH_3), 1.98 (s, 12H, CH_3).

^{19}F NMR (376 MHz, $CDCl_3$): δ -80.86 (m, 6F, CF_3, isomers A and B), -114.16 (m, 2F, isomer A), -115.03 (m, 2F, isomer B), -121.89 (m, 2F, isomer A), -122.89 (m, 2F, isomer A), -123.41 (m, 2F, isomer B), -123.67 (m, 2F, isomers A), -126.17 (m, 4F, isomers A and B) ppm.

^{13}C NMR (100.5 MHz, $CDCl_3$): δ 168.0, 165.8, 163.6, 155.7, 147.2, 144.3, 134.5, 133.4, 129.3, 129.1, 129.0, 128.8, 126.9, 126.6, 125.2, 124.4-108.4, 27.8,

24.7, 20.8, 18.4, 18.2, 17.9 ppm. Signals are listed without assignment to isomer A or B.

HRMS (ESI+): calcd for $[M+H]^+ = C_{28}H_{28}F_{13}N_2^+$: 639.2045. Found: 639.2054.

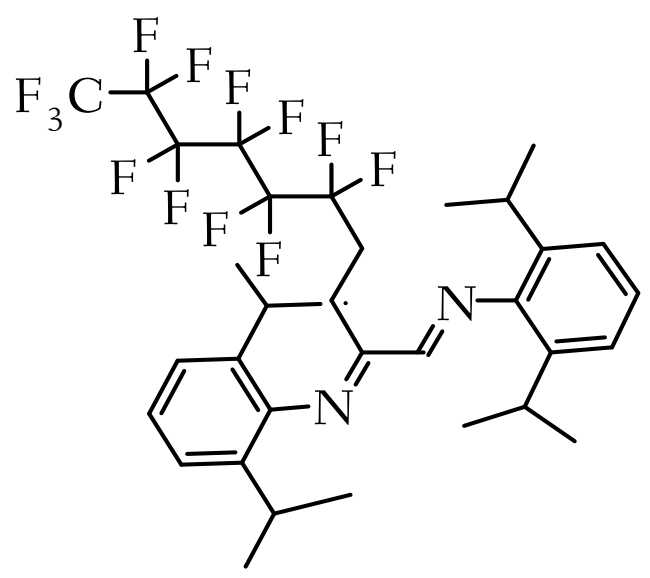

both isomers E/Z

IIIb

The spectra display two distinct sets of signals corresponding to the E and Z isomers. Two species, A and B, are observed in an approximate 1:2 ratio. However, the correlation between A/B and the E/Z configurations has not been established.

^{1}H NMR (400 MHz, $CDCl_3$): isomer A: δ 8.05 (s, 1H, CH), 7.25-7.00 (m, 6H, Ar), 2.95(h, J=6.90Hz ,2H, CH_{iPr}), 2.88-2.70 (m, 2H, CH_2),2.68 (h, J=6.90Hz, 2H, CH_{iPr}), 2.52(m, 2H, CH_2), 1.24 (d, J=6.90Hz ,6H, CH_3), 1.24 (d, J=6.90Hz,12H, CH_3) ,1.17 (d, J=6.90Hz, 6H, CH_3). **Isomer B:** δ 7.62 (s, 1H, CH) 7.25-7.00 (m, 6H, Ar), 3.41 (m, 2H, CH_2), 2.88-2.70 (m, 2H, $2CH_2$) ,2.72 (m, 4H, CH_{iPr}), 1.17 (d, J=6.90Hz, 6H, CH_3) ,1.13 (d, J=6.90Hz, 6H, CH_3), 1.09 (d, J=6.90Hz, 12H, CH_3).

^{19}F NMR (376 MHz, $CDCl_3$): δ -80.75 (m, 3F, CF_3, isomers A and B), -114.20 (m, 2F, isomers B),-115.46 (m, 2F, isomers A),-121.91 (m, 2F, isomers A and B),-122.84 (m, 2F, isomers A and B),-123.40 (m, 2F, isomers B),-123.87(m, 2F, isomers A), -126.10 (m, 2F, isomers A and B) ppm.

2.3. Synthesis of IVa

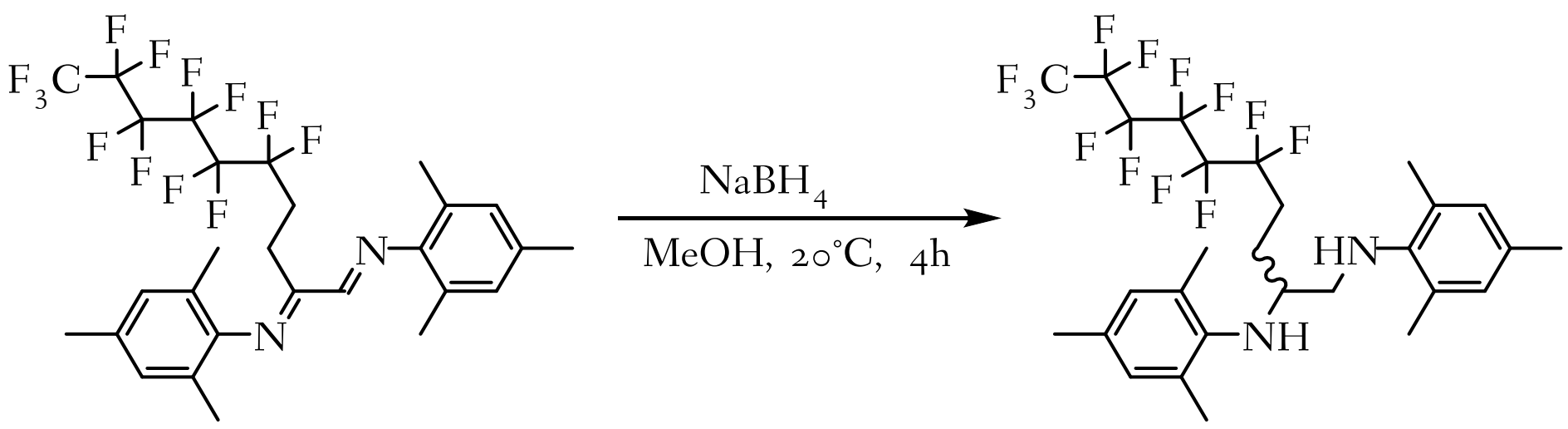

$NaBH_4$ (332 mg, 8.70 mmol) was added portion wise over 30 min to a solution of diimine **IIIa** (348 mg, 0.54 mmol) in anhydrous methanol. The reaction mixture, initially pale yellow, gradually became colorless during the addition. The mixture was stirred for 4 h, after which it was diluted with distilled water and extracted with diethyl ether (3 × 8 mL). The organic phase was dried over magnesium sulfate and evaporated to dryness to obtain the diamine **IVa** as a yellow oil (160 mg, 46% yield), which was used in subsequent reactions without further purification.

^{1}H NMR (400 MHz, $CDCl_3$): δ 6.83 (s, 2H, Ar), 6.81 (s, 2H, Ar), 3.47 (q, 1H, CH), 3.06 (dd, J=5.80 Hz, 1H, CH_2), 2.85 (dd, J=5.80 Hz, 1H, CH_2), 2.70 (br, 2H, NH), 2.47-2.28 (m, 2H, CH_2), 2.27 (s, 6H, CH_3), 2.24 (s, 3H, CH_3), 2.23 (s, 3H, CH_3), 2.18 (s, 6H, CH_3), 2.06-1.93 (m, 1H, CH_2), 1.84-1.63 (m, 1H, CH_2) ppm.

^{19}F NMR (376 MHz, $CDCl_3$): δ -80.80 (t, 3F), -114.50 (m, 2F), -121.93 (m, 2F), -122.88 (m, 2F), -123.56 (m, 2F), -126.16 (m, 2F) ppm.

13C NMR (100.5 MHz, CDCl3): δ 143.1, 143.2, 131.9, 131.5, 130.1, 129.9, 129.8, 129.7, 128.9, 118.8, 116.0, 113.3, 111.1, 108.7, 56.8, 53.2, 28.5, 28.1, 25.2, 20.6, 18.3, 18.2, 1.2ppm.

HRMS (ESI+): calcd for $[M+H]^+ = C_{28}H_{32}F_{13}N_2^+$: 643.2358. Found: 643.2365.

2.4. Synthesis of IVb

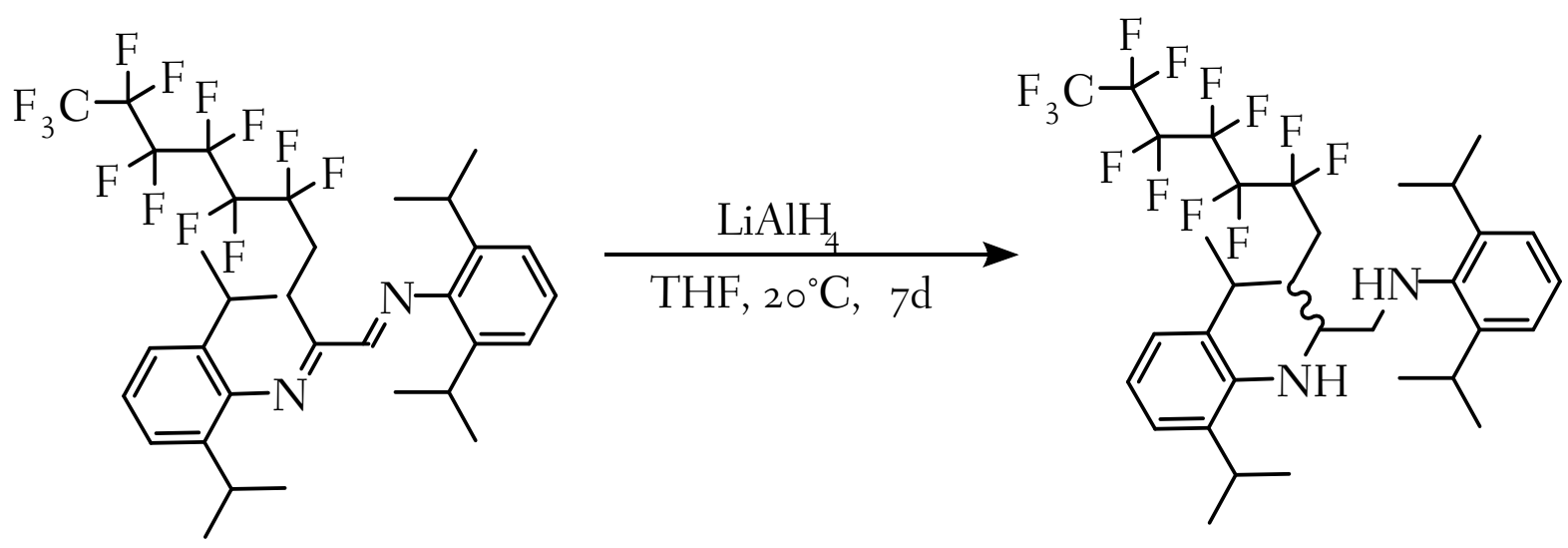

To a solution of diimine **IIIb** (600 mg, 0.84 mmol) in anhydrous THF (100 mL) LiAlH₄ (two pellets, dispersed in mineral oil) was added. The reaction mixture was stirred for one week, after which it was carefully quenched with 1 M aqueous NaOH. The resulting mixture was extracted with diethyl ether. The organic layer was dried over anhydrous magnesium sulfate and concentrated under reduced pressure to afford the corresponding diamine **IVb** as an orange oil (196 mg, 32% yield), which was used in the subsequent reaction without further purification.

^{1}H NMR (400 MHz, $CDCl_3$) 7.25-7.00 (m, 6H, Ar), 3.45-3.14 (m, 5H, 4CH+CH), 3.10-2.90 (m, 2H, CH_2), 2.88-2.39 (m, 2H CH_2), 2.37-2.12 (br, 2H, NH), 2.00-1.90 (m, 2H, CH_2) ,1.45-1.06 (m, 24H, CH_3) ppm.

^{19}F NMR (376 MHz, $CDCl_3$) δ -80.83 (t, 3F) -114.8-113.3 (m, 2F), -121.90 (m, 2F), -122.87 (m, 2F), -123.54 (m, 2F), -126.15 (m, 2F) ppm.

HRMS (ESI+): calcd for $[M+H]^+ = C_{34}H_{43}F_{13}N_2^+$: 727.3297. Found 727.3355.

2.5. General procedure for synthesis of SIMesF$_2$·HCl, SIPrF$_2$·HCl, SIMesF$_1$·HCl and SIPrF$_1$·HCl

A mixture of the proper diamine [**IIa** (247 mg, 0.25 mmol), **IIb** (268 mg, 0.25 mmol), **IVa** (160 mg, 0.25 mmol), or **IVb** (182 mg, 0.25 mmol)], ammonium tetrafluoroborate (30 mg, 0.28 mmol), and triethyl orthoformate (0.5 mL) was heated at 125 °C and stirred for 15 h. The excess triethyl orthoformate was removed under reduced pressure, and the product was isolated by precipitation with diethyl ether (**SIMesF$_2$·HCl**, **SIMesF$_1$·HCl**) or hexane (**SIPrF$_2$·HCl**, **SIPrF$_1$·HCl**). The product was dissolved in 5 mL of methanol in a flask, and 1 g of AMBERSEP® 21K XLT mesh anion-exchange resin was added to exchange the tetrafluoroborate ions with chloride ions. The flask was stirred for

two days, after which the mixture was filtered and the solution evaporated to afford the corresponding imidazolinium chloride salts. The imidazolinium salts **SIMesF$_2$·HCl** (189 mg, 73% yield), **SIPrF$_2$·HCl** (249 mg, 89% yield), **SIMesF$_1$·HCl** (103 mg, 60% yield) or **SIPrF$_1$·HCl** (54 mg, 28% yield) were obtained as white solids.

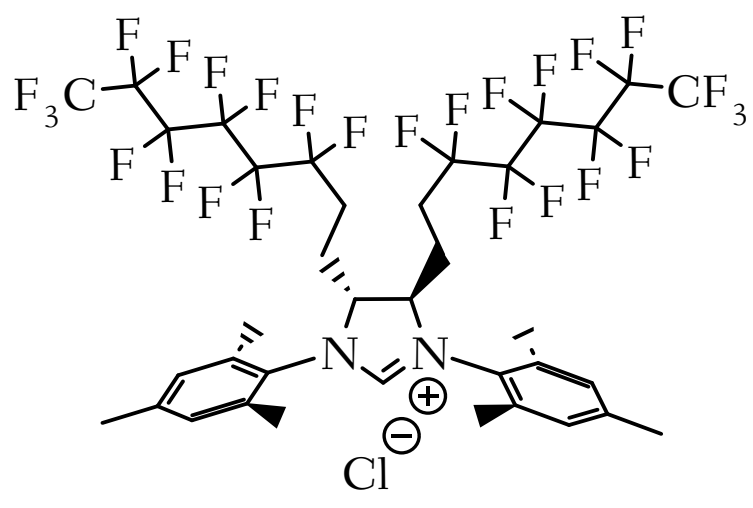

SIMesF $_2$·HCl

^{1}H NMR (400 MHz, CDCl$_3$): δ 10.56 (s, 1H, CH_{Im}), 7.01 (m, 4H, CH_{Ar}), 4.60 (br, 2H, CH_{Im}), 2.44 (s, 6H, CH_3), 2.41 (s, 6H, CH_3), 2.30 (s, 6H, CH_3), 2.30-1.95 (m, 8H, CH_2) ppm.

^{19}F NMR (376 MHz, CDCl$_3$): δ -80.80 (m, 6F) -113.98 (q, 4F), -121.90 (m, 4F), -122.91 (m, 2F), -123.78 (m, 2F), -126.21 (m, 4F) ppm.

^{13}C NMR (100.5 MHz, CDCl$_3$): δ 161.0, 141.3, 135.7, 134.5, 130.9, 128.9, 120.2-108.24, 68.2, 28.3, 25.3, 21.1, 19.1, 18.6 ppm.

HRMS (ESI+): calcd for $[M]^+ = C_{37}H_{33}F_{26}N_2^+$: 999.2223. Found: 999.2142.

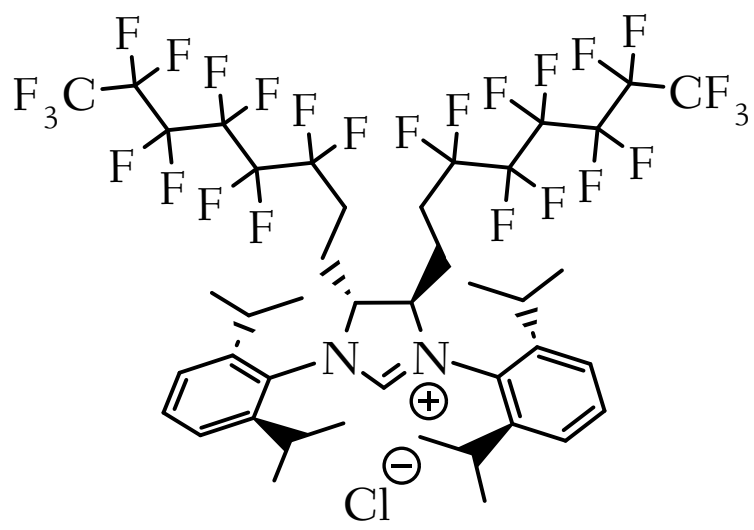

SIPrF $_2$·HCl

^{1}H NMR (400 MHz, CDCl$_3$): δ 10.10 (s, 1H, CH_{Im}), 7.53 (t, J=7.80 Hz, 2H, Ar), 7.38-7.30 (m, 4H, Ar), 4.97-4.78 (m, 2H, CH_{Im}), 3.17-2.93 (m, 4H, CH_{iPr}), 2.45-2.21 (m, 4H, CH_2), 2.20-1.89 (m, 4H, CH_2), 1.48-1.29 (m, 24H, CH_3) ppm.

^{19}F NMR (376 MHz, CDCl$_3$): δ -80.80 (m, 6F) -113.98 (m, 4F), -121.90 (m, 4F), -122.91 (m, 2F), -123.76 (m, 2F), -126.21 (m, 4F) ppm.

^{13}C NMR (100.5 MHz, CDCl$_3$): δ 160.4, 147.2, 146.4, 132.2, 127.8, 125.7, 125.6, 70.4, 29.9, 26.6, 25.9, 24.3, 23.1 ppm.

HRMS (ESI+): calcd for $[M]^+ = C_{43}H_{45}F_{26}N_2^+$: 1083.3162. Found: 1083.3259.

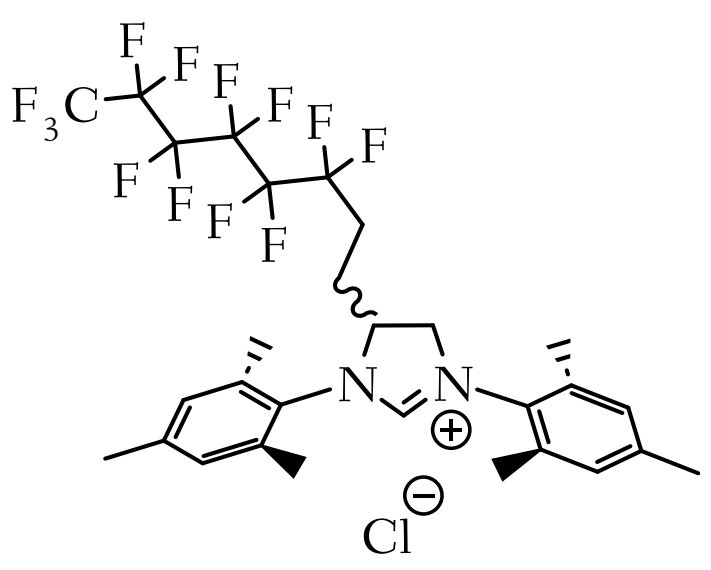

SIMesF $_1$·HCl

^{1}H NMR (400 MHz, CDCl$_3$): δ 9.58 (s, 1H, CH_{Im}), 6.99 (m, 4H, Ar), 5.30-5.28 (m, 1H, CH_{Im}), 4.99 (m, 1H, CH_{2Im}), 3.98 (m, 1H, CH_{2Im}), 2.43-2.41 (m, 12H, CH_3), 2.31-2.28 (m, 6H, CH_3), 2.19-2.14 (m, 4H, CH_2) ppm.

^{19}F NMR (376 MHz, CDCl$_3$): δ -80.76 (m, 3F) -114.08 (m, 2F), -121.88 (m, 2F), -122.87 (m, 2F), -123.36 (m, 2F), -126.14 (m, 2F) ppm.

^{13}C NMR (100.5 MHz, CDCl$_3$): δ 159.9, 141.1, 141.0, 135.4, 130.9, 130.5, 130.0, 128.8, 63.3, 57.2, 31.1, 21.2, 21.1, 19.0, 18.8, 18.3, 18.1 ppm.

HRMS (ESI+): calcd for $[M]^+ = C_{29}H_{30}F_{13}N_2^+$: 653.2196. Found: 653.2242.

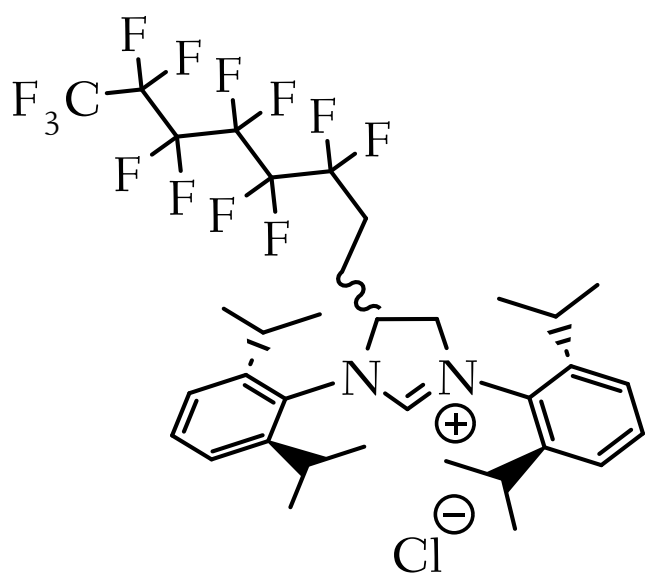

SIPrF$_1$·HCl

^{1}H NMR (400 MHz, $CDCl_3$): δ 8.84 (s, 1H, CH_{Im}), 7.56-7.43 (m, 2H, Ar), 7.38-7.26 (m, 4H, Ar), 5.66-5.32 (m, 2H, CH_{2Im}), 4.12 (m, 1H, CH_{Im}), 3.33-3.15 (m, 2H, CH_{iPr}), 3.11-3.00 (m, 1H, CH_{iPr}), 2.99-2.87 (m, 1H, CH_{iPr}), 2.45-1.84 (m, 4H, CH_2), 1.51-1.18 (m, 24H, CH_3) ppm.

^{19}F NMR (376 MHz, $CDCl_3$): δ -80.79 (m, 3F), -114.11(m, 2F), -121.92 (m, 2F), -122.5 (m, 2F), -123.0 (m, 2F), -126.1 (m, 2F) ppm.

^{13}C NMR (100.5 MHz, $CDCl_3$): δ 158.7, 147.4, 147.1, 146.8, 145.7, 132.0, 131.9, 129.3, 127.6, 125.9, ,125.5, 125.1, 124.9,120.4-108.3, 65.8, 60.1, 29.9, 29.6, 29.5, 29.4, 26.4, 24.6, 24.1, 23.9, 22.0 ppm.

HRMS (ESI+): calcd for $[M]^+ = C_{35}H_{42}F_{13}N_2^+$: 737.3135. Found: 737.3152.

2.6. General procedure for synthesis of IMesF$_1$·HCl, IPrF$_1$·HCl

Paraformaldehyde (10 mg, 0.33 mmol) was treated with a 2M solution of HCl in diethyl ether (1.0 mL, 2.00 mmol) and the mixture was allowed to stand for 15 min until complete dissolution. This solution was then added to a stirred solution of **IIIa** (192 mg, 0.30 mmol) or **IIIb** (216 mg, 0.30 mmol) in 3 mL of EtOAc and the reaction mixture was stirred at room temperature for 20 h. At the end of the reaction time a white precipitate was observed. After completion of the reaction, the solvent was removed under reduced pressure. The crude solid obtained from **IIIa** was washed with diethyl ether, whereas the crude residue obtained from **IIIb** was washed with hexane. The resulting crude white solids were purified by flash column chromatography on silica gel, eluting sequentially with neat dichloromethane and ethyl acetate to remove impurities, followed by methanol to afford **IMesF$_1$·HCl** (142 mg, 69% yield) and **IPrF$_1$·HCl** (139 mg, 60% yield) as white powders.

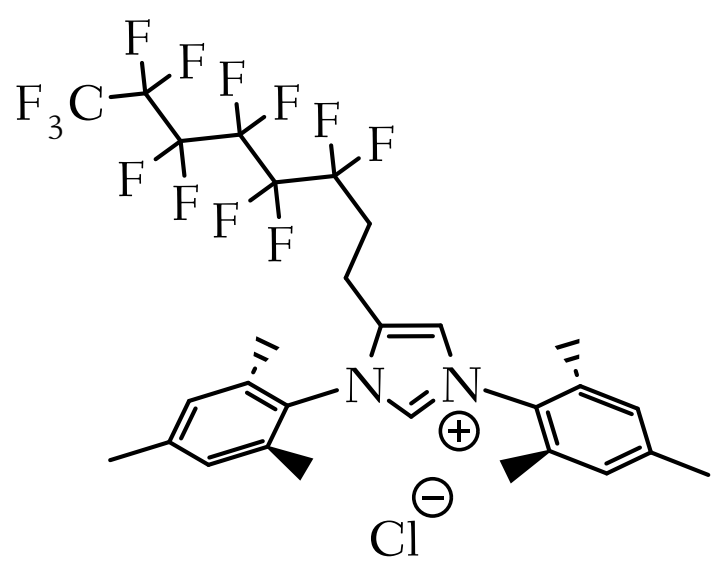

IMesF$_1$·HCl

^{1}H NMR (400 MHz, $CDCl_3$): δ 10.94 (s,1H, CH_{Im}), 7.51 (s, 1H, CH_{Im}), 7.07 (s, 2H, Ar), 7.00 (s, 2H, Ar), 2.77 (m, 2H, CH_2), 2.44 (m, 2H, CH_2), 2.35 (s, 3H, CH_3), 2.32 (s, 3H, CH_3), 2.18 (s, 6H, CH_3), 2.13 (s, 6H, CH_3) ppm.

^{19}F NMR (376 MHz, $CDCl_3$): δ -80.78 (t, 3F) -114.08 (m, 2F), -121.88 (m, 2F),-122.87 (m, 2F), -123.38 (m, 2F), -126.14 (m, 2F) ppm.

^{13}C NMR (100.5 MHz, $CDCl_3$): δ 142.0, 141.4, 140.1, 134.6, 133.7, 130.6, 130.4, 130.0, 128.0, 121.5, 118.7-108.3, 29.8, 29.0, 28.8, 28.6, 21.3, 17.7 ppm.

HRMS (ESI+): calcd for $[M]^+ = C_{29}H_{28}F_{13}N_2^+$: 651.2039. Found 651.2047.

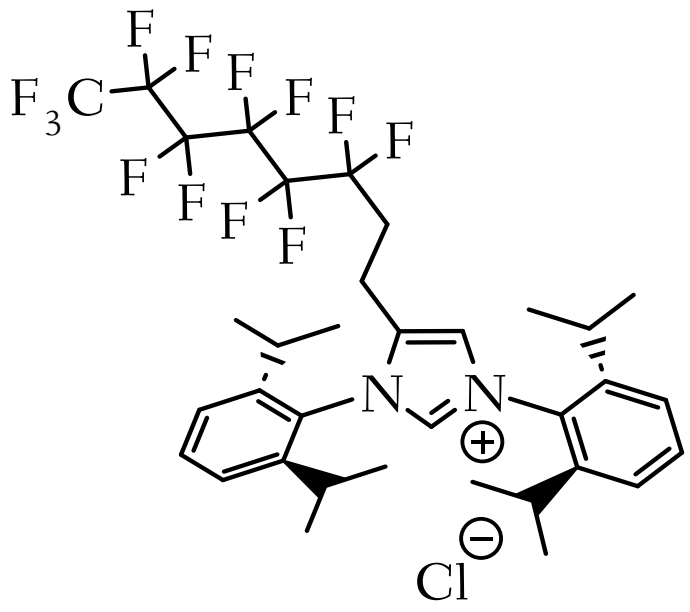

IPrF$_1$·HCl

^{1}H NMR (400 MHz, $CDCl_3$): δ 10.77 (s, 1H, CH_{Im}), 8.02 (s, 1H, CH_{Im}), 7.62 (t, J=7.8 Hz, 1H, Ar), 7.54 (t, J=7.8 Hz, 1H, Ar), 7.40 (d, J=7.8 Hz, 2H, Ar), 7.32 (d, J=7.8 Hz, 2H, Ar), 2.88-2.75 (m, 2H, CH_2), 2.75-2.54 (m, 2H, CH_2), 2.44 (h, J=6.8 Hz, 2H, CH_{iPr}), 2.31 (h, J=6.8 Hz, 2H, CH_{iPr}), 1.35-1.26 (m, 24H, CH_3) ppm.

^{19}F NMR (376 MHz, $CDCl_3$): δ -80.76 (t, 3F), -114.11 (m, 2F), -122.92 (m, 2F), -121.92 (m, 2F), -123.01 (m, 2F), -126.12 (m, 2F) ppm.

^{13}C NMR (100.5 MHz, $CDCl_3$): δ 145.5, 145.1, 139.6, 134.9, 132.8, 132.1, 130.2, 127.3, 125.3, 124.8, 29.4, 29.3, 25.7, 24.83, 23.87, 22.72 ppm.

HRMS (ESI+): calcd for $[M]^+ = C_{35}H_{40}F_{13}N_2^+$: 735.2978. Found 735.3033.

2.7. General procedure for synthesis of Ir-SIMesF$_2$, Ir-SIMesF$_1$

Potassium tert-butoxide (21 mg, 0.19 mmol) was added to a stirred solution of **SIMesF$_1$·HCl** (102 mg, 0.15 mmol) or **SIMesF$_2$·HCl** (156 mg, 0.15 mmol) in THF (5 mL) at room temperature in a glove box. The resulting suspension was stirred for 15 minutes. A solution of [IrCl(COD)]₂ (50 mg, 0.075 mmol) in THF (1 mL) was added and the resulting solution was stirred at room temperature overnight. The solvent was removed under reduced pressure to give the crude product, which was purified by flash chromatography on silica gel to afford **Ir-SIMesF$_1$** (DCM, Rf= 0.77, 105 mg, 71% yield) and **Ir-SIMesF$_2$** (DCM, 86 mg, 43% yield) as orange powders.

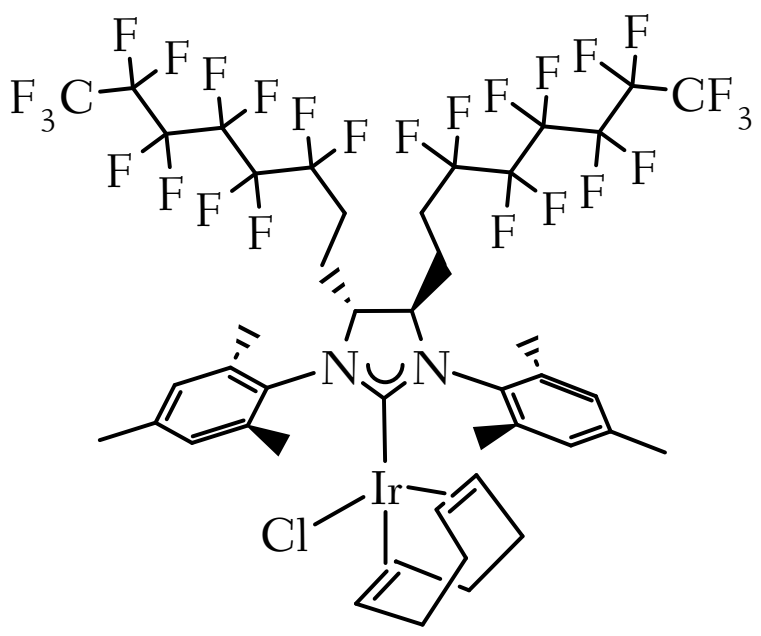

Ir-SIMesF$_2$

^{1}H NMR (400 MHz, $CDCl_3$): δ 7.05 (s, 1H, Ar), 6.99 (s, 1H, Ar), 6.95-6.90 (2s, 2H, Ar), 4.28 (m, 1H, CH), 4.08 (m, 1H, CH), 3.78 (m, 2H, CH_{Im}), 3.00 (m, 1H, CH), 2.72 (m, 1H, CH), 2.67 (s, 3H, CH_3), 2.55 (s, 3H, CH_3), 2.48 (s, 3H, CH_3), 2.33 (s, 3H, CH_3), 2.30 (s, 3H, CH_3), 2.20 (s, 3H, CH_3), 2.15-1.50 (m, 16H, CH_2) ppm.

^{19}F NMR (376 MHz, $CDCl_3$): δ -80.83 (m, 6F), -114.28 (q, 4F), -121.94 (m, 4F), -122.93 (m, 2F), -123.91 (m, 2F), -126.24 (m, 4F) ppm.

^{13}C NMR (100.5 MHz, $CDCl_3$): δ 206.7, 138.5, 138.3, 137.9, 136.3, 135.3, 134.8, 134.7, 130.6, 130.4, 129.3, 118.7-108.3, 86.7, 83.3, 68.4, 68.1, 55.1, 49.9, 31.4, 30.7, 29.8, 27.2, 26.5, 21.3, 21.1, 20.9, 18.7ppm.

HRMS (ESI+): calcd for $[M\text{-}Cl]^+ = C_{45}H_{44}F_{26}N_2Ir^+$: 1299.2719. Found: 1299.2670. calcd for $[M\text{- } C_8H_{12}ClIr]^+ = C_{37}H_{33}F_{26}N_2^+$: 999.2223. Found: 999.2142.

The ^{1}H NMR, ^{13}C NMR and ^{19}F NMR spectra agree with those previously reported in the literature.[4]

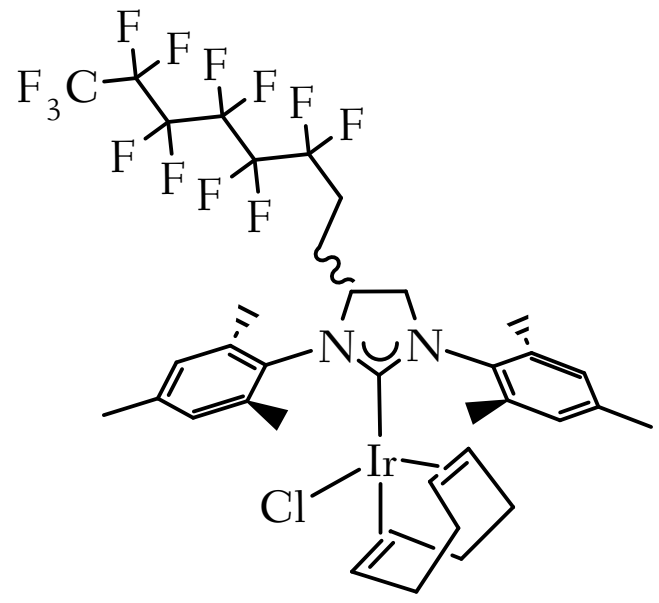

Ir-SIMesF 1

The spectra display two distinct sets of signals corresponding to two isomers.

^{1}H NMR (400 MHz, CDCl$_3$): δ 7.06-6.89 (m, 4H, Ar), 4.31-3.94 (m, 4H, CH_{2Im}+2CH_{COD}), 3.67-3.52 (m, 1H, CH_{Im}), 3.16-2.98 (m, 1H, CH_{COD}), 2.87-2.76 (m, 1H, CH_{COD}), 2.68-2.44 (m, 6H, CH_3), 2.42-2.21 (m, 12H, CH_3), 2.10-1.77 (m, 4H, CH_2), 1.75-1.06 (m, 8H, CH_2) ppm.

^{19}F NMR (376 MHz, CDCl$_3$): δ -80.80 (m, 6F), -114.04 (m, 4F), -121.86 (m, 4F), -122.86 (m, 2F), -123.48 (m, 2F), -126.12 (m, 4F) ppm.

^{13}C NMR (100.5 MHz, CDCl$_3$): δ 209.0, 207.3, 138.8, 138.3, 138.2, 138.1, 138.0, 137.7, 136.4, 136.3, 135.6, 135.3, 135.2, 135.05, 134.9, 130.4, 130.2, 130.2, 130.1, 129.1, 128.9, 128.7, 128.6, 86.2, 84.7, 83.6, 83.3, 63.5, 63.2, 57.1, 56.8, 54.2, 52.4, 50.6, 50.1, 35.4, 34.1, 32.8, 31.8, 30.5, 29.3, 28.0, 26.9, 25.2, 21.4, 21.2, 21.1, 21.1, 20.5, 20.16, 20.1, 19.9, 19.0, 18.8, 18.4 ppm.

HRMS (ESI+): calcd for [M-Cl]$^+$ = $C_{37}H_{41}F_{13}N_2Ir^+$: 953.2691. Found: 953.2680.

2.8. General procedure for synthesis of Ir-IMesF$_1$, Ir-IPrF$_1$

A vial was charged with **IMesF$_1$·HCl** (122 mg, 0.178 mmol) or **IPrF$_1$·HCl** (137 mg ,0.178 mmol), [IrCl(COD)]$_2$ (60 mg, 0.089 mmol) and K_2CO_3 (74 mg, 0.534 mmol). The resulting mixture was suspended in acetone (3.0 mL) and stirred for 20 h at 60 ºC. After cooling to room temperature, the crude reaction mixture was purified by column chromatography on silica gel to obtain **Ir-IMesF$_1$** (DCM, Rf=0.56, 126 mg, 72%) and **Ir-IPrF$_1$** (DCM, Rf=0.63, 133 mg, 70%) as orange powders.

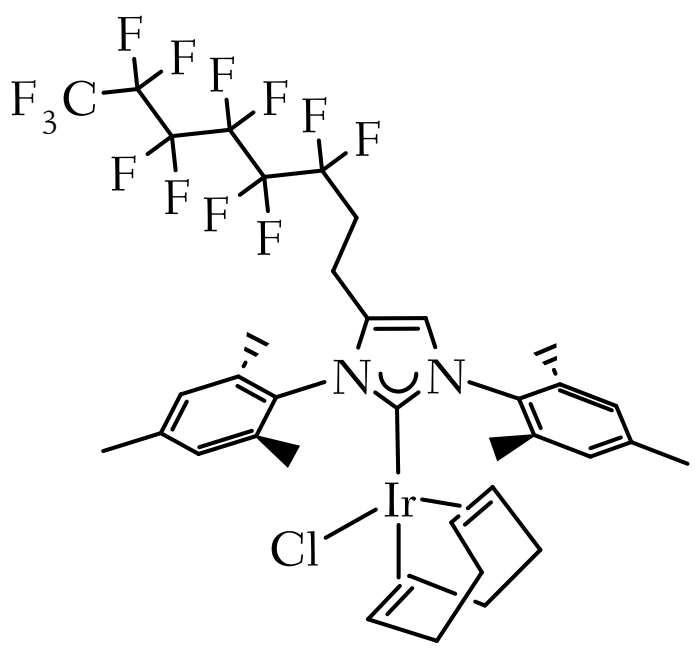

Ir-IMesF 1

^{1}H NMR (400 MHz, CDCl$_3$): δ 7.06-6.92 (m, 4H, Ar), 6.74 (m, 1H, CH_{Im}), 4.20-4.06 (m, 2H, CH), 3.06-2.97 (m, 1H, CH), 2.97-2.88 (m, 1H, CH), 2.53-2.09 (m, 22H, CH_2 + CH_3), 1.77-1.58 (m, 4H, CH_2), 1.42-1.33 (m, 4H, CH_2) ppm.

^{19}F NMR (376 MHz, CDCl$_3$): δ -80.76 (m, 3F) -114.57 (m, 2F), -121.91 (m, 2F), -122.88 (m, 2F), -123.60 (m, 2F), -126.14 (m, 2F) ppm.

^{13}C NMR (100.5 MHz, CDCl$_3$): δ 182.1, 139.2, 138.7, 137.6, 137.2, 136.1, 134.7, 134.4, 133.3, 132.4, 130.1, 129.8, 128.6, 128.3, 120.5, 83.1, 82.4, 53.6, 51.9, 51.1, 34.1, 33.2, 29.5, 29.1, 28.2, 21.3, 19.9, 18.4, 16.1 δ ppm.

HRMS (ESI+): calcd for [M-Cl]$^+$ = $C_{37}H_{39}F_{13}N_2Ir^+$: 951.2535. Found: 951.2605, calcd for [M-Cl+CH_3CN]$^+$ = $C_{39}H_{42}F_{13}N_3Ir^+$: 992.2800. Found: 992.2658.

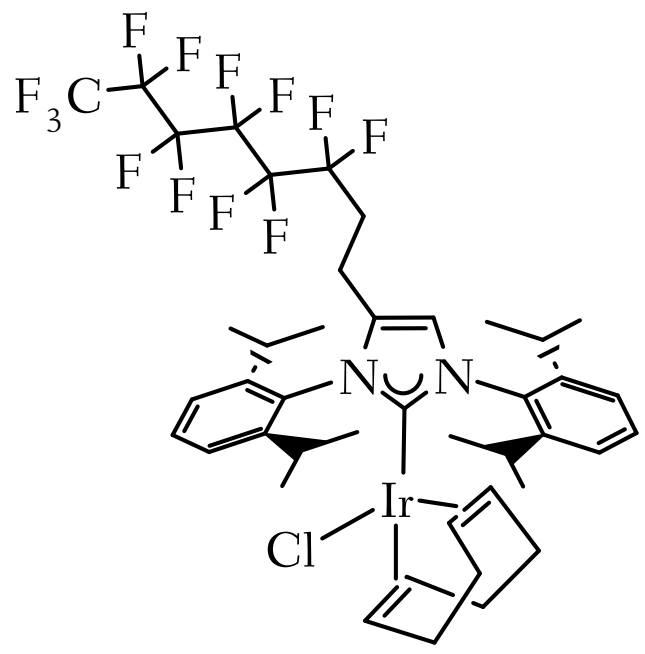

Ir-IPrF 1

¹H NMR (400 MHz, CDCl$_3$): δ 7.53 (t, J=7.8 Hz, 1H, Ar), 7.45 (t, J=7.8Hz, 1H, Ar), 7.48-7.18 (br, 4H, Ar), 6.85 (m, 1H, CH_{Im}), 4.35-4.06 (m, 2H, CH), 3.67-3.26 (m, 2H, CH_{iPr}), 3.17-2.68 (m, 4H, 2CH +2CH_{iPr}), 2.65-2.04 (m, 4H, CH_2), 1.78-0.88 (m, 32H, CH_2+ CH_3) ppm.

¹⁹F NMR (376 MHz, CDCl$_3$) δ -80.78 (m, 6F) -114.49 (m, 4F), -121.92 (m, 4F), -122.87 (m, 2F), -123.37 (m, 2F), -126.15 (m, 4F) ppm.

¹³C NMR (100.5 MHz, CDCl$_3$) δ 184.2, 146.9, 136.5, 133.1, 130.4, 129.9, 124.4, 122.1, 84.3, 81.1, 53.9, 49.0, 35.2, 32.3, 32.1, 31.1, 29.8, 29.3, 28.5, 25.5, 16.5, 14.3 ppm.

HRMS (ESI+): calcd for $[M-Cl]^+$ = $C_{43}H_{51}F_{13}N_2Ir^+$: 1035.3474. Found: 1035.3474, calcd for $[M-C_8H_{12}ClIr]^+$ = $C_{35}H_{40}F_{13}N_2^+$: 735.2978. Found 735.2922.

3. Crystallographic data

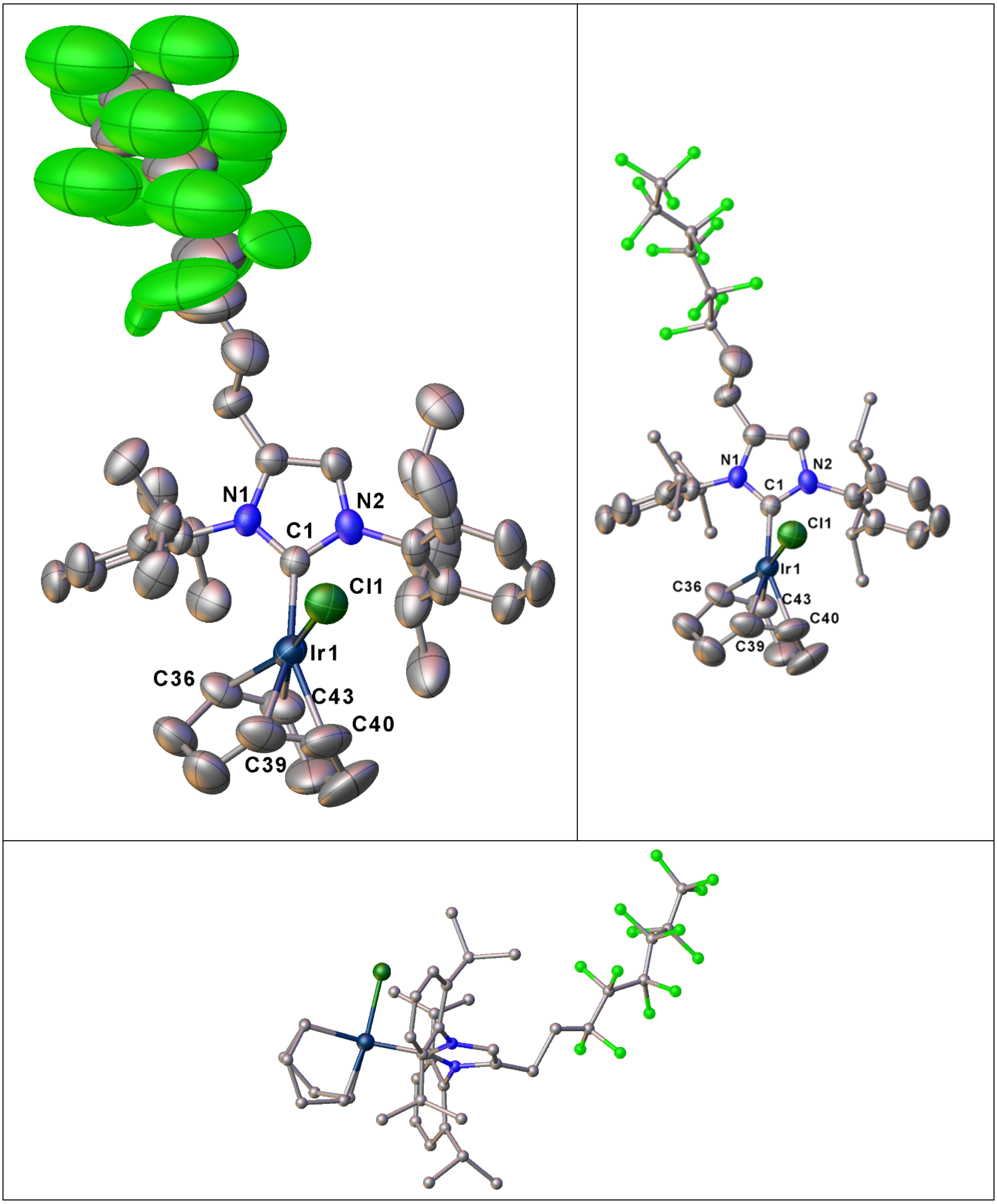

Figure S1: ORTEP view of complex **Ir-IPrF$_1$**. Ellipsoids are shown at 50% probability levels. Hydrogen atoms have been omitted for clarity. Selected bond distances (Å) and angles (°): C1-Ir1 2.059(8), Ir1-Cl1 2.342(3), Ir1-C39 2.184(12), C36-Ir1 2.10(1), Ir1-C40 2.155(13), Ir1-C43 2.079(12), C1-Ir-Cl1 87.4(2), C1-Ir1-C40 156.8(6), C39-Ir1-C1 163.6(5), C1-Ir1-C36 96.9(4), C1-Ir1-C43 98.8(4). Right: same figure but with the atoms of isopropyl groups

and the fluorinated chain shown in balls and sticks only to aid clarity. Below: balls and sticks view of the complex, to highlight the square planar coordination around the iridium metal centre.

Crystals of complex **Ir-IPrF$_1$** suitable for SC-XRD were isolated by slow evaporation of a pentane solution of the complex. A translucent light colourless, Frag (Prism)-shaped crystal was mounted on the goniometer. Data were collected from a single crystal at 150.00 K on a Bruker D8 KAPPA diffractometer with a microfocus sealed tube using a multilayer mirror as monochromator and a PhotonIII_C14 CPAD detector. The diffractometer was equipped with a Cu$K_{α}$ radiation (λ = 1.54178 Å). All data were integrated with SAINT V8.42, yielding 189197 reflections of which 9730 where independent (average redundancy 19.44) and 86.0% were greater than 2σ(F^2).[5] A Multi-Scan absorption correction using SADABS 2016/2 was applied.[6] The structure was solved by dual methods with SHELXT 2018/2 and refined by full-matrix least-squares methods against F^2 using SHELXL 2019/3.[7,8] All non-hydrogen atoms were refined with anisotropic displacement parameters. All hydrogen atoms were refined isotropic on calculated positions using a riding model with their U_{iso} values constrained to 1.5 times the U_{eq} of their pivot atoms for terminal sp^3 carbon atoms and 1.2 times for all other carbon atoms.

CCDC number	2551245
Empirical formula	$C_{43}H_{51}ClF_{13}IrN_2$
Formula weight	1070.51
Temperature [K]	150.00
Crystal system	monoclinic
Space group (number)	$P2_1/n$
a [Å]	11.3717(7)
b [Å]	14.2886(9)
c [Å]	27.6501(18)
α [°]	90
β [°]	90.024(3)
γ [°]	90
Volume [Å^3]	4492.7(5)
Z	4
$ρ_{calc}$ [gcm^{-3}]	1.583

μ [mm^{-1}]	7.066
F(000)	2136
Crystal size [mm^3]	0.04×0.095×0.16
Crystal colour	translucent light colourless
Crystal shape	Frag (Prism)
Radiation	CuK_α (λ=1.54178 Å)
2θ range [°]	6.392 to 159.692 (0.79 Å)
Index ranges	$-14 \le h \le 14$<br>$-18 \le k \le 16$<br>$-35 \le l \le 35$
Reflections collected	189197
Independent reflections	9730<br>R_{int} = 0.0686<br>R_{sigma} = 0.0222
Completeness to θ = 67.679°	99.7
Data / Restraints / Parameters	9730 / 204 / 471
Goodness-of-fit on F^2	1.119
Final R indexes [$I \ge 2\sigma(I)$]	R_1 = 0.0999<br>wR_2 = 0.2788
Final R indexes [all data]	R_1 = 0.1108<br>wR_2 = 0.2988
Largest peak/hole [eÅ^{-3}]	3.60/−2.13

4. Characterization data for the synthesized compounds

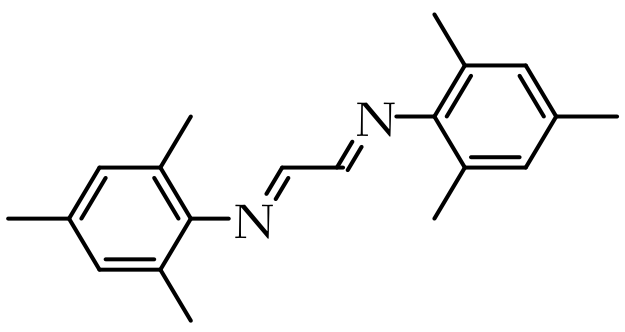

Ia

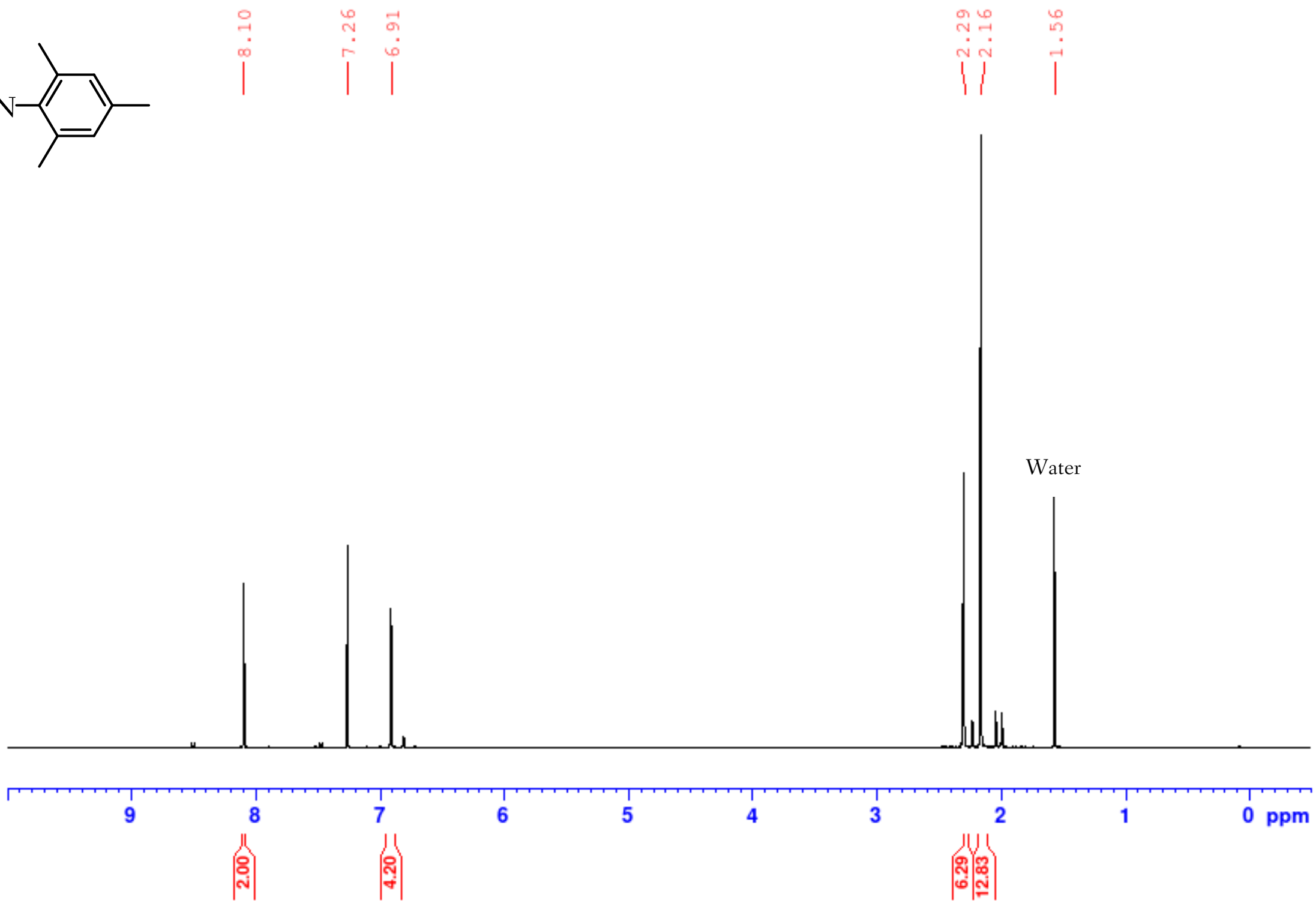

Figure S2. ^{1}H NMR Spectrum 400 MHz, $CDCl_3$, **Ia.**

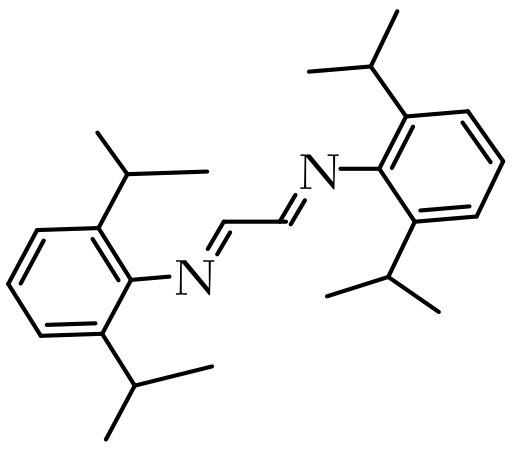

Ib

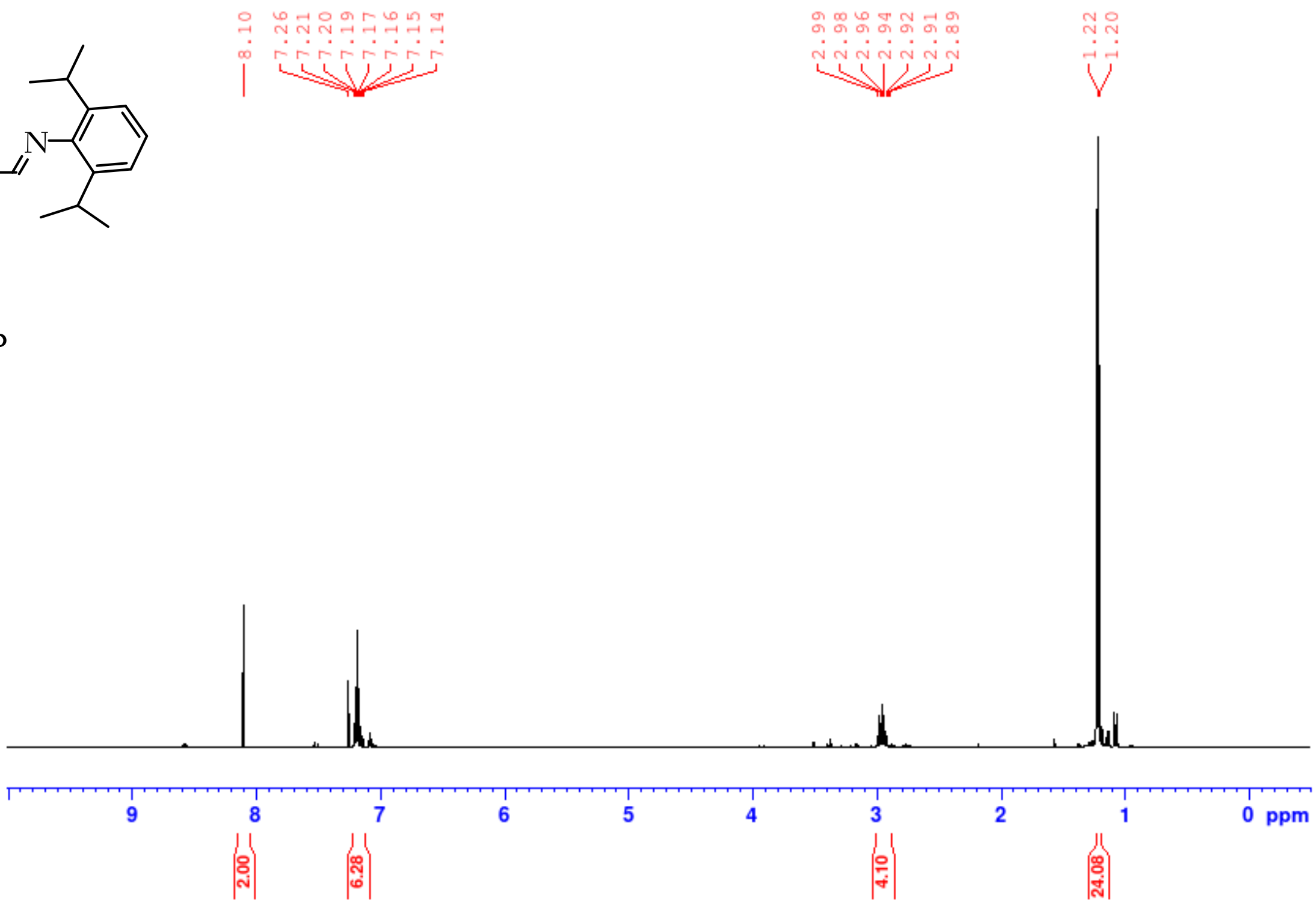

Figure S3. ^{1}H NMR Spectrum 400 MHz, $CDCl_3$, **Ib**.

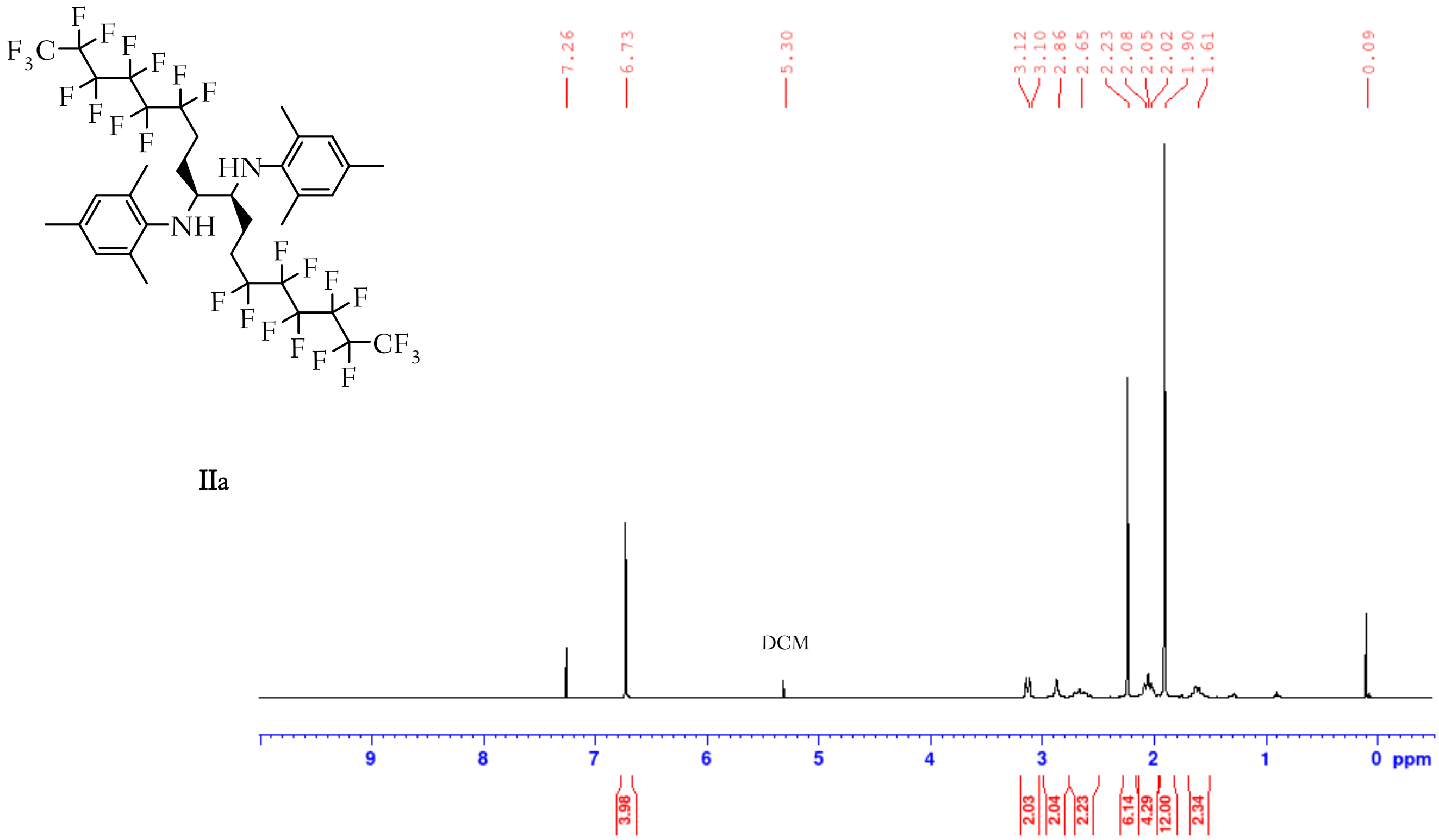

IIa

Figure S4. ^{1}H NMR Spectrum 400 MHz, $CDCl_3$, **IIa**.

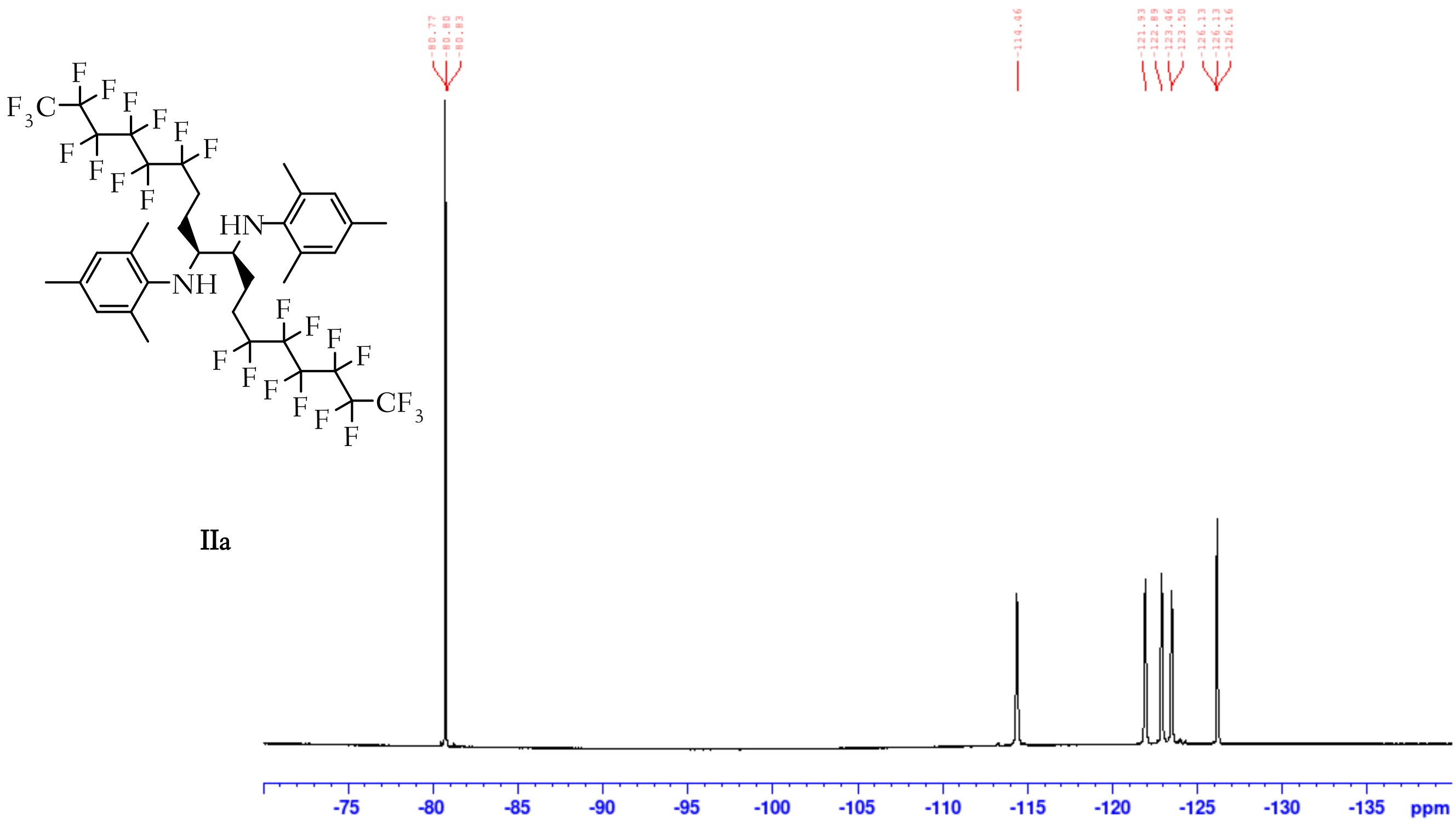

IIa

Figure S5. ^{19}F NMR Spectrum 376 MHz, $CDCl_3$, **IIa**.

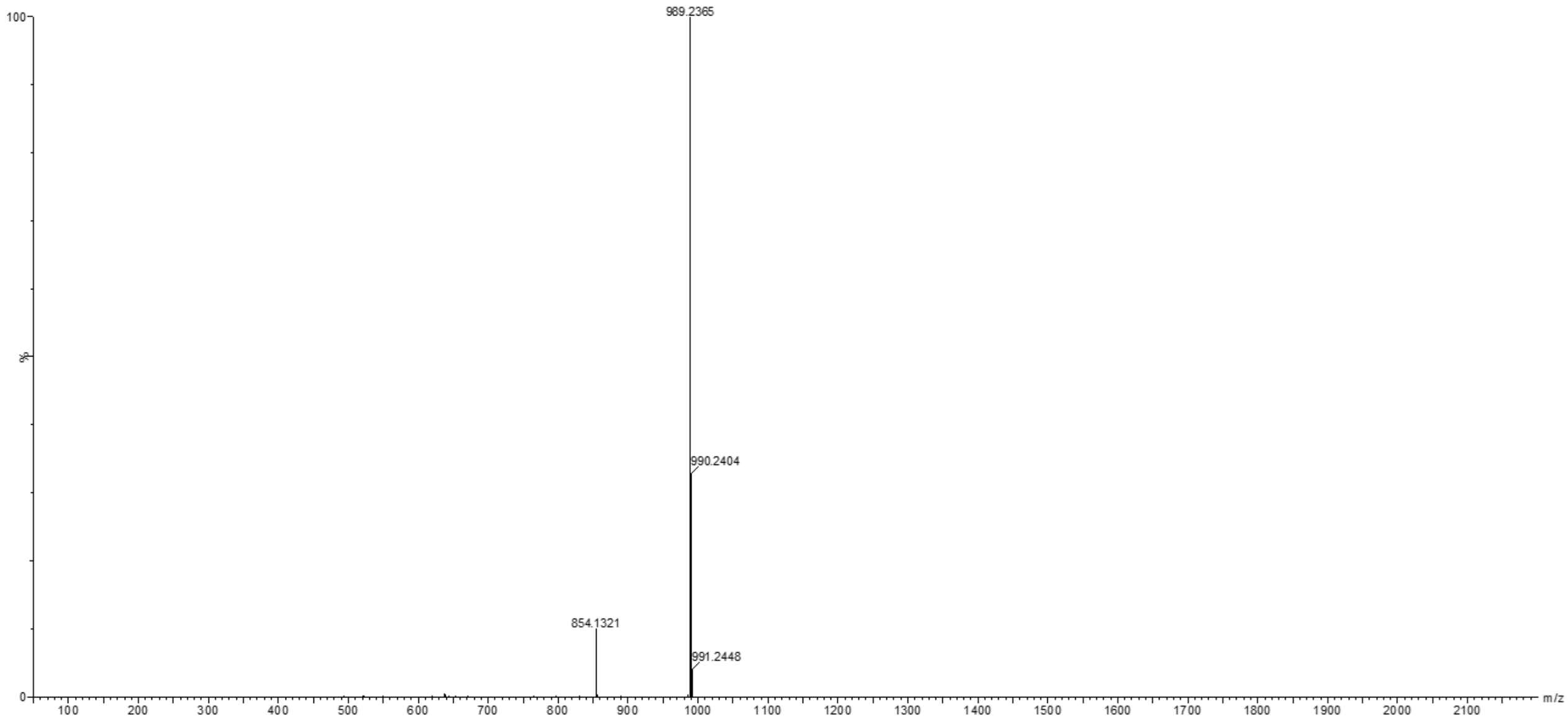

Figure S6. HRMS (ESI+), **IIa**.

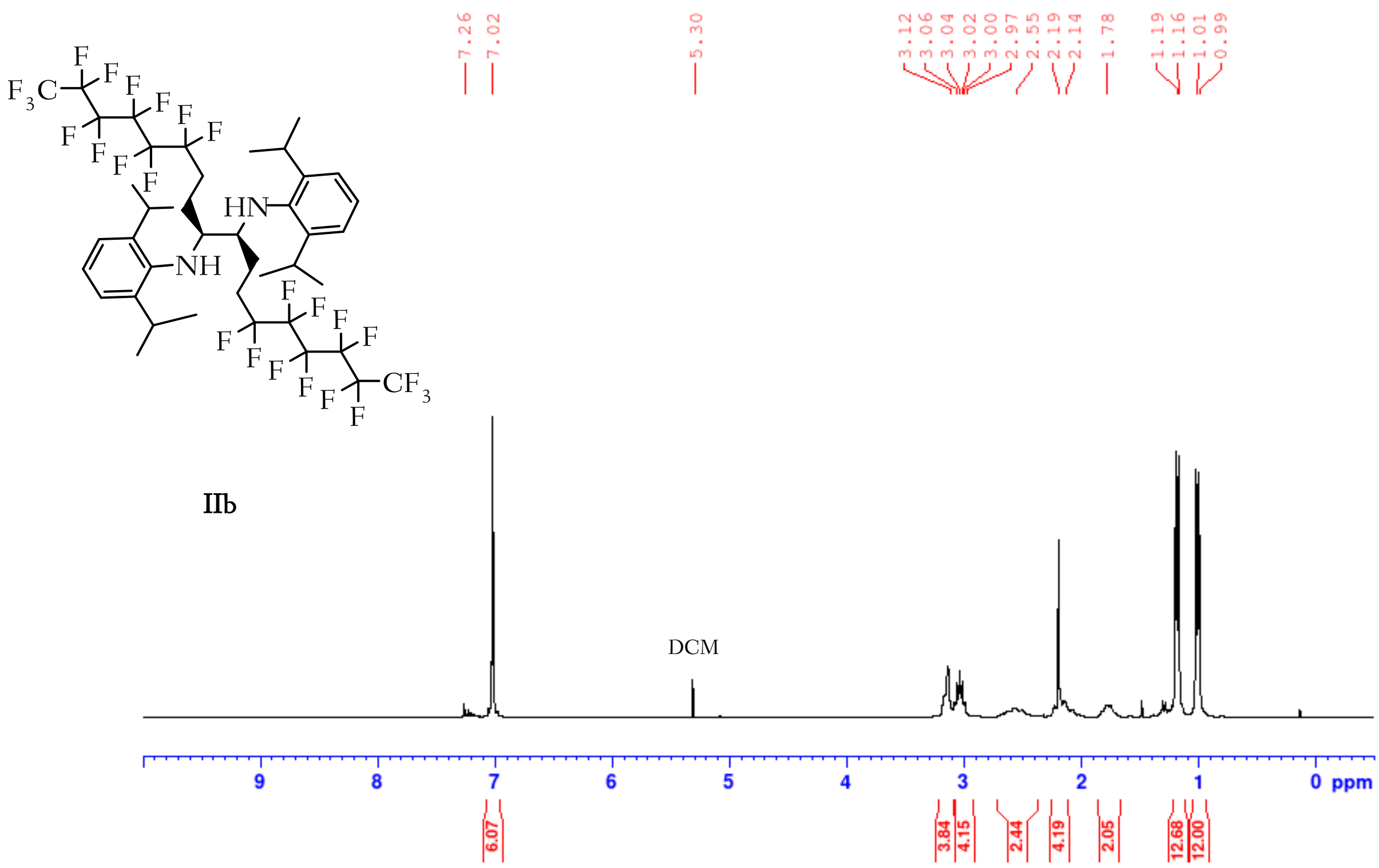

Figure S7. ^{1}H NMR Spectrum 400 MHz, $CDCl_3$, **IIb**.

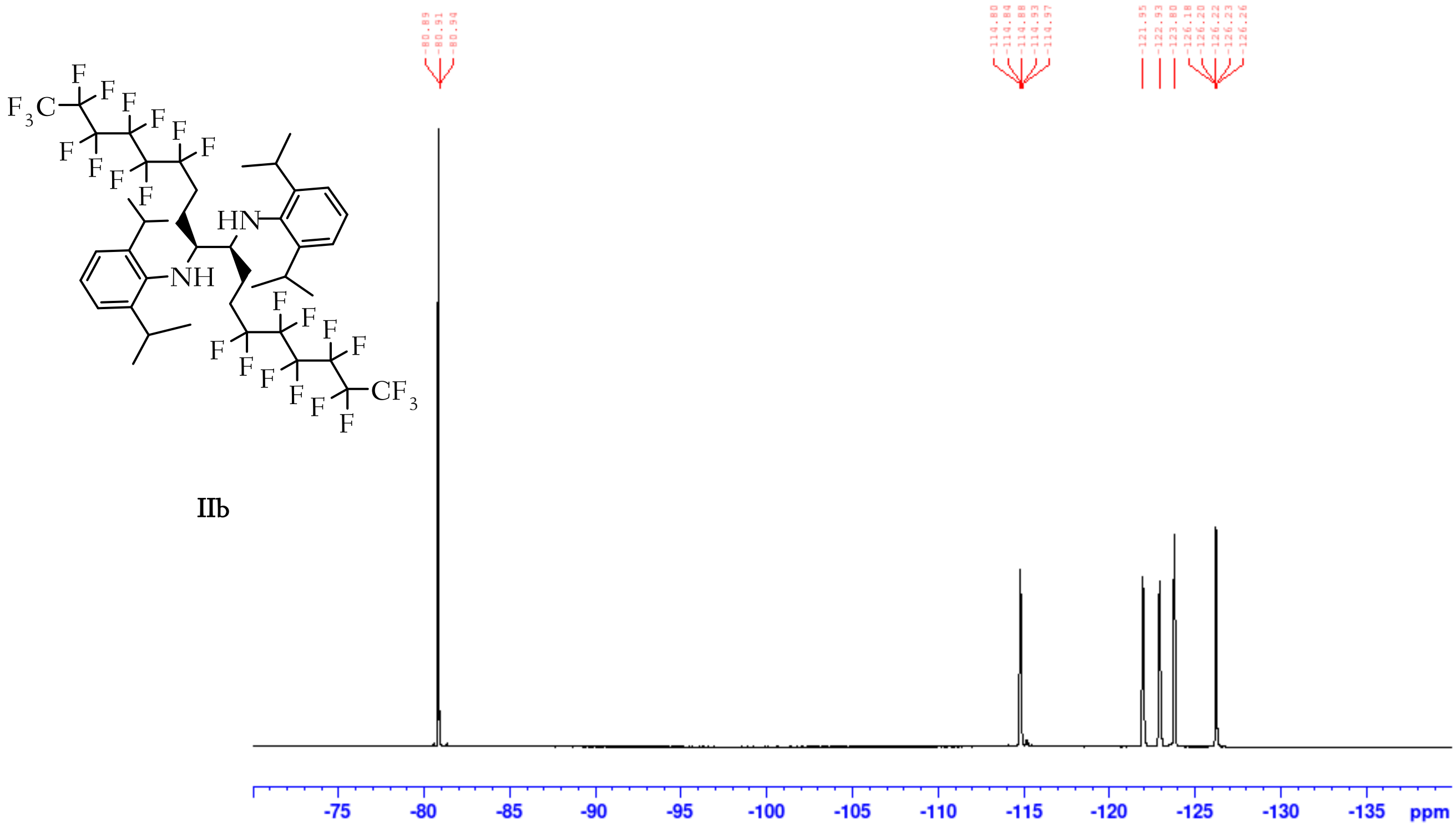

Figure S8. ^{19}F NMR Spectrum 376 MHz, $CDCl_3$, **IIb**.

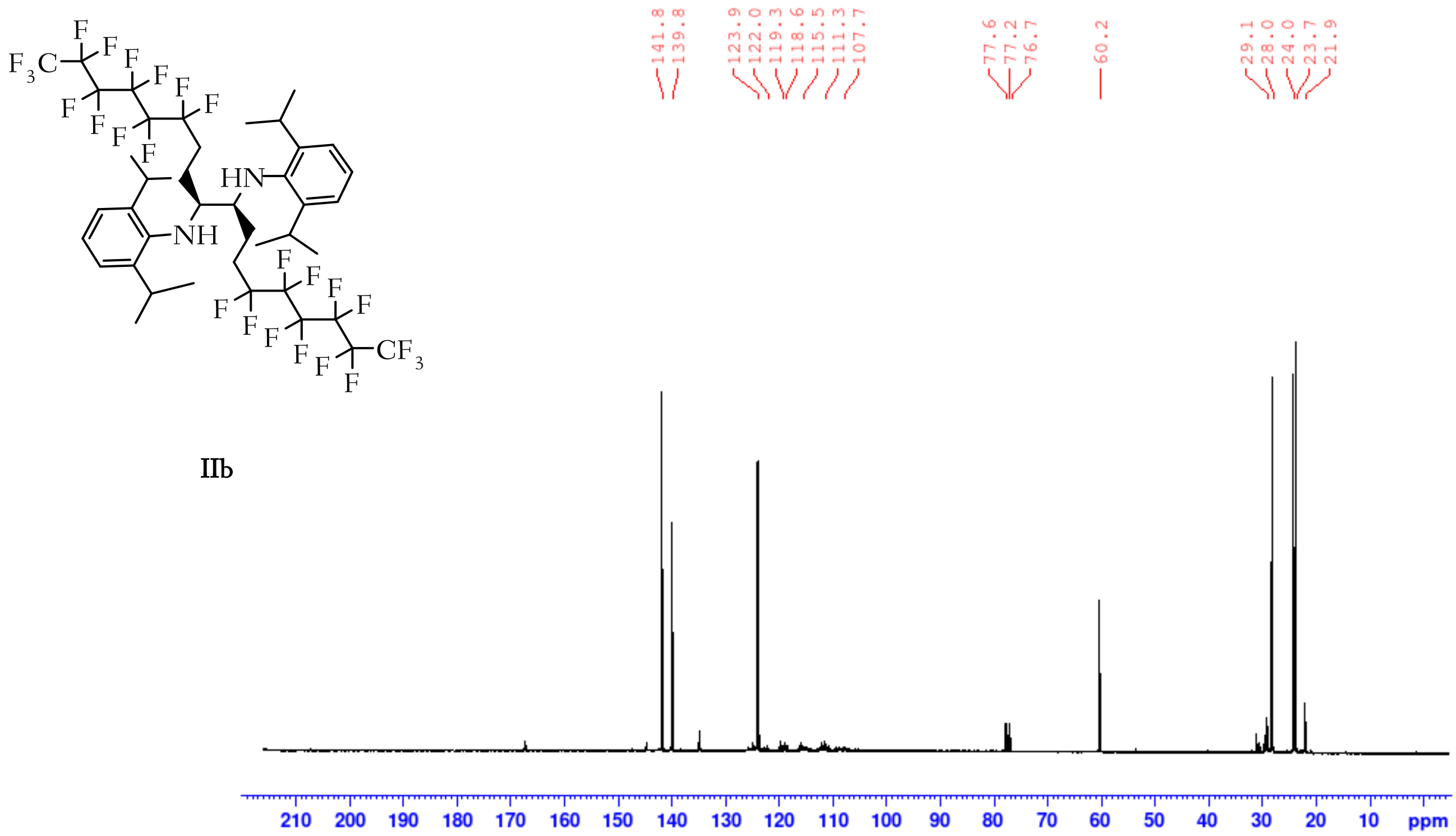

Figure S9. ^{13}C NMR Spectrum 100.5 MHz, $CDCl_3$, **IIb**.

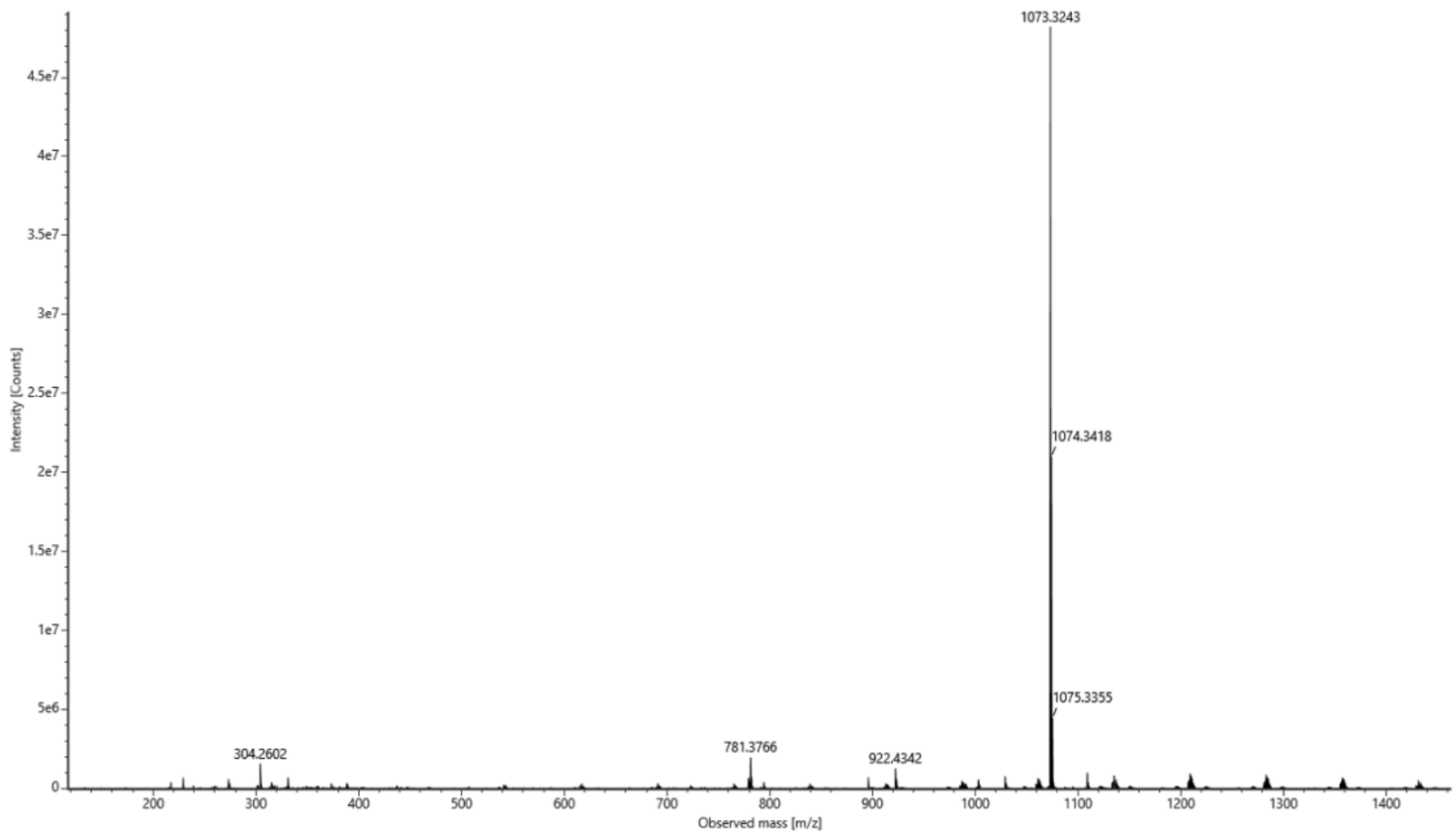

Figure S10. HRMS (ESI+), **IIb**.

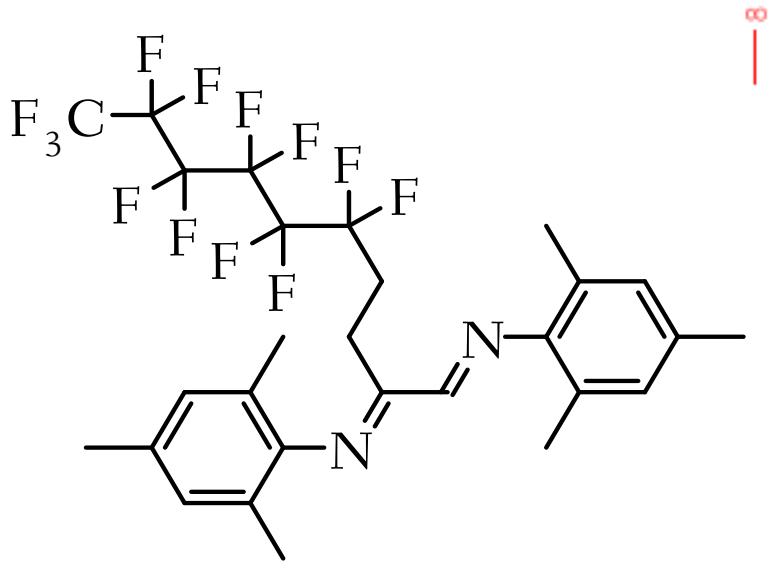

both isomers E/Z

IIIa

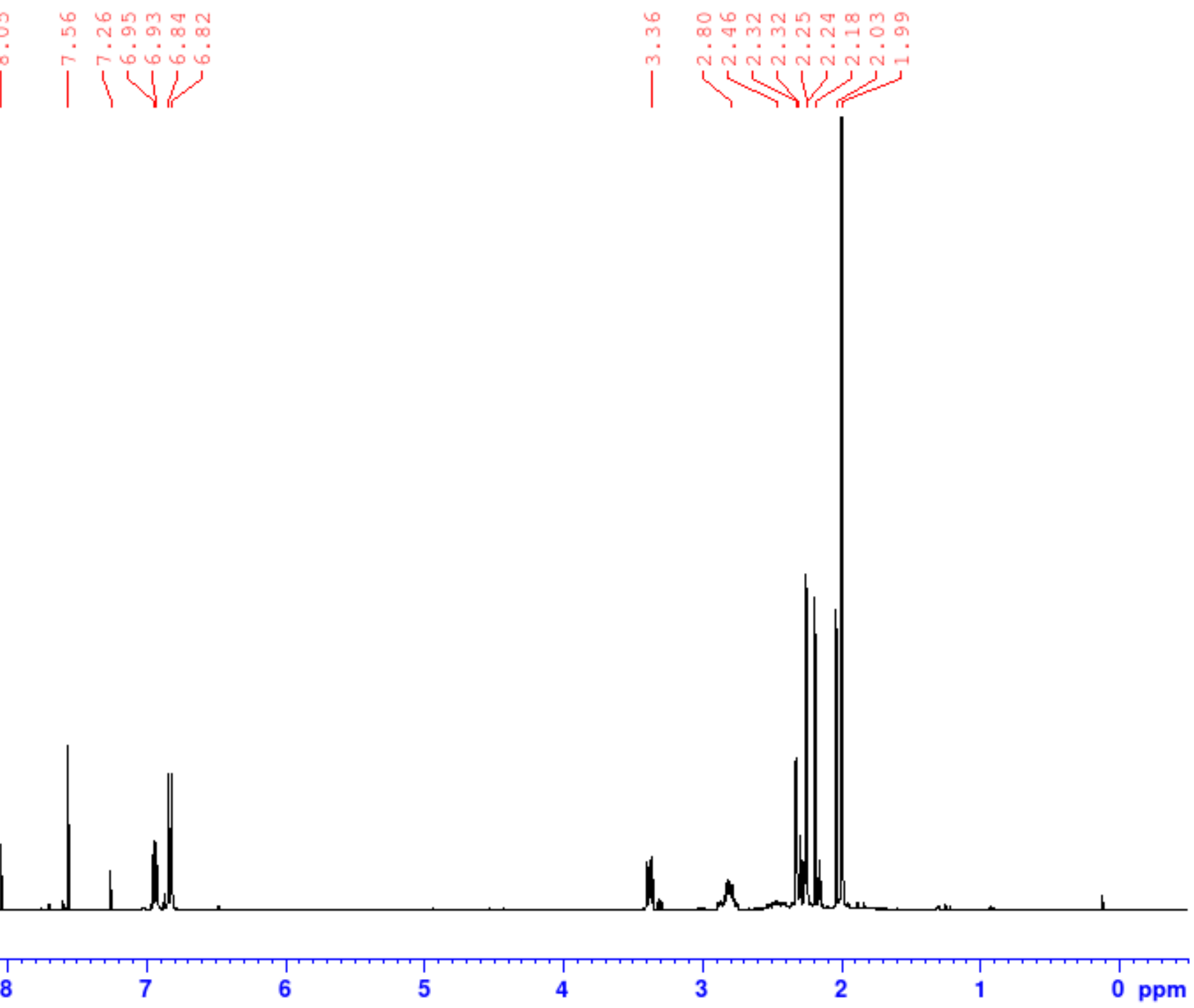

Figure S11. ^{1}H NMR Spectrum 400 MHz, $CDCl_3$, **IIIa**.

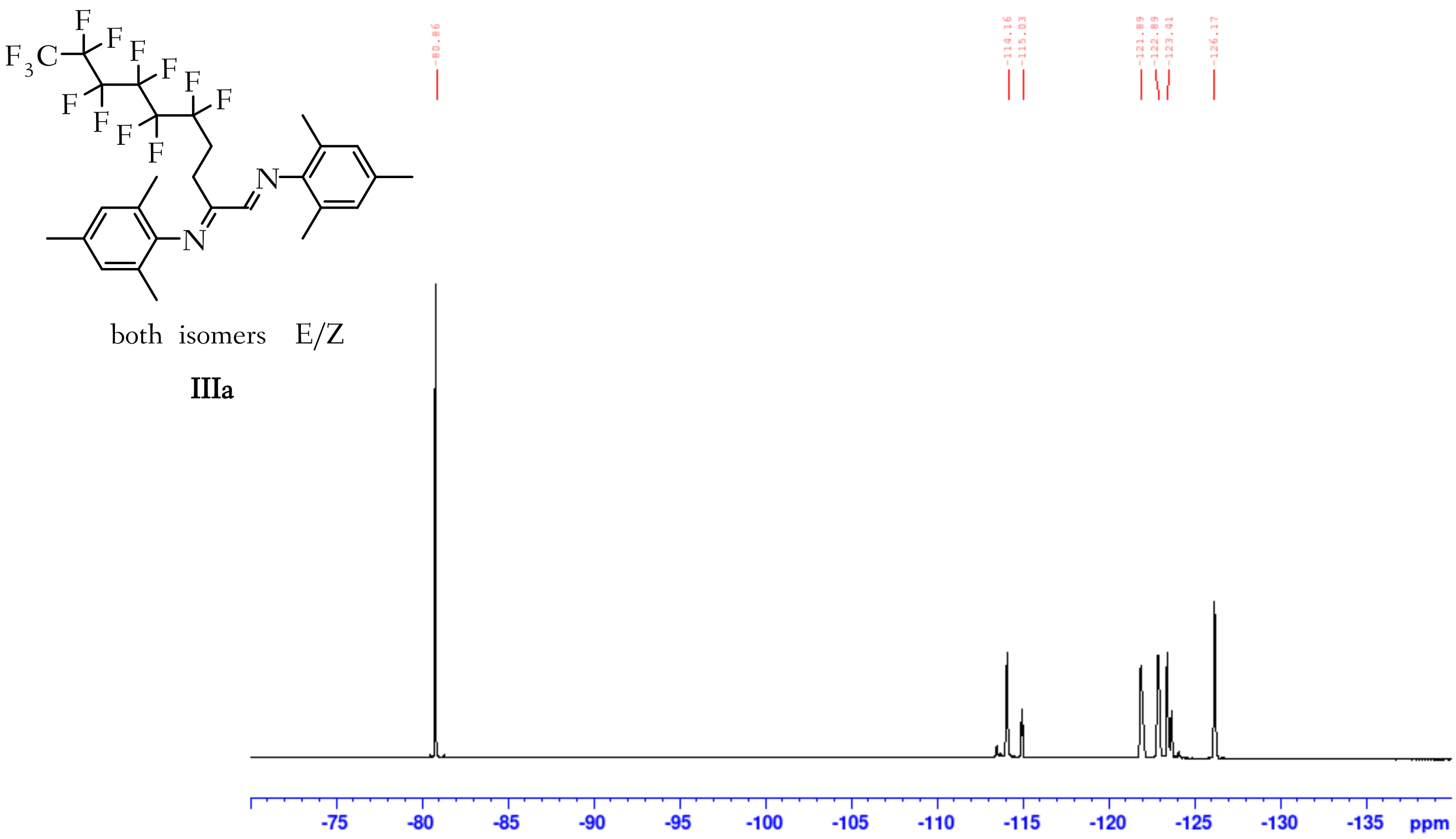

Figure S12. ^{19}F NMR Spectrum 376 MHz, $CDCl_3$, **IIIa**

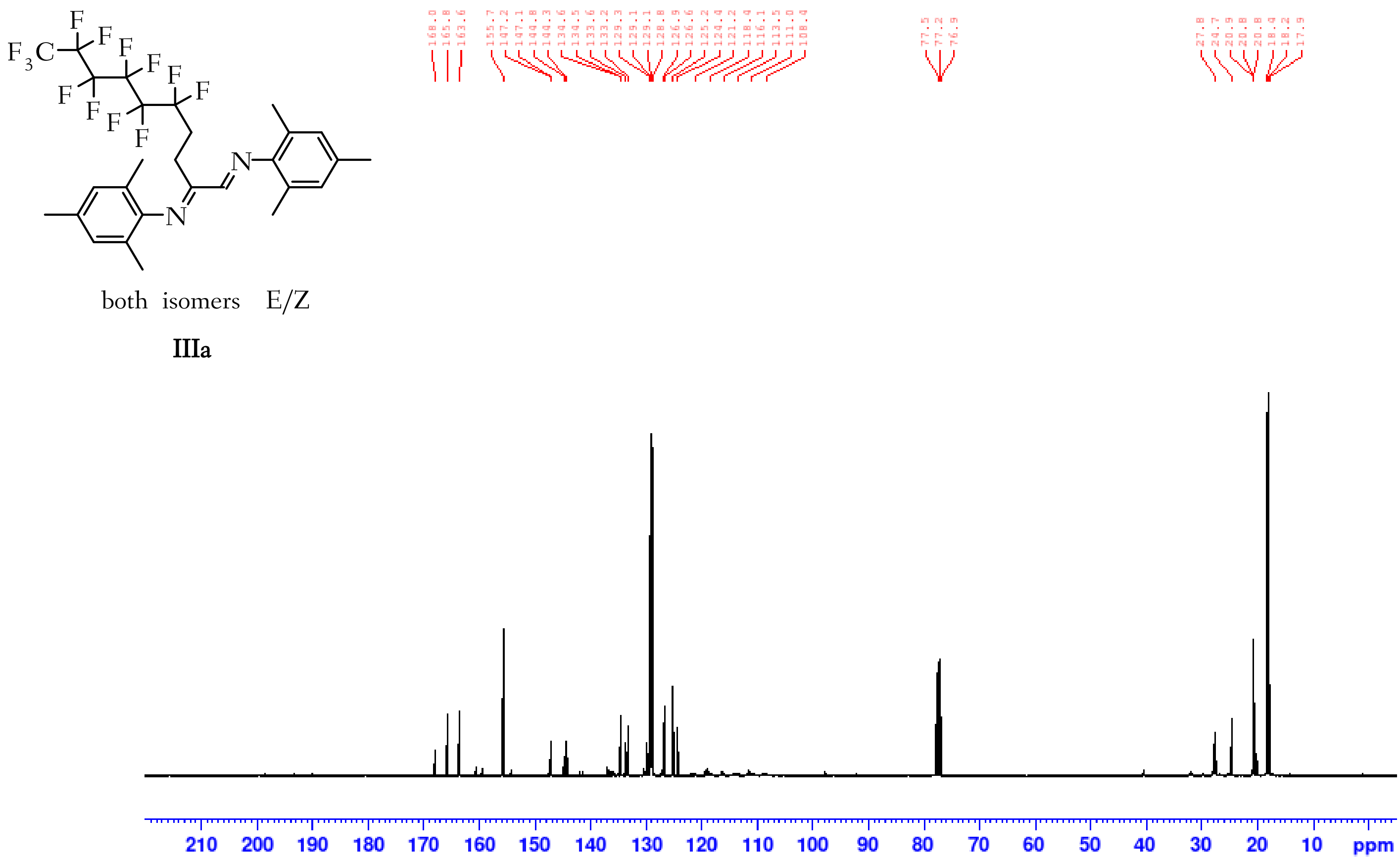

Figure S13. ^{13}C NMR Spectrum 100.5 MHz, $CDCl_3$, **IIIa**.

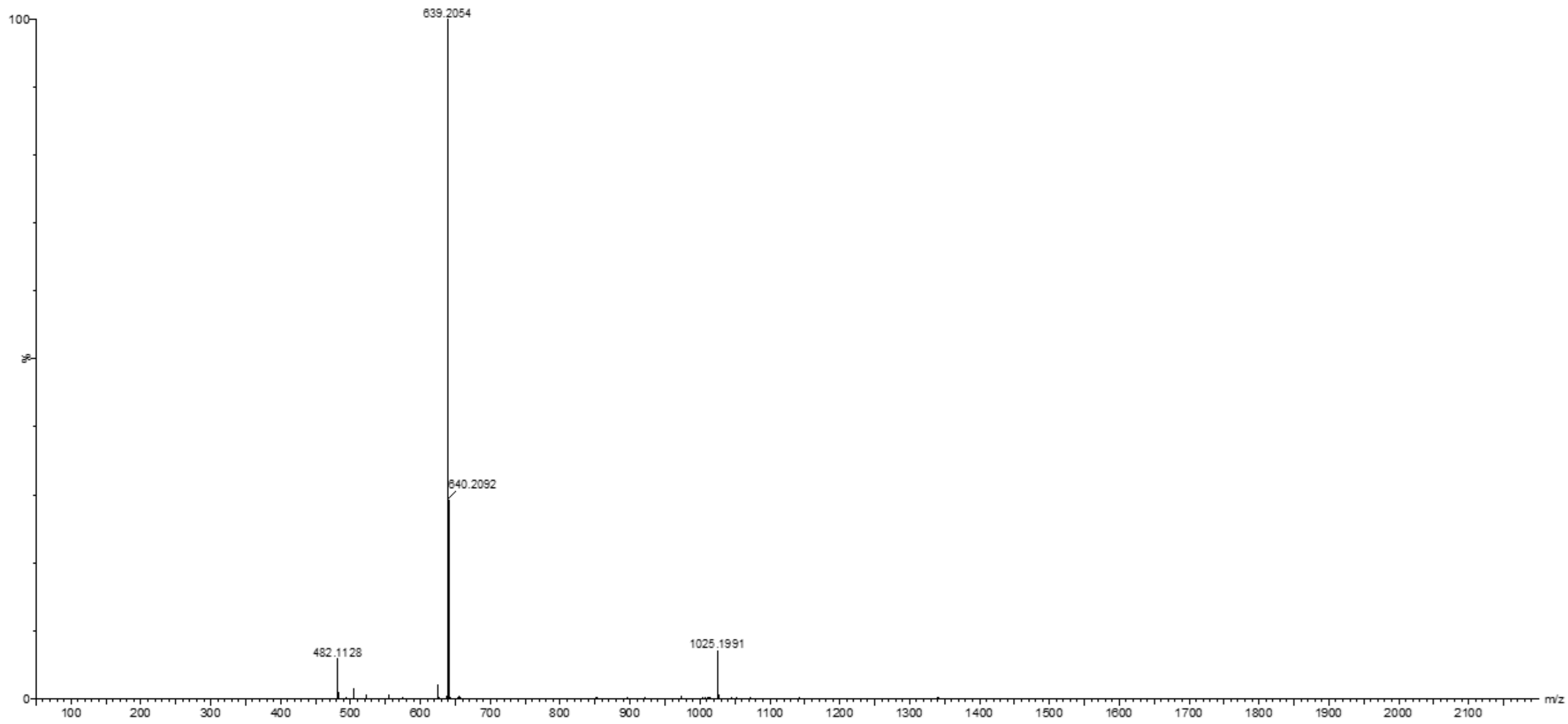

Figure S14. HRMS (ESI+), **IIIa**.

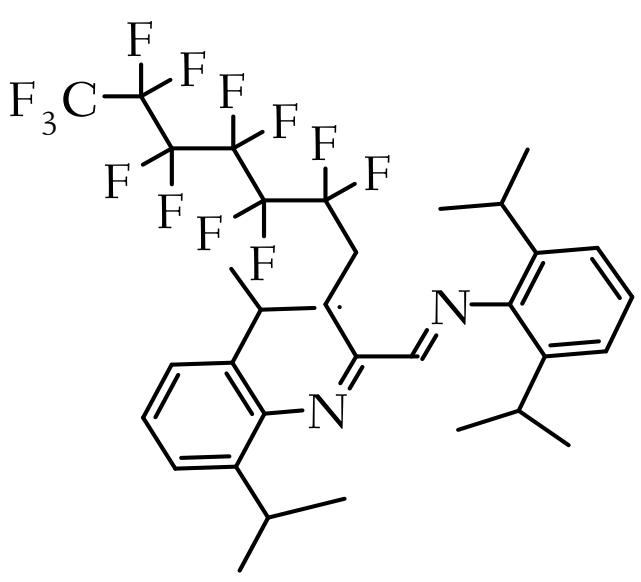

both isomers E/Z

IIIb

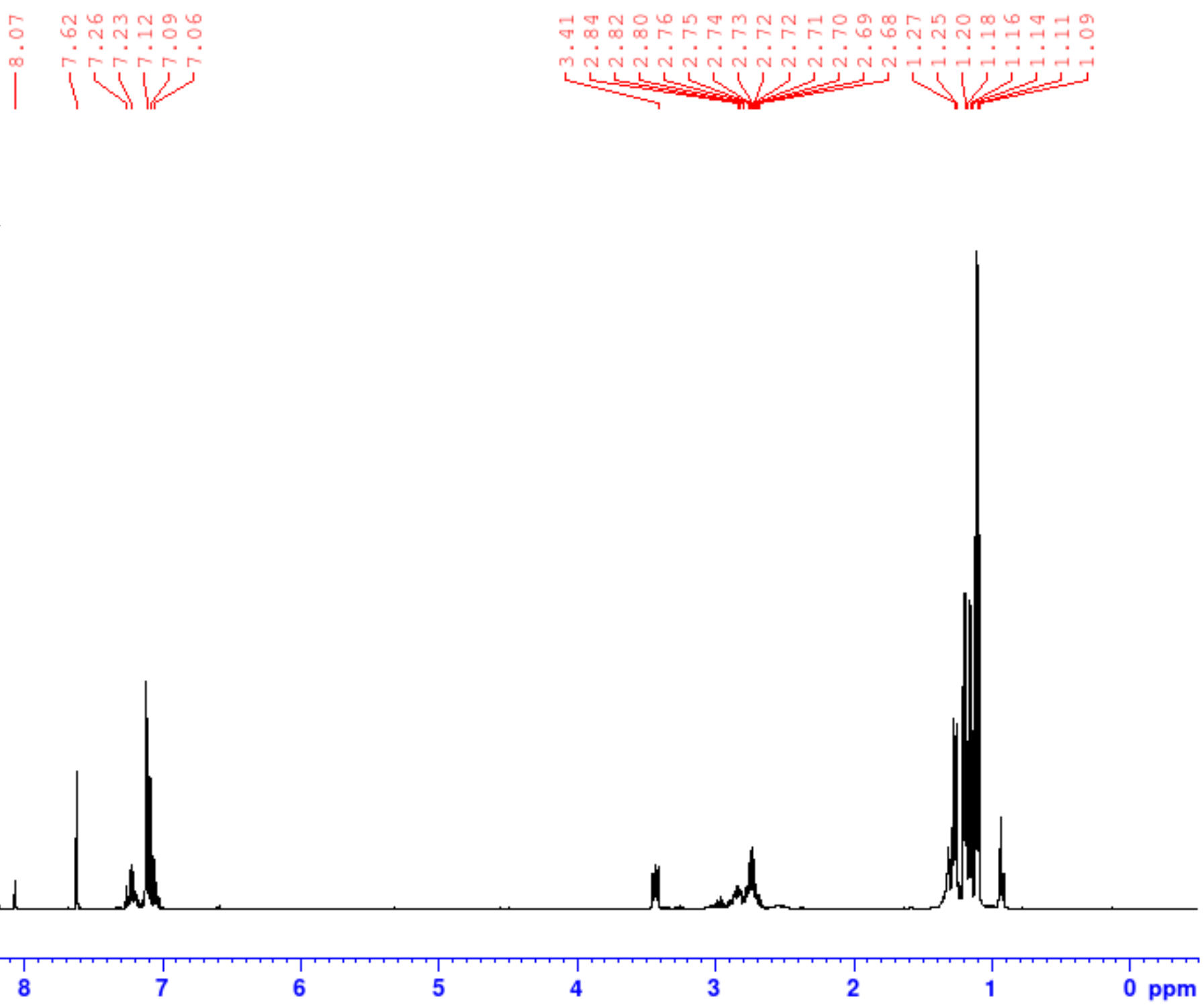

Figure S15. ^{1}H NMR Spectrum 400 MHz, $CDCl_3$, **IIIb**.

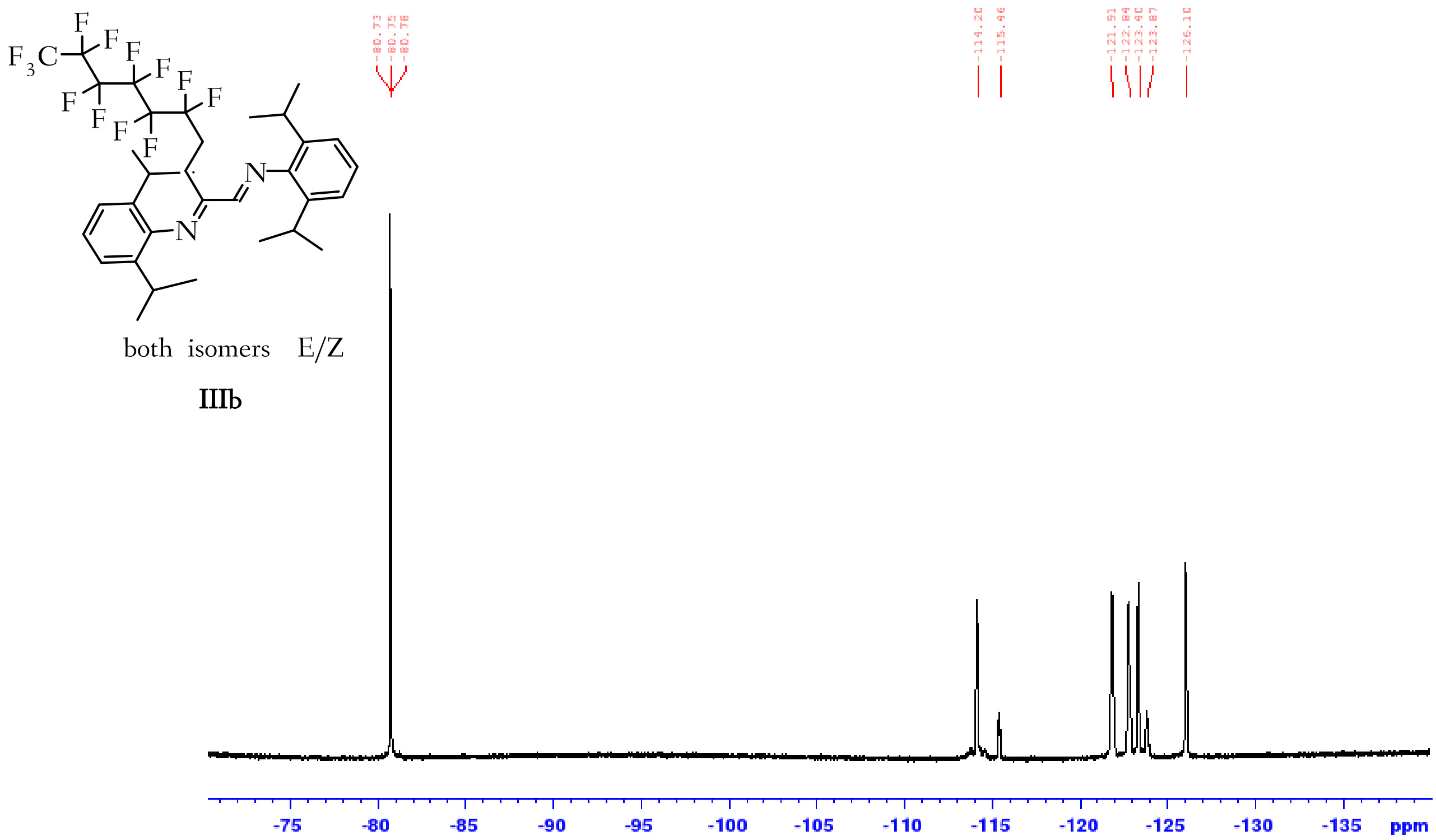

Figure S16. ^{19}F NMR Spectrum 376 MHz, $CDCl_3$, **IIIb**.

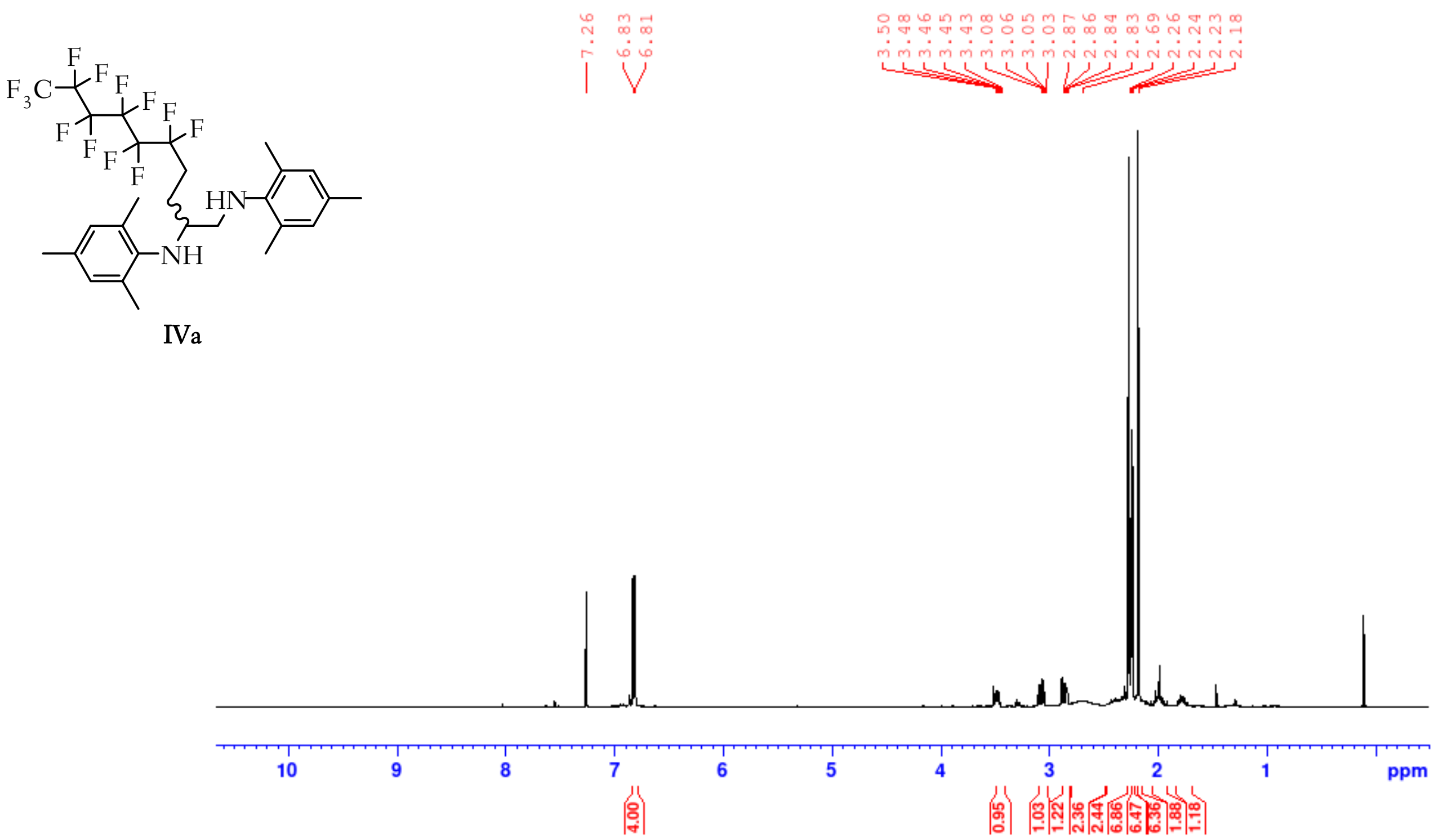

Figure S17. ^{1}H NMR Spectrum 400 MHz, $CDCl_3$, **IVa**.

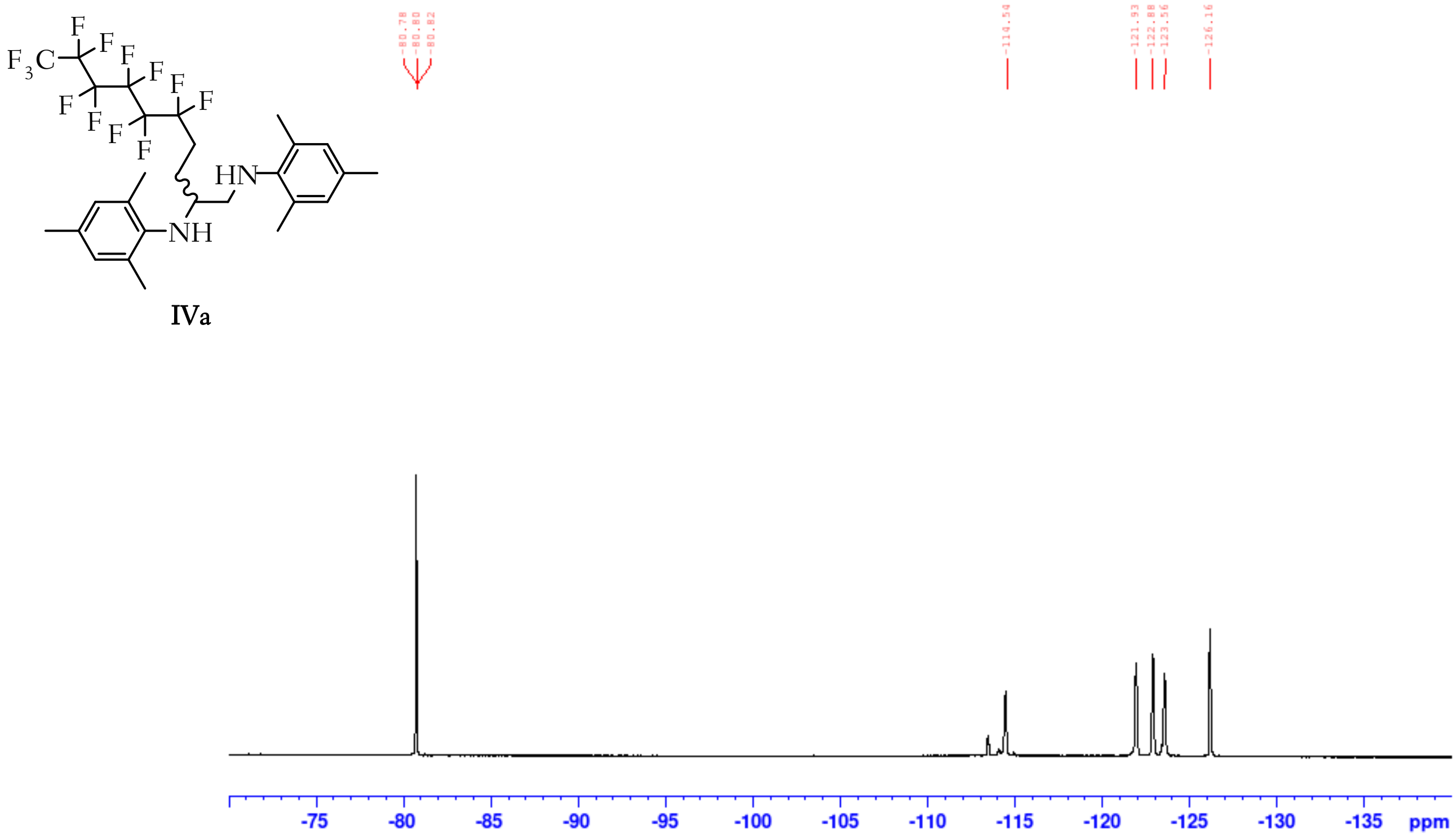

Figure S18. ¹⁹F NMR Spectrum 400 MHz, $CDCl_3$, **IVa**.

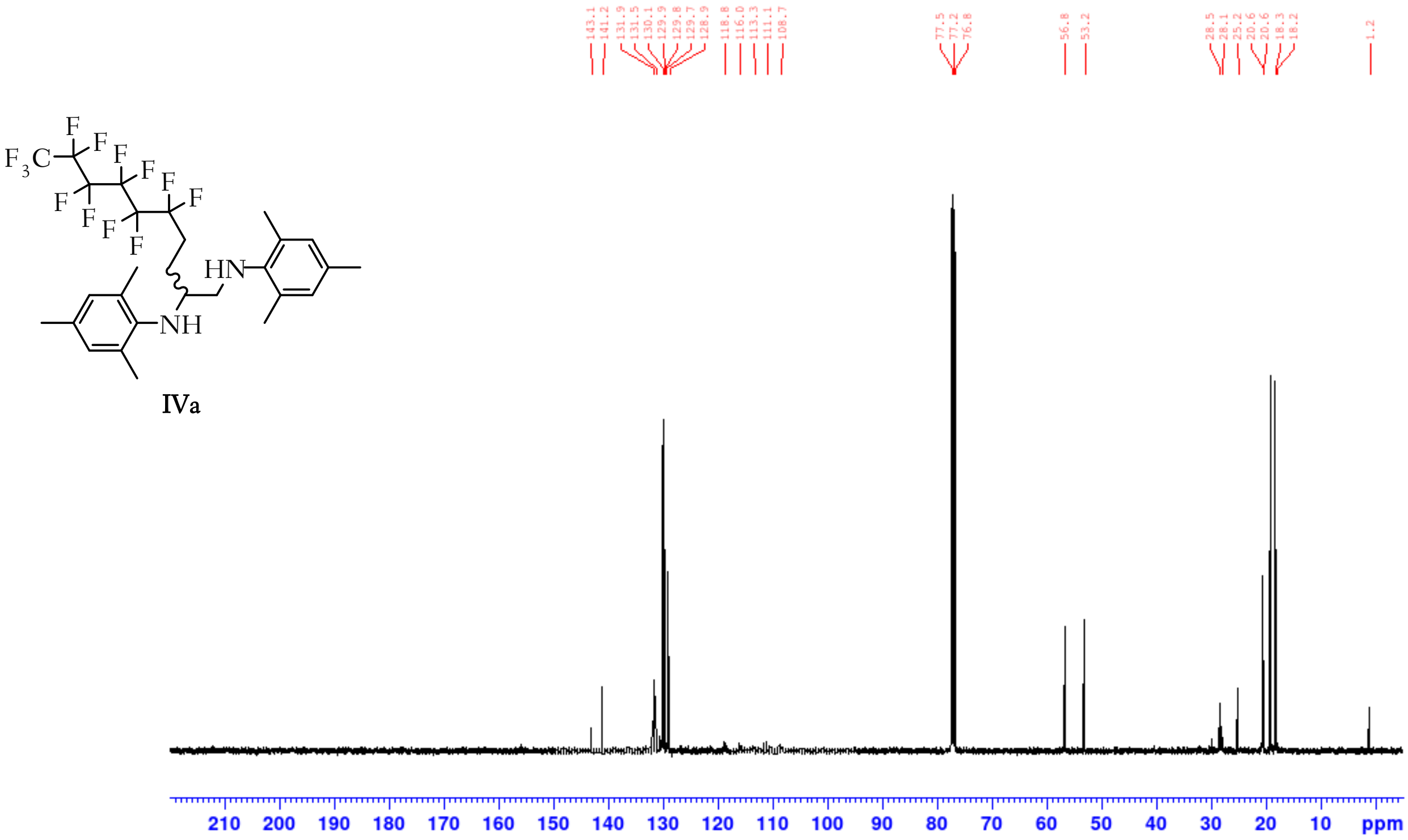

Figure S19. ¹³C NMR Spectrum 400 MHz, $CDCl_3$, **IVa**.

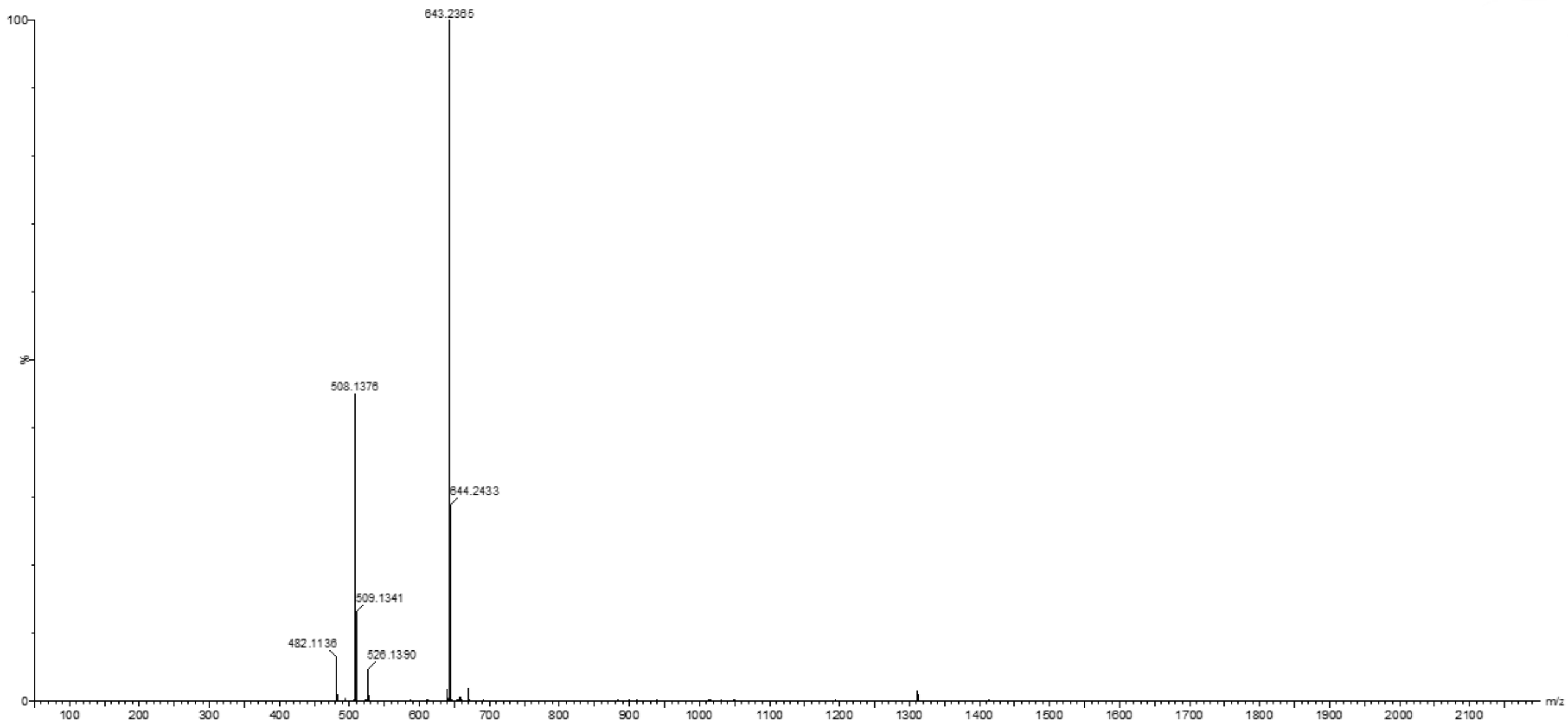

Figure S20. HRMS (ESI+), **IVa**.

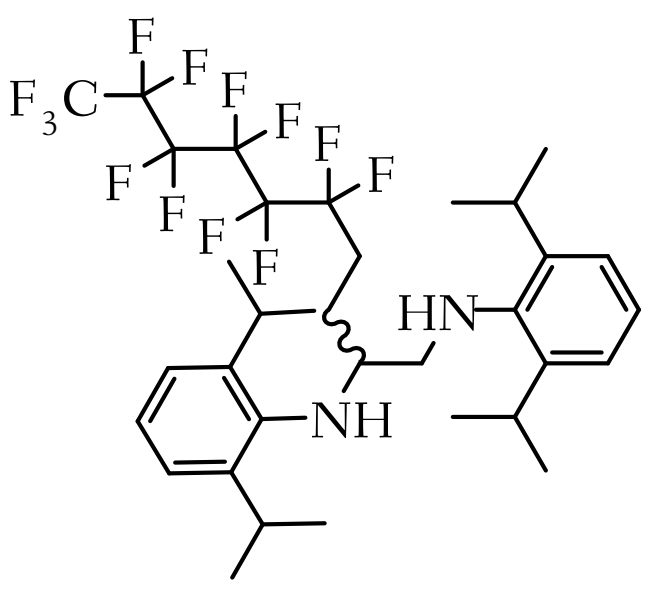

IVb

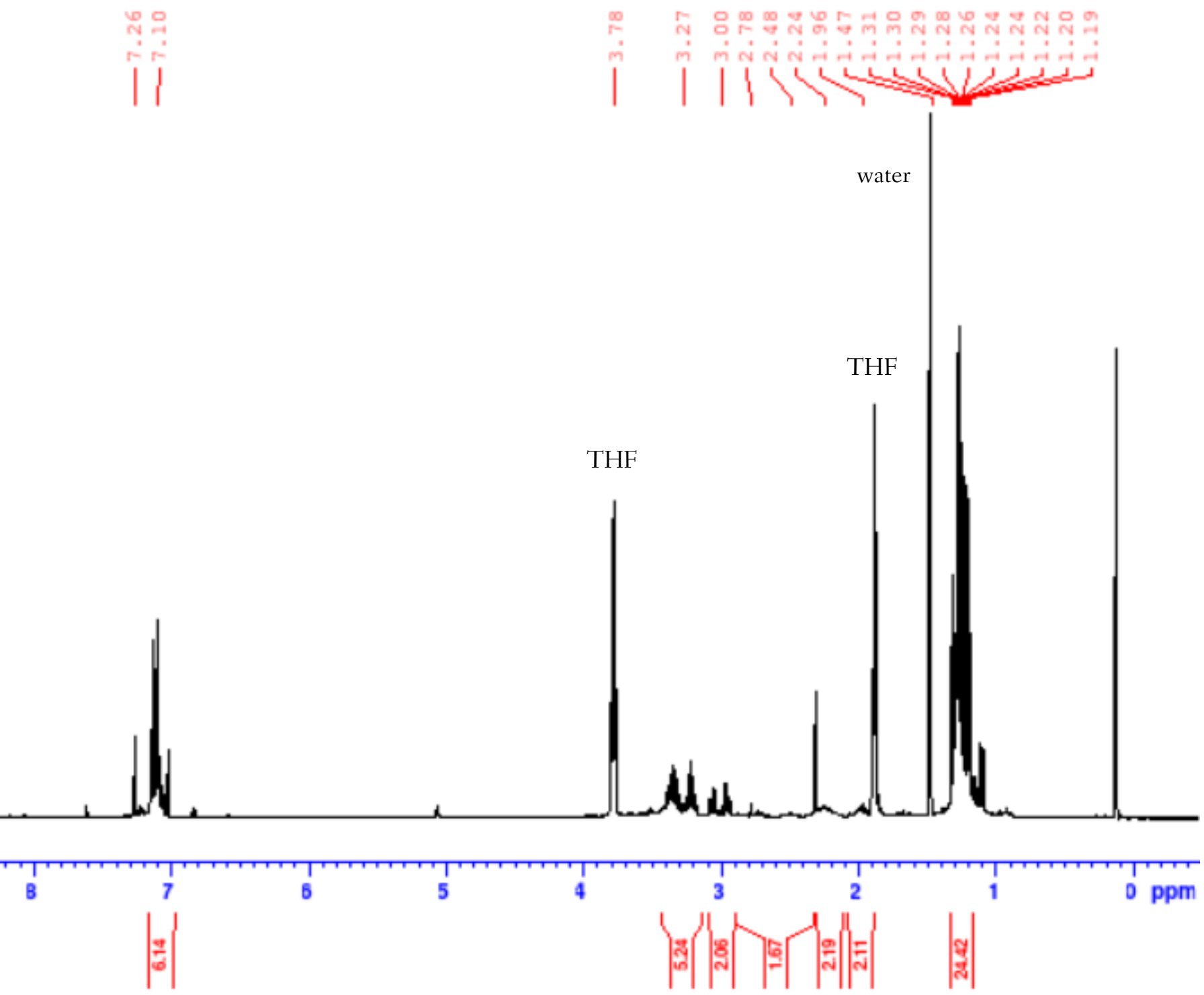

Figure S21. ^{1}H NMR Spectrum 400 MHz, $CDCl_3$, **IVb**.

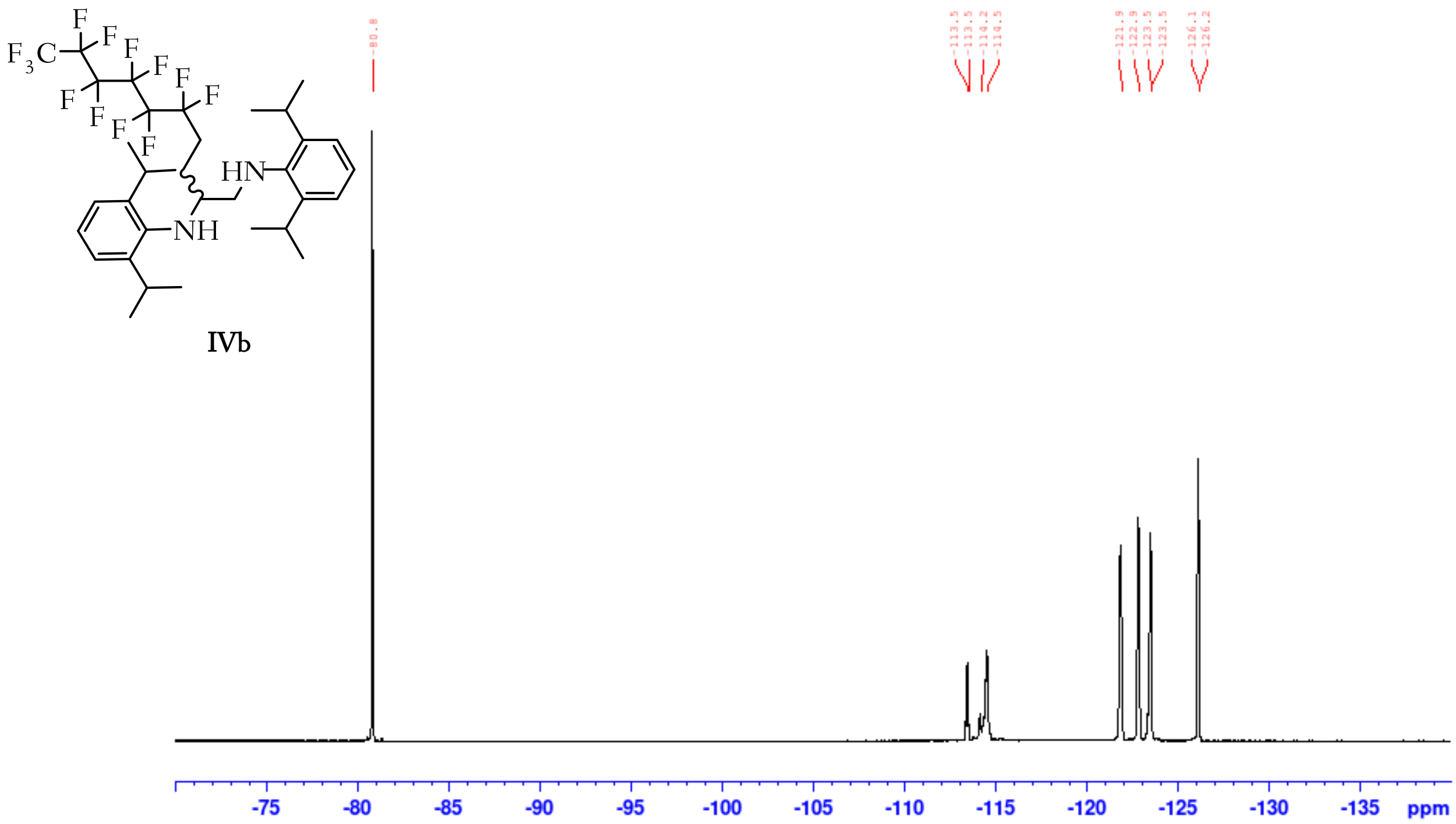

Figure S22. ^{1}H NMR Spectrum 400 MHz, $CDCl_3$, **IVb**.

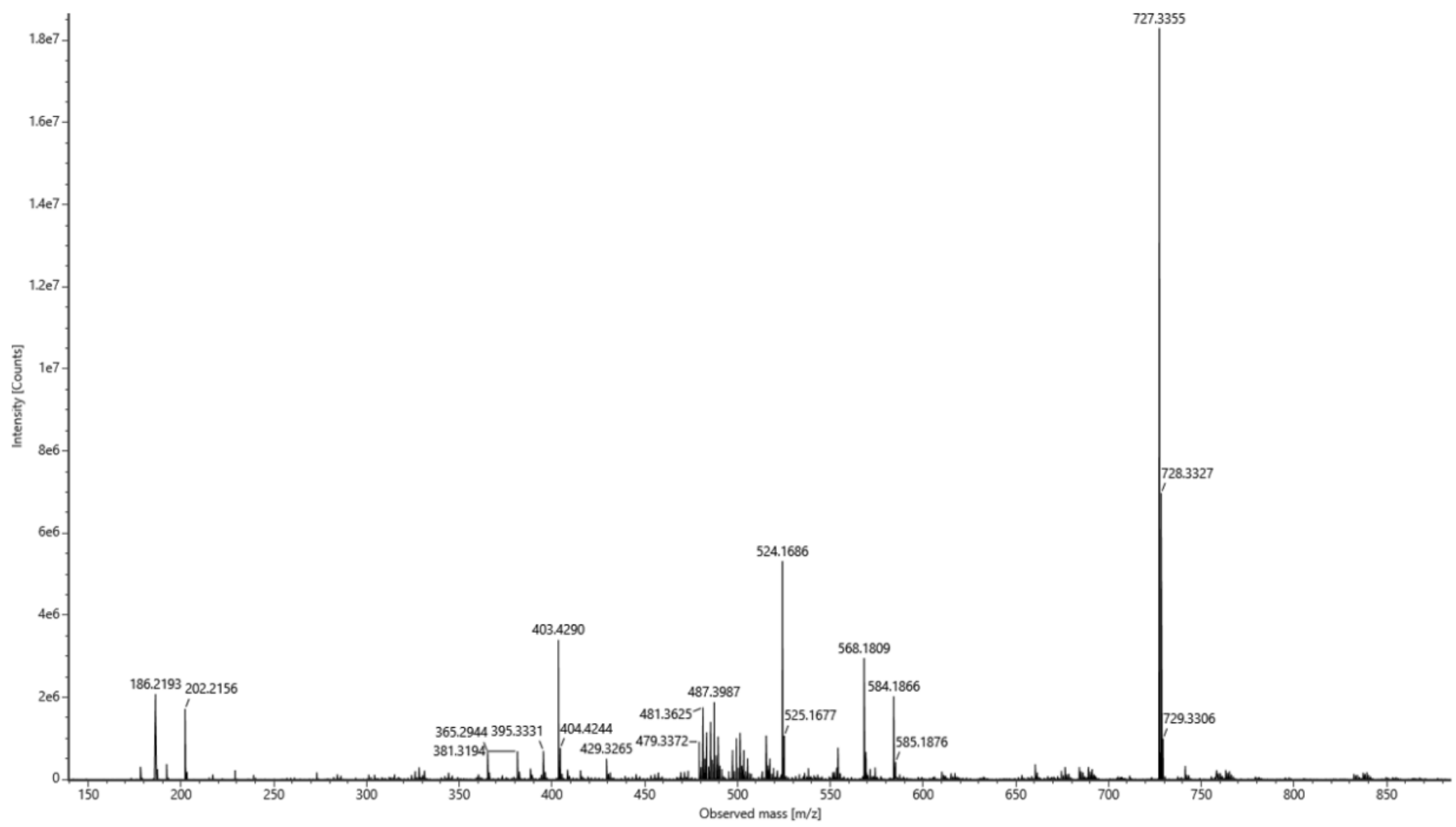

Figure S23. HRMS (ESI+), **IVb**.

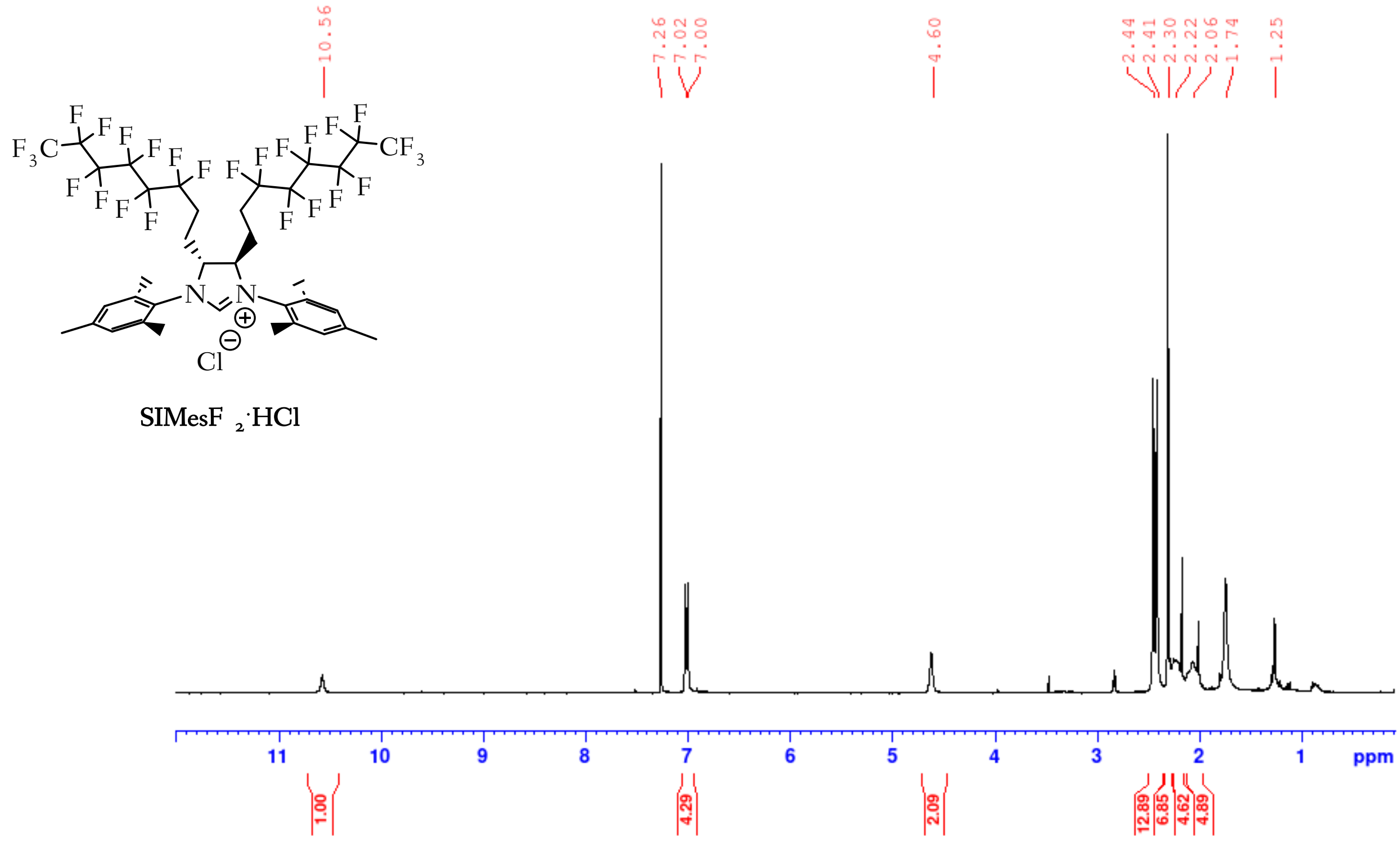

Figure S24. ^{1}H NMR Spectrum 400 MHz, $CDCl_3$, **5a**.

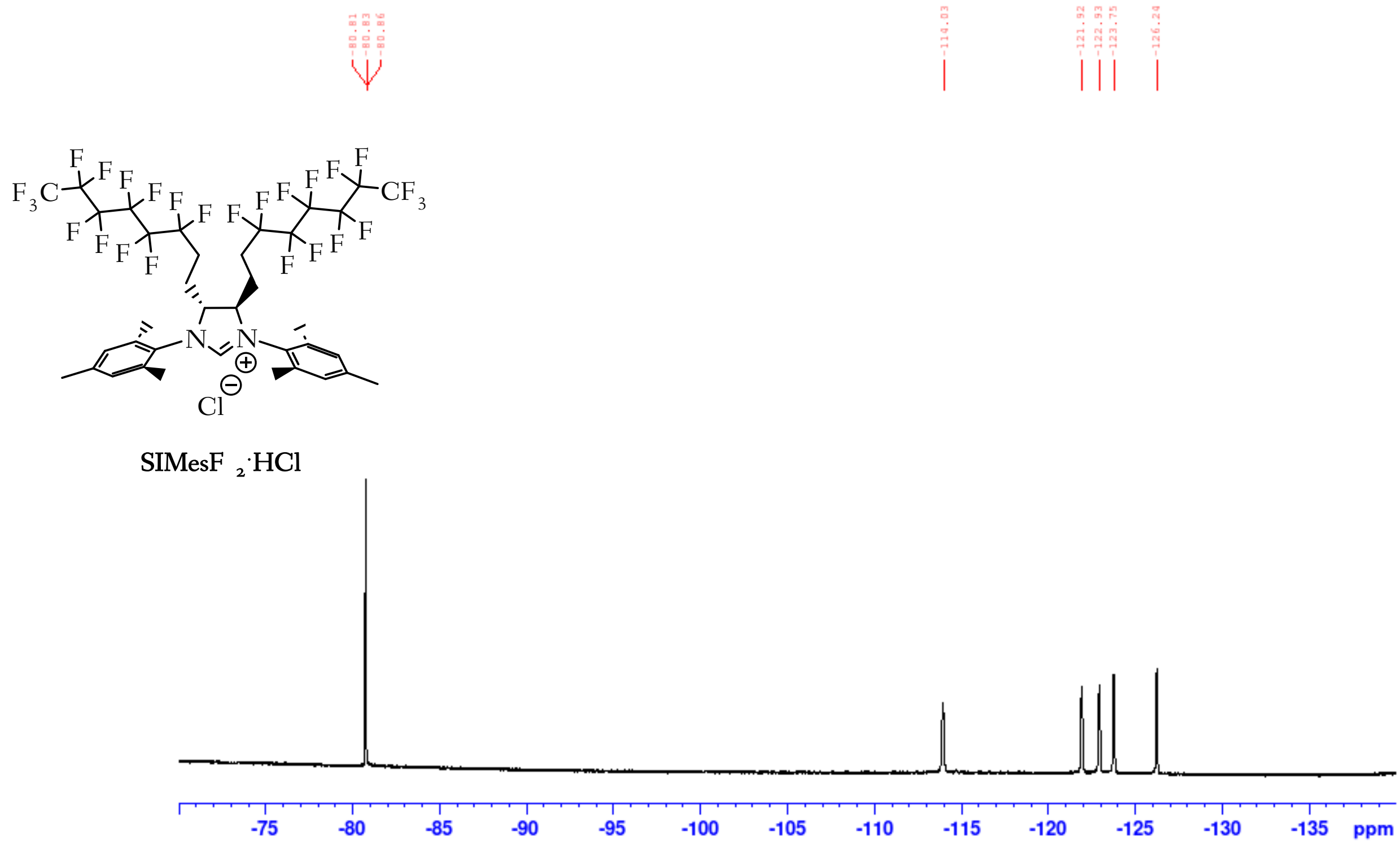

Figure S25. ^{19}F NMR Spectrum 376 MHz, $CDCl_3$, **SIMesF$_2$·HCl**.

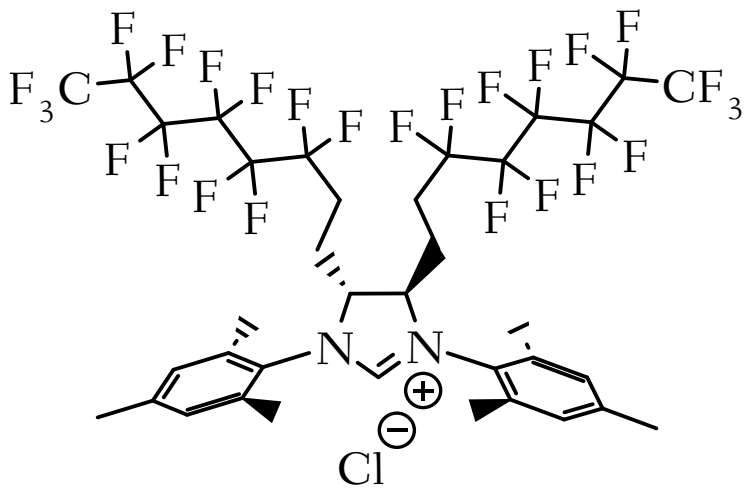

SIMesF$_2$·HCl

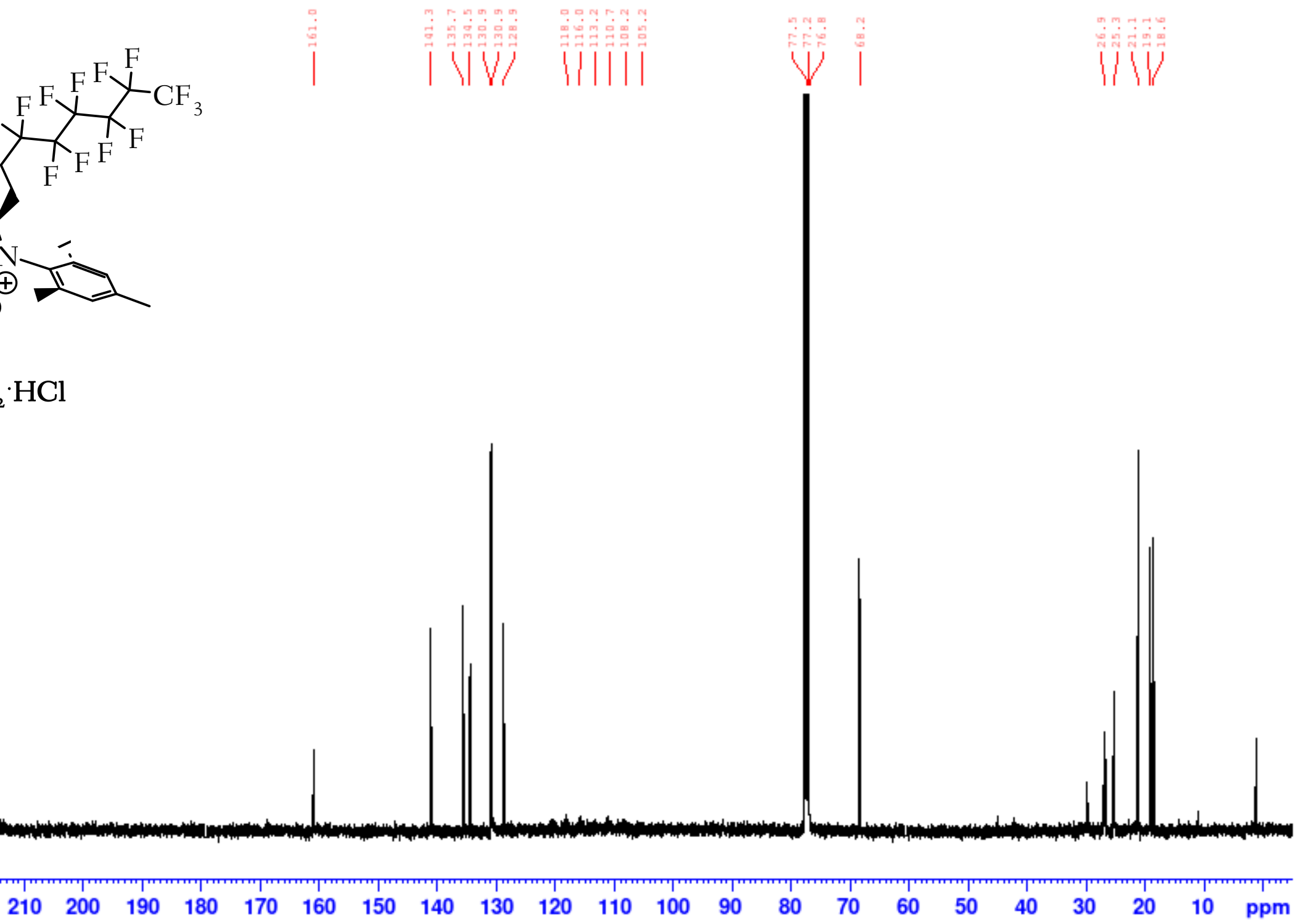

Figure S26. ^{13}C NMR Spectrum 100.5 MHz, $CDCl_3$, **SIMesF$_2$·HCl**.

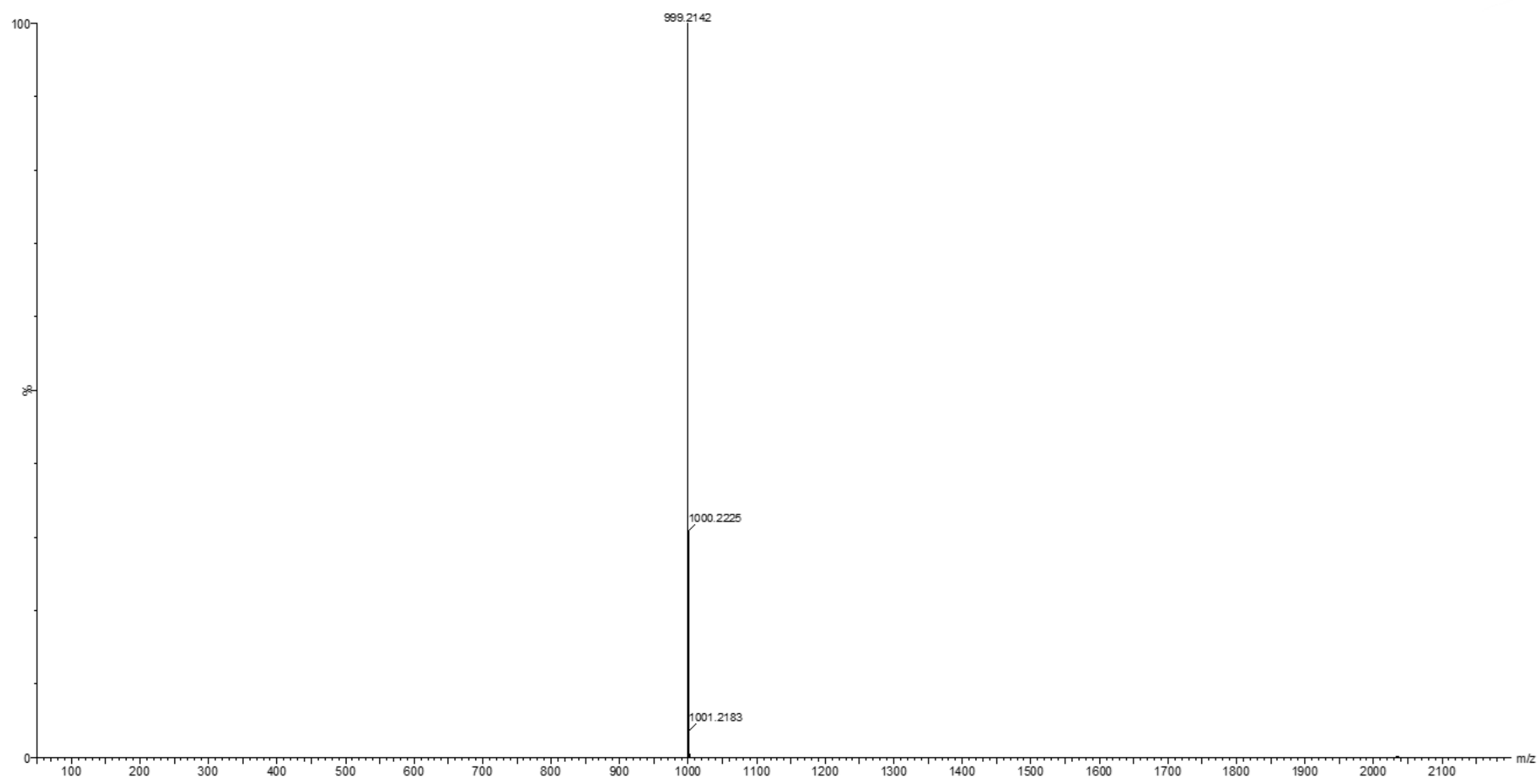

Figure S27. HRMS (ESI+), **SIMesF$_2$·HCl**.

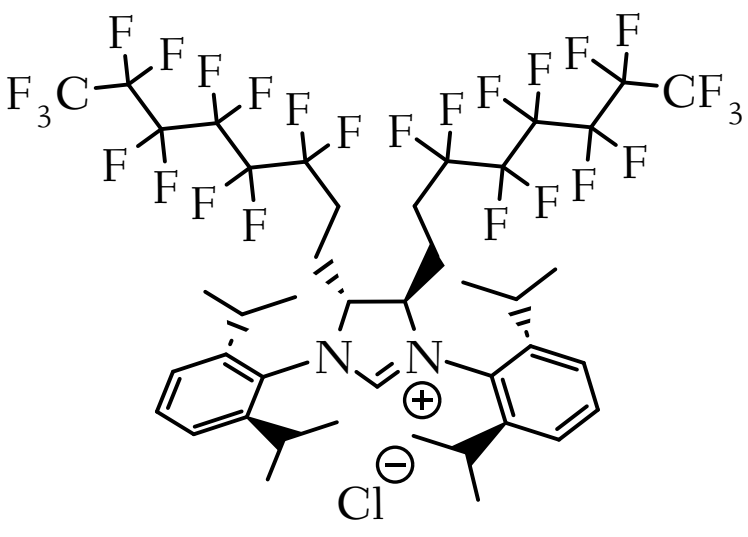

SIPrF $_2$·HCl

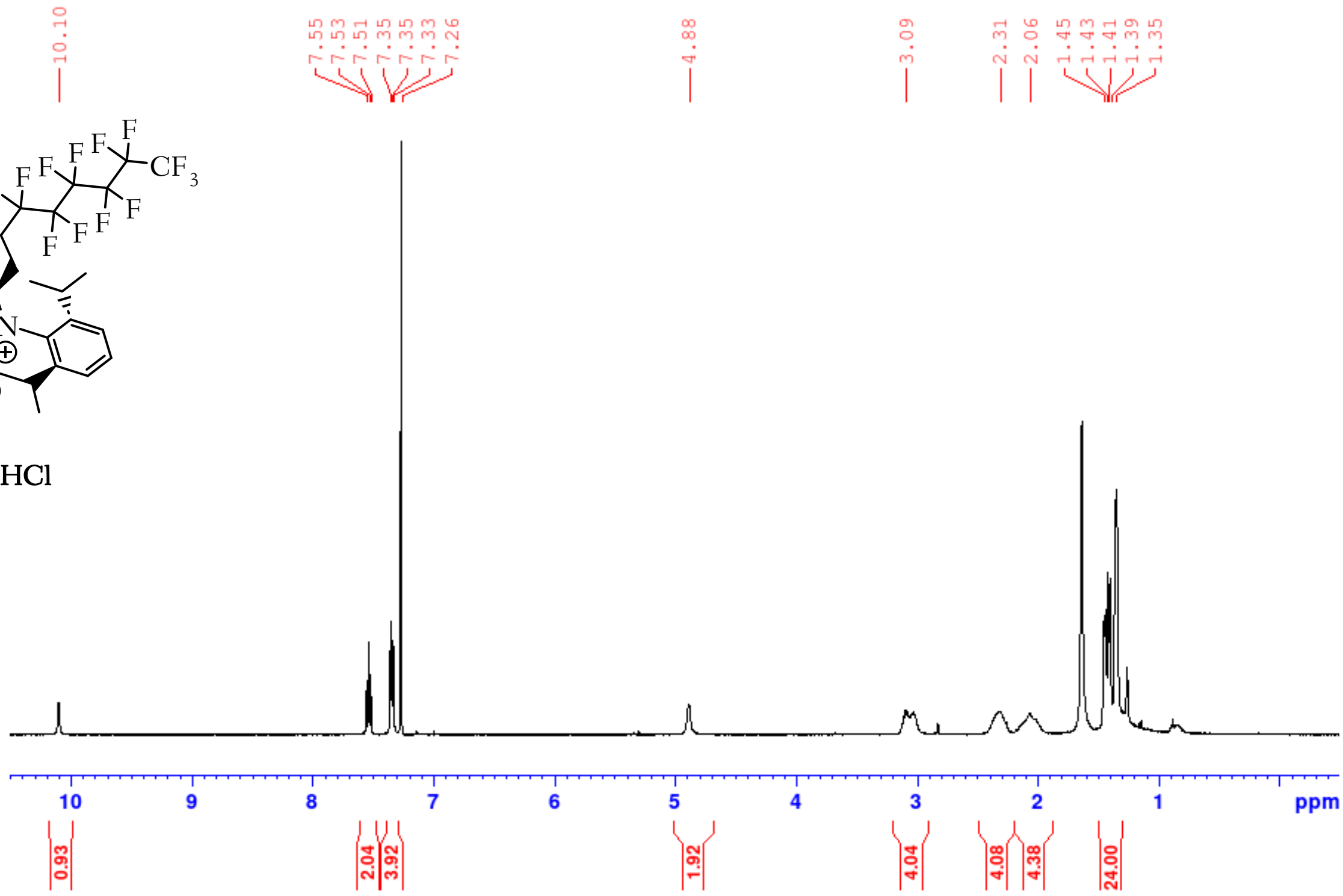

Figure S28. ^{1}H NMR Spectrum 400 MHz, $CDCl_3$, **SIPrF$_2$·HCl**.

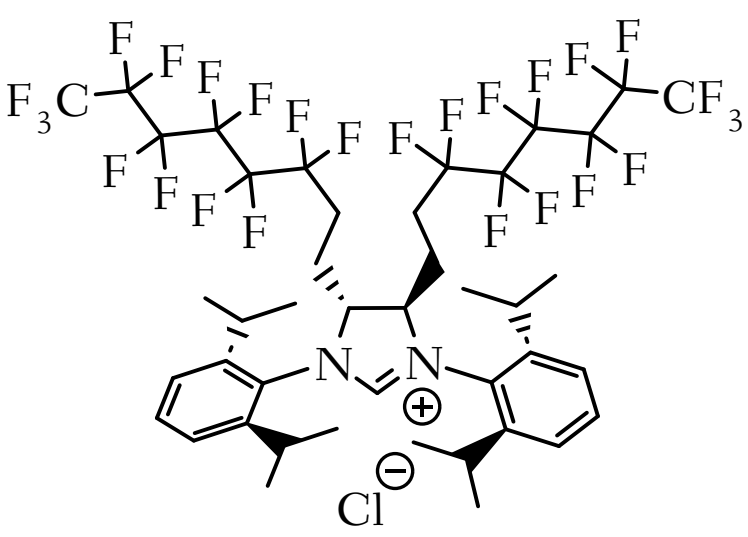

SIPrF $_2$·HCl

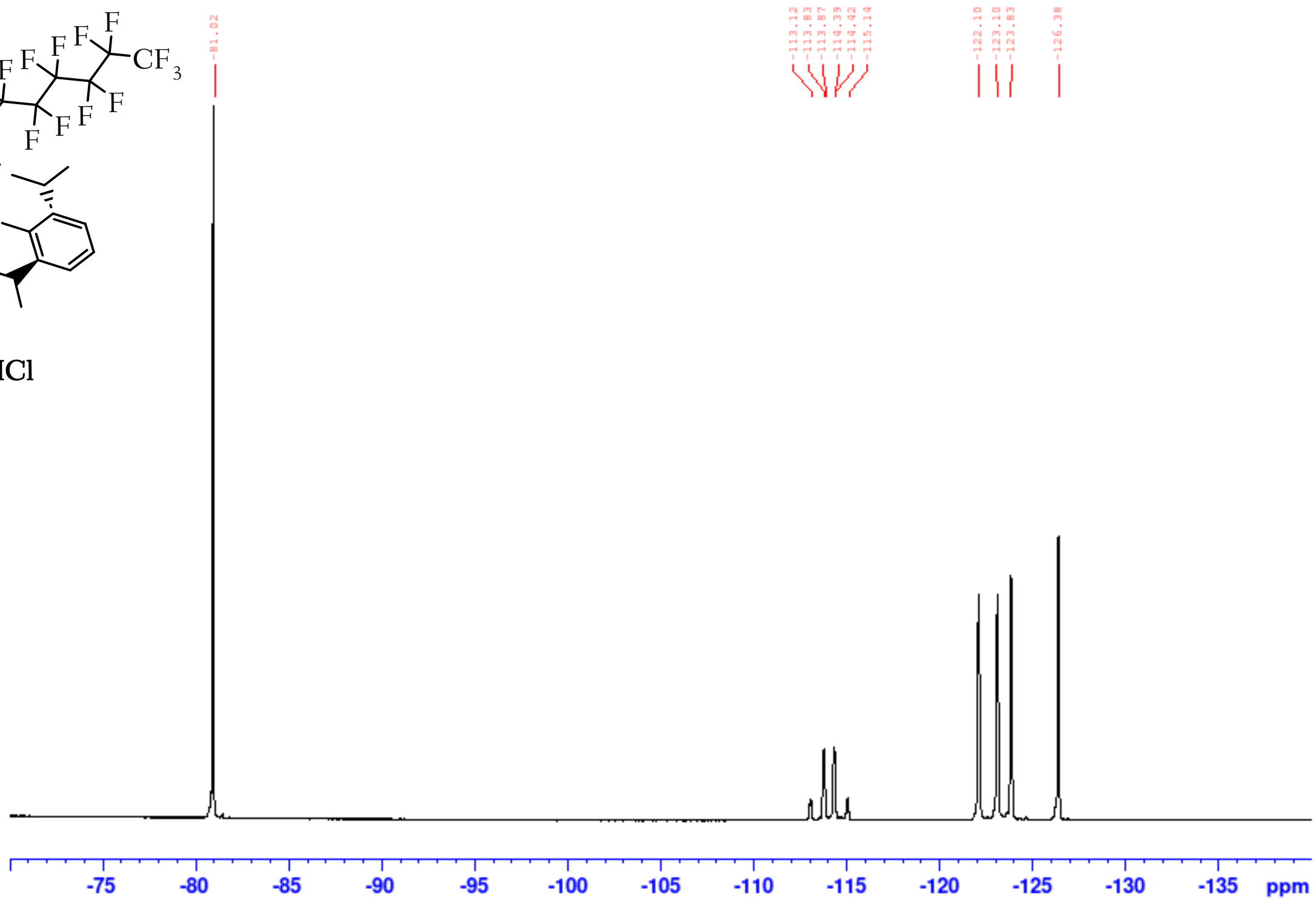

Figure S29. ^{19}F NMR Spectrum 376 MHz, $CDCl_3$, **SIPrF$_2$·HCl**.

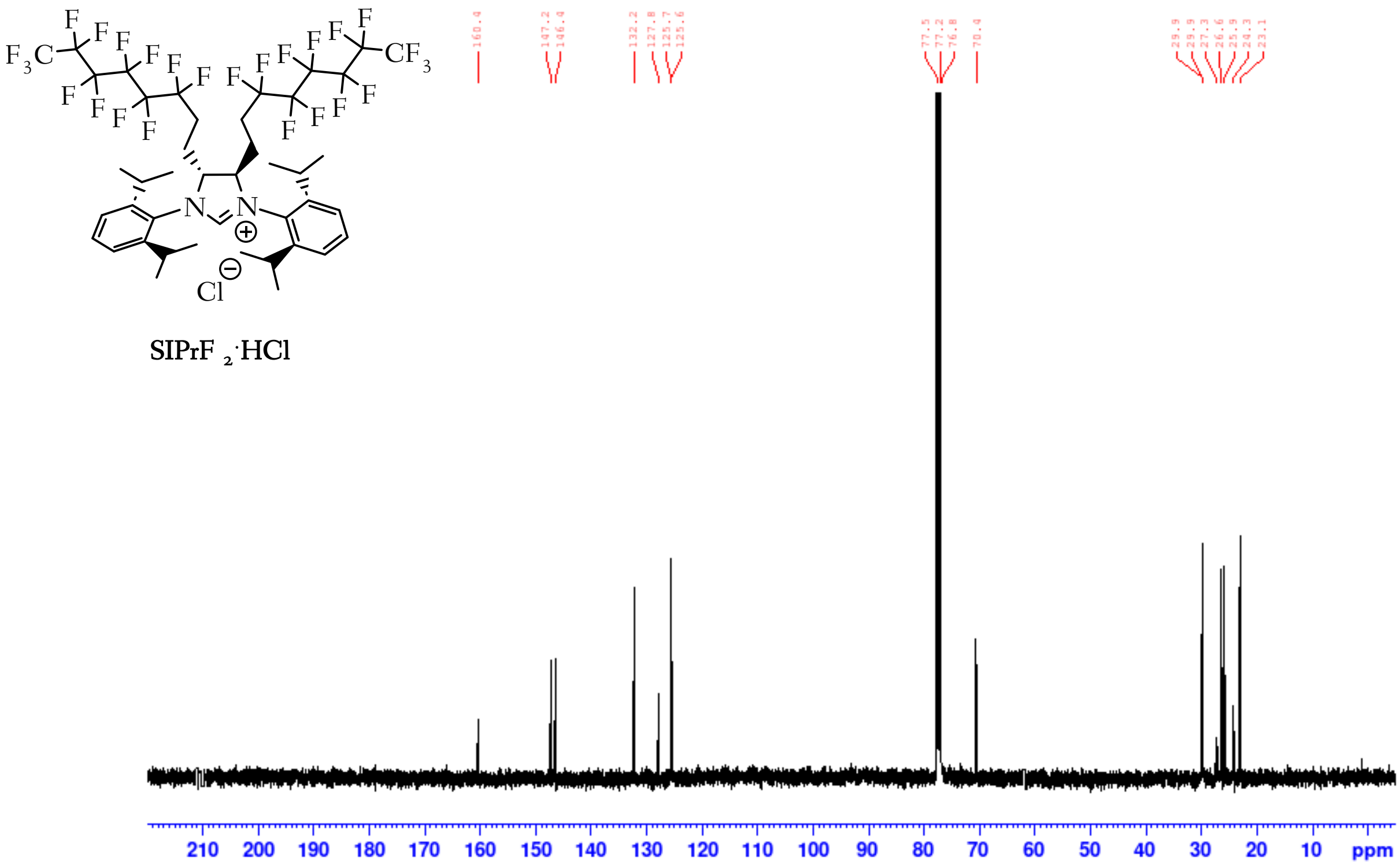

Figure S30. ^{13}C NMR Spectrum 100.5 MHz, $CDCl_3$, **SIPrF$_2$·HCl**.

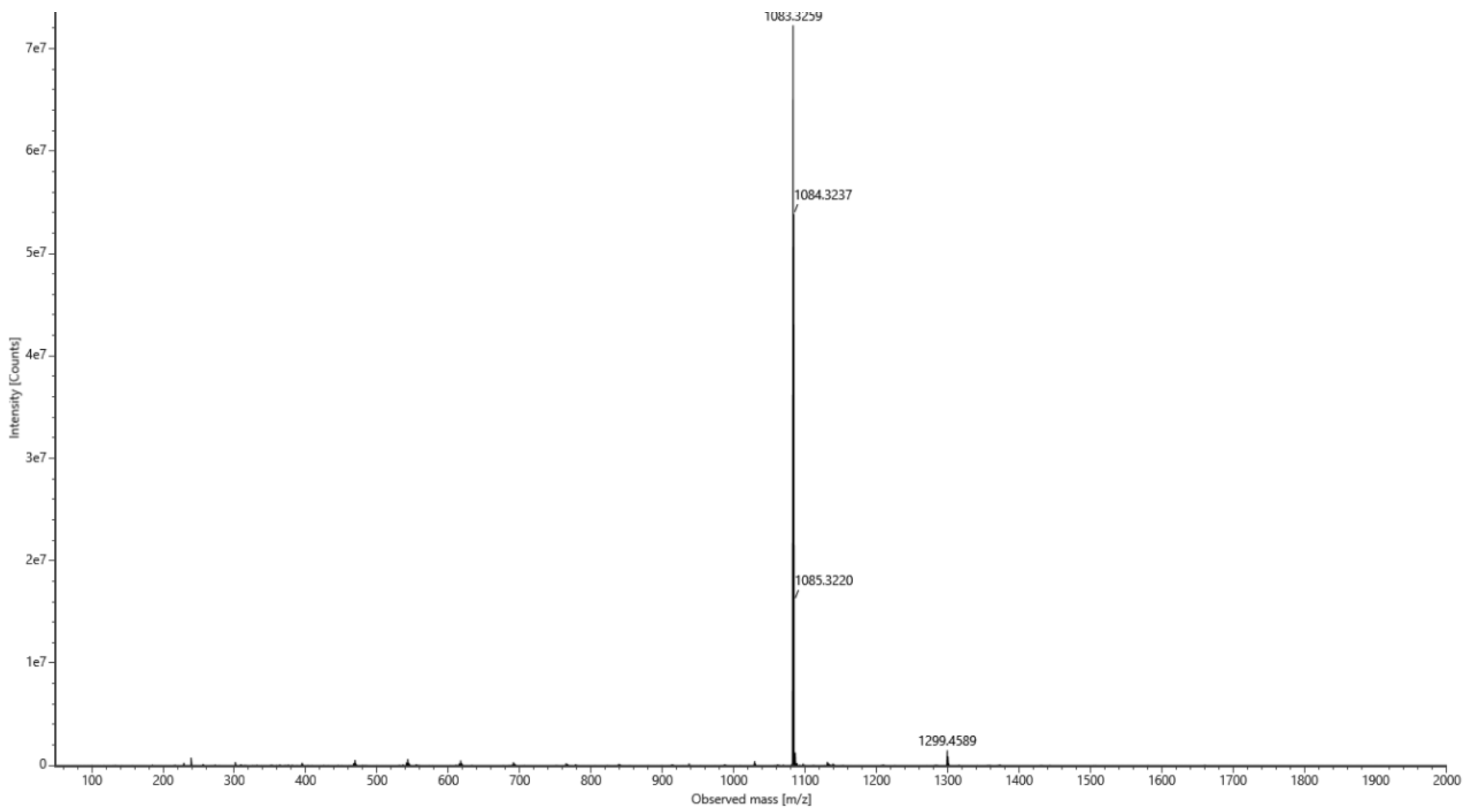

Figure S31. HRMS (ESI+) **SIPrF$_2$·HCl**.

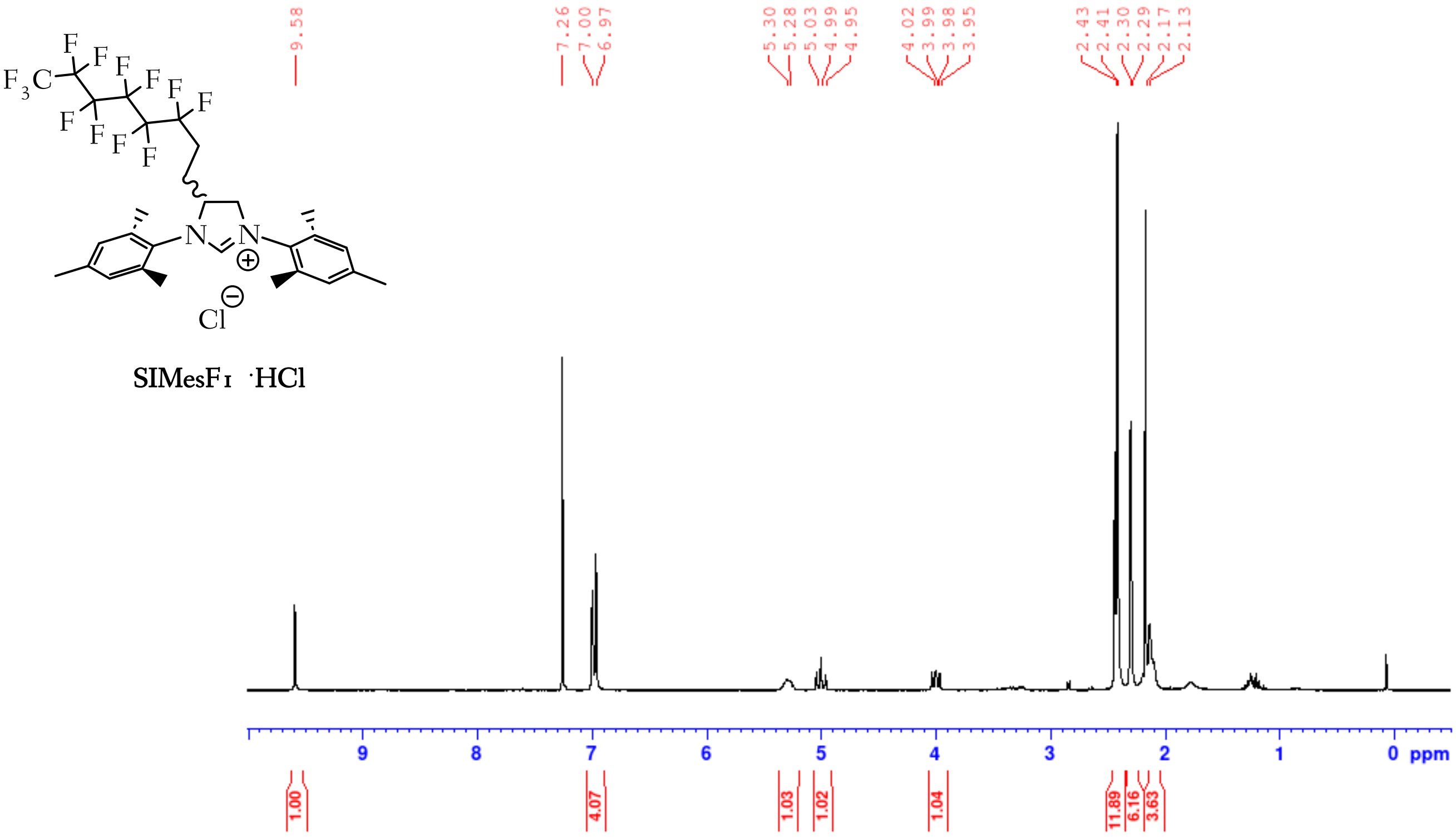

Figure S32. ¹H NMR Spectrum 400 MHz, $CDCl_3$, **SIMesF₁·HCl**.

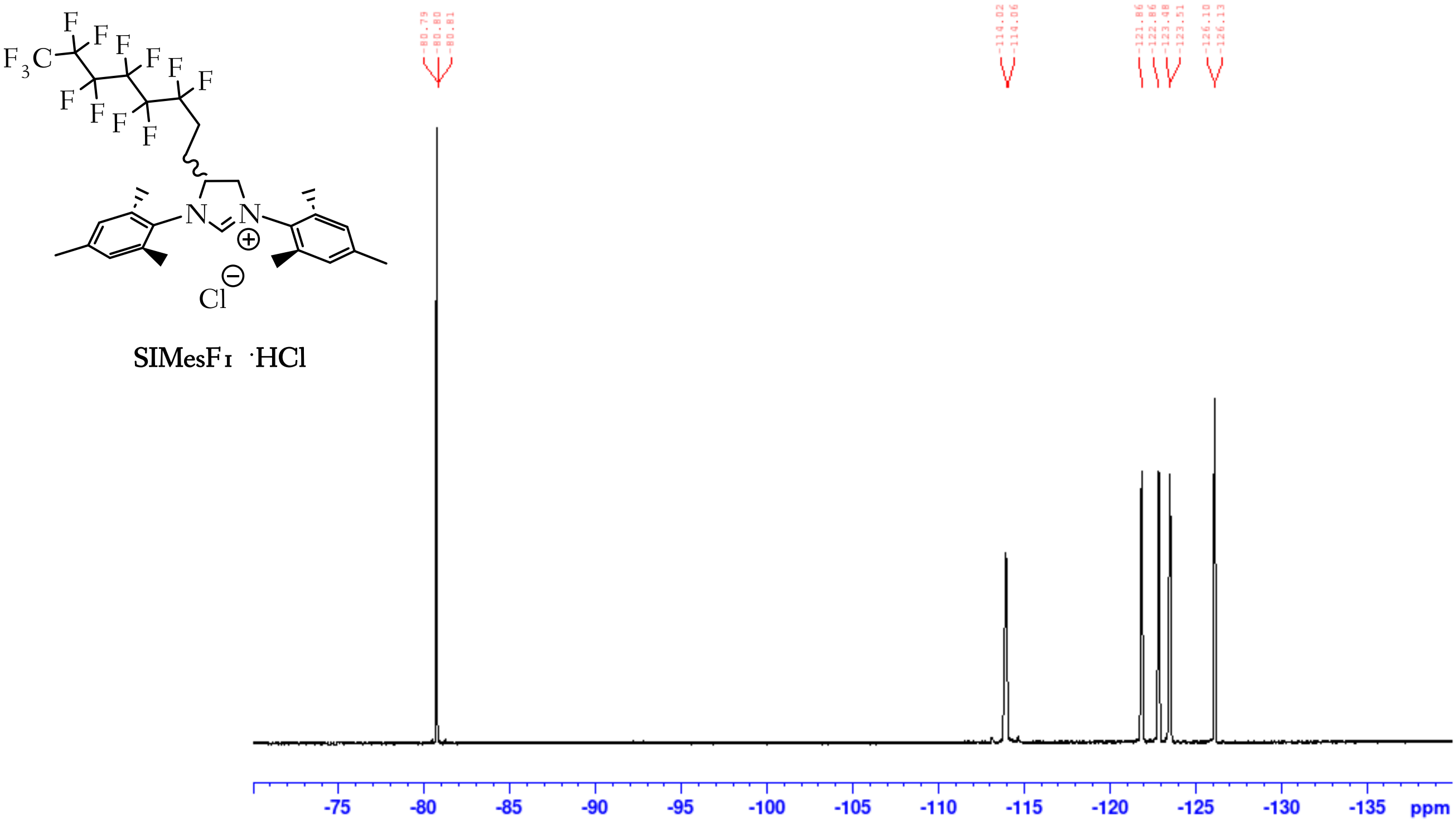

Figure S33. ¹⁹F NMR Spectrum 376 MHz, $CDCl_3$, **SIMesF₁·HCl**.

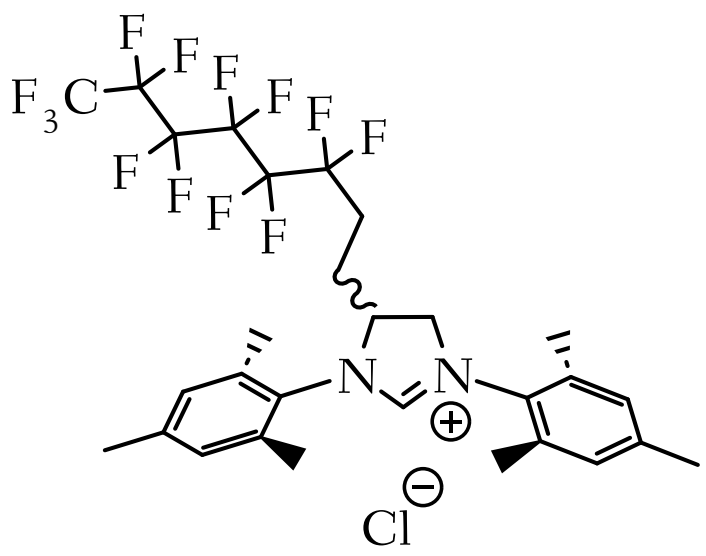

SIMesF1 ·HCl

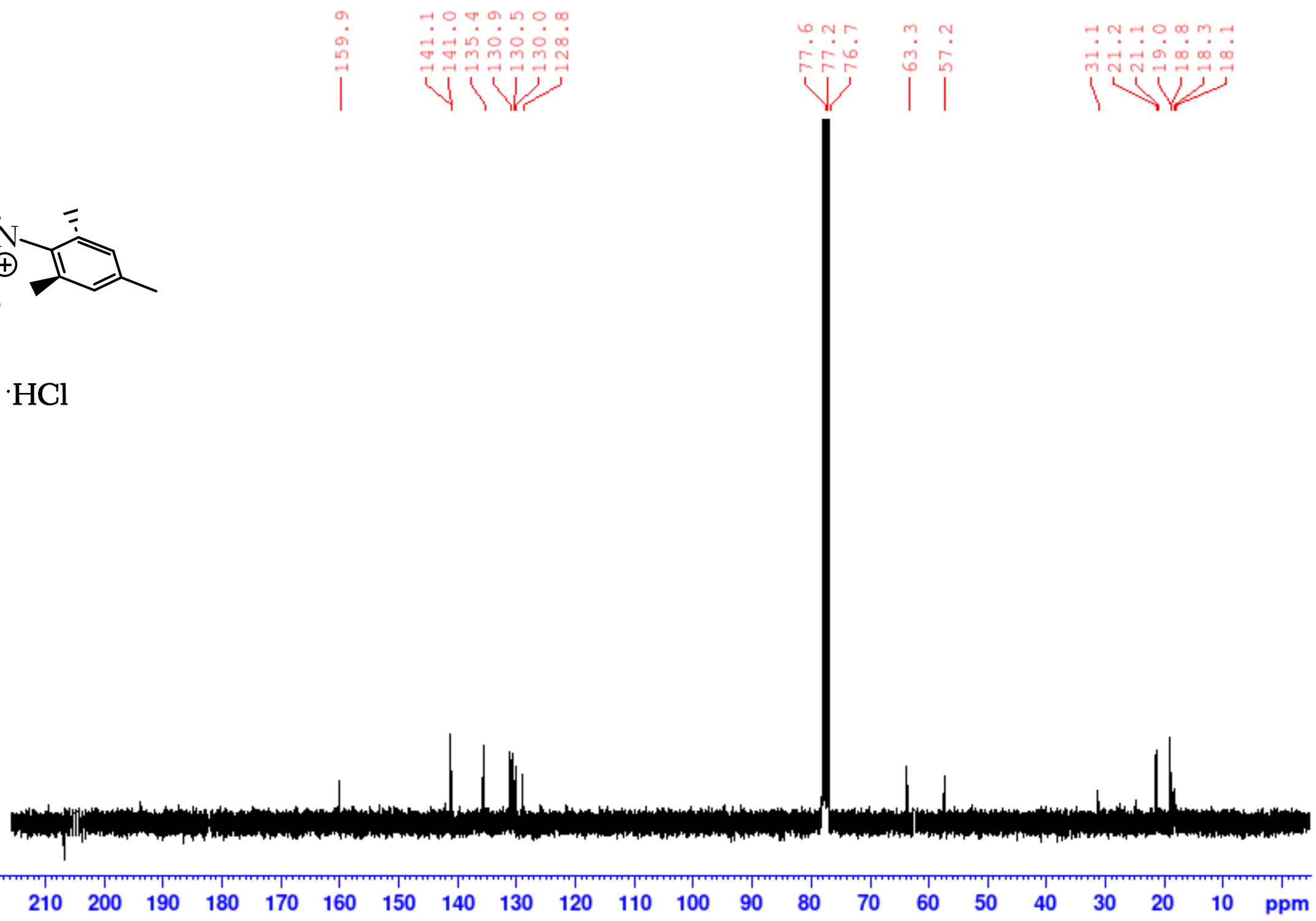

Figure S34. ^{13}C NMR Spectrum 100.5 MHz, $CDCl_3$, **SIMesF$_1$·HCl.**

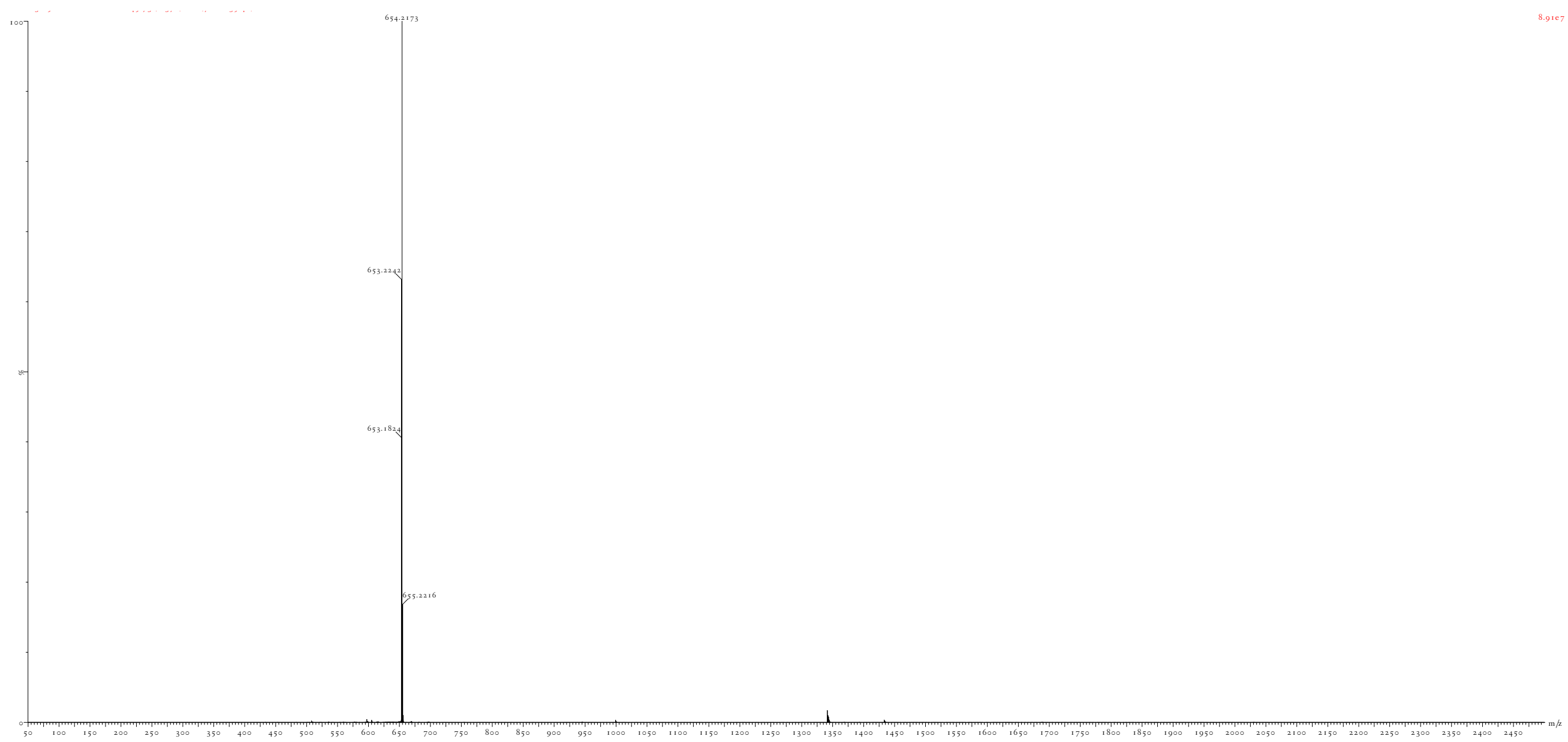

Figure S35. HRMS (ESI+) **SIMesF$_1$·HCl.**

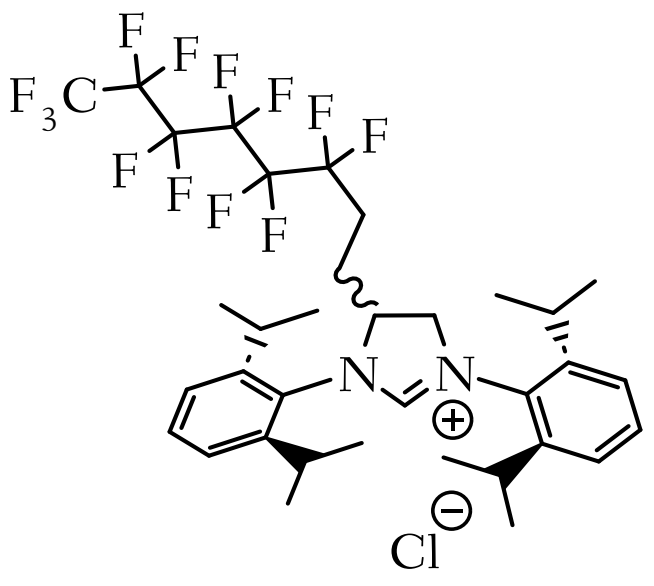

SIPrF₁·HCl

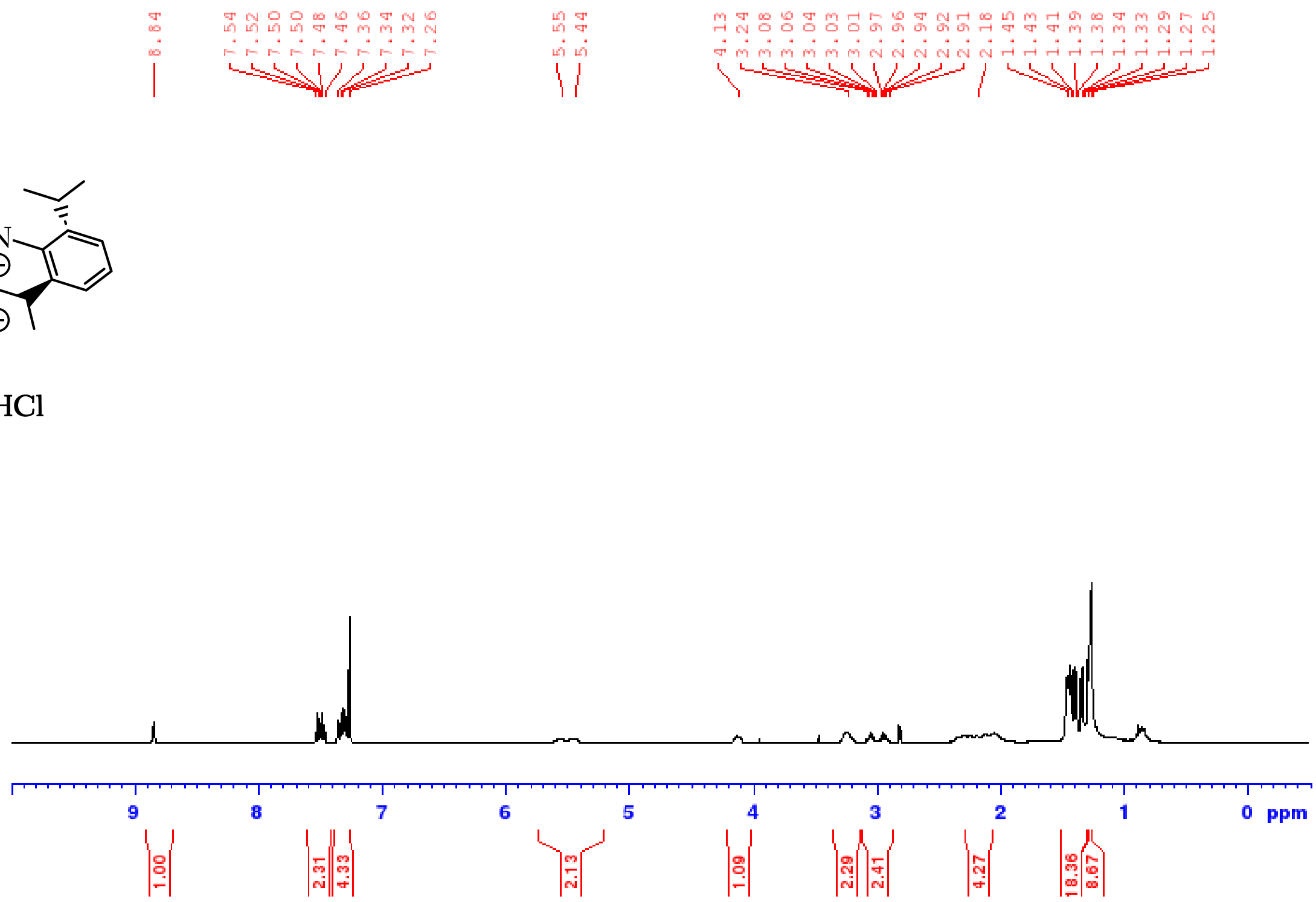

Figure S36. ^{1}H NMR Spectrum 400 MHz, $CDCl_3$, **SIPrF₁·HCl**.

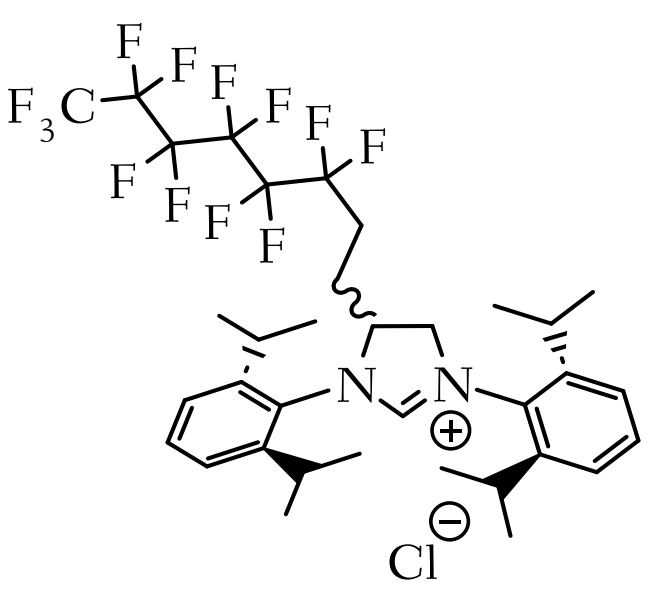

SIPrF₁·HCl

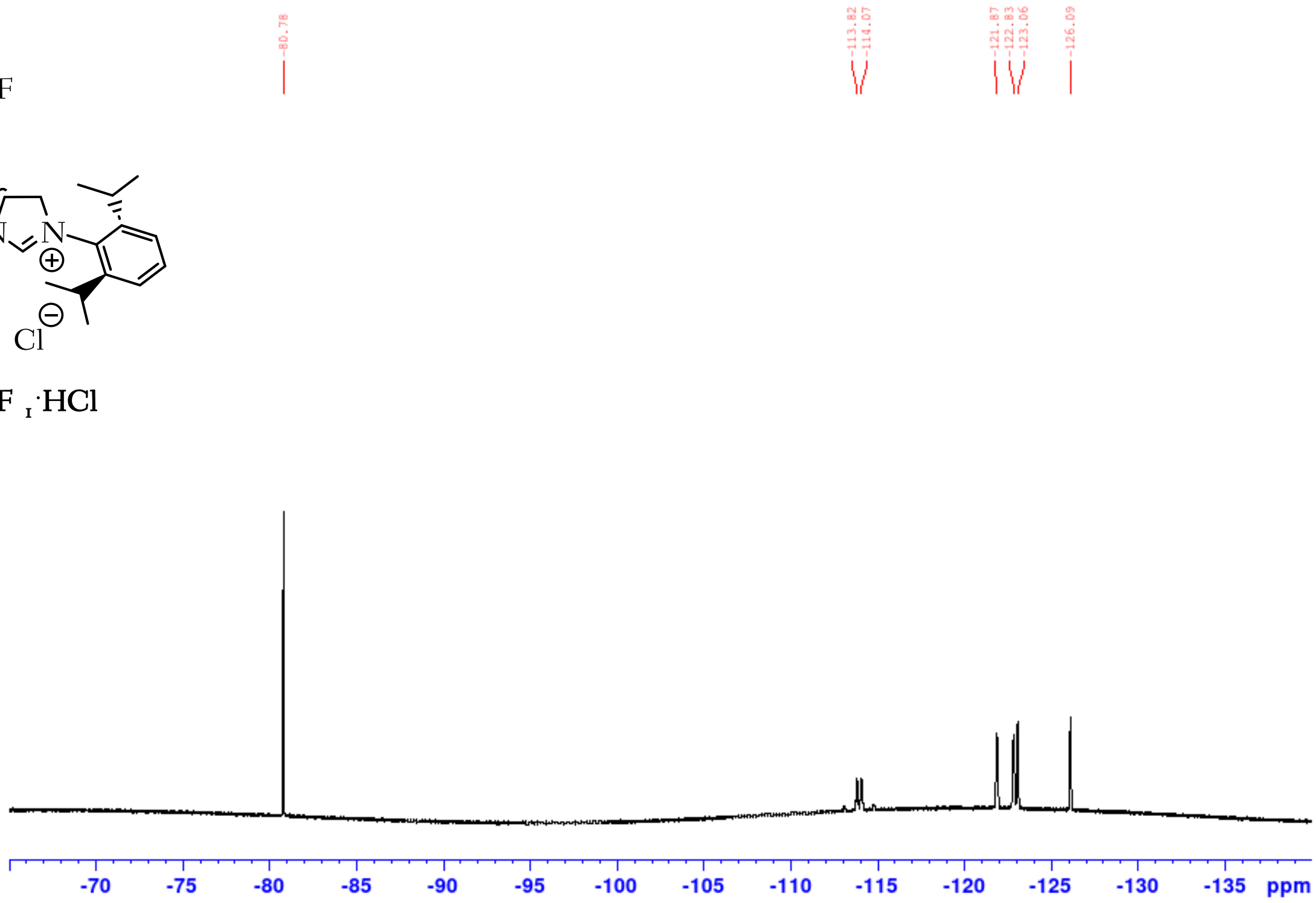

Figure S37. ^{19}F NMR Spectrum 376 MHz, $CDCl_3$, **SIPrF₁·HCl**.

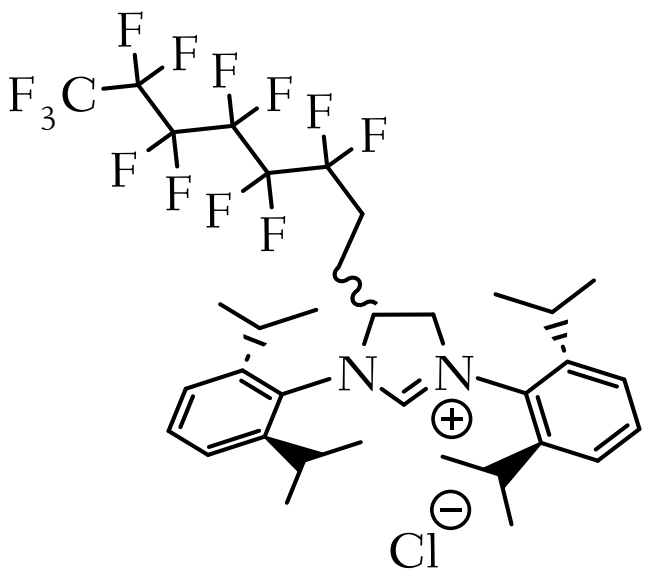

SIPrF$_1$·HCl

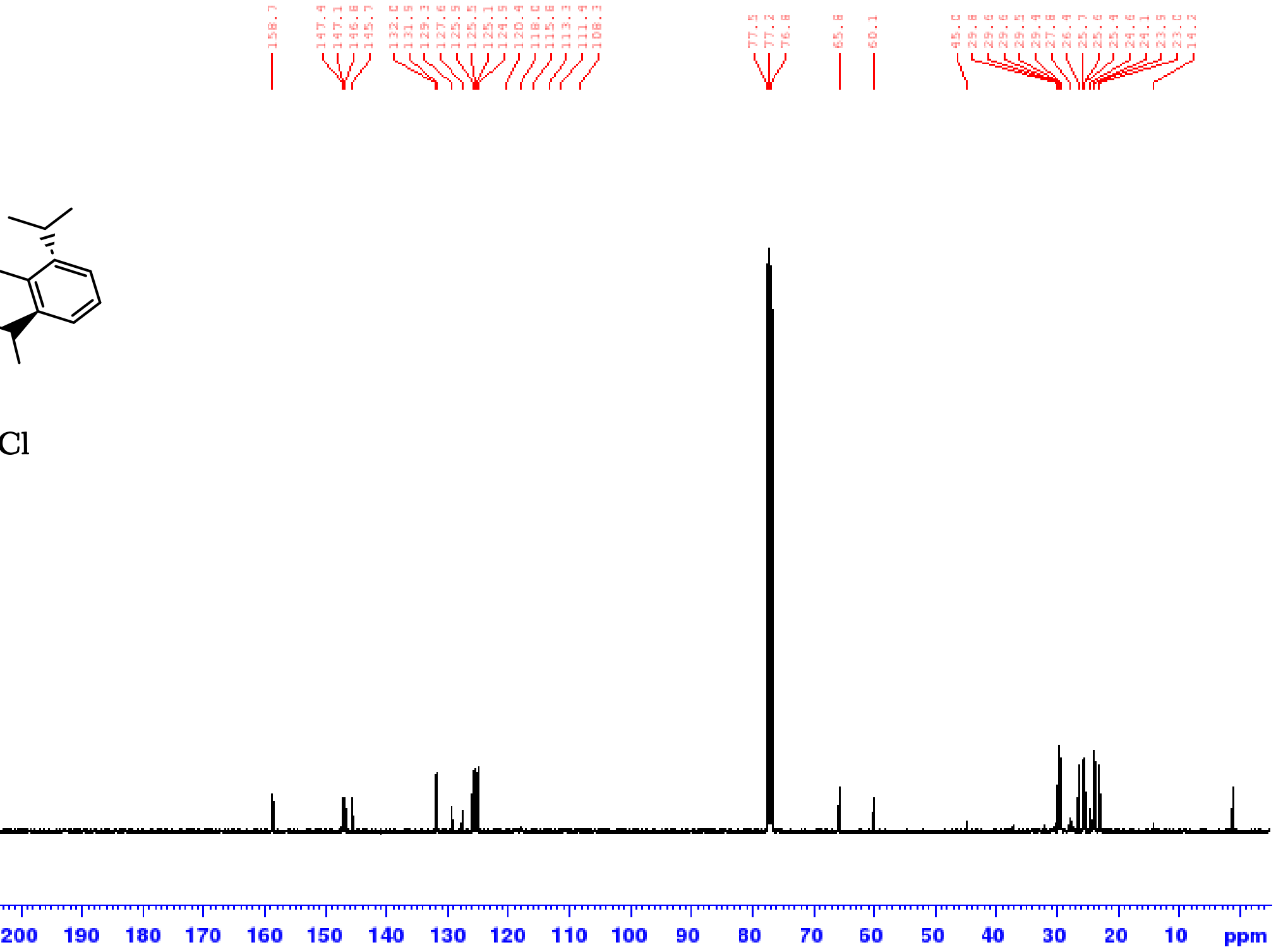

Figure S38. ^{13}C NMR Spectrum 100.5 MHz, $CDCl_3$, **SIPrF$_1$·HCl**.

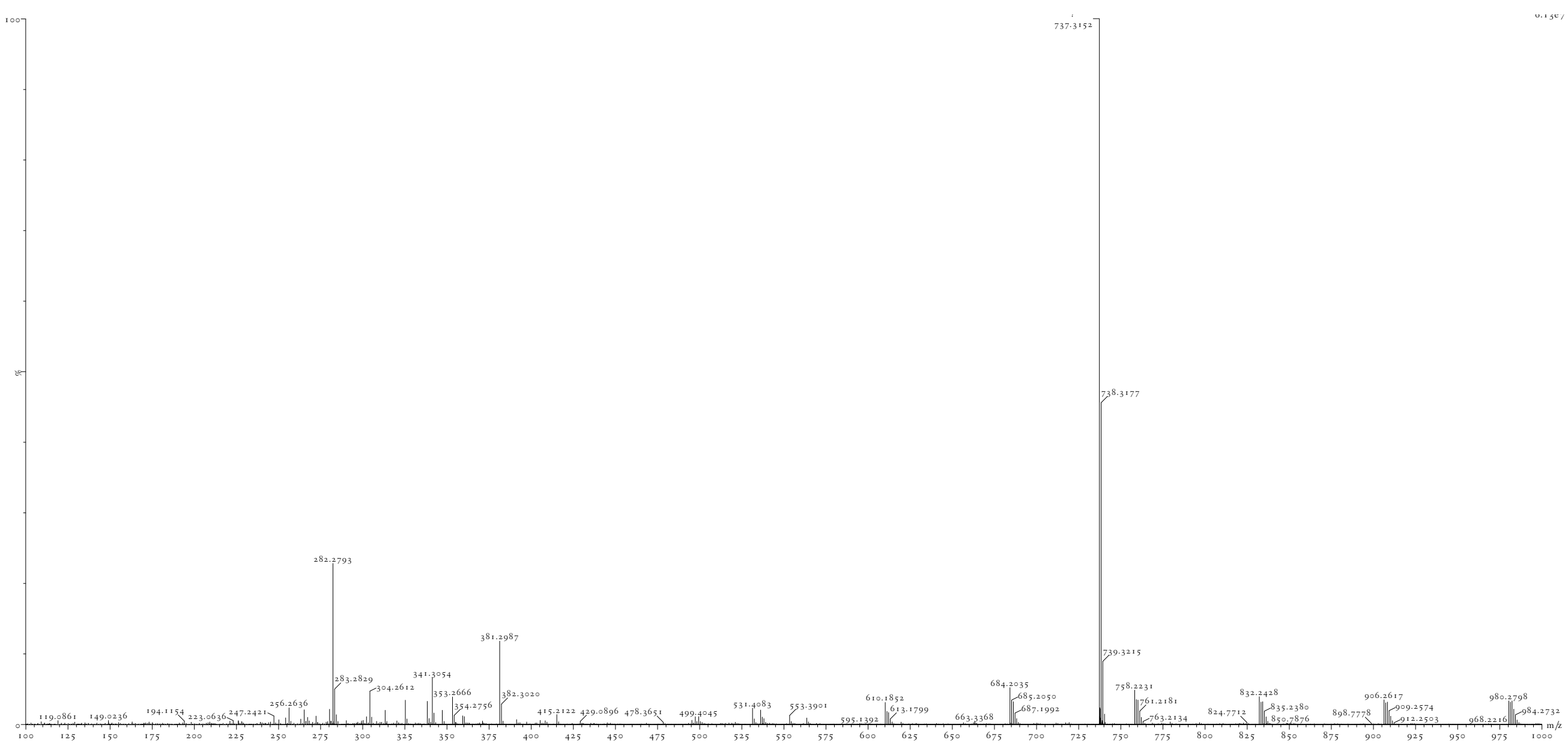

Figure S39. HRMS (ESI+), $CDCl_3$, **SIPrF$_1$·HCl**.

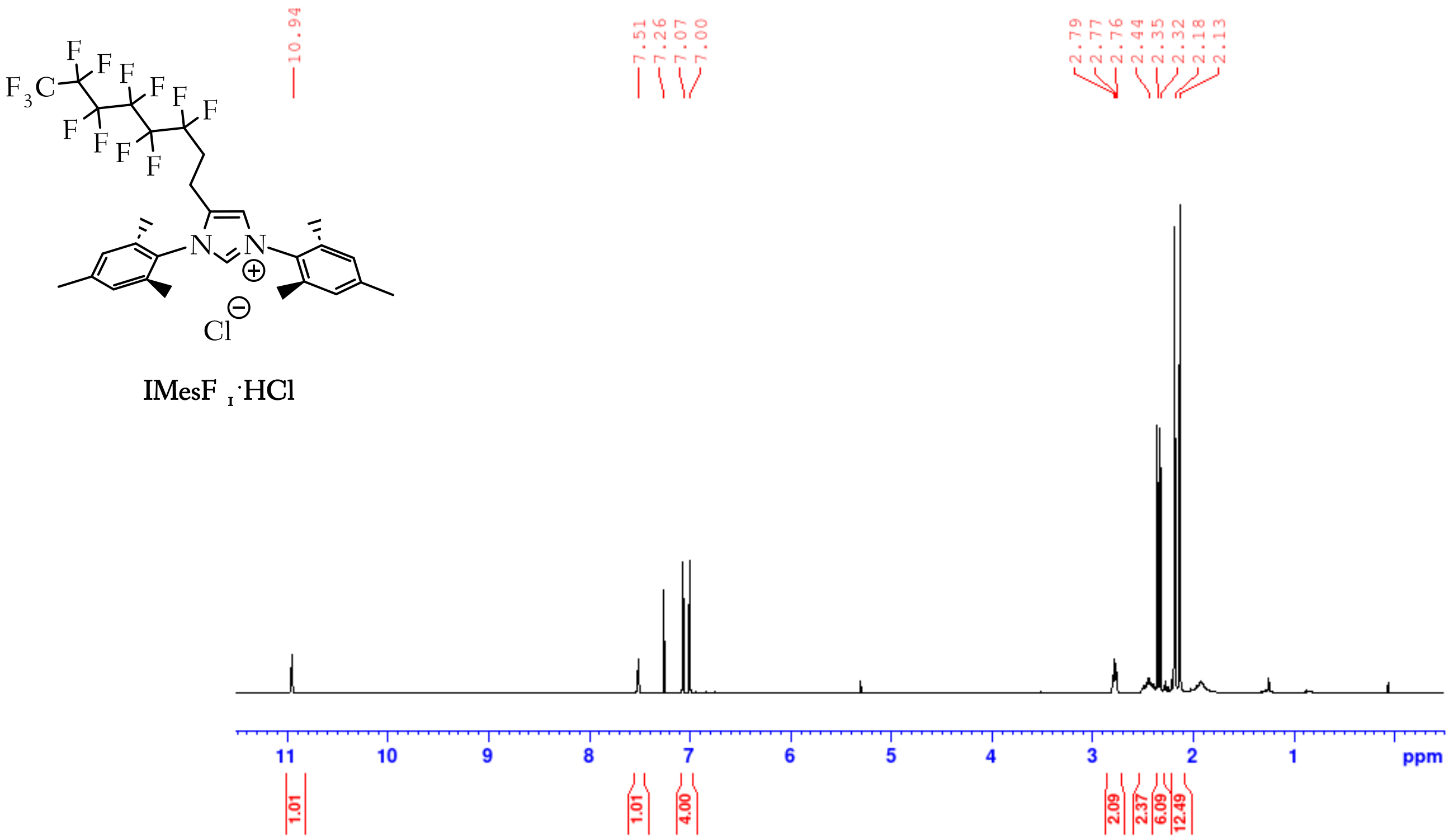

Figure S40. ^{1}H NMR Spectrum 400 MHz, $CDCl_3$, **IMesF$_1$·HCl**.

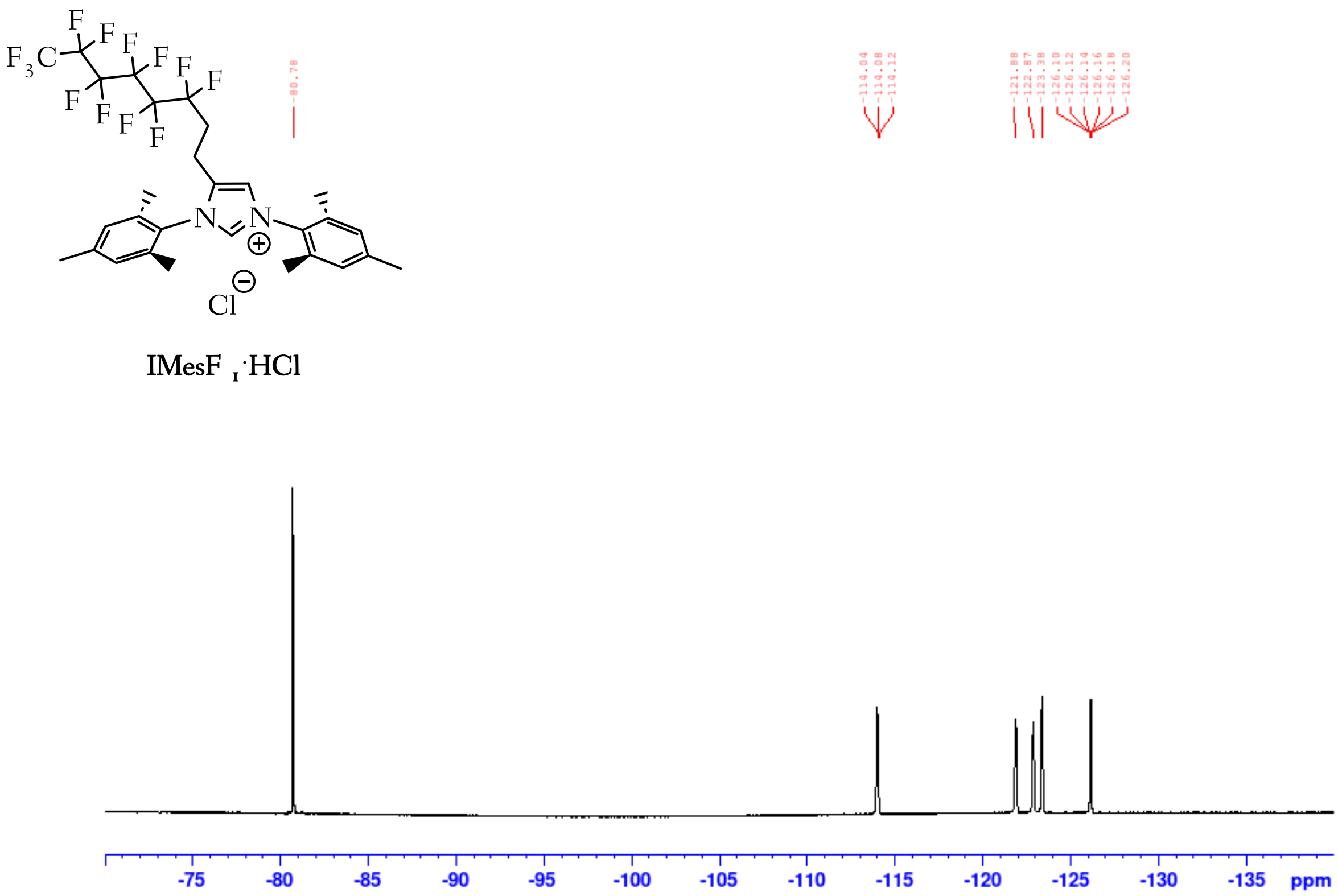

Figure S41. ^{19}F NMR Spectrum 376 MHz, $CDCl_3$, **IMesF$_1$·HCl**.

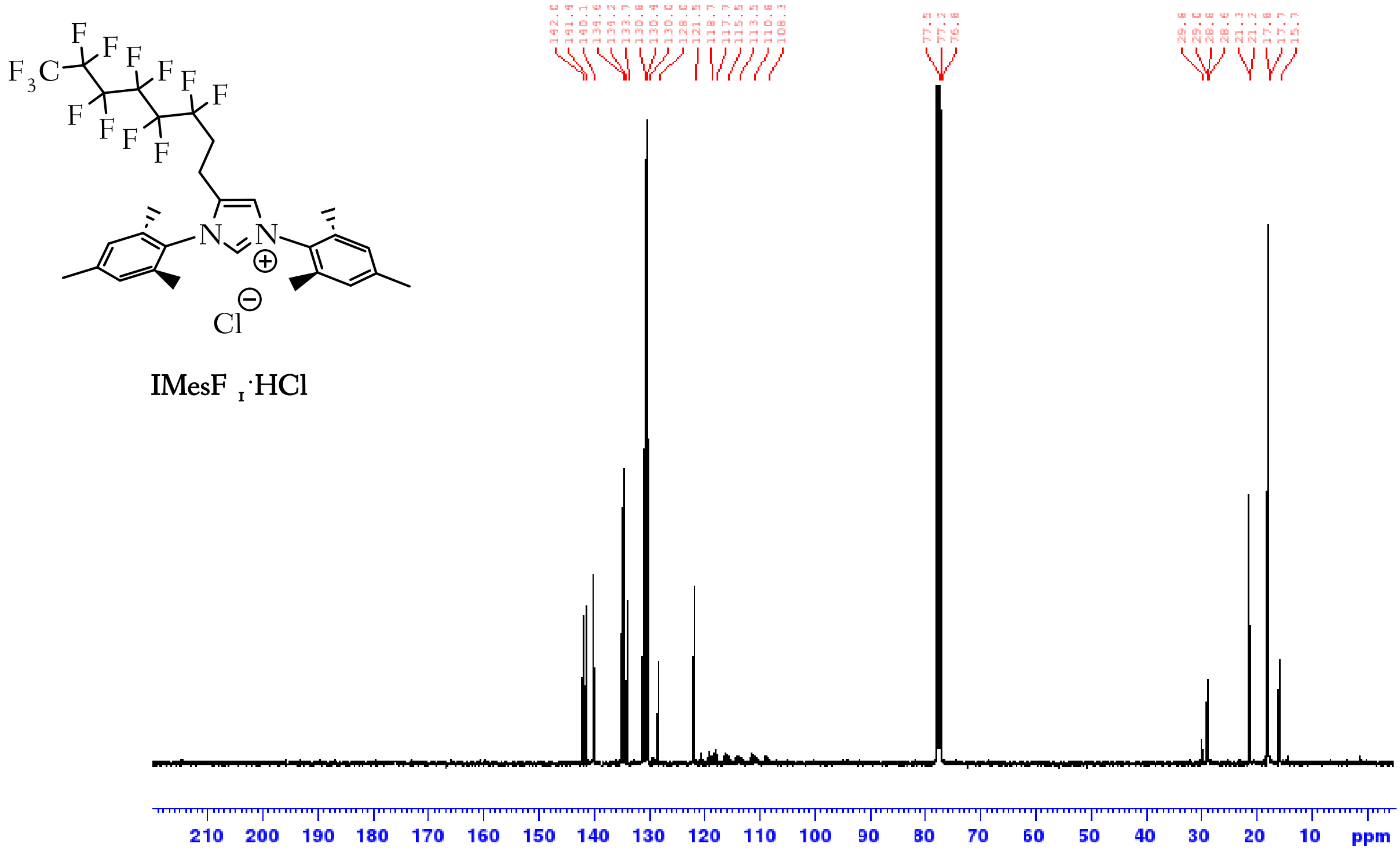

IMesF$_1$·HCl

Figure S42. ^{13}C NMR Spectrum 100.5 MHz, $CDCl_3$, **IMesF$_1$·HCl**.

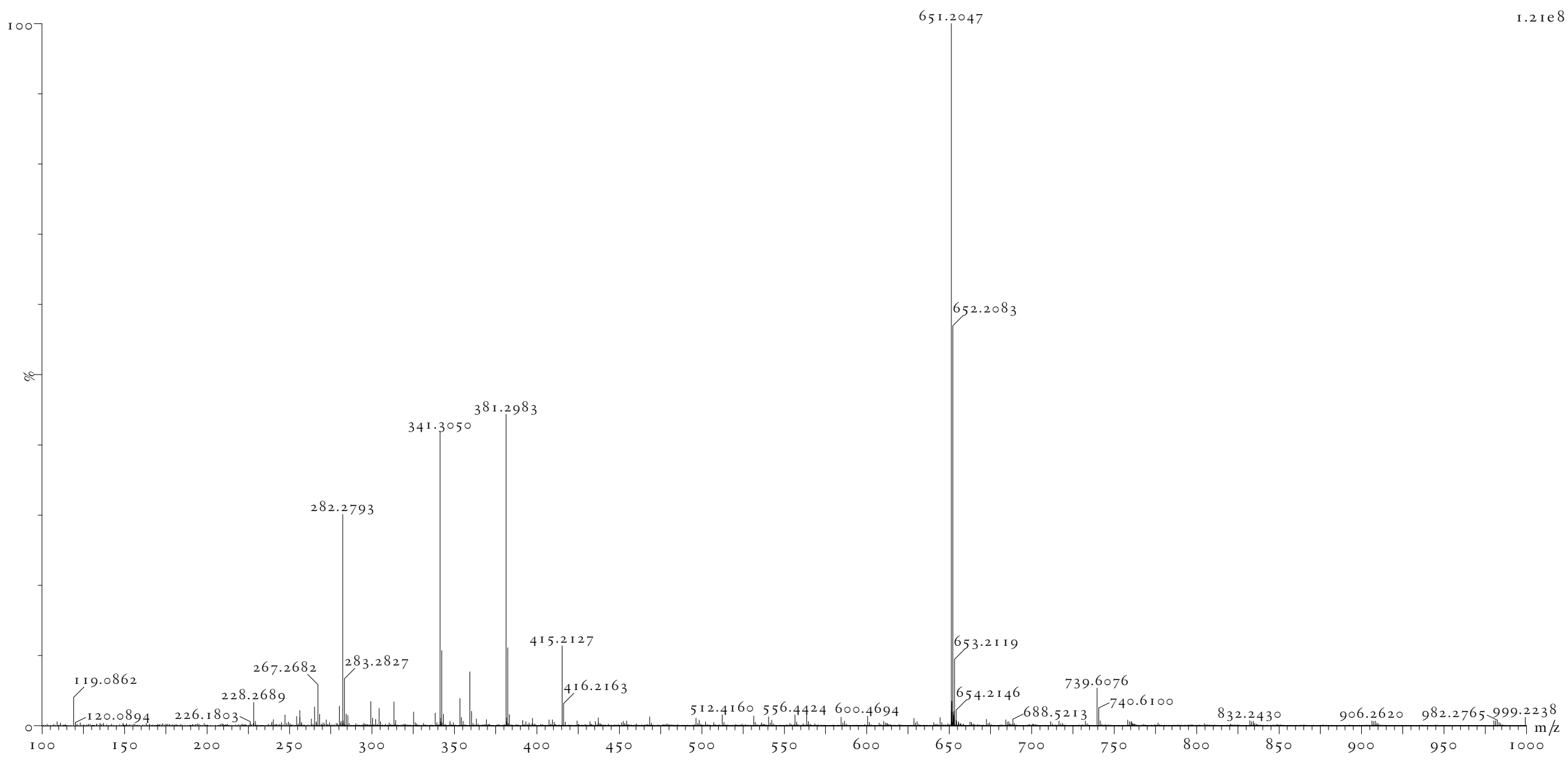

Figure S43. HRMS (ESI+), **IMesF$_1$·HCl**.

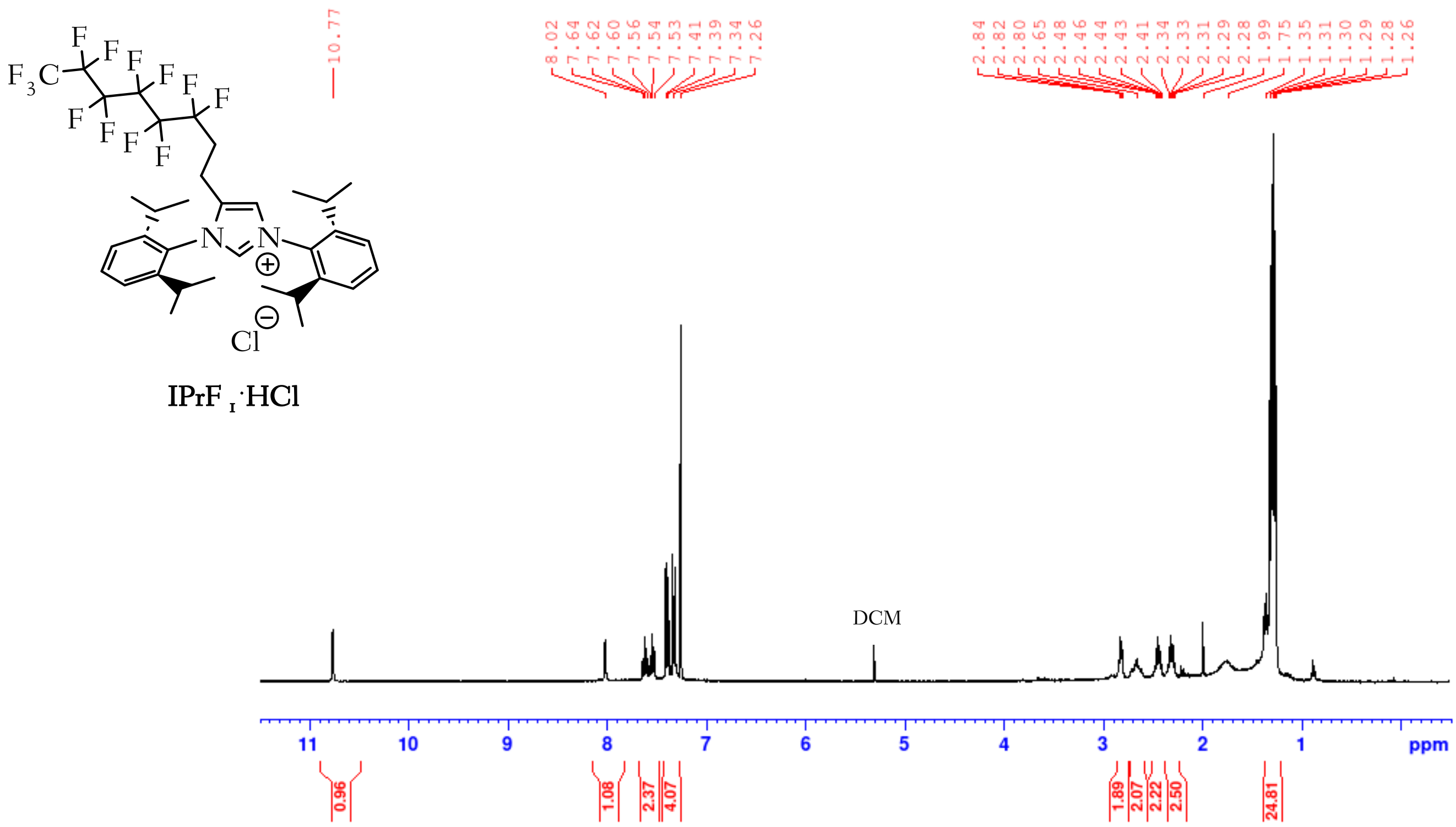

Figure S44. ^{1}H NMR Spectrum 400 MHz, $CDCl_3$, **IPrF$_1$·HCl**.

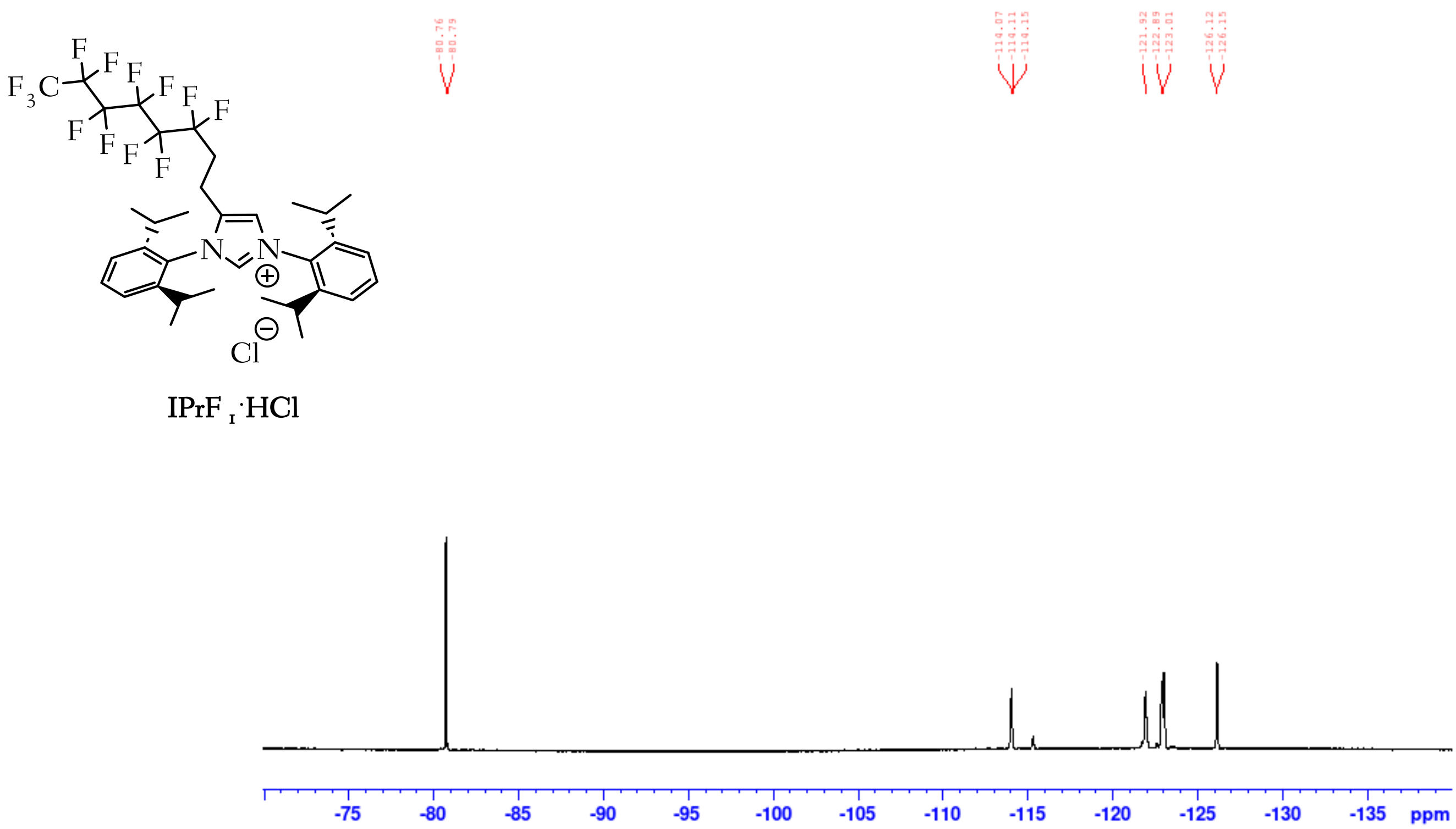

Figure S45. ^{19}F NMR Spectrum 376 MHz, $CDCl_3$, **IPrF$_1$·HCl**.

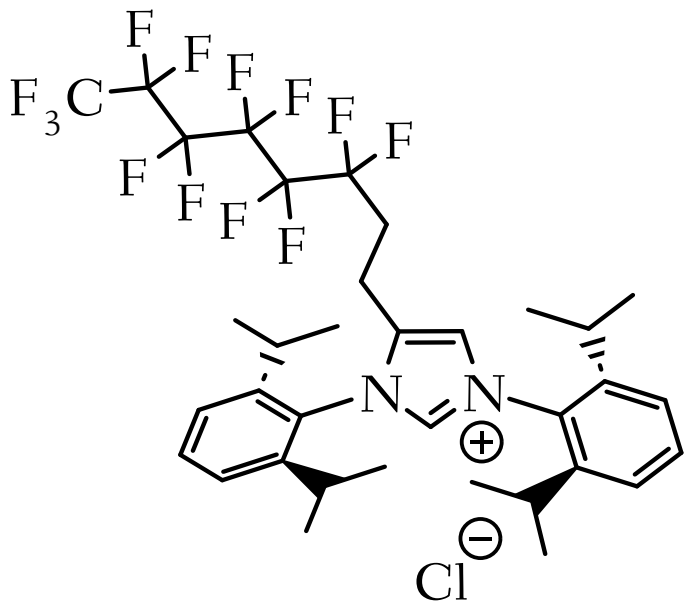

IPrF$_1$·HCl

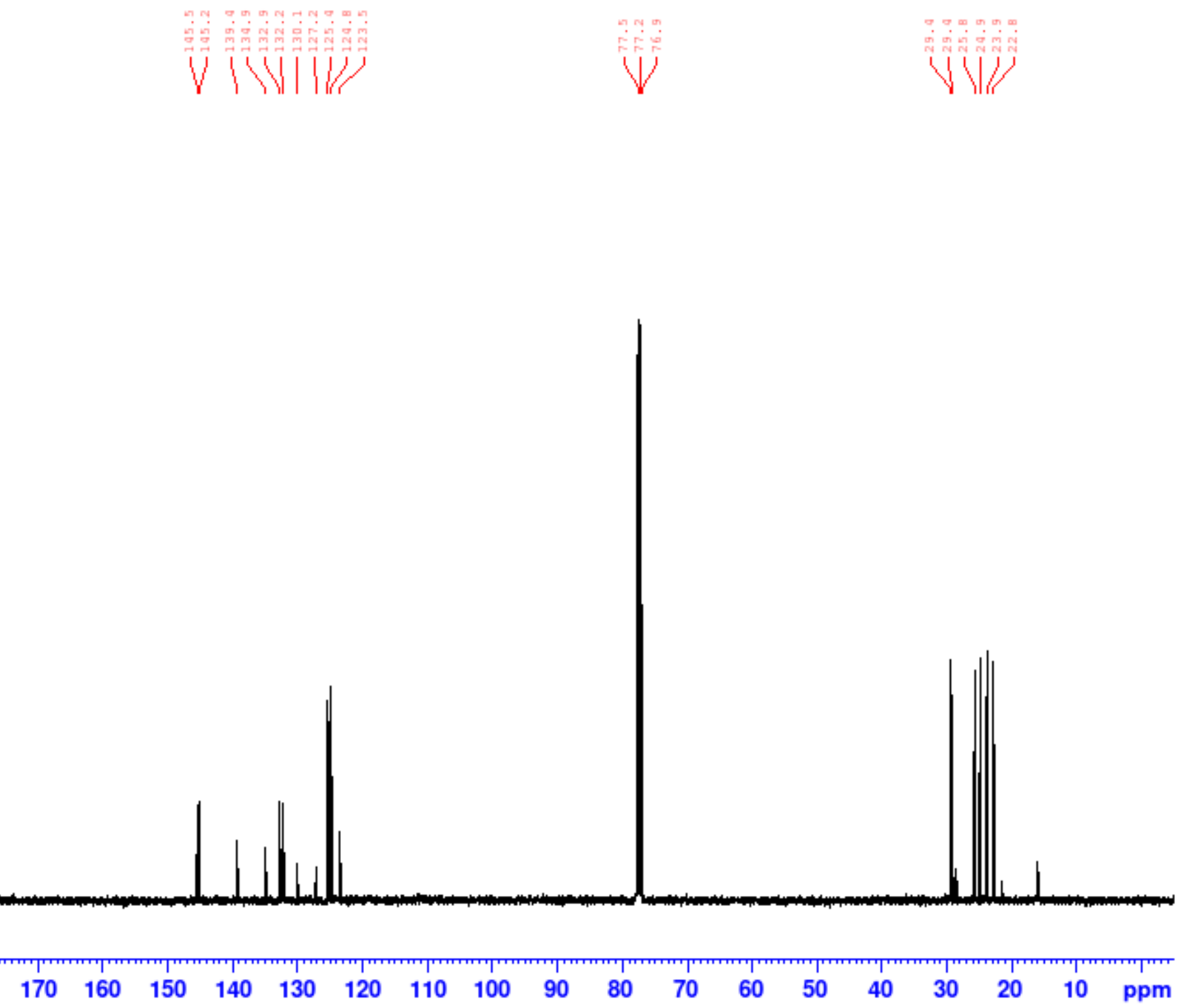

Figure S46. ^{13}C NMR Spectrum 100.5 MHz, $CDCl_3$, **IPrF$_1$·HCl**.

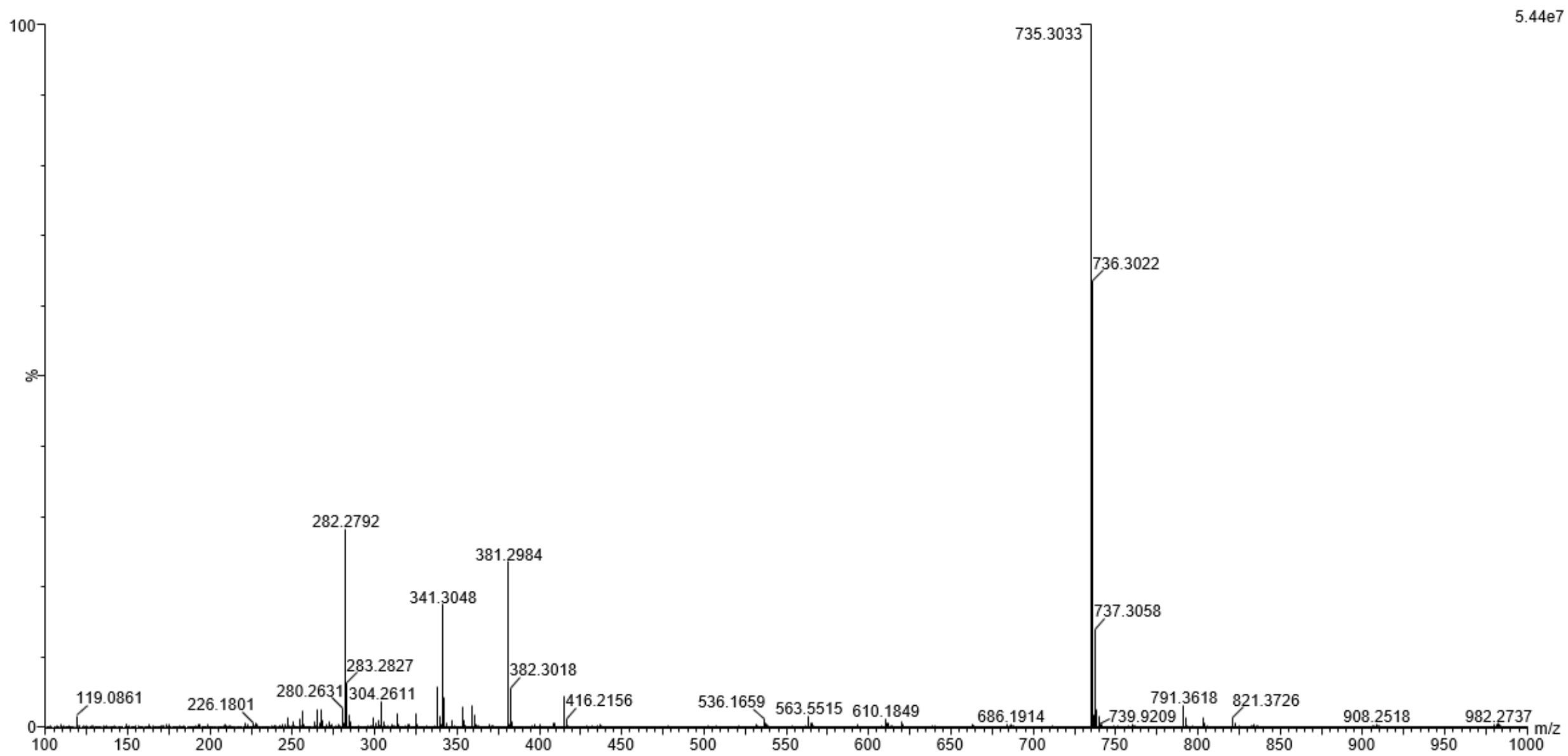

Figure S47. HRMS (ESI+), **IPrF$_1$·HCl**.

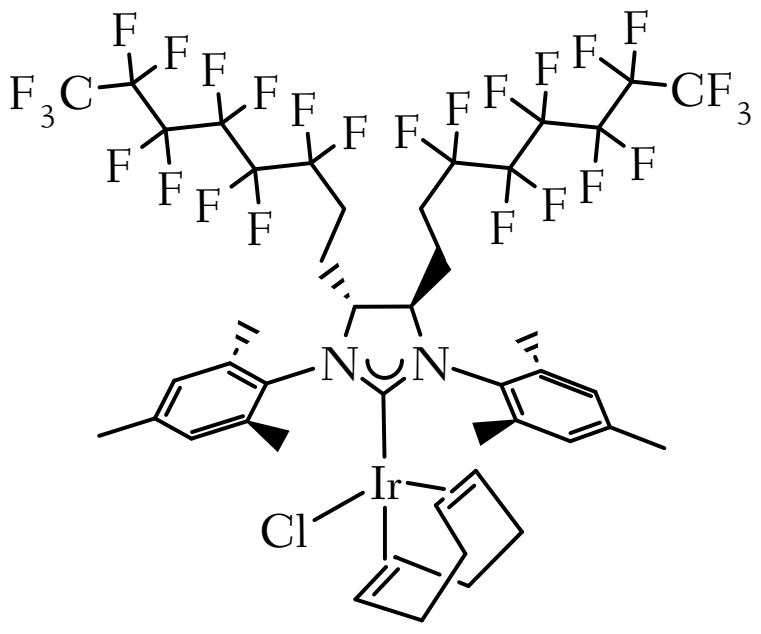

Ir-SIMesF$_2$

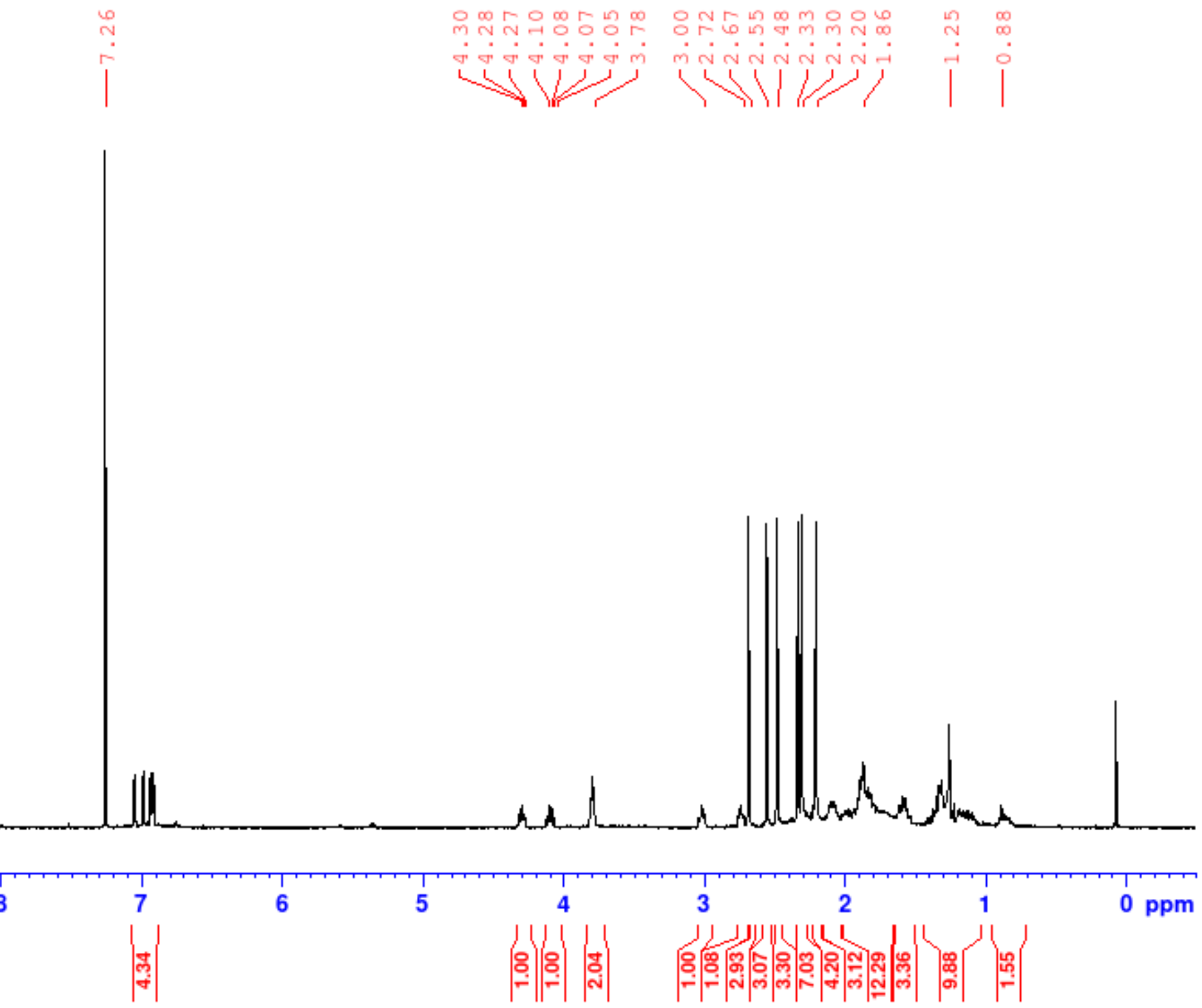

Figure S48. ^{1}H NMR Spectrum, $CDCl_3$, **Ir-SIMesF$_2$**.

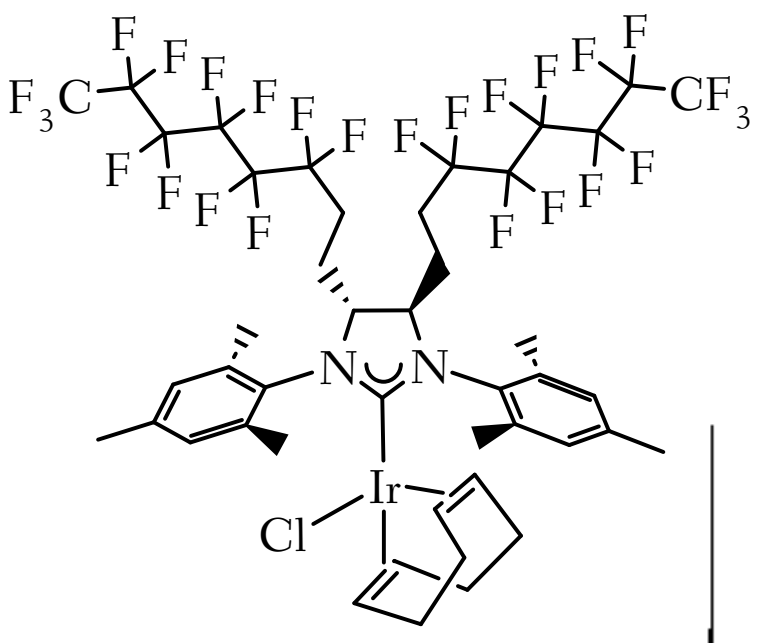

Ir-SIMesF$_2$

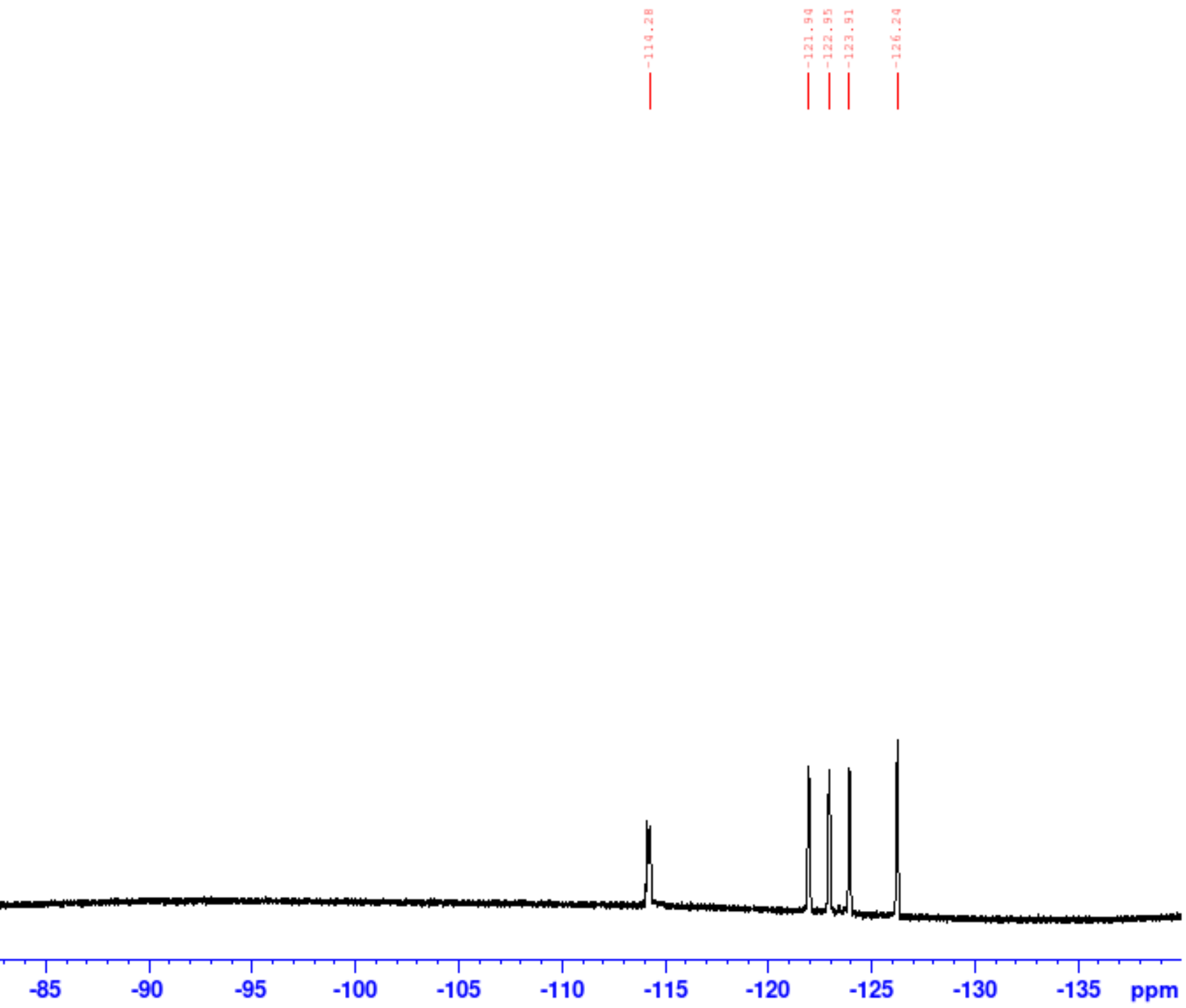

Figure S49. ^{19}F NMR Spectrum 376 MHz, $CDCl_3$, **Ir-SIMesF$_2$**.

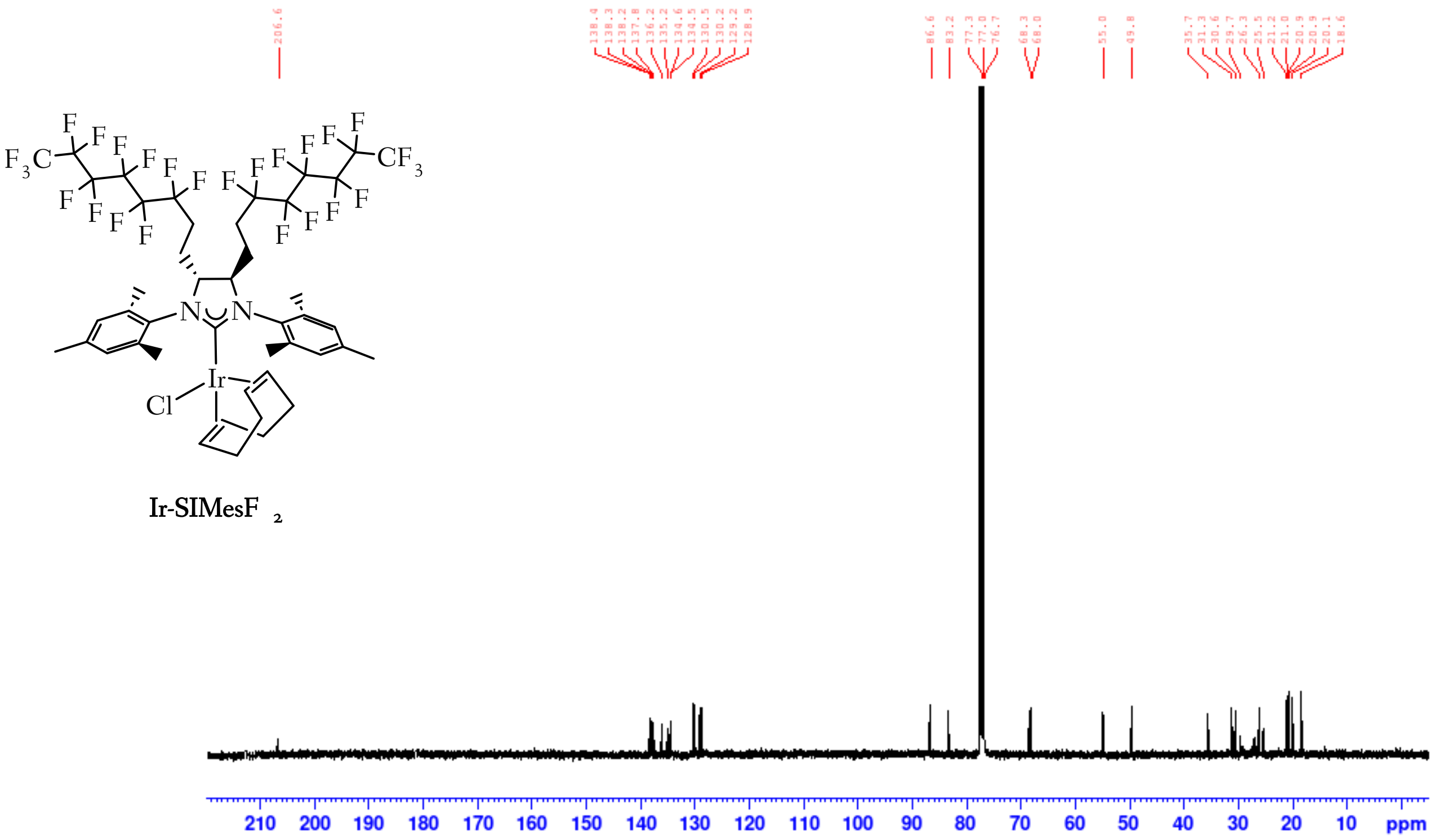

Ir-SIMesF $_2$

Figure S50. ^{13}C NMR Spectrum 400 MHz, $CDCl_3$, **Ir-SIMesF$_2$**.

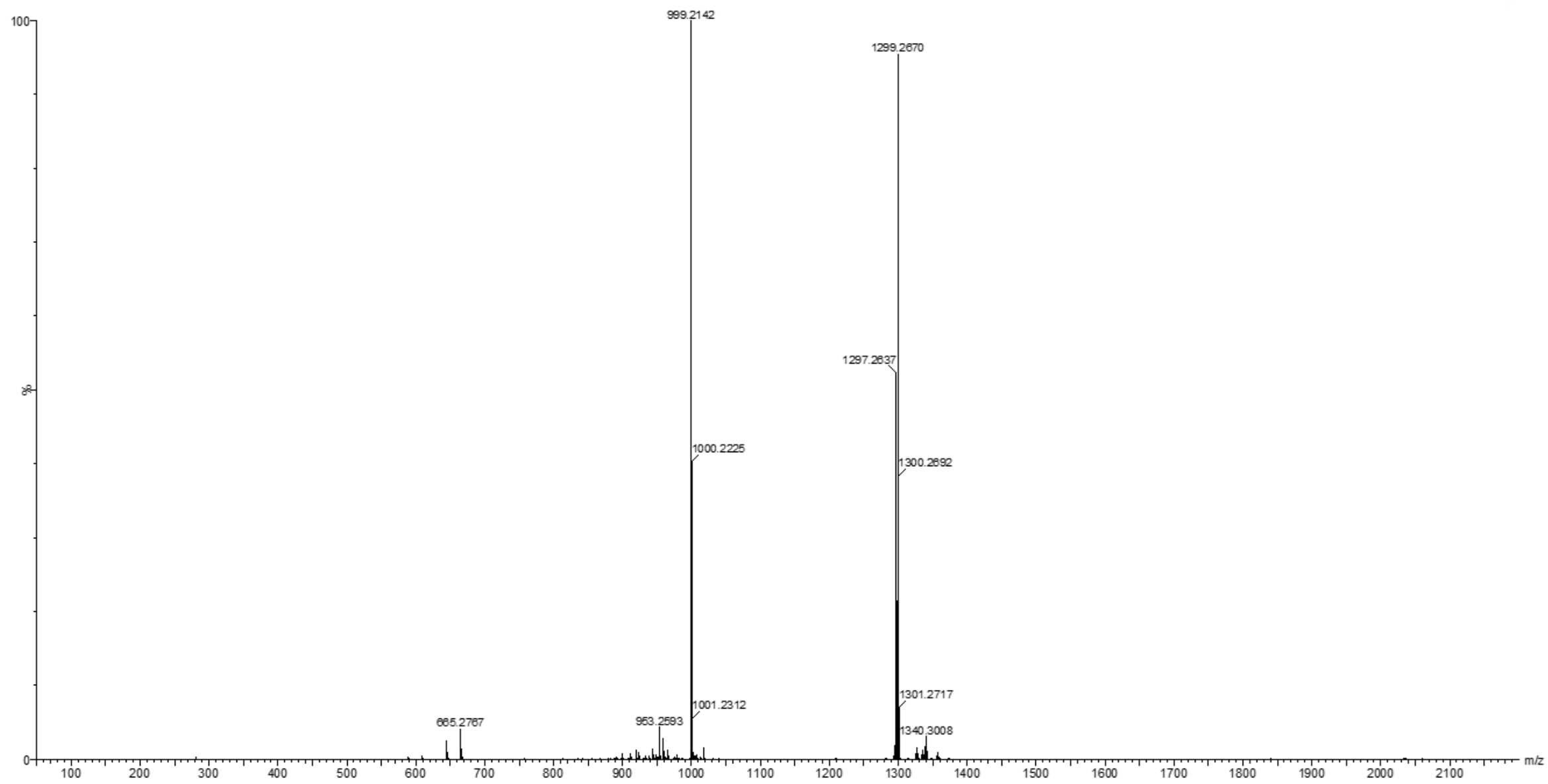

Figure S51. HRMS (ESI+), $CDCl_3$, **Ir-SIMesF$_2$**.

.

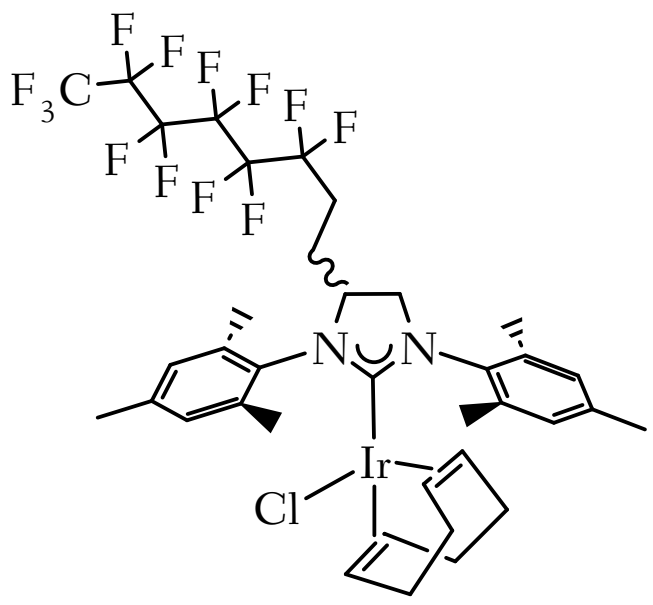

Ir-SIMesF1

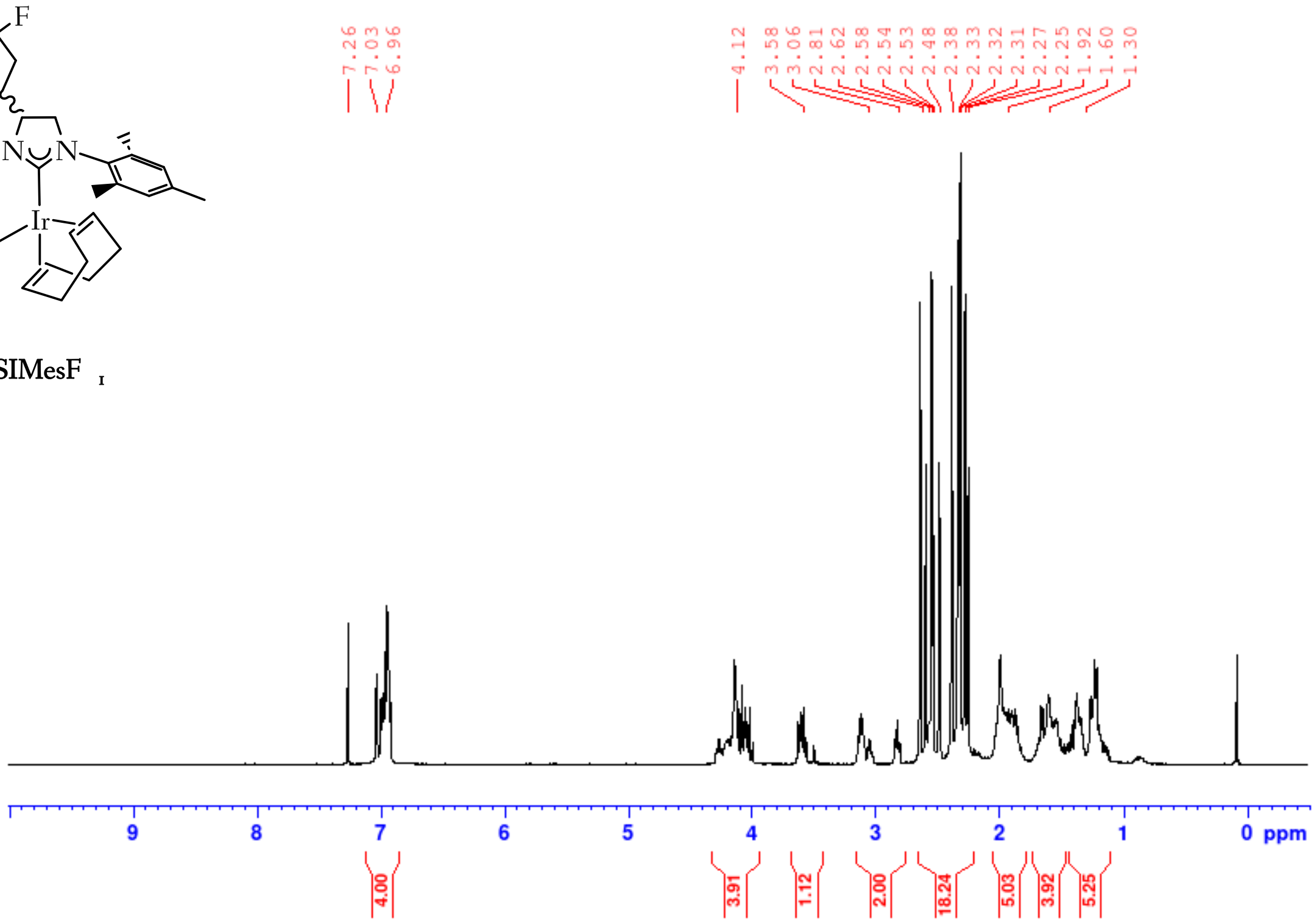

Figure S52. ^{1}H NMR Spectrum 400 MHz, $CDCl_3$, **Ir-SIMesF1**.

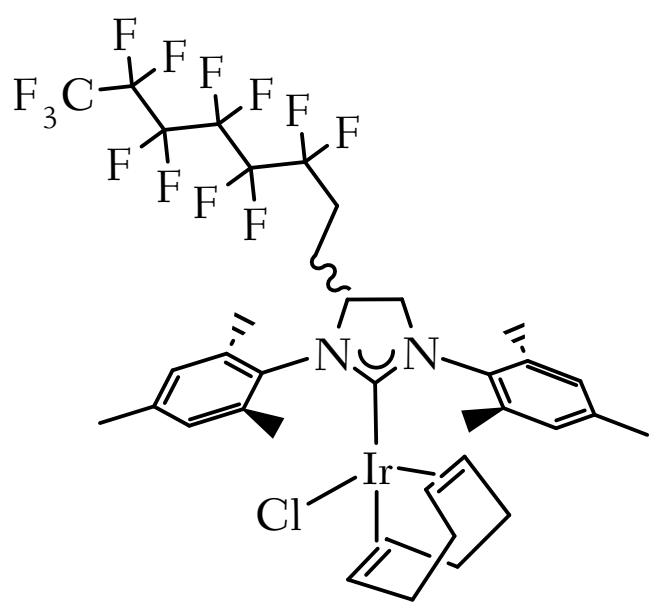

Ir-SIMesF1

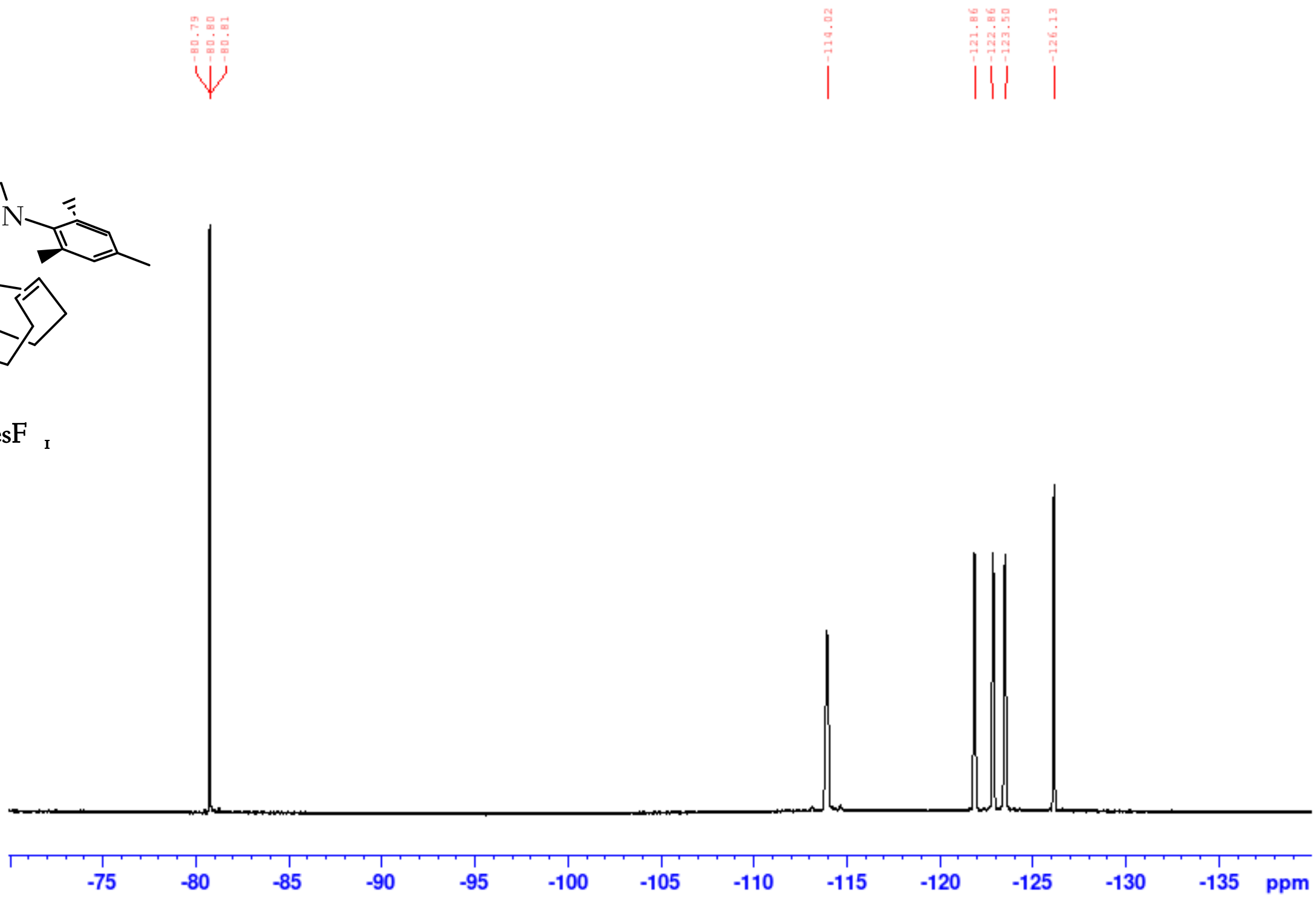

Figure S53. ^{19}F NMR Spectrum 376 MHz, $CDCl_3$, **Ir-SIMesF1**.

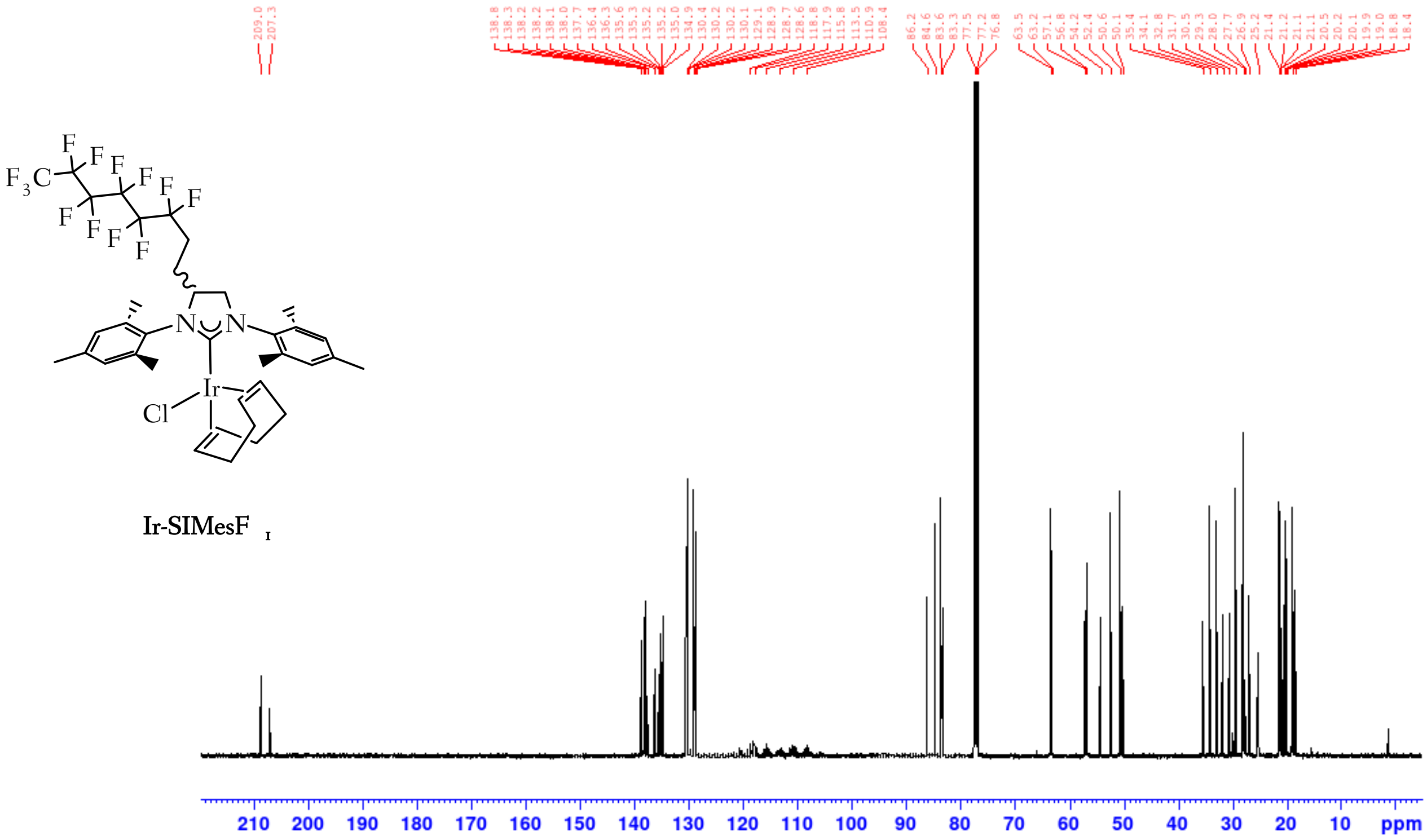

Figure S54. ^{13}C NMR Spectrum 400 MHz, $CDCl_3$, **Ir-SIMesF$_1$**.

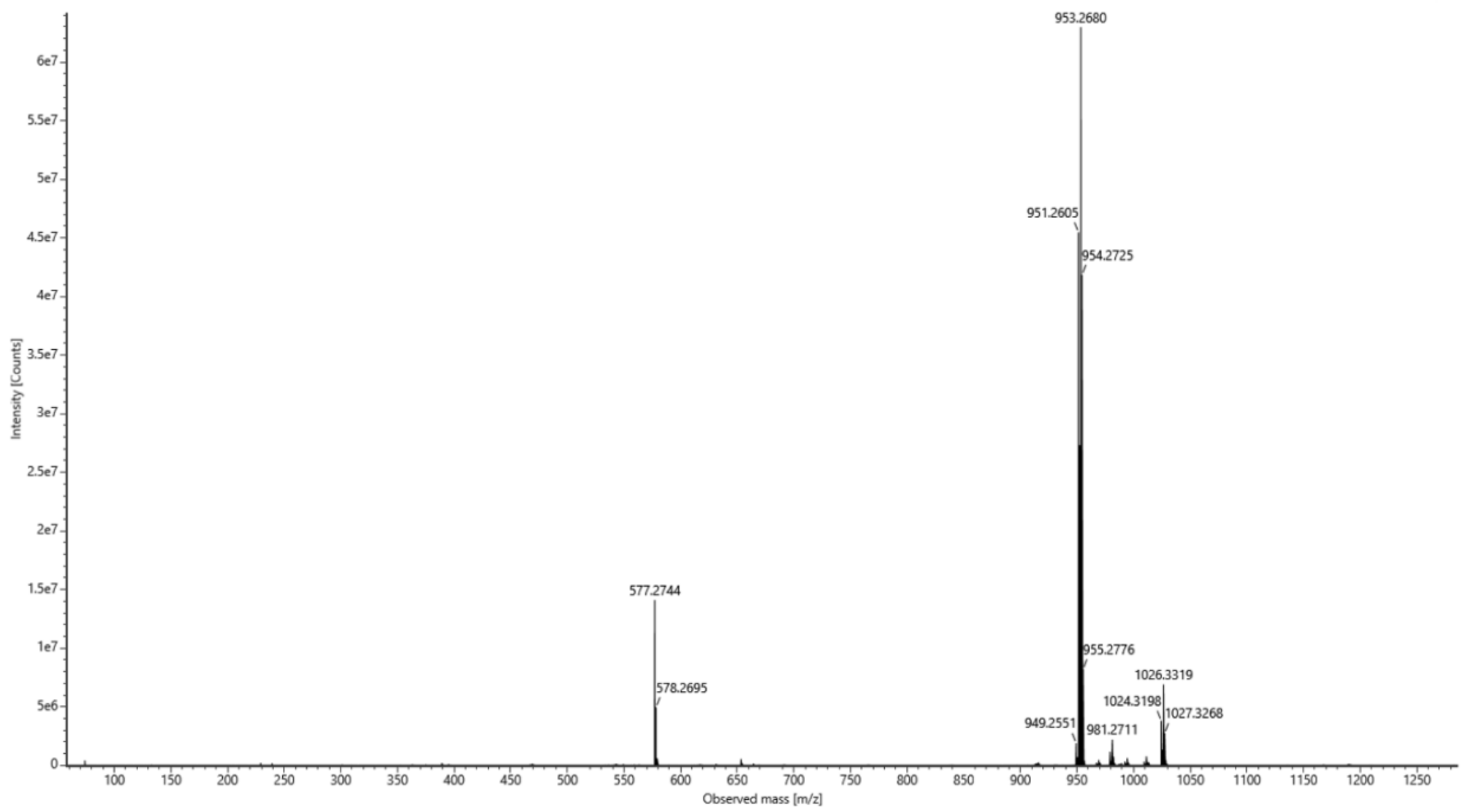

Figure S55. HRMS (ESI+), **Ir-SIMesF$_1$**.

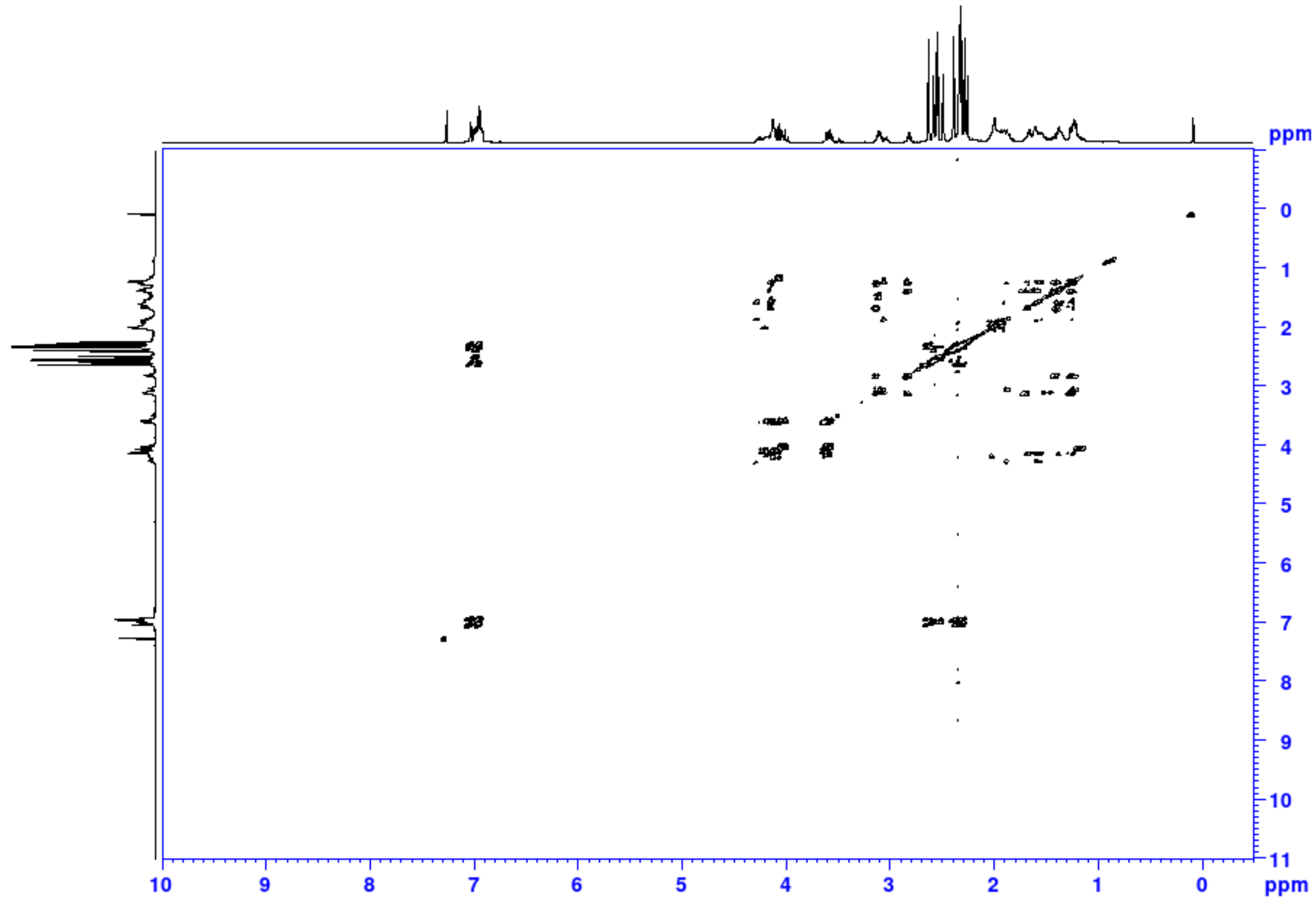

Figure S56. COSY, $CDCl_3$, **Ir-SIMesF$_1$**.

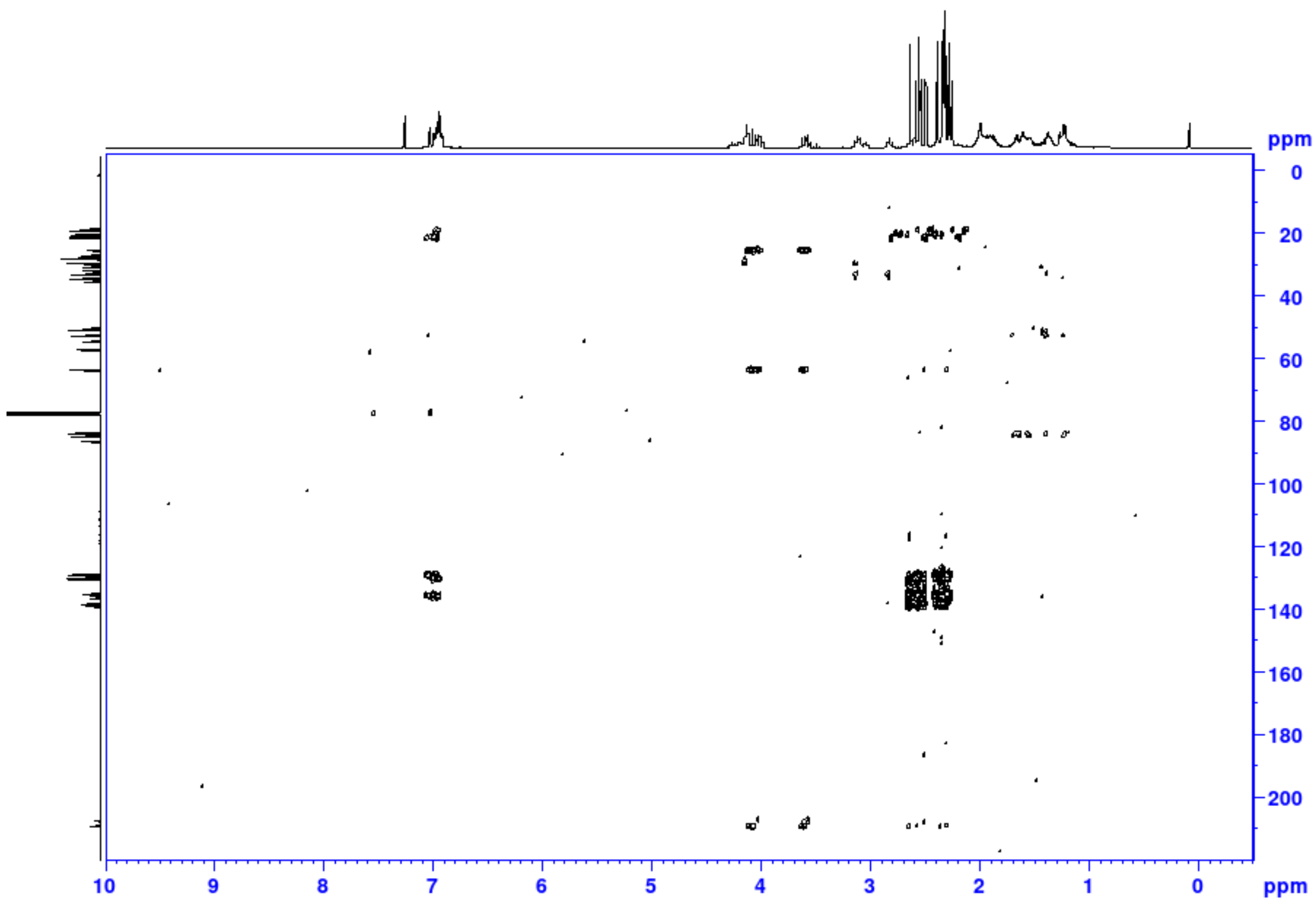

Figure S57. ^1H-^{13}C HSQC, $CDCl_3$, **Ir-SIMesF$_1$**.

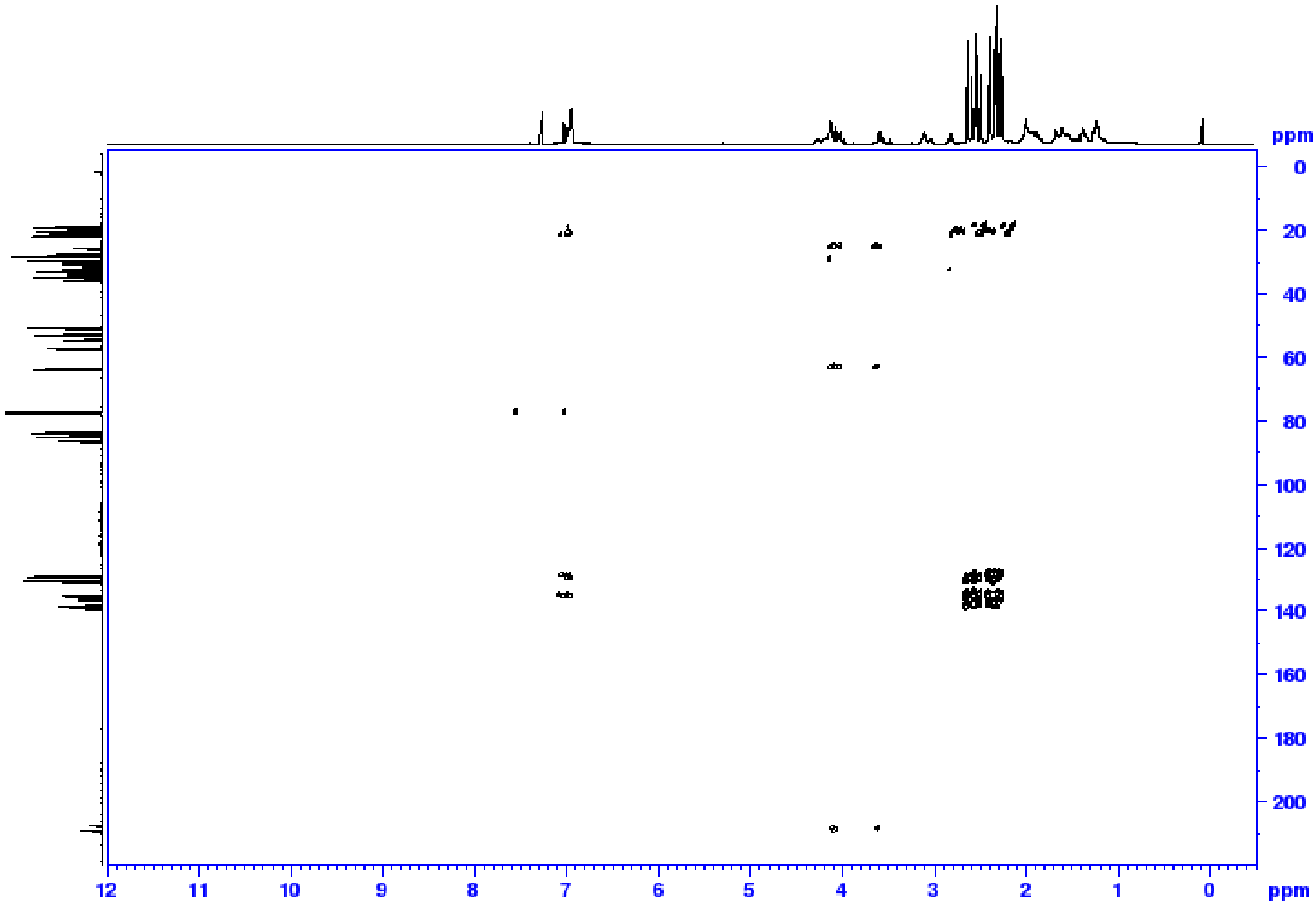

Figure S58. ¹H-¹³C HMBC, $CDCl_3$, **Ir-SIMesF₁**.

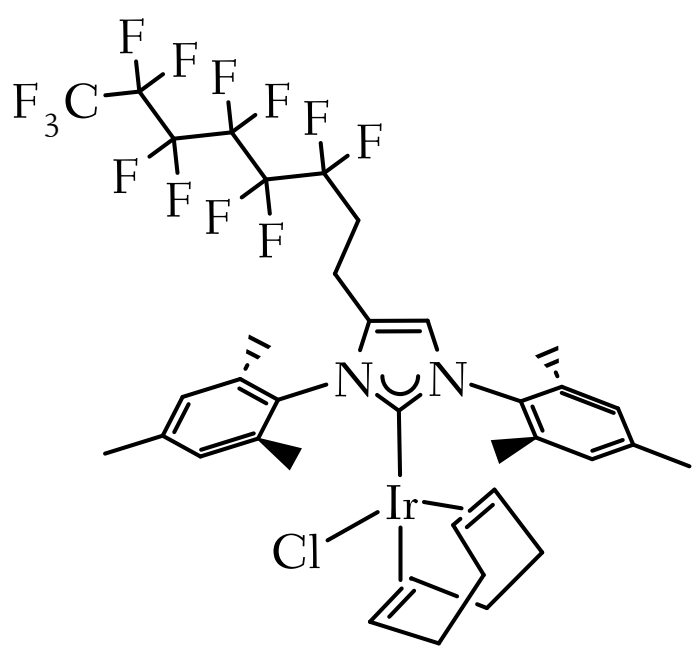

Ir-IMesF₁

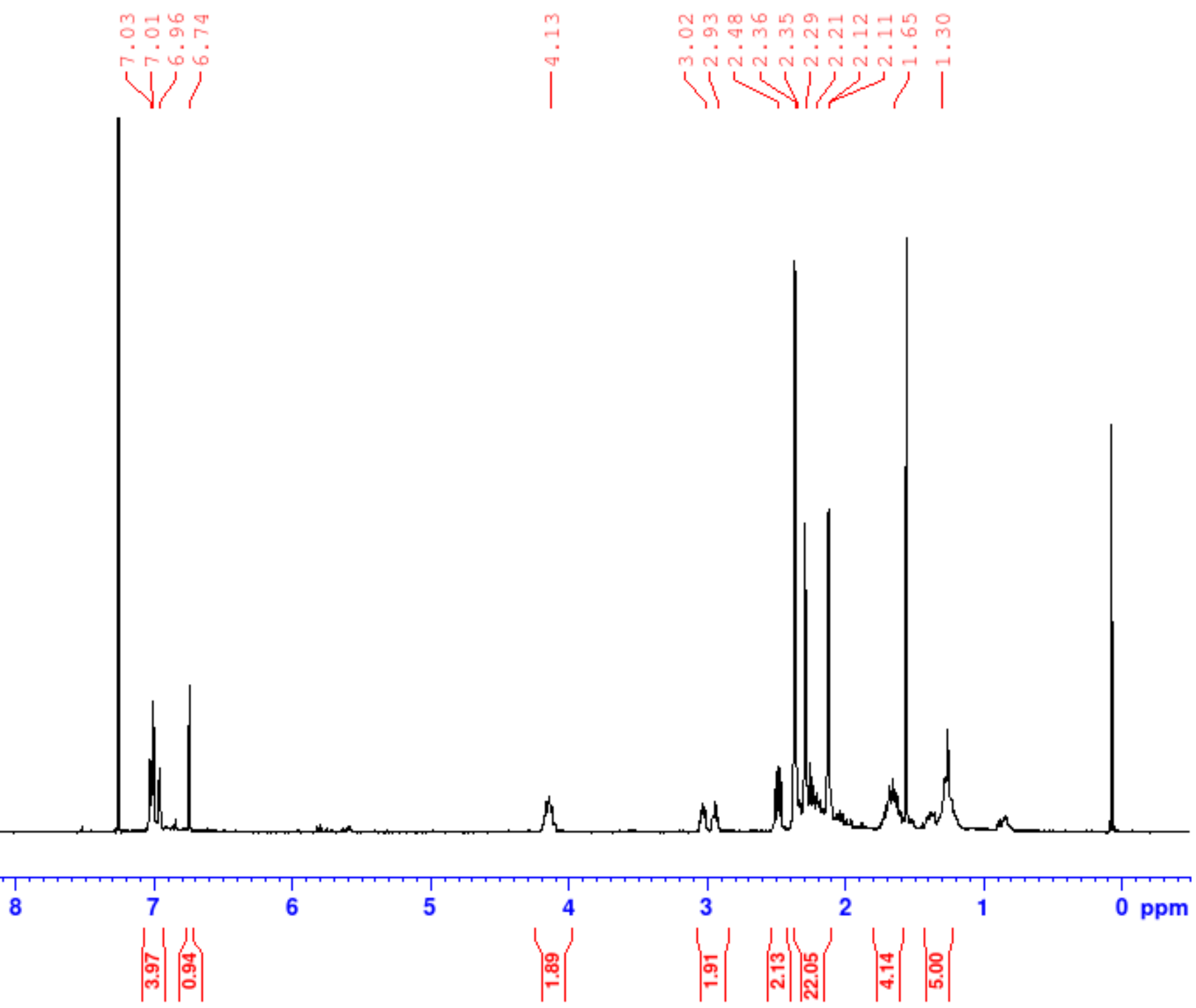

Figure S59. ¹H NMR Spectrum 400 MHz, $CDCl_3$, **Ir-IMesF₁**.

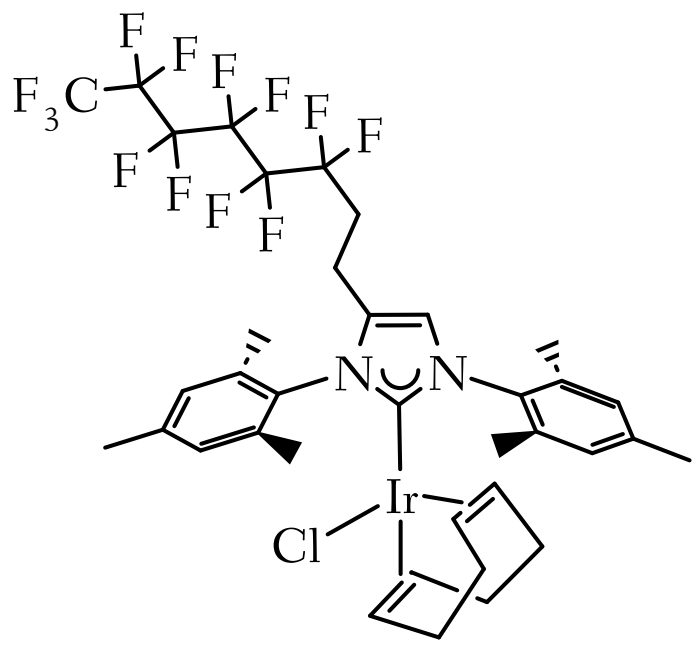

Ir-IMesF 1

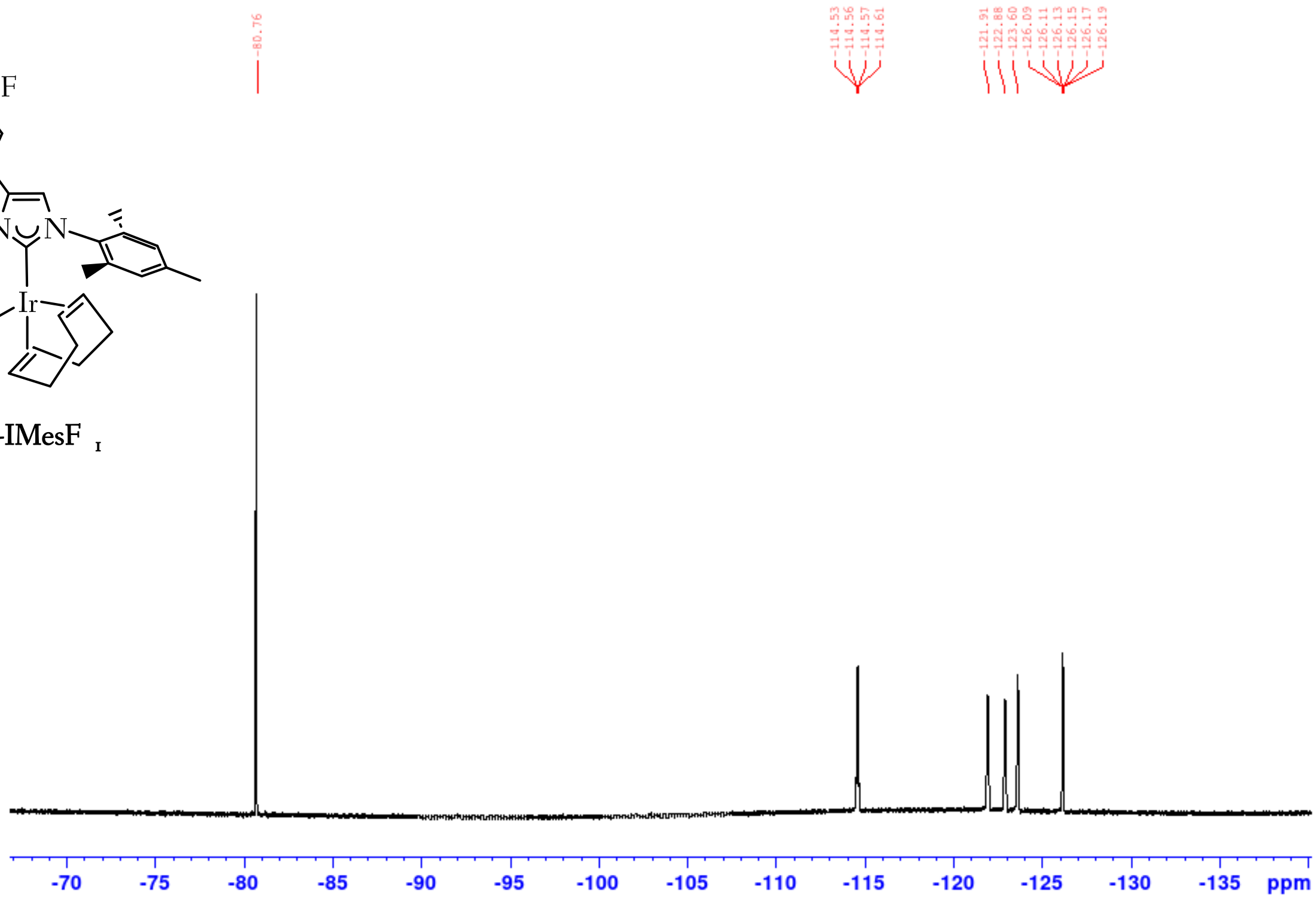

Figure S60. ^{19}F NMR Spectrum 376 MHz, $CDCl_3$, **Ir-IMesF**$_1$.

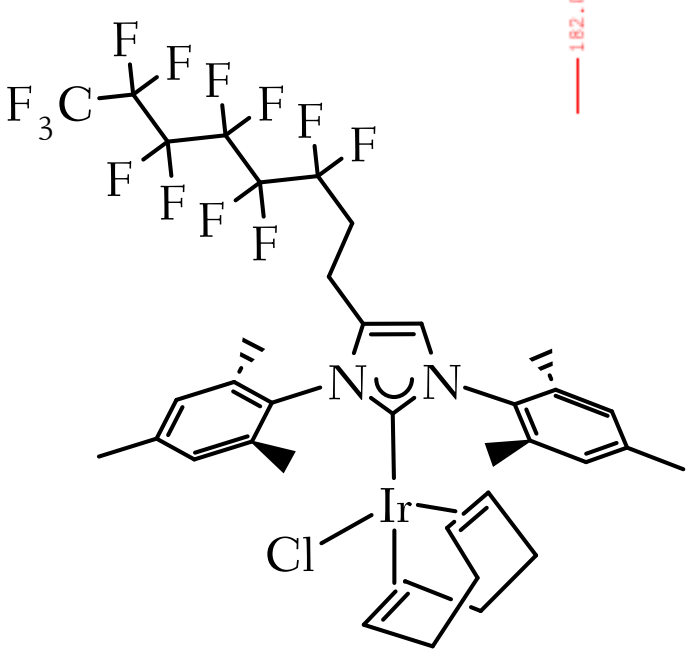

Ir-IMesF 1

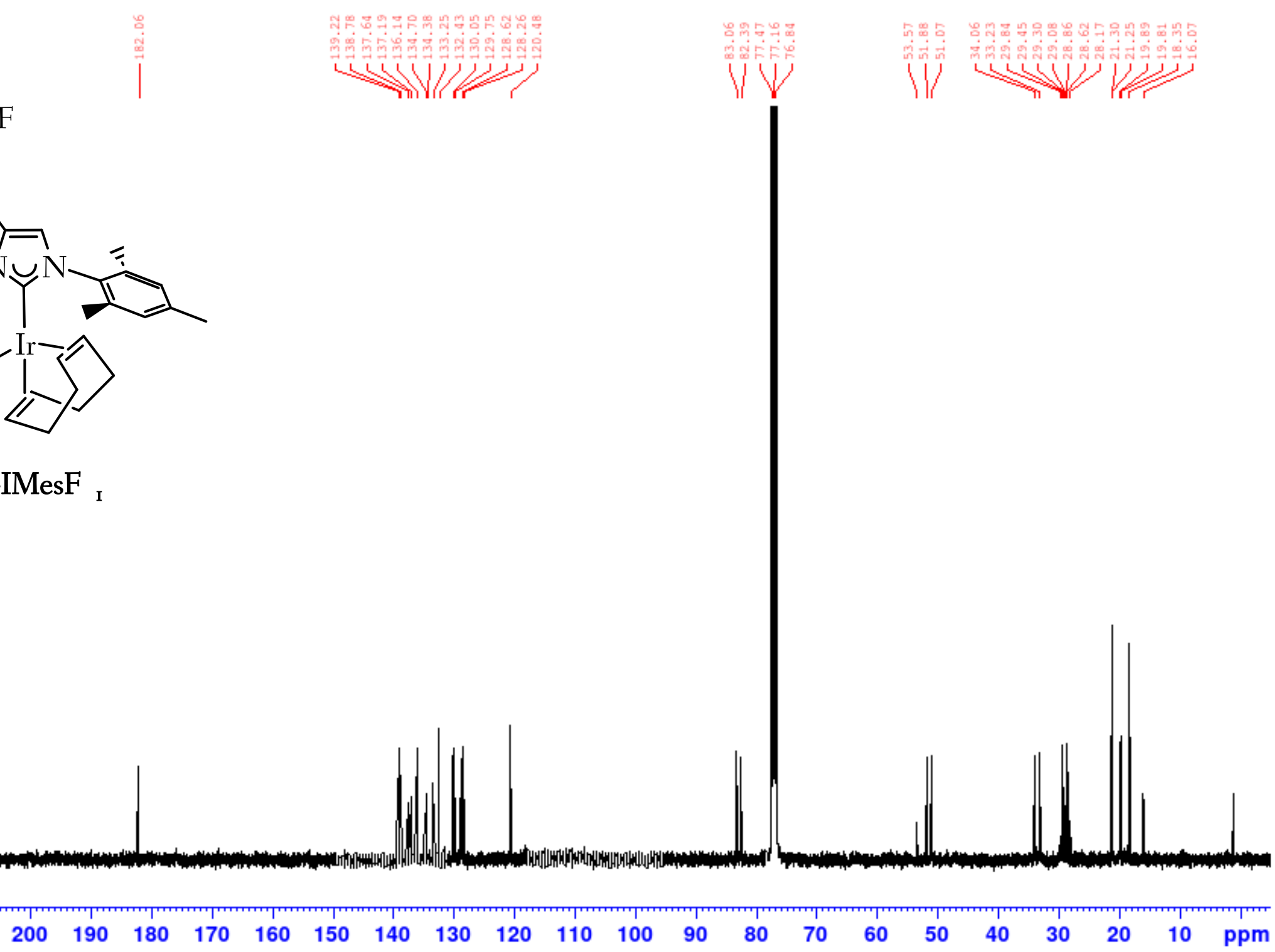

Figure S61. ^{13}C NMR Spectrum 100.5 MHz, $CDCl_3$, **Ir-IMesF1**.

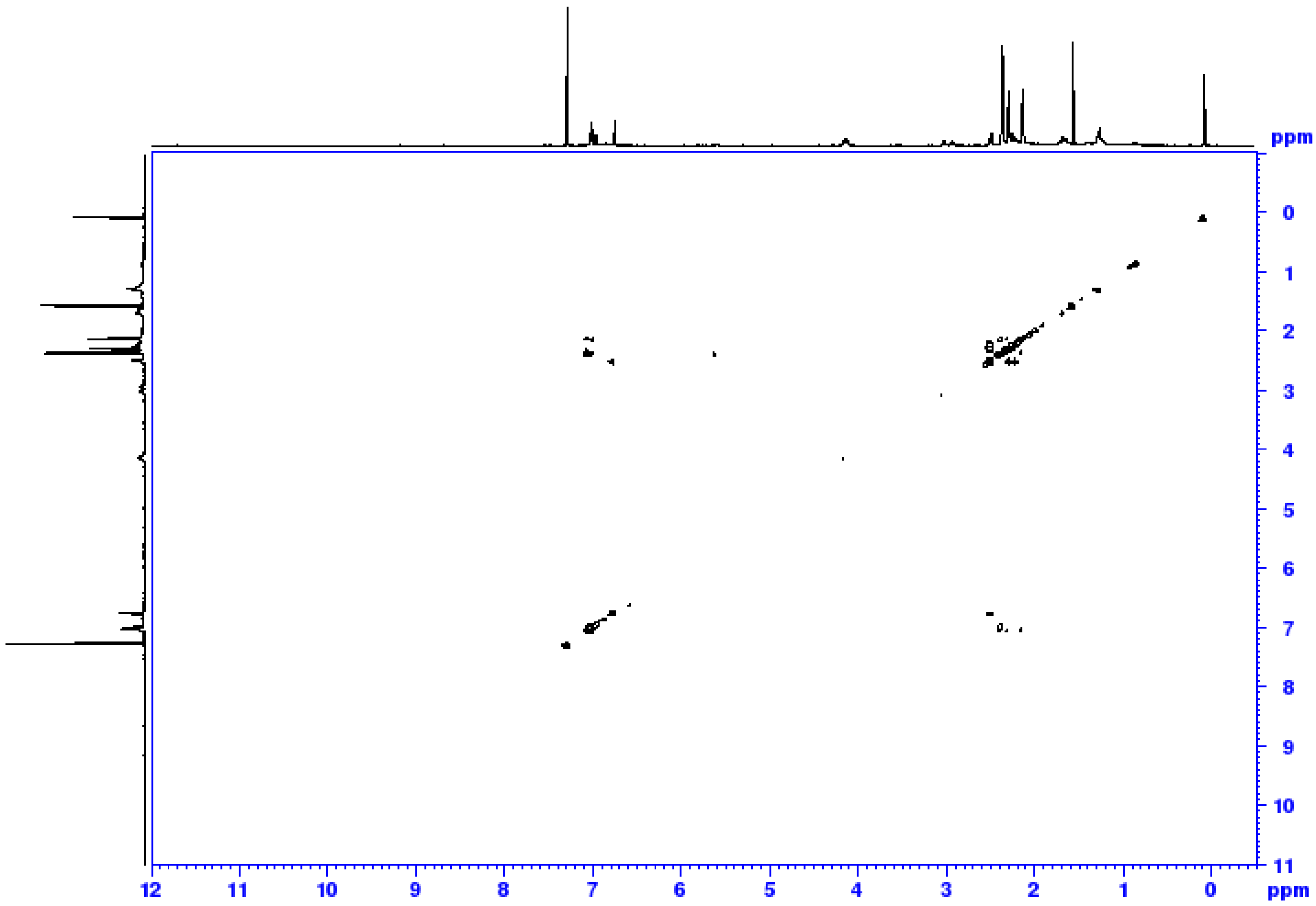

Figure S62. COSY, $CDCl_3$, Ir-IMesF1

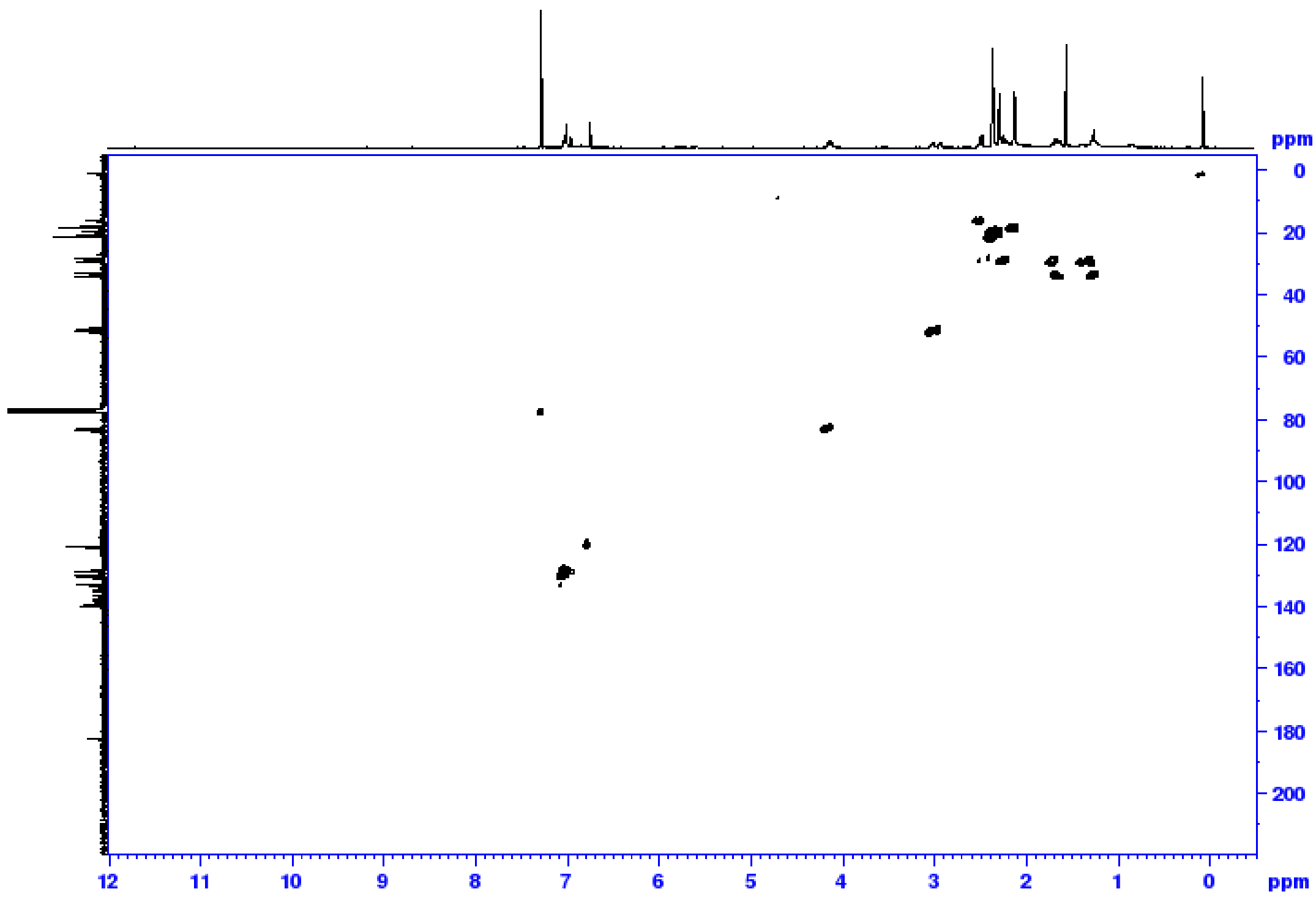

Figure S63. ^{1}H-^{13}C HSQC, $CDCl_3$, Ir-IMesF1.

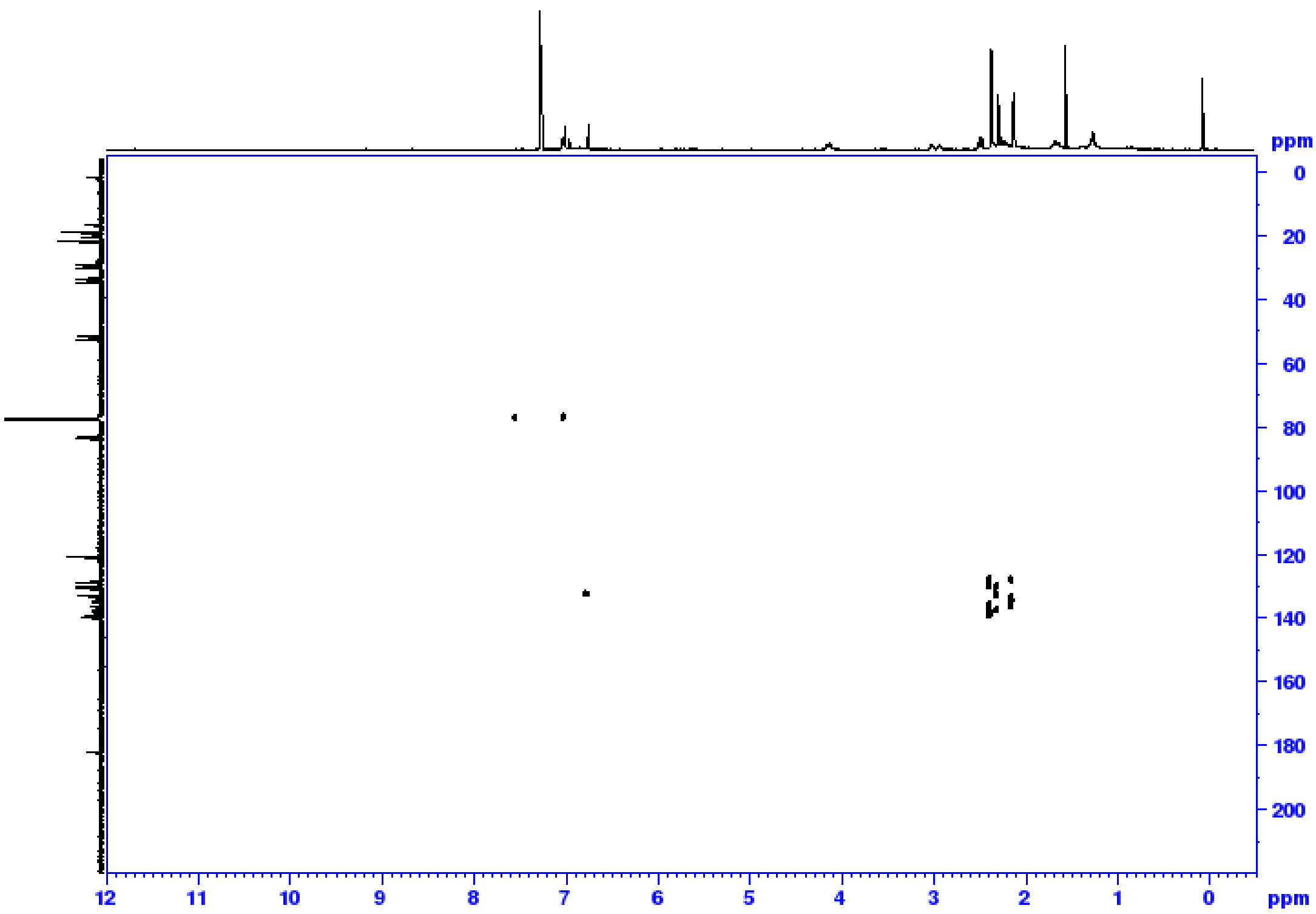

Figure S64. ^{1}H-^{13}C HMBC, $CDCl_3$, **Ir-IMesF1**.

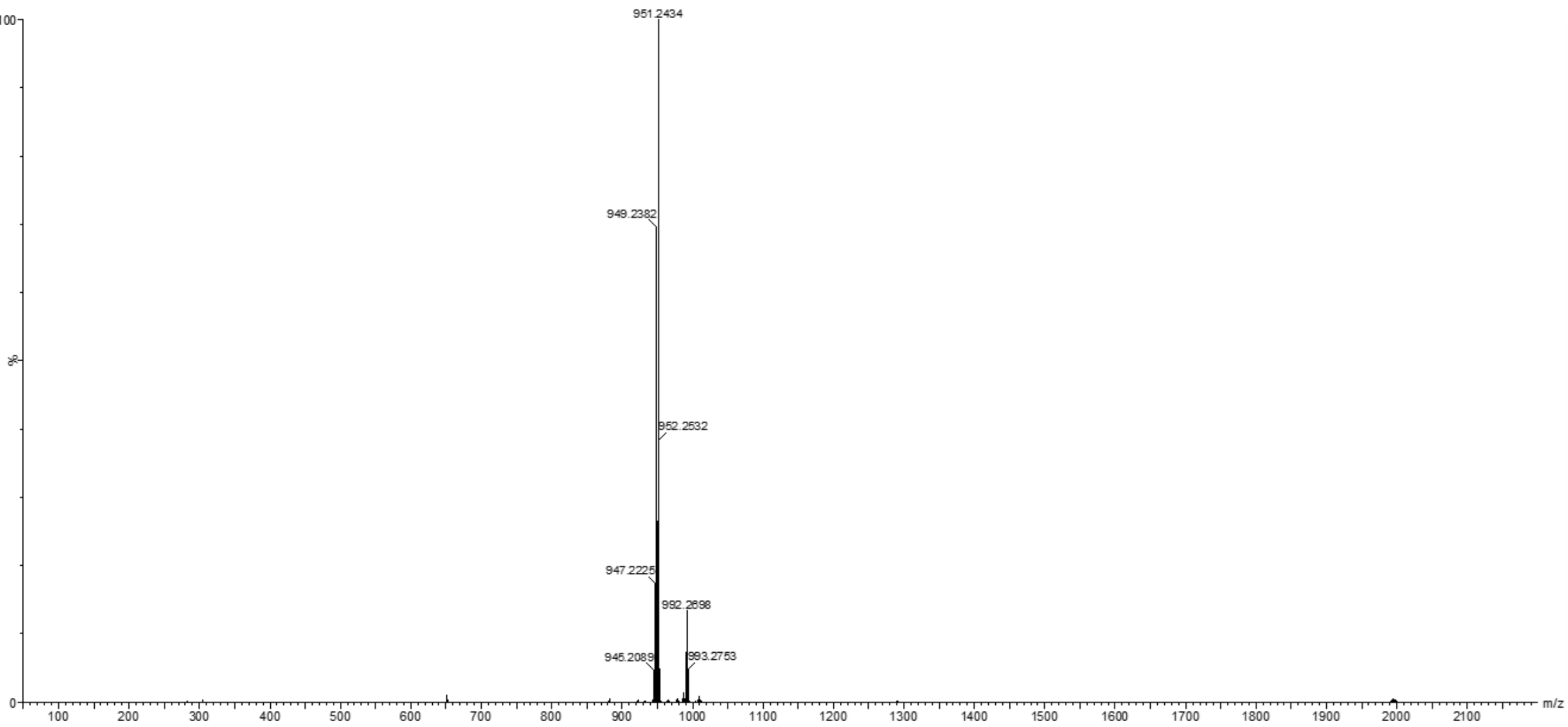

Figure S65. HRMS (ESI+) **Ir-IMesF1**.

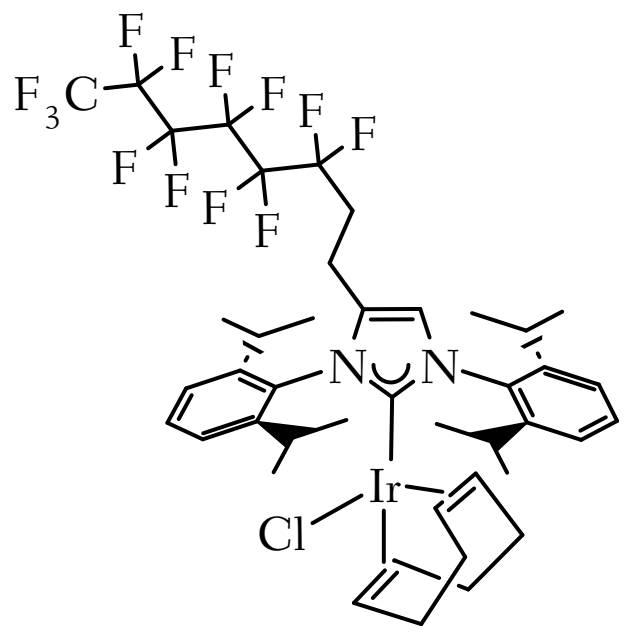

Ir-IPrF 1

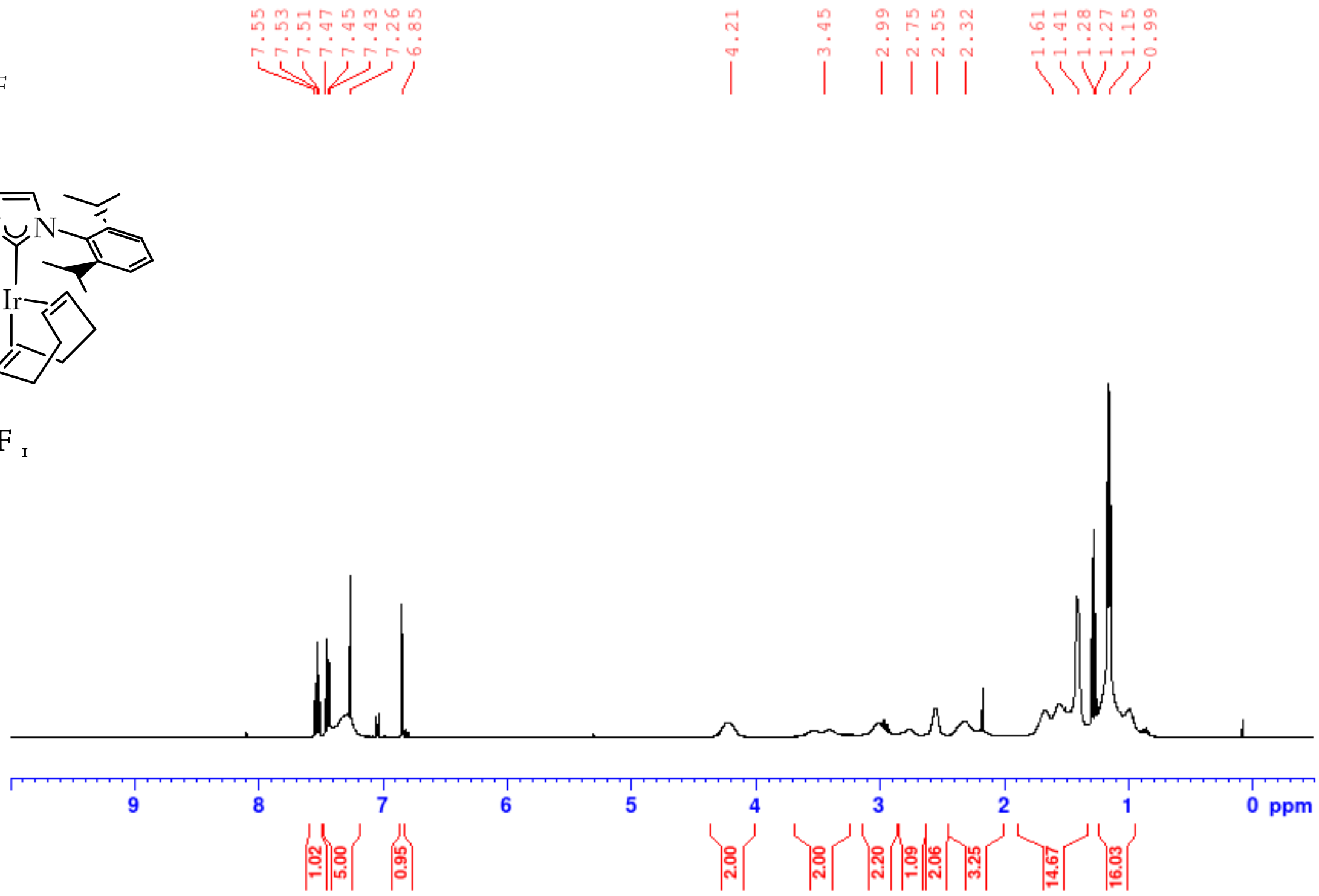

Figure S66. ^{1}H NMR Spectrum 400 MHz, $CDCl_3$, **Ir-IPrF**$_1$.

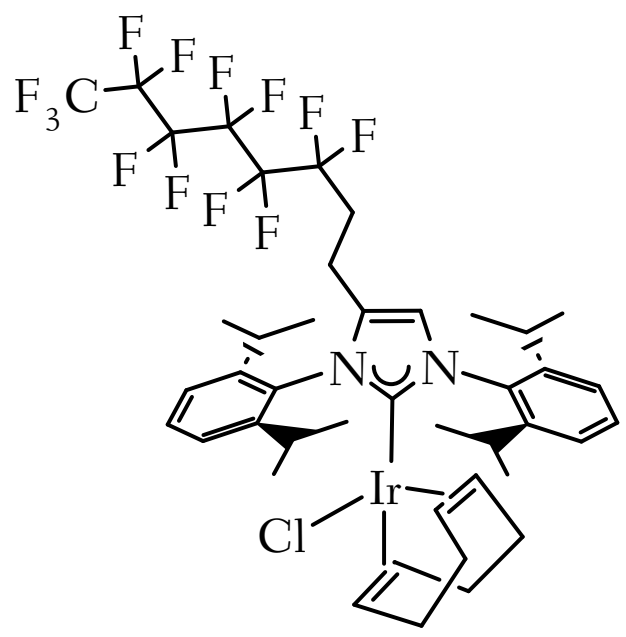

Ir-IPrF 1

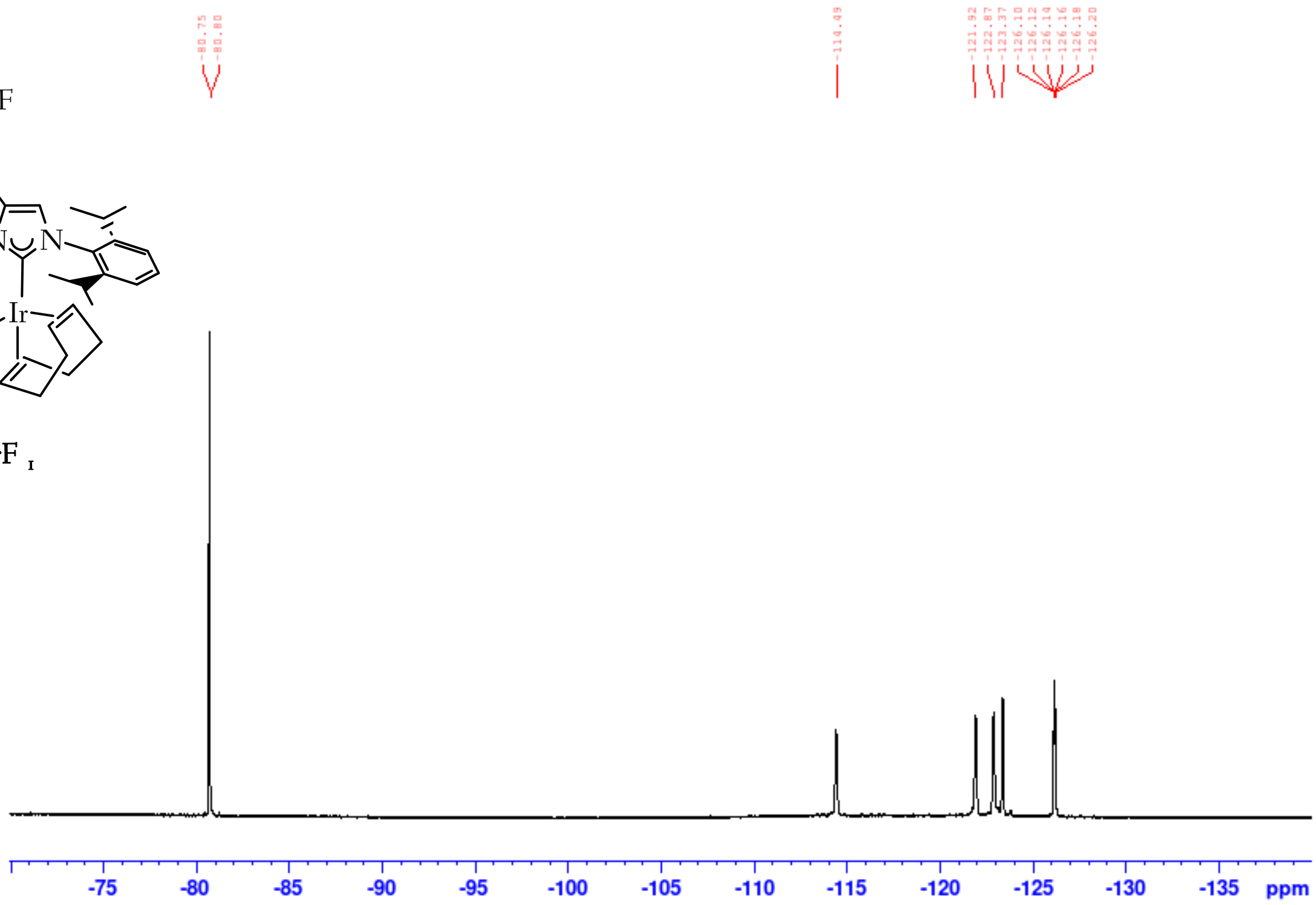

Figure S67. ^{19}F NMR Spectrum 376 MHz, $CDCl_3$, **Ir-IPrF**$_1$.

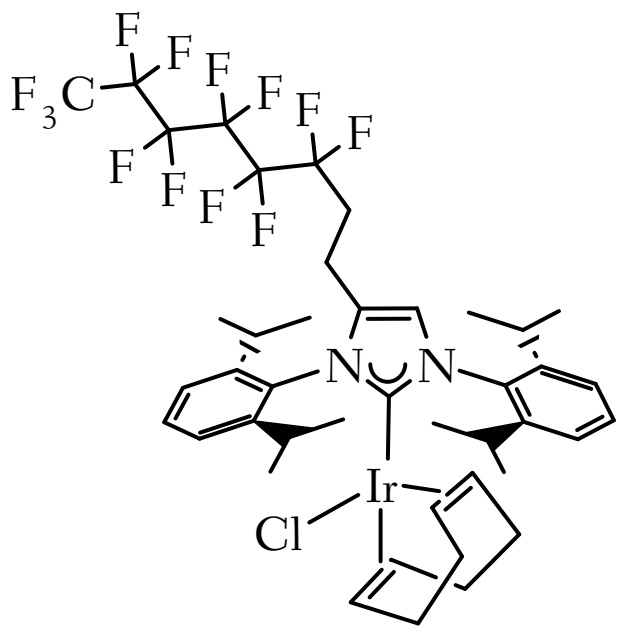

Ir-IPrF$_1$

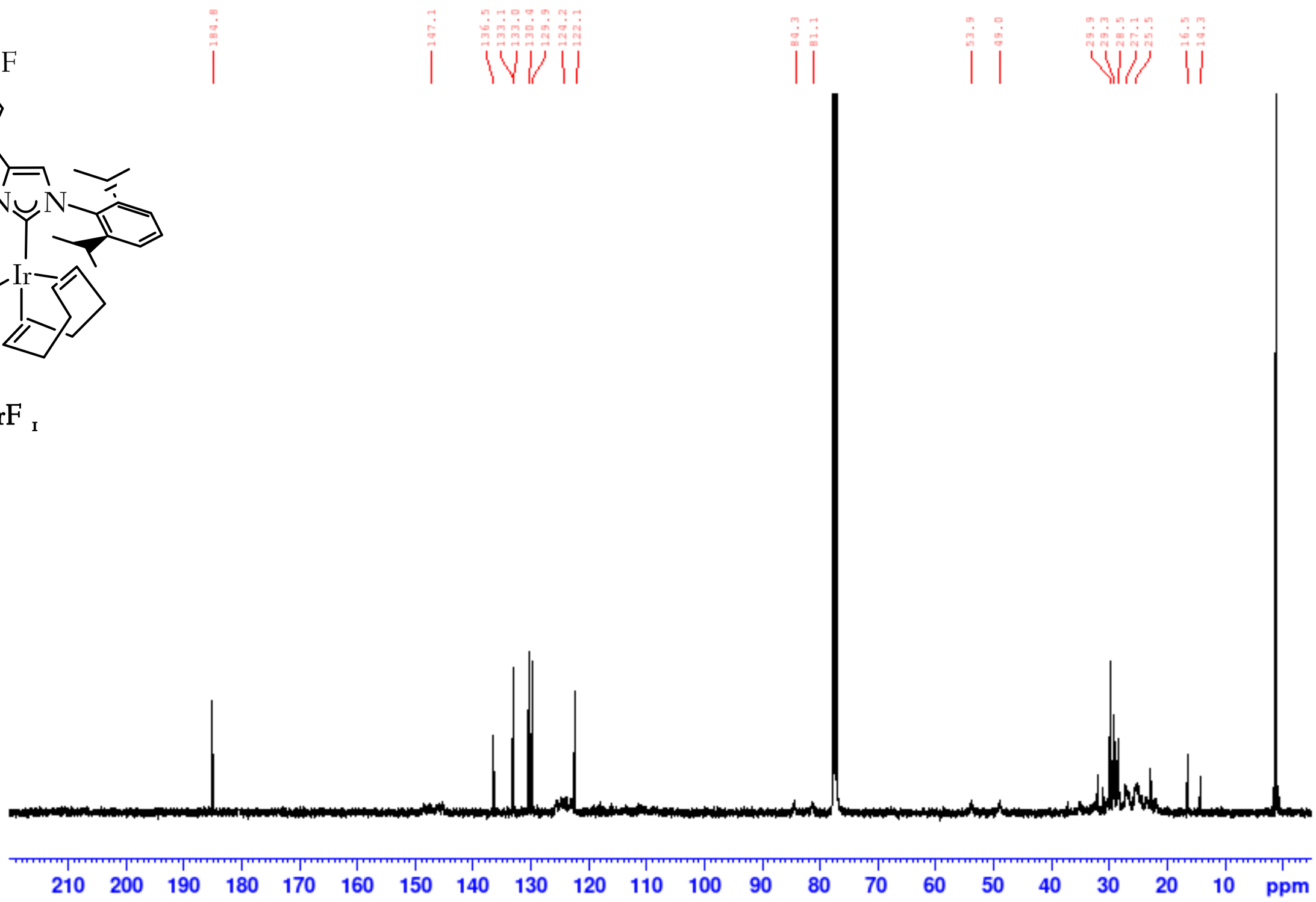

Figure S68. ^{13}C NMR Spectrum 100.5 MHz, $CDCl_3$, **Ir-IPrF$_1$**.

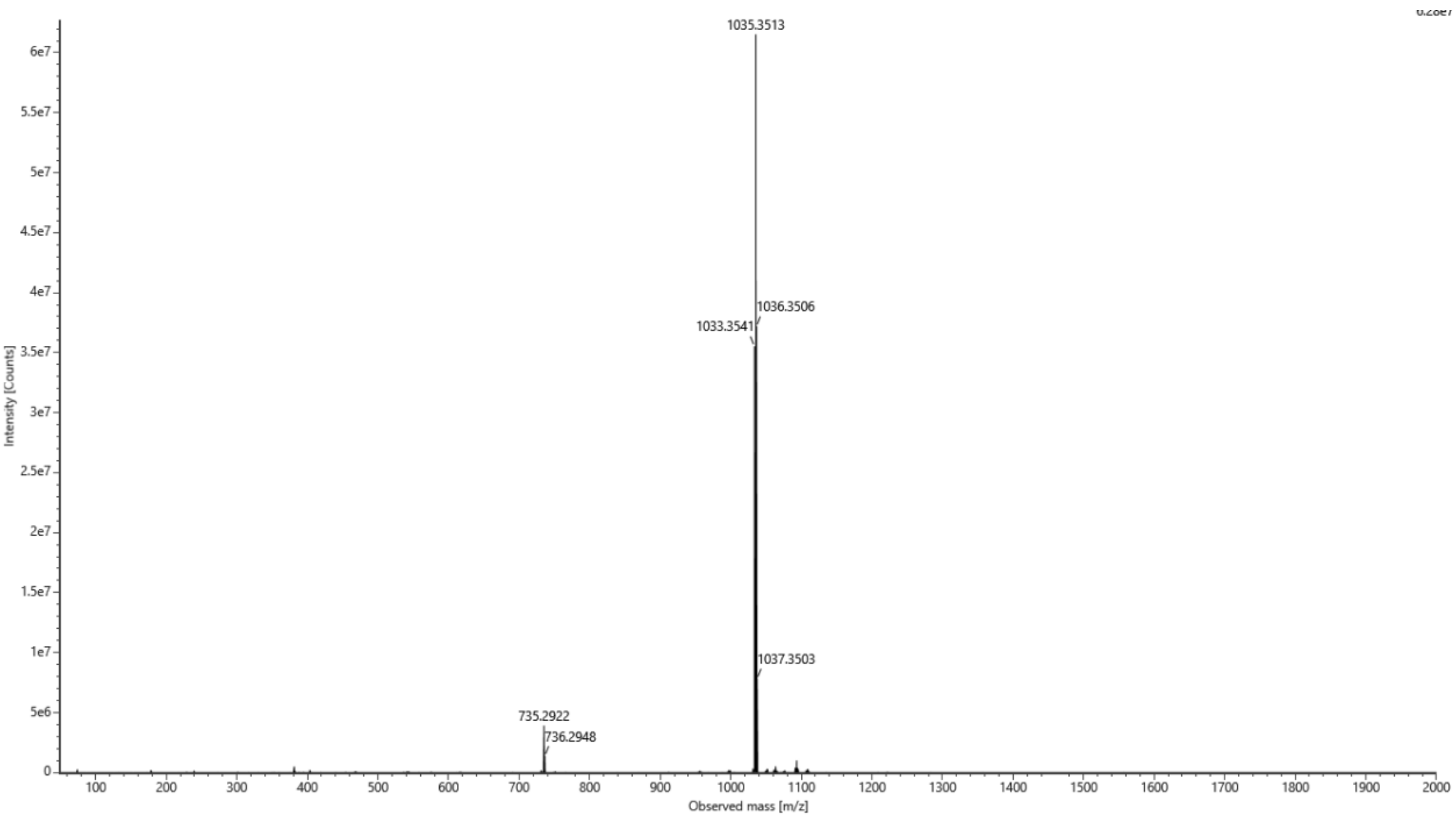

Figure S69. HRMS (ESI+) **Ir-IPrF$_1$**

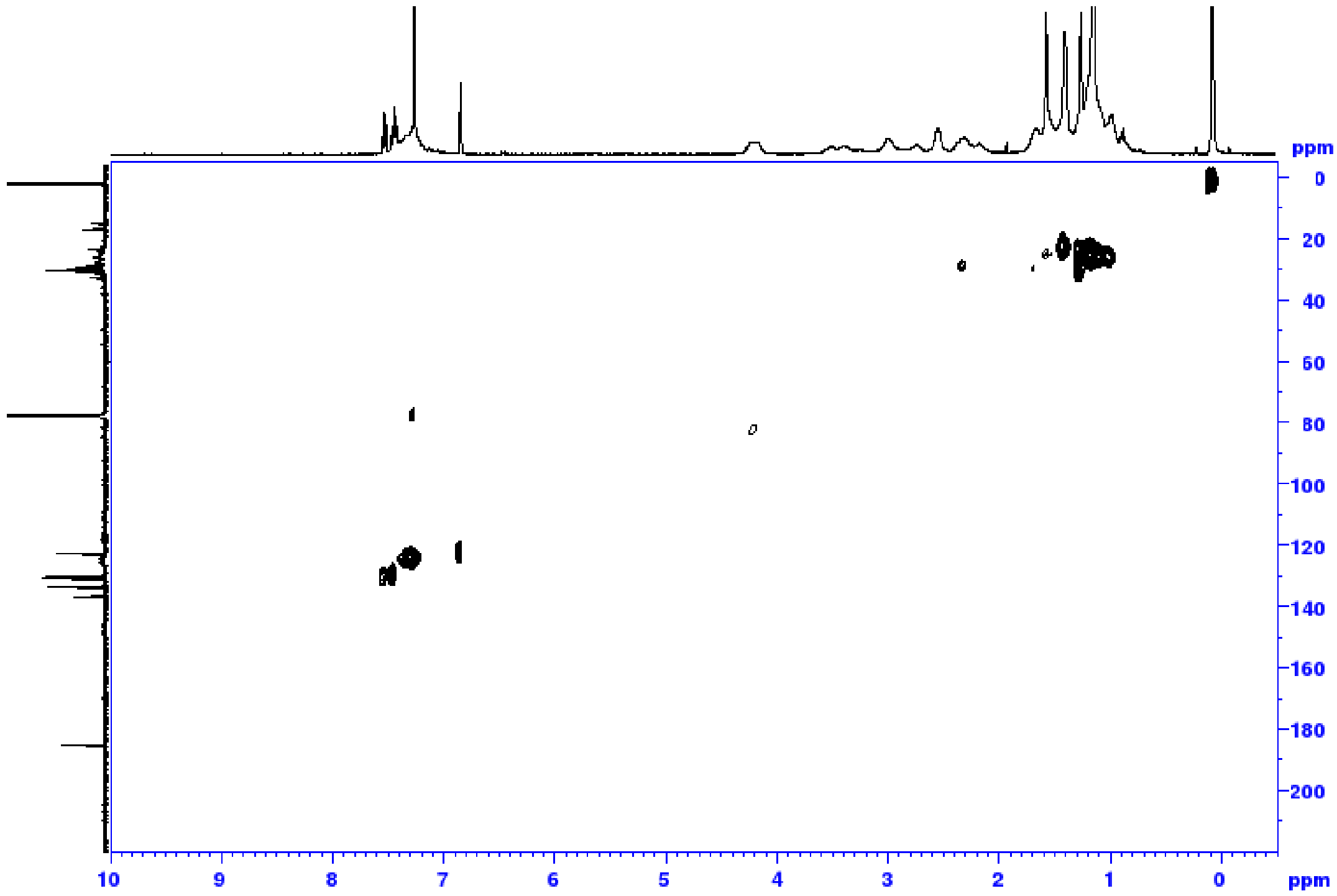

Figure S70. ^{1}H-^{13}C HSQC Spectrum, $CDCl_3$, **Ir-IPrF$_1$**.

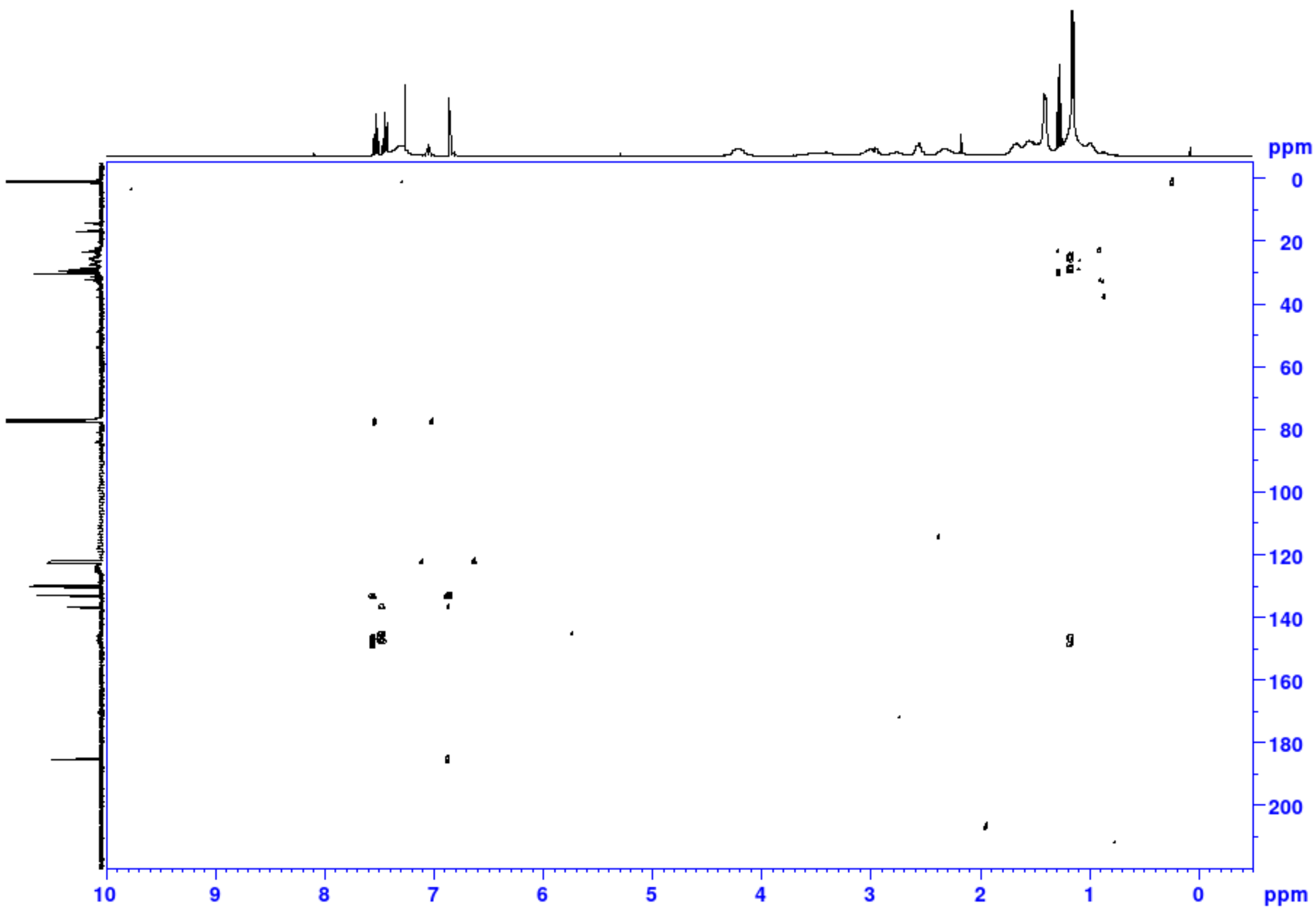

Figure S71. ^{1}H-^{13}C HMBC Spectrum, $CDCl_3$, **Ir-IPrF$_1$**.

5. Hyperpolarization experiments

In all characterization experiments that follow the sample consisted of 700 μL of a solution containing 6 mM catalyst, 20 mM sodium [1-^{13}C]pyruvate, and 30 mM DMSO in methanol-d_4. Prior to measurements, the catalysts were activated for 10 min at 6 bar and ~30 sccm parahydrogen at room temperature.

5.1. Catalyst activation

The hydride region was investigated at 265 K for **Ir-IMesF$_1$**. The sample was pressurized at 6 bar and consecutive spectra under 20 s bubbling of pH_2 (50%) were acquired.

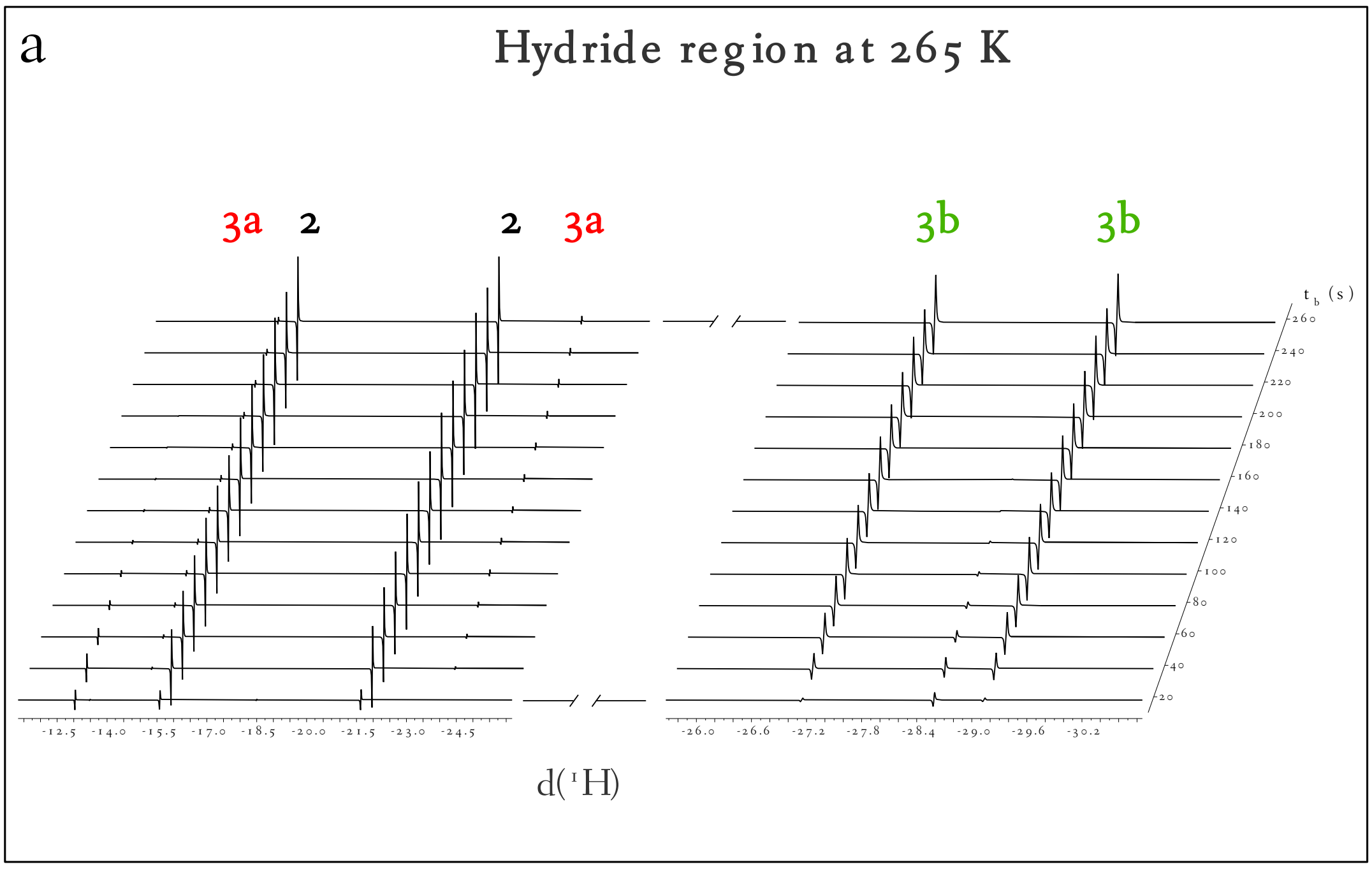

Figure S72: *Hydride region at 265 K for **Ir-IMesF$_1$** under consecutive 20s-long parahydrogen bubbling.*

5.2. Temperature and Magnetic Field profiles

Following catalyst activation, magnetic field optimization was performed by bubbling 50% parahydrogen for 25 s at 6 bar and ~80 sccm. The sample was then manually transferred in 3.0 s ± 1.5 s to a 1.4 T benchtop NMR spectrometer for signal acquisition. All field profiles were recorded at 280 ± 0.1 K. The optimal transfer field was 0.35 μT for all tested catalysts.

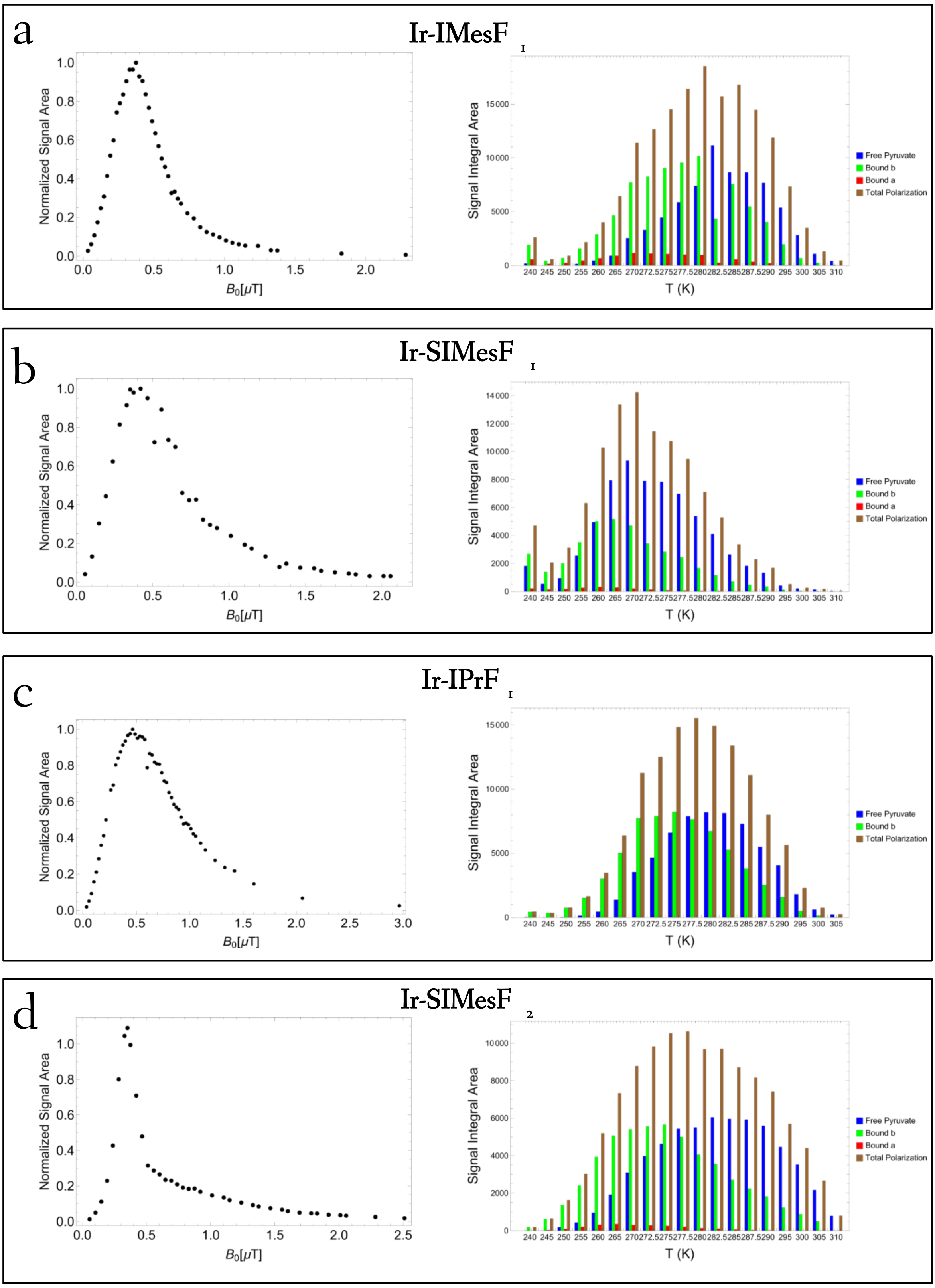

Figure S73: *Magnetic field and temperature dependence of ^{13}C polarization for a solution containing 6 mM catalyst, 20 mM [1-^{13}C]pyruvate, and 30 mM DMSO in CD_3OD: (a)* ***Ir-IMesF$_1$****, (b)* ***Ir-SIMesF$_1$****, (c)* ***Ir-IPrF$_1$****, and (d)* ***Ir-SIMesF$_2$****. Parahydrogen was bubbled for 25 s at 6 bar and 80 sccm. The optimal polarization transfer field (PTF) was ~0.35 μT for all catalysts. Temperature profiles were recorded at PTF of ~0.35 μT under identical bubbling conditions. Maximum total carbon polarization is observed at ~280 K for the unsaturated catalysts* ***Ir-IMesF$_1$*** *and* ***Ir-IPrF$_1$****, and at ~270 K for the saturated* ***Ir-SIMesF$_1$*** *and* ***Ir-SIMesF$_2$****.*

Temperature-dependent experiments were subsequently carried out for each catalyst at ∼0.35 μT under identical bubbling conditions in the range between 240 K and 310 K. The sample temperature was allowed to equilibrate for about 3 minutes using the setup described previously[9] and the SABRE-SHEATH experiment followed as described above. In Fig. S73, bar plots report the integrated signal areas of the **3a** species (red), the longitudinally bound **3b** species (green), and free pyruvate (blue); brown bars denote the total signal at each temperature. An isolated higher signal at 240 K is observed for **Ir-IMesF$_1$** and **Ir-SIMesF$_1$** (Fig. S73a,b). As this feature does not follow the general temperature trend and was not reproducible, it is attributed to residual magnetization at the start of the bubbling sequence.

As previously reported by Ettedgui, Swenson et al.,[4] we also observe an additional peak at low temperature for **Ir-SIMesF$_2$**, albeit with reduced resolution (Fig. S74). This feature was tentatively attributed by the authors to catalyst chirality, leading to chemically inequivalent equatorially bound forms (**3b** and **3b'**). We do not observe an analogous signal for the single-chain fluorinated catalysts reported here. This may indicate that the bulkier double-chain environment restricts the mobility of the NHC ligand around the Ir–C bond, whereas such effects are less pronounced for the single-chain systems, or that the limited resolution at 1.4 T prevents its detection.

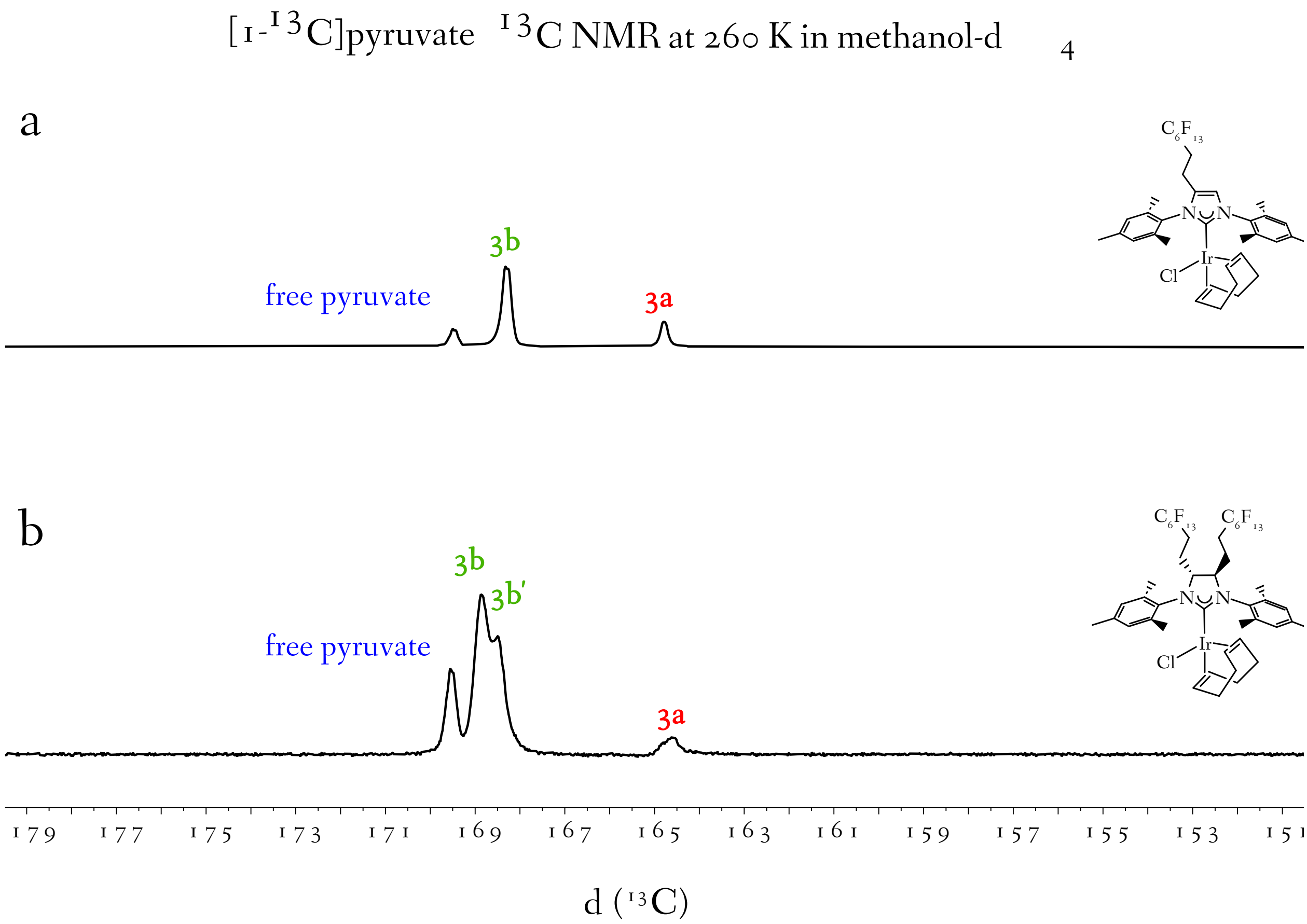

Figure S74: *a) ^{13}C NMR at 1.4 T and 260 K of 6mM* ***Ir-IMesF$_1$*** *30 mM [1-^{13}C] pyruvate sodium and 30 mM DMSO in methanol-d$_4$ at 260 K; b) the same as in a) for* ***Ir-SIMesF$_2$****.*

The maximum total polarization is observed at 280 ± 0.1 K for the unsaturated catalysts **Ir-IMesF$_1$** and **Ir-IPrF$_1$**, and at 270 ± 0.1 K for the saturated **Ir-SIMesF$_1$** and **Ir-SIMesF$_2$**, in substantial agreement with earlier reports.[4]

5.3. Build-up times

Following catalyst activation, the buildup kinetics was determined by bubbling 50% parahydrogen at 6 bar and ~80 sccm at 0.35 μT for a variable time and then manually moving the sample to the benchtop NMR at 1.4 T for signal detection.

<table>
<tr>
<td>Ir-IMesF₁</td>
<td>
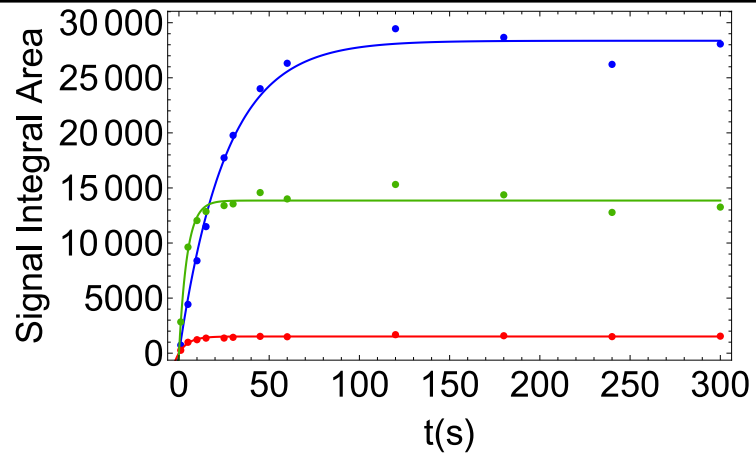

T$_b$(free)=25.8±1.4 s
T$_b$(3a)=5.4±0.6 s
T$_b$(3b)=4.5±0.5 s
T=280 K
</td>
<td></td>
</tr>
<tr>
<td>Ir-IPrF₁</td>
<td>
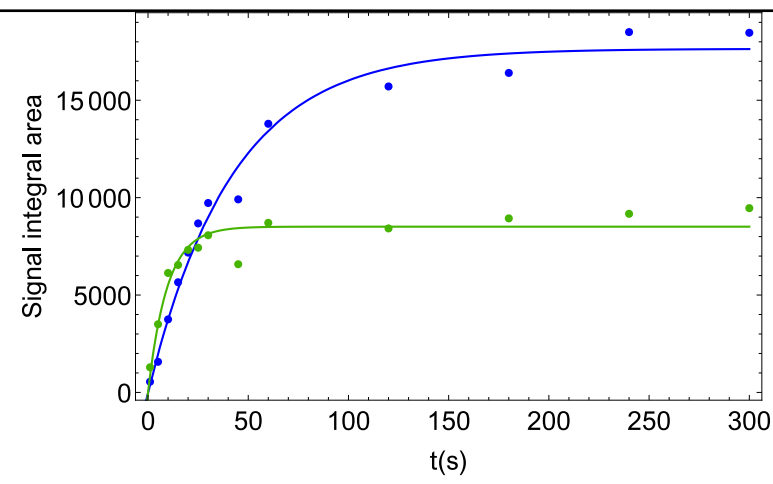

T$_b$(free)=41.9±3.2 s
T$_b$(3b)=9.5±1.4 s
T=280 K
</td>
<td></td>
</tr>
<tr>
<td>Ir-SIMesF₁</td>
<td>
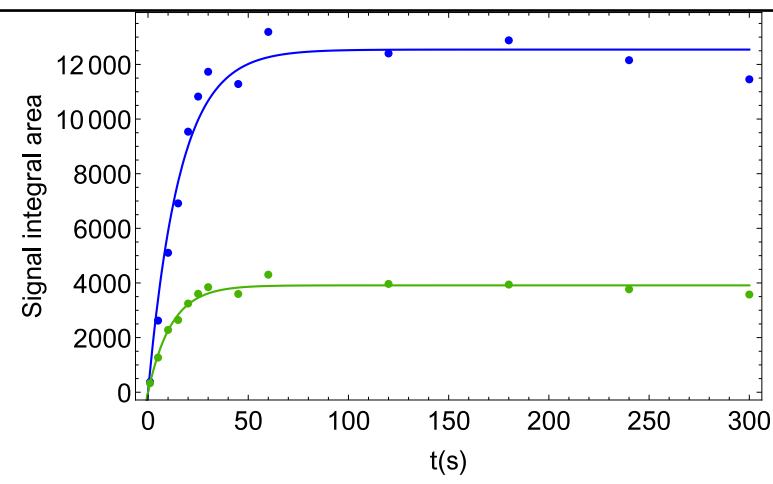

T$_b$(free)=15.8±1.5 s
T$_b$(3b)=11.6±1.0 s
T=280 K
</td>
<td>
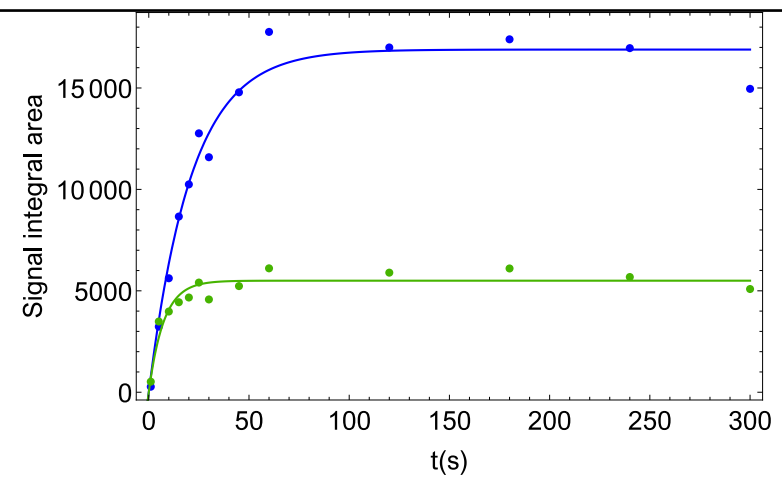

T$_b$(free)=21.2±1.9 s
T$_b$(3b)=7.4±1.9 s
T=272.5 K
</td>
</tr>
<tr>
<td>Ir-SIMesF₂</td>
<td>
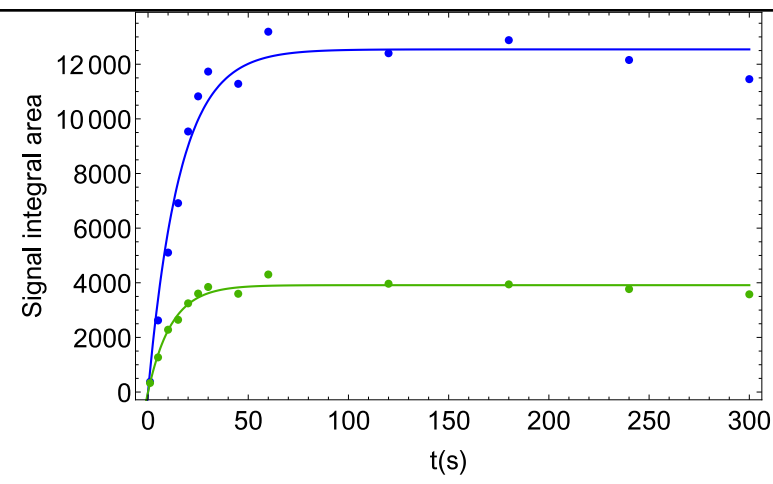

T$_b$(free)=12.2±2.5 s
T$_b$(3b)=6.0±2.0 s
T=280 K
</td>
<td></td>
</tr>
</table>

5.4. T_1 measurements

The carbon signal decay time constant of [1-^{13}C]pyruvate was determined experimentally both at the PTF of 0.35 μT field and at the magnetic field generated within the stack of ring magnets used during the Re-D protocol.

Hyperpolarization was achieved by bubbling for 25 s at 0.35 μT (SABRE-SHEATH conditions). For measurements at 0.35 μT the sample was let to evolve for a variable time before the read-out at 1.4 T. For the ring magnets, the sample was transferred to the corresponding field (∼>150 mT along the vertical axis and 10-50 mT on the transverse plane measured with a Gaussmeter AEGTEST, AEG-8103-2), allowed to evolve for a variable delay, and subsequently manually shuttled to the 1.4 T detection field for signal acquisition. A mono-exponential fitting function $S(t) = S_0 e^{-t/T_1}$ has been used for the T_1 estimation for both the free pyruvate and the **3b** bound form.

		Ir-IMesF$_1$ (s)	Ir-IPrF$_1$ (s)	Ir-SIMesF$_1$ (s)	Ir-IMes (s)	Ir-SIMesF$_2$ (s)
0.35 □T	$T_{1,a}$(free)	20.5±0.7	16.9 ± 1.4	48.7 ± 2.0	27.4 ± 0.7	10.9 ± 0.6
	$T_{1,a}$(**3b**)	9.4±0.4	8.8 ± 0.7	14.9 ± 2.0	9.1 ± 0.6	5.7 ± 0.4
Ring magnets	T_1(free)	82.0 ± 4.8	77.0 ± 3.0	95.3 ± 3.3	103.6 ± 4.3	74.6 ± 8.4
	T_1(**3b**)	86.5 ± 6.7	17.6 ± 1.5	72.5 ± 7.4	65.3 ± 8.5	50.2 ± 5.6

Table SI2: *Signal decay time constant $T_{1,a}$ measurements at 0.35 μT and T_1 at the magnetic field provided by ring magnets (∼>150 mT on the vertical axis and 10-50 mT on the transverse plane for each ring magnet).*

Depending on the catalyst, T_1 values at the ring-magnet field are longer by a factor of approximately 2–4 compared to the decay time constant measured at 0.35 μT. The data are summarized in Table SI2.

5.5. Extraction and Filtration

The procedure to obtain an aqueous solution with minimal residual iridium is described in the main text. Figure S75 shows the single-scan hyperpolarized spectrum recorded after completion of the Re-D, extraction, and filtration protocol for a sample initially containing 6 mM **Ir-IMesF$_1$**, 20 mM sodium [1-^{13}C]pyruvate, and 30 mM DMSO. The corresponding thermal equilibrium spectrum at 1.4 T (d1=240 s, ns=1024) is shown for reference. An enhancement of ∼5200 corresponding to a ^{13}C polarization level of 0.6% has been estimated (see SI Section 5 for polarization calculation).

The same protocol was applied to **Ir-IPrF$_1$**, **Ir-SIMesF$_1$**, and **Ir-SIMesF$_2$** and polarization values are reported in Fig. 2 in the main text. As outlined there, the Re-D, extraction, and filtration procedure employs two 5mm NMR tubes. Tube 1 is used for hyperpolarization of 100 μL of catalyst-containing solution. Following catalyst activation, hyperpolarization is performed by bubbling 50% parahydrogen at 6 bar and ∼80 sccm at 270–275 K, depending on the catalyst.

Tube 1 is then manually transferred to a region containing stacked ring magnets. The magnetic field was measured with a Hall probe to be >150 mT along the vertical axis, with transverse components varying in the range 10–50 mT for each magnet ring. Ethyl acetate (300 μL) and D_2O (400 μL) are subsequently injected into tube 1 and mixed by gentle hydrogen bubbling for 6 s. Phase separation into organic and aqueous layers occurs as shown in Fig. S75b.

Approximately 300 μL of the aqueous phase is withdrawn using a syringe connected to 1/16″ PTFE tubing and transferred into tube 2 positioned inside the benchtop NMR spectrometer. Prior to entering tube 2, the solution passes through a filter consisting of a short 6 mm OD PTFE cartridge packed with 50 mg of perfluorinated silica (see Fig. SI75b).

The photographs in Fig. SI75b show tube 1, tube 2, and the *home-made* perfluorinated silica filter used in the experiments. In tube 1, the organic and aqueous phases are clearly separated on opposite sides of the phase boundary. Tube 2 contains the filtered aqueous phase and is the same tube from which the representative hyperpolarized and thermal spectra shown in Fig. SI75a were acquired.

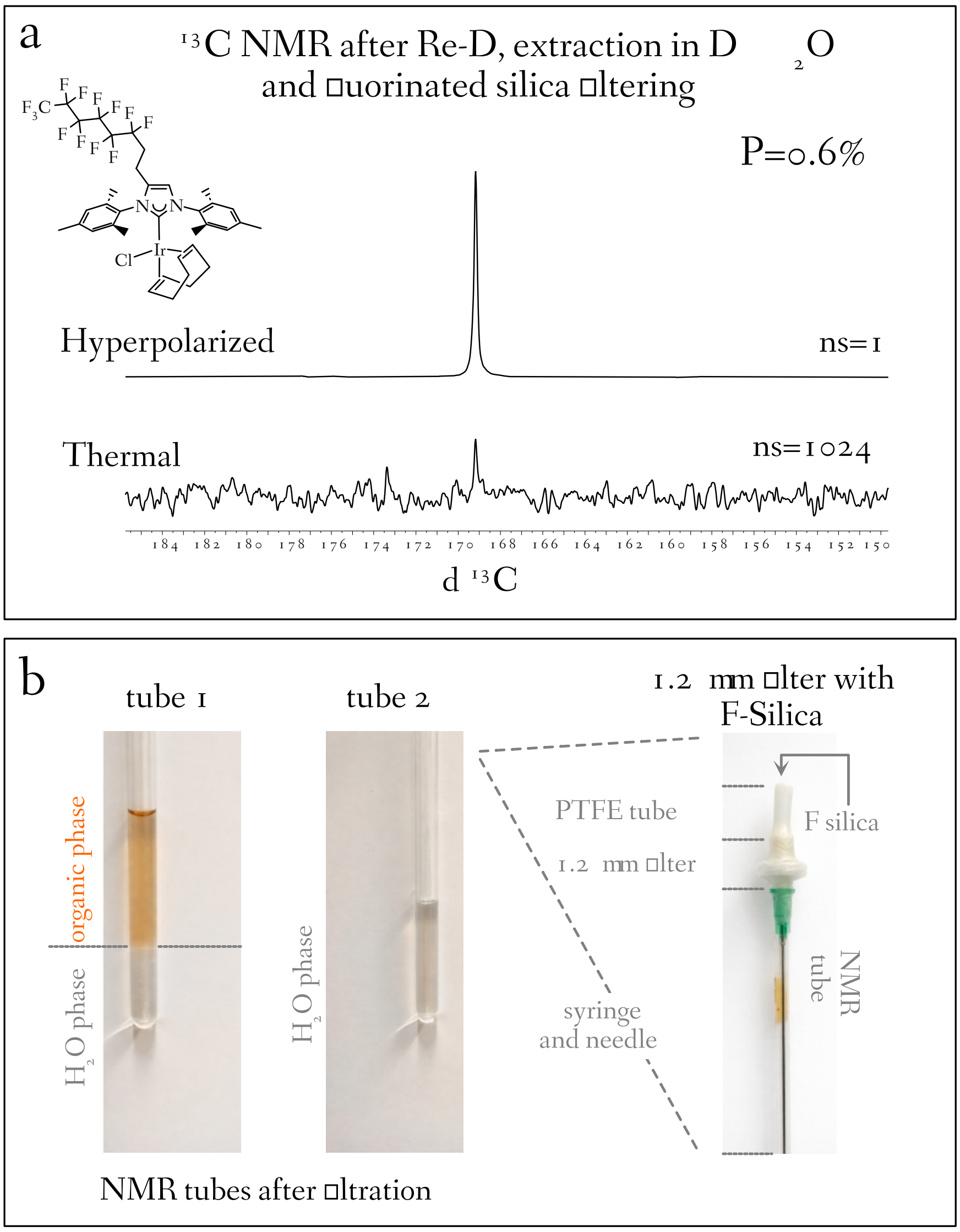

Figure S75: *a) ^{13}C NMR of an initial 6mM* ***Ir-IMesF$_1$****, 30 mM [1-^{13}C] pyruvate sodium and 40 mM DMSO after Re-D, extraction of the aqueous fraction (~300 μL) and filtering through a 1.2 μm Whatman filter filled with 150*

mg of perfluorinated silica. Hyperpolarized single-scan spectrum on top and thermal spectrum acquired with ns=1024 and d1=240 s at the bottom. The final elemental Ir content after the protocol was measured ICP MS and it is reported in SI 5.7.

5.6. Residual Iridium, pyruvate and solvents concentration in D_2O

After the hyperpolarization, extraction and filtration experiments via the revised Re-D SABRE protocol, the ^{13}C NMR spectra of the three **Ir-IMesF$_1$** replicates were measured on a 400MHz NMR spectrometer. For the quantification of residual solvents and pyruvate, we used the natural abundance carboxylic ^{13}C NMR signal of ethyl acetate as an internal standard, considering a solubility in D_2O of 83 g/L and assuming that the solution was saturated. ^{13}C natural abundance was taken as 1.07%.

**Ir-IMesF$_1$**	Ethyl Acetate	Methanol	DMSO	[1-^{13}C]pyruvate
Sample 1	0.94 M	3.6 M	n.d.	7.8 mM
Sample 2	0.94 M	1.7 M	< 2 mM	5.7 mM
Sample 3	0.94 M	3.7 M	n.d.	3.7 mM

5.7. ICP-MS data

At the end of the experiment, the aqueous phase was collected and diluted (1:200 for Ir-IMes samples; 1:20 for fluorinated catalyst samples) with distilled water, followed by acidification with concentrated HNO₃ to a final concentration of 3.5%.

	Catalyst	Ir (ppb)	Ir (ppb) sample	$\bar{x} \pm \frac{Max[x_i]-Min[x_i]}{2}$ (ppb)
1	**Ir-IMes**	13.5	2970	
2	**Ir-IMes**	27.8	6102.4	
3	**Ir-IMes**	64.5	14172.4	7748 ± 5600
4	**Ir-IMesF$_1$**	4.3	89.9	
5	**Ir-IMesF$_1$**	7.2	150.9	
6	**Ir-IMesF$_1$**	4.5	94.3	111.7 ± 30
7	**Ir-SIMesF$_2$**	7.9	167.0	
8	**Ir-SIMesF$_2$**	25.2	534.6	
9	**Ir-SIMesF$_2$**	16.2	341.9	347.8 ± 183.8
10	**Ir-IMesF$_1$** + fluorinated silica	8.3	173.8	

11	**Ir-IMesF₁** + fluorinated silica	2.8	58.4	
12	**Ir-IMesF₁** + fluorinated silica	2.7	56.4	96.2 ± 58.7

5.8. Polarization calculation

The polarization was calculated according to:

$$P = 1.15 \times 10^{-6} \times \frac{I_{hyperpolarized}}{I_{thermal}} \times \frac{ns_{thermal}}{ns_{hyperpolarized}}$$

Where $I_{hyperpolarized}$, $I_{thermal}$, $ns_{hyperpolarized}$, $ns_{thermal}$ are the integrals of the hyperpolarized and thermal signals and the number of scans of the hyperpolarized (always 1) and thermal signals respectively; 1.15×10^{-6} is the thermal ^{13}C polarization at 1.4 T and 40 °C. The thermal polarization is calculated via the standard expression $P_{th} = \tanh(\hbar\gamma B_0 / 2k_B T)$, where $\hbar$ is the reduced Planck constant, γ is the nuclear gyromagnetic ratio, k_B is the Boltzmann constant and T the temperature in the appropriate units. For the methanol-d_4 experiments, the reported total ^{13}C polarization was calculated from the summed integrated intensities of free pyruvate and the bound **3a** and **3b** species in the hyperpolarized and thermal-equilibrium spectra of the same sample.

We have also verified that the value of the carbon polarization can be estimated using an external reference represented by a sample of labelled sodium acetate at 2.5 M concentration.

We confirmed that polarization values determined using the external reference are consistent with those obtained from the ratio of the hyperpolarized and thermal-equilibrium signal intensities of the same sample. This approach was therefore used to quantify all polarization levels reported in the main text for the aqueous solutions obtained after the filtration process. In this case, the polarization was calculated according to:

$$P = 1.15 \times 10^{-6} \times \frac{I_{hyperpolarized}}{I_{reference}} \times \frac{c_{reference}}{c_{hyperpolarized}} \times \frac{ns_{thermal}}{ns_{hyperpolarized}}$$

In our case $I_{reference} = 19796.23$, $c_{reference} = 2.5$ M, $ns_{thermal} = 128$, $ns_{hyperpolarized} = 1$

5.9. LDH Solution for the In Vitro Conversion of Pyruvate to Lactate

To probe the pyruvate-to-lactate conversion in vitro, L-lactate dehydrogenase (LDH) from bovine heart (1000 units/mL) was diluted to 250 units/mL in phosphate-buffered saline containing 150 mM sodium phosphate, 150 mM NaCl, and 10 mM NADH. For each SABRE experiment, 200 µL of the LDH solution (corresponding to 50 units) was used.

The NMR tube containing the LDH solution was placed inside the benchtop NMR spectrometer and maintained at 40 °C for 10 minutes prior to the injection of the aqueous hyperpolarized pyruvate solution.

6. Characterization of SABRE active catalysts

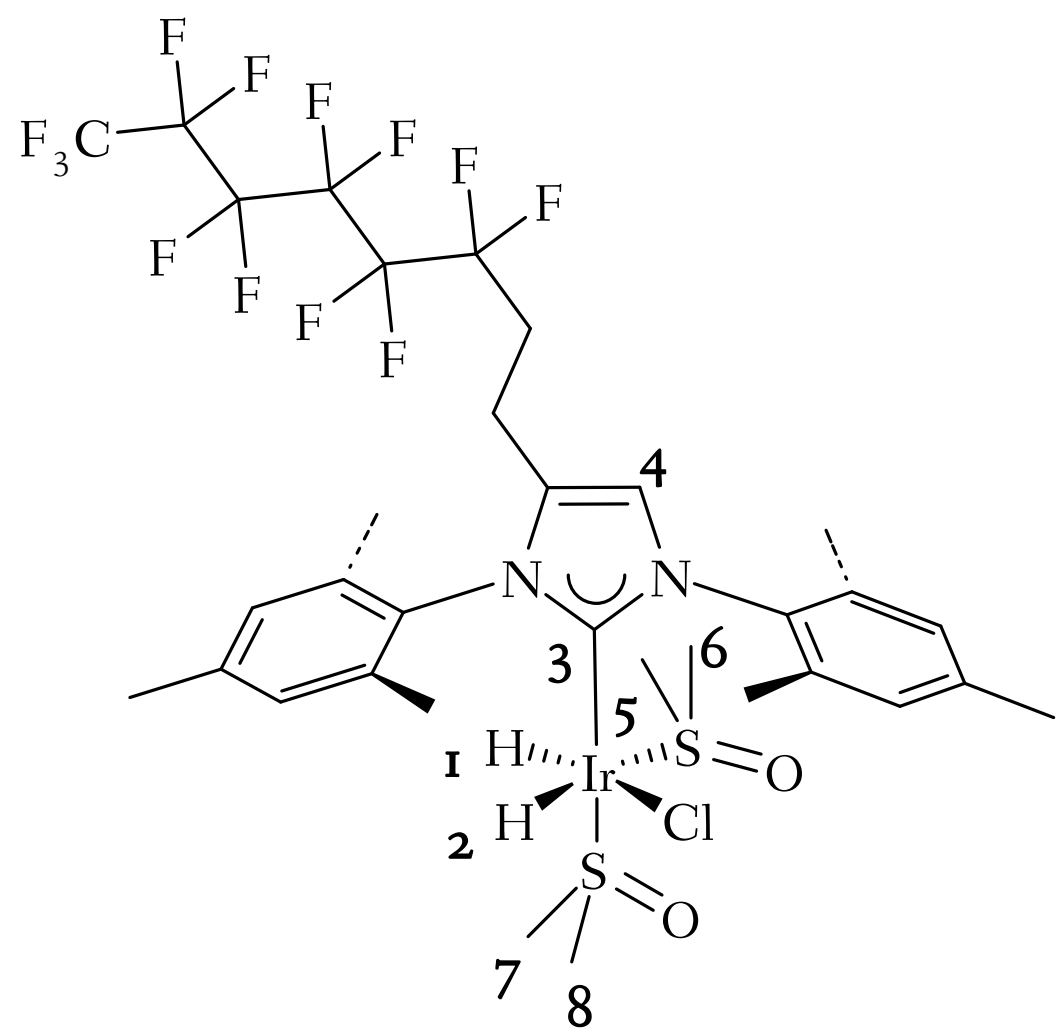

Figure S76: *Chemical structure of* **2** *determined from data given in Table SI3.*

Resonance Label	^{1}H	^{13}C
1	-21.60	-
2	-15.56	-
3	-	152.91
4	7.22	120.79
5	2.93	47.88
6	3.14	41.99
7	3.18	44.16
8	3.22	57.39

Table SI3: *NMR data for* **2** *shown in Figure S76. Data were collected at 263 K at a field of 9.4 T using methanol-d_4 as a solvent. Resonance labels are stated according to Figure S76.*

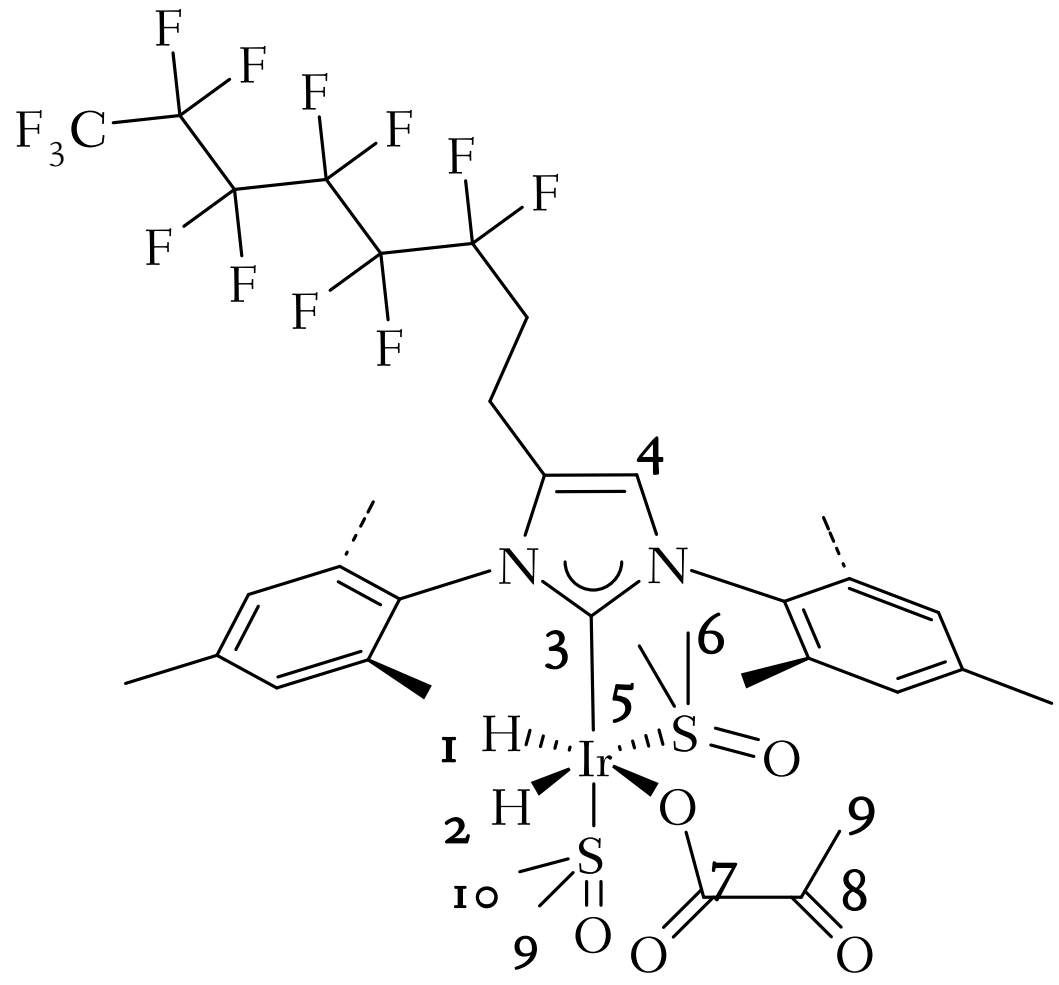

Figure S77: *Chemical structure of **3a** determined from data given in Table SI4.*

Resonance Label	^{1}H	^{13}C
**1**	-24.06	-
**2**	-14.95	-
**3**	-	154.04
**4**	7.15	120.14
**5**	2.90	45.98
**6**	3.35	56.73
**7**	-	165.10
**8**	-	196.52
**9**	2.17	17.66

Table SI4: *NMR data for **3a** shown in Figure S77. Data were collected at 263 K at a field of 9.4 T using methanol-d_4 as a solvent. Resonance labels are stated according to Figure S77.*

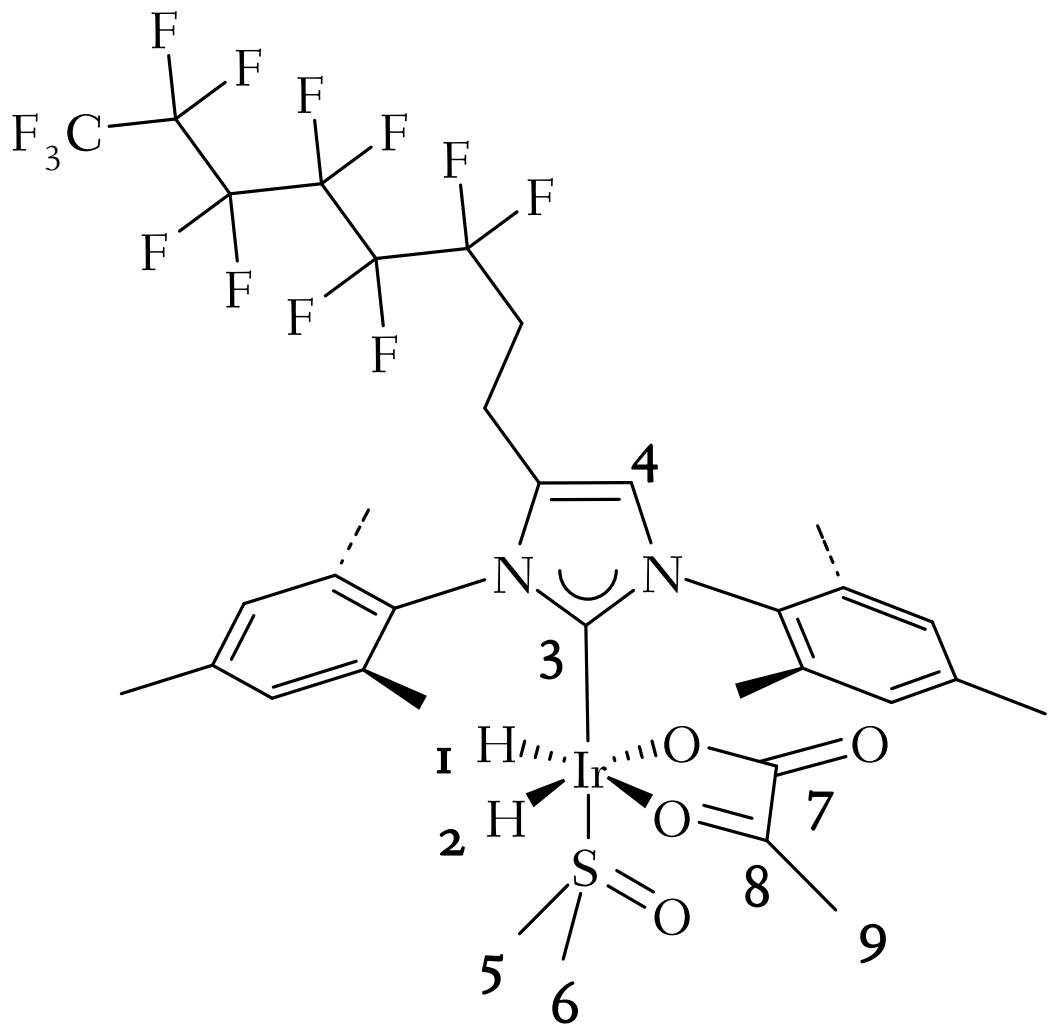

Figure S78: *Chemical structure of **3b** determined from data given in Table SI5.*

Resonance Label	^{1}H	^{13}C
1	-29.08	-
2	-27.14	-
3	-	160.92
4	7.19	120.02
5	2.93	51.49
6	3.06	48.50
7	-	172.00
8	-	206.38
9	2.05	16.70

Table SI5: *NMR data for **3b** shown in Figure S78. Data were collected at 263 K at a field of 9.4 T using methanol-d_4 as a solvent. Resonance labels are stated according to Figure S78.*

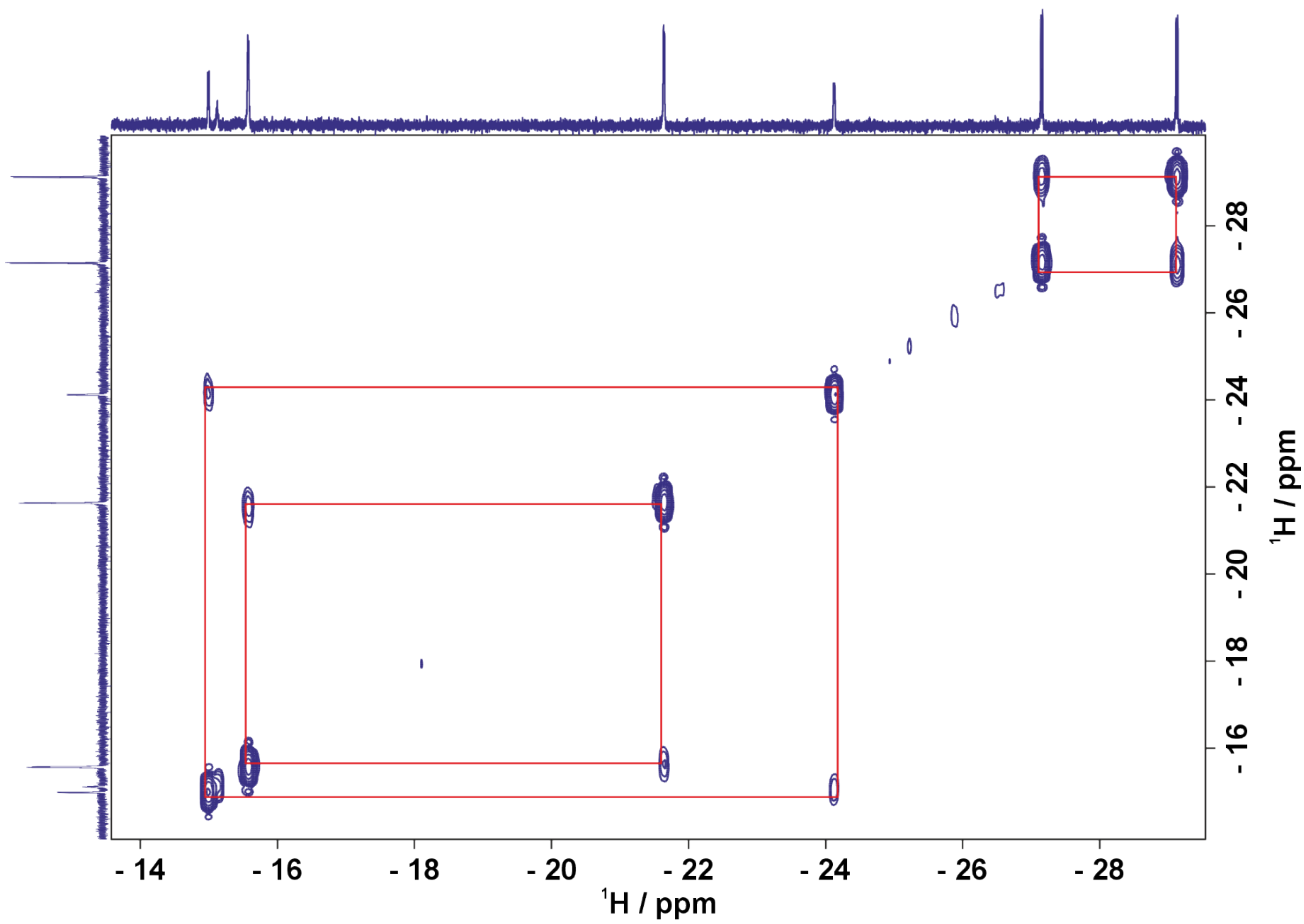

Figure S79: *^{1}H NMR COSY data collected from a 700 μL methanol-d$_4$ solution (30 mM* ***Ir-IMesF$_1$****, 150 mM DMSO. 100 mM sodium [2-^{13}C]pyruvate) under a 4 bar atmosphere of H_2 at a temperature of 263 K and an acquisition field of 9.4 T.*

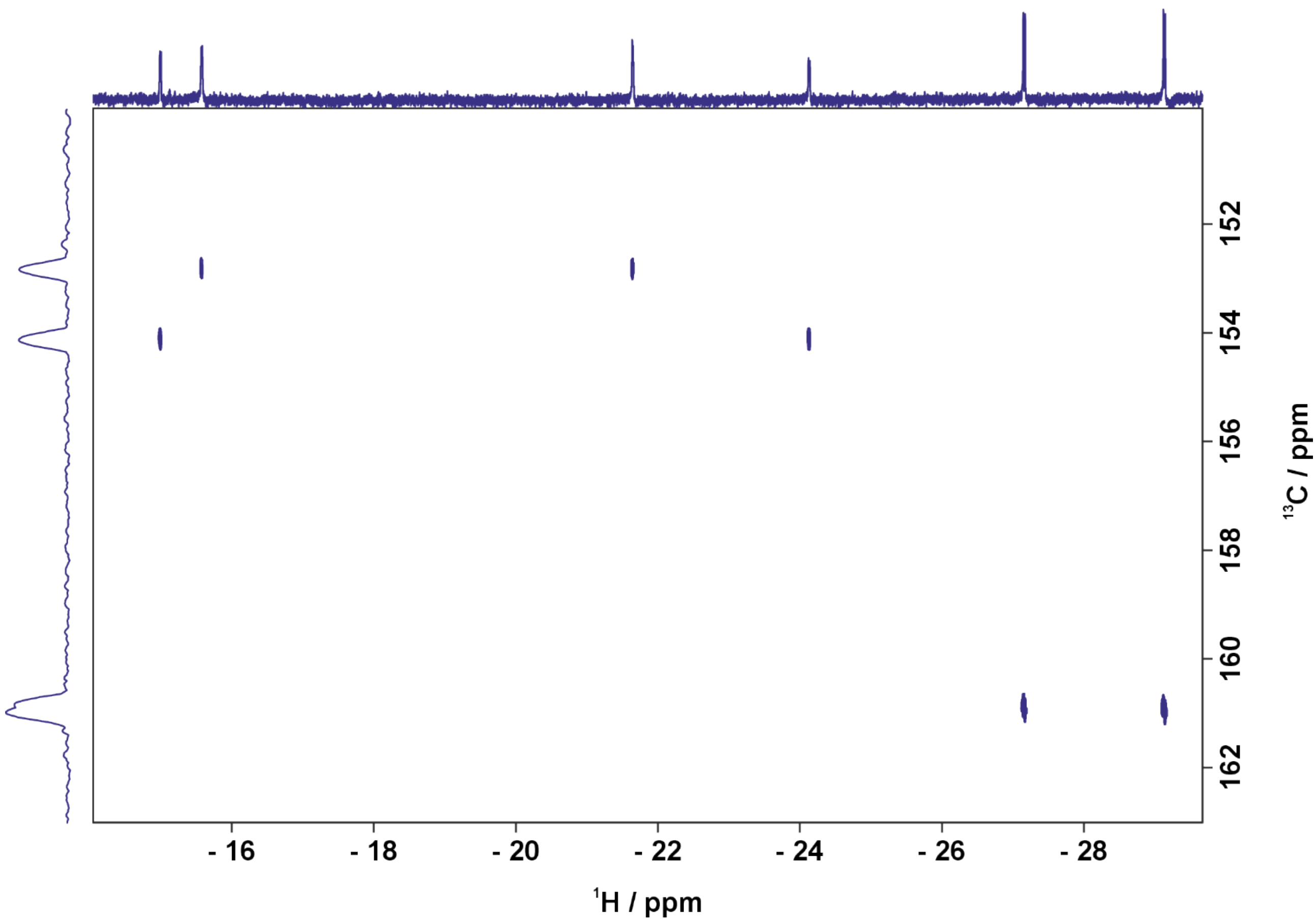

Figure S80: *^{1}H-^{13}C HMQC NMR data collected from a 700 μL methanol-d$_4$ solution (30 mM* ***Ir-IMesF$_1$****, 150 mM DMSO. 100 mM sodium [2-^{13}C]pyruvate) under a 4 bar atmosphere of H_2 at a temperature of 263 K and an acquisition field of 9.4 T. A coupling constant of 12 Hz was used.*

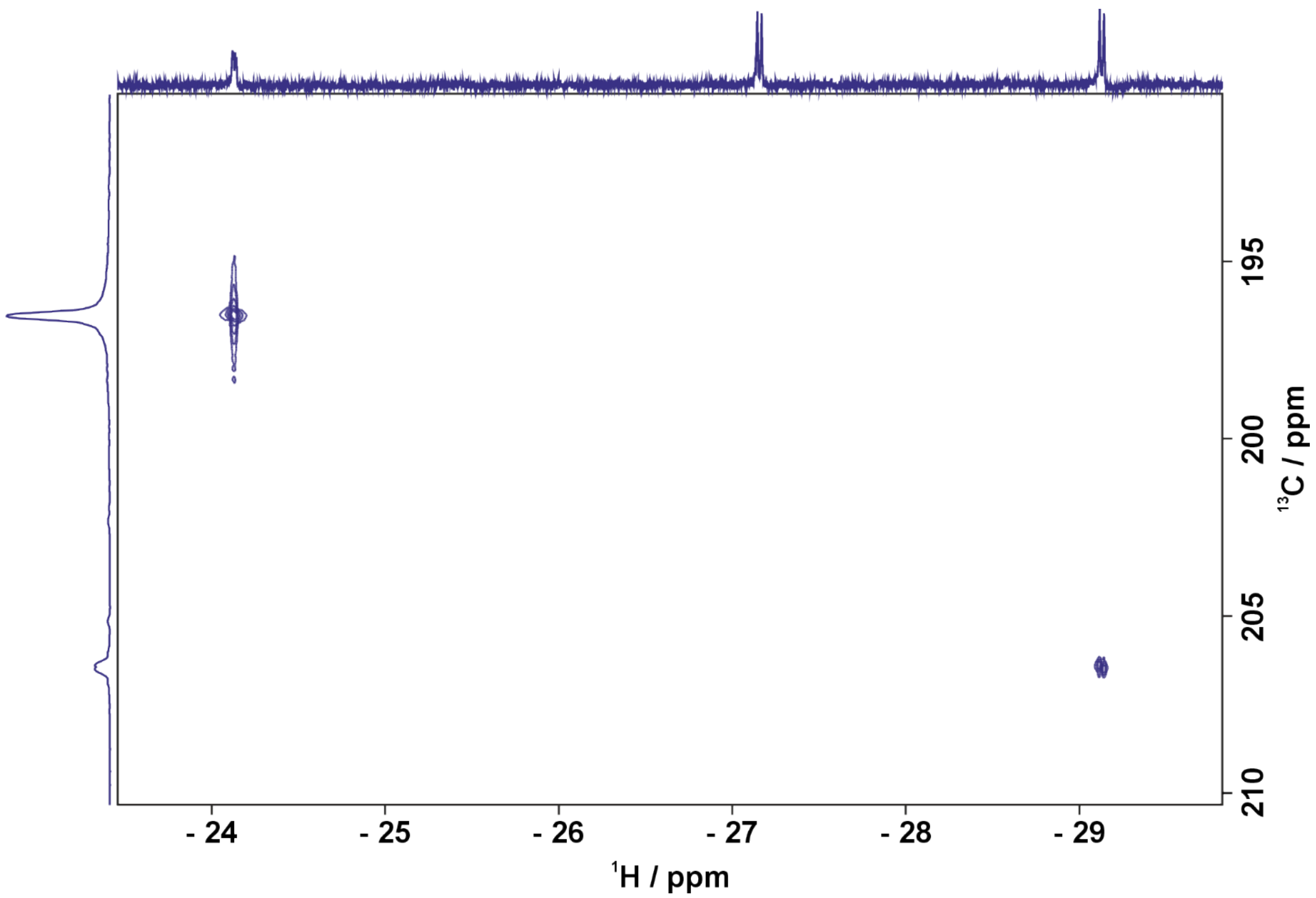

Figure S81: *$^{1}H^{13}C$ HMQC NMR data collected from a 700 μL methanol-d_4 solution (30 mM **Ir-IMesF$_1$**, 150 mM DMSO. 100 mM sodium [2-^{13}C]pyruvate) under a 4 bar atmosphere of H_2 at a temperature of 263 K and an acquisition field of 9.4 T. A coupling constant of 12 Hz was used.*

References

(1) D. P. Curran, S. Hadida and H. Mu, J. Org. Chem., **1997**, 62, 6714-6715. https://doi.org/10.1021/jo9710590

(2) Clavier, H.; Correa, A.; Cavallo, L.; Escudero-Adán, E. C.; Benet-Buchholz, J.; Slawin, A. M. Z.; Nolan, S. P. [Pd(NHC)(Allyl)Cl] Complexes: Synthesis and Determination of the NHC Percent Buried Volume (% V_{bur}) Steric Parameter. *Eur J Inorg Chem* **2009**, *2009* (13), 2007–2007. https://doi.org/10.1002/ejic.200900292.

(3) Hošek, J.; Rybáčková, M.; Čejka, J.; Cvačka, J.; Kvíčala, J. "Synthesis of Heavy Fluorous Ruthenium Metathesis Catalysts Using the Stereoselective Addition of Polyfluoroalkyllithium to Sterically Hindered Diimines" Organometallics **2015**, 34, 3327–3334. https://doi.org/10.1021/acs.organomet.5b00325

(4) Ettedgui, J.; Blackman, B.; Raju, N.; Kotler, S. A.; Chekmenev, E. Y.; Goodson, B. M.; Merkle, H.; Woodroofe, C. C.; LeClair, C. A.; Krishna, M. C.; Swenson, R. E. Perfluorinated Iridium Catalyst for Signal Amplification by Reversible Exchange Provides Metal-Free Aqueous Hyperpolarized [1-13C]-Pyruvate. *J. Am. Chem. Soc.* **2024**, *146* (1), 946–953. https://doi.org/10.1021/jacs.3c11499.

(5) *SAINT*, **2025**, *V8.42*, Bruker AXS SE.

(6) L. Krause, R. Herbst-Irmer, G. M. Sheldrick, D. Stalke, *J. Appl. Cryst.* **2015**, *48*, 3–10, doi:10.1107/S1600576714022985.

(7) G. M. Sheldrick, *Acta Cryst.* **2015**, *A71*, 3–8, doi:10.1107/S2053273314026370

(8) G. M. Sheldrick, *Acta Cryst.* **2015**, *C71*, 3–8, doi:10.1107/S2053229614024218.

(9) Franco, L.; Floreani, F.; Mamone, S.; Faramawy, A. M.; Ruzzi, M.; Tubaro, C.; Stevanato, G. A Controlled System for Parahydrogen Hyperpolarization Experiments. *Molecules* **2025**, *30* (21), 4299. https://doi.org/10.3390/molecules30214299.
